# Amazonian Amphibian Diversity Is Primarily Derived from Late Miocene Andean Lineages

**DOI:** 10.1371/journal.pbio.1000056

**Published:** 2009-03-10

**Authors:** Juan C Santos, Luis A Coloma, Kyle Summers, Janalee P Caldwell, Richard Ree, David C Cannatella

**Affiliations:** 1 Section of Integrative Biology and Texas Natural Science Center, The University of Texas, Austin, Texas, United States of America; 2 Museo de Zoología, Escuela de Biología, Pontificia Universidad Católica del Ecuador, Quito, Ecuador; 3 Department of Biology, East Carolina University, Greenville, North Carolina, United States of America; 4 Sam Noble Oklahoma Museum of Natural History and Department of Zoology, University of Oklahoma, Norman, Oklahoma, United States of America; 5 Botany Department, Field Museum of Natural History, Chicago, Illinois, United States of America; University of California Berkeley, United States of America

## Abstract

The Neotropics contains half of remaining rainforests and Earth's largest reservoir of amphibian biodiversity. However, determinants of Neotropical biodiversity (i.e., vicariance, dispersals, extinctions, and radiations) earlier than the Quaternary are largely unstudied. Using a novel method of ancestral area reconstruction and relaxed Bayesian clock analyses, we reconstructed the biogeography of the poison frog clade (Dendrobatidae). We rejected an Amazonian center-of-origin in favor of a complex connectivity model expanding over the Neotropics. We inferred 14 dispersals into and 18 out of Amazonia to adjacent regions; the Andes were the major source of dispersals into Amazonia. We found three episodes of lineage dispersal with two interleaved periods of vicariant events between South and Central America. During the late Miocene, Amazonian, and Central American-Chocoan lineages significantly increased their diversity compared to the Andean and Guianan-Venezuelan-Brazilian Shield counterparts. Significant percentage of dendrobatid diversity in Amazonia and Chocó resulted from repeated immigrations, with radiations at <10.0 million years ago (MYA), rather than in situ diversification. In contrast, the Andes, Venezuelan Highlands, and Guiana Shield have undergone extended in situ diversification at near constant rate since the Oligocene. The effects of Miocene paleogeographic events on Neotropical diversification dynamics provided the framework under which Quaternary patterns of endemism evolved.

## Author Summary

The Neotropics, which includes South and Central America, contains half of remaining rainforests and the largest reservoir of amphibian diversity. Why there are so many species in certain areas and how such diversity arose before the Quaternary (i.e., more that 1.8 million years ago [MYA]) are largely unstudied. One hypothesis is that the Amazon Basin was the key source of diversity, and species dispersed from there to other areas. Here, we reconstruct a time-calibrated phylogeny and track, in space and time, the distribution of the endemic and species-rich clade of poison frogs (Dendrobatidae) during the Cenozoic (more than 65 MYA) across the continental Neotropics. Our results indicate a far more complex pattern of lineage dispersals and radiations during the past 10 MY. Rather than the Amazon Basin being the center of origin, our results show that the diversity stemmed from repeated dispersals from adjacent areas, especially from the Andes. We also found a recurrent pattern of colonization of Central America from the Chocó at 4–5 MY earlier than the formation of the Panamanian Land Bridge at 1.5 MYA. Thus, the major patterns of dispersals and radiations in the Neotropics were already set by ∼5–6 MYA (the Miocene–Pliocene boundary), but the ongoing process of Neotropical radiation is still happening now, especially in the Chocó–Central America region and Amazonian rainforest.

## Abstract 3

Phylogenetic analysis and ancestral range modeling of the poison-frog clade (Dendrobatidae) indicates that Amazonian species richness derives from repeated dispersals from adjacent regions, especially the Andes.

## Introduction

Tropical regions contain more than half of biological diversity on just 7% of the Earth's surface [1,2]. Differences in biodiversity between tropical and temperate regions have been attributed to contrasting speciation and extinction rates [3]. Within the Neotropical realm, the Amazon Basin and the Chocoan region contain half of Earth's remaining rainforests and one of the largest reservoirs of terrestrial biodiversity. However, the impact of pre-Quaternary ecogeographic constraints on Neotropical biodiversity is largely unknown and the mechanisms contributing to species richness are unclear [3,4]. For example, the well-documented high α−diversity (species richness) of the flora and fauna of the Amazon rainforest [5] is usually attributed to local geoclimatic dynamics that promote monotonic accumulation of lineages [6,7]. However, the lower β−diversity (species turnover relative to distance) within the Amazon Basin is puzzling [8] and vastly underestimated. Current hypotheses are based on restricted, mostly Quaternary, spatiotemporal scales involving paleogeographic or ecological events (e.g., riverine barriers, Pleistocene climate change) [3], persistence of conservative niches [9], and analyses of phylogeography and endemicity [10]. In addition to speciation/extinction processes [3], major paleogeological events promote diversification, yielding complex phylogenetic patterns of vicariance, dispersal, and secondary sympatry [6]. Using phylogeographic analyses of the endemic and diverse clade of poison frogs (Dendrobatidae), we reconstructed Neotropical biogeography from the Oligocene to the present and revealed a widespread and highly dynamic pattern of multiple dispersals and radiations during the Miocene.

**Figure 1:**
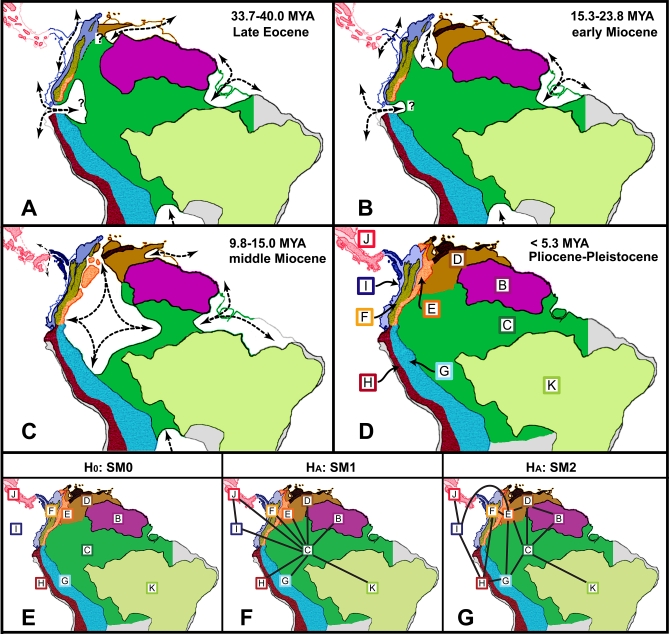
Biogeographic Areas, Extension of the Floodbasin System or Marine Incursions (Hatched Arrows), and Possible Connections within Cenozoic Paleogeographic Maps of Central and South America, Modified after Hoorn et al. and Diaz de Gamero (A–D) correspond to paleogeographic reconstruction of northern South America. (A) Early to late Eocene; (B) late Oligocene to middle Miocene; (C) middle to late Miocene; and (D) early Pliocene to the present. Uncertainties about the limit between biogeographic regions before the Pliocene are indicated by “?”. The hypotheses about the spatial configuration of biogeographic areas on the origin of Neotropical diversity are indicated in the panels (E–G) (input hypothesis matrices are provided in Figure S6). (E) The null model (H_0_: SM0), which assumes no spatial structure and equal rates of dispersal among all areas; (F) the center-of-origin model (H_A_: SM1), which assumes the Amazonia as the primary center of origin with widespread ancestral ranges constrained to include the Amazon Basin; and (G) the stepping-stone model (H_A_: SM2), which the assumes the historical spatial arrangement of biogeographic areas and constrains dispersals among geographically adjacent areas. The biogeographic areas used in the DEC modeling are C, the Amazon Basin; B, Guiana Shield; D, Venezuelan Highlands and Trinidad and Tobago Islands; E, North Oriental Andes; F, North Occidental Andes; G, Central Oriental Andes; and H, Central Occidental Andes; I, Chocoan rainforest, Magdalena Valley, and Gorgona Island; J, Central America west of the Gatun Fault Zone in Panamá; and K, the Brazilian Shield [14, 90].

Major geoclimatic events have shaped the Neotropics. The most important include the isolation and reconnection of South America, the uplift of the Andes, the extensive floodbasin system in the Amazonian Miocene, the formation of Orinoco and Amazon drainages, and the dry−wet climate cycles of the Pliocene−Pleistocene (Figure 1). The Panamanian Land Bridge (PLB) between the Chocó and Central America, which formed progressively until the Pliocene [11], was an important biogeographic catalyst of dispersal and vicariance events at the Miocene−Pliocene boundary (e.g., *Alpheus* shrimps and freshwater teleost fishes) [12,13]. Similarly, the uplift of the Andes advanced the formation of the Amazon River, converting a widespread, northwest-flowing Miocene floodbasin into the current eastward-running Amazon Basin [14,15]. Two Miocene marine incursions into this wetland system isolated several aquatic taxa as living relicts, including the Amazon River dolphin, lineages of marine-derived teleosts and stingrays, and brackish water mollusks [16,17]. However, controversies exist about the magnitude and duration of these geoclimatic events [18].

Although well known for its megadiversity, no studies of the Neotropics have examined diversification patterns in highly speciose and widespread lineages over broad temporal and spatial scales. A general explanation that associates rates of speciation with paleogeographic events is lacking. Here, we test two general hypotheses about the spatial configuration of biogeographic areas on the origin of Neotropical diversity (Figure 1). First, under the center-of-origin hypothesis, lineages from the currently most diverse area (i.e., Amazon Basin) dispersed to other areas (Figure 1, H_A_: SM1). Second, under the stepping-stone hypothesis, paleogeographic events constrained the patterns of lineage diversification in the Neotropics among geographically adjacent areas (Figure 1, H_A_: SM2). Using a recently developed maximum likelihood (ML) procedure that estimates geographic range evolution, we tested both hypotheses against a null biogeographic model (Figure 1 H_0_: SM0) using a well-sampled Neotropical clade, the poison frogs (Dendrobatidae). We sampled 223 of the ∼353 (264 described and 34–89 undescribed) species, distributed from Central America and Guiana Shield to southeast Brazil and from Andean páramos (4,000 meters above sea level [masl]) to lowland rainforests (<300 masl). However, ∼40% of the species diversity remains unsampled (Table S11). Because the true diversity (i.e., described, undescribed, and extinct species) cannot be accurately assessed [19,20], macroevolutionary inference should account for missing diversity. Our goals are to (1) infer how geographic range evolution yielded current species distributions, (2) estimate the general patterns of speciation and extinction under necessarily incomplete taxon sampling, and (3) synthesize these findings with paleogeographic events to explain current patterns of species richness.

## Results

**Figure 2:**
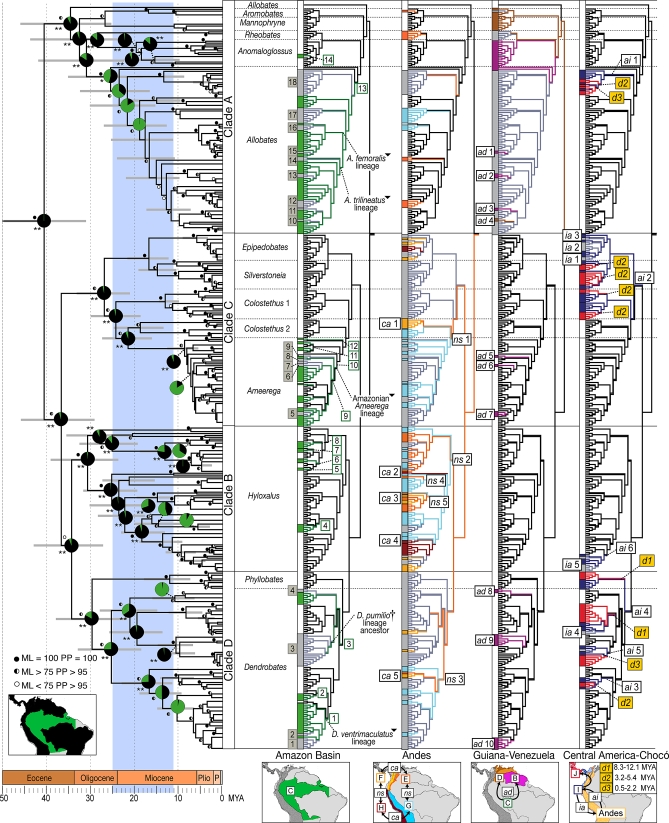
Chronogram and Major Biogeographic Events of the Poison Frogs Divided by Major Regions and Inferred from the DEC Analysis The green slice of the pie charts is the proportion of the likelihood associated with Amazonian reconstruction using Bayesian analysis of ancestral traits. The test results against the null hypothesis of an Amazonian ancestral state are indicated as *, *p <* 0.05, significant, and **, *p <* 0.001, highly significant. In the chronogram, support values from 200 nonparametric bootstrap replicates (ML), Bayesian posterior probabilities (PP), and the 95% CI of the estimated node age are also indicated. The major geographical events reconstructed using the SM2 model were mapped onto the ML phylogeny. The Amazon Basin subtree includes dispersals into Amazonia (numbered white squares), out of Amazonia (numbered gray squares), major lineage diversifications (5), and a lineage extinction (†). The Andes subtree includes cross-Andean (*ca* boxes) events, Northern-Southern Andean (*ns* boxes) dispersals, and the identification number of each event (see Table S12). The Guiana-Venezuela subtree includes dispersals toward this region from the Amazon Basin lineages (*ad* boxes) and the identification number of each event (see Table S12). The Central America-Chocó subtree indicates the three dispersals from Chocó and Central America (*d1*, *d2*, and *d3* yellow boxes), dispersals from the Andes to the Chocó (*ai* boxes), from the Chocó to the Andes (*ia* boxes) and the identification number of each event (see Table S12). For all subtrees, the tree branches and the rectangles at the tips of each tree are color coded as follows: (1) gray rectangles connected by gray branches mean extant lineages distributed in other areas different from the subtree region, but that descend from an ancestor distributed in the exemplified subtree region; (2) white rectangles connected by black branches indicate extant lineages distributed in other areas different from the subtree region; (3) color-coded rectangle and branches indicate extant lineages distributed in the exemplified subtree region. The duration of the Miocene floodbasin systems is indicated by blue-gray area. For detailed ancestral area reconstructions and date estimates with confidence intervals see Table S8.

**Figure 3:**
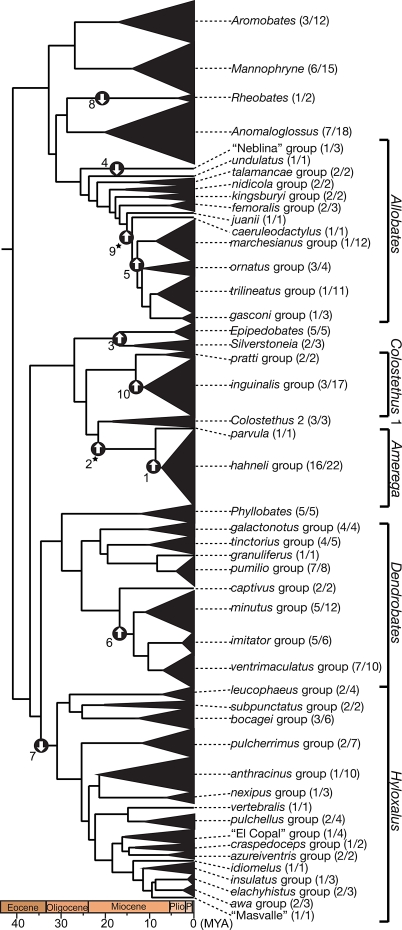
The Tree GSPF Level Tree Chronogram of Poison Frogs with Known Taxonomic Diversity (i.e., Numbers within Parentheses) and Significant Diversification Rate Changes for Nodes or Lineages from Table 3 Taxonomic diversity is indicated by the species sampled in our study (left number) and the total number of species described per group or genus (right number) (upward arrow indicates rate increase, and downward indicates rate decrease). The highest diversification increase (node 1) corresponds to the widespread *Ameerega* lineage that rapidly expanded into Amazonian since the late Miocene. The Andean-Amazonian increase (node 2) corresponds to the diversification of clade *Colostethus* 2 in the early Miocene. The Chocoan increases (nodes 3 and 10) correspond to the lowland radiation in western Colombian and Ecuadorian lowlands of *Colostethus* sensu lato, *Epipedobates*, and *Silverstoneia* during the middle Miocene and before the formation of the PLB. The Guiana decrease in the early Miocene (node 4) corresponds to the *Allobates* lineage of the Guianan tepuis. The Amazonian rate increases (nodes 5 and 9) correspond the largest radiation of *Allobates* since the Miocene to the Pliocene. The Andean-Amazonian increase (node 6) corresponds to the diversification of clade *Dendrobates* from the eastern Central Andean foothills into Amazonia since the early Miocene. The Andean decreases (nodes 7 and 8) correspond to a slow down of the diversification of *Hyloxalus* (clade B) and *Rheobates* (clade A) lineages during the late Oligocene and Pliocene, respectively. The significant increases indicated by a star next to nodes 2 and 9 might be dependent on deeper nodes (i.e., 1 and 5, respectively) and produced by a “trickle-down effect” (see “Lineage Diversification” in Results).

We reconstructed a ML phylogeny from an extensive taxon sample (Figure S3A–S3D), and a relaxed-clock Bayesian chronogram from a subsample of the previous [21]. The phylogeny is in general agreement with previous studies [22,23], and the significance of branch support is provided in Figure 2 (for detailed support values see Figure 3A–3D). Although our taxon sampling is the most extensive for poison frogs (i.e., ∼63.5% of the diversity in the family), we were concerned that incomplete taxon sampling might cause tree imbalance and introduce bias into divergence time estimation (Table 1) [24,25]. We assessed imbalance by testing the null model that each branch has an equal probability of splitting (Equal Rate Markov or ERM) using three tree imbalance tree indices: Colless *I_C_*, Sackin *I_S_*, and likelihood shape *s* (Table S1; see Materials and Methods). Overall, our results showed that the imbalance indices for all trees (i.e., ML, chronogram, and super-region phylogenies) have a tendency to depart from, but do not reject, the equal rates model (ERM). Therefore, our inferences about macroevolutionary events (e.g., ancestral area reconstruction, divergence times, and diversification rates) should not be affected significantly by incomplete taxon sampling.

**Table 1:**
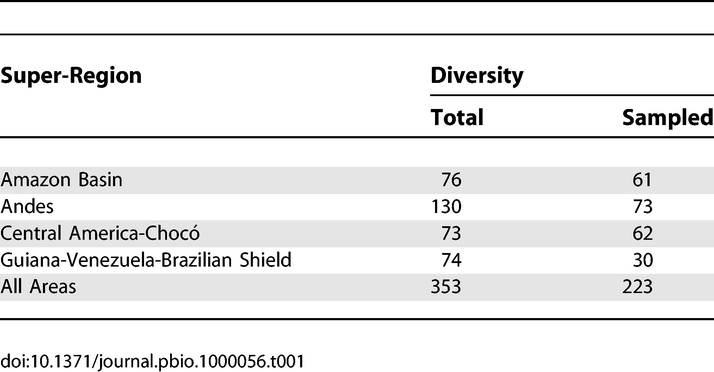
The Total Taxonomic Diversity (Described and Undescribed Species) and the Number of Species Sampled in the Poison Frog Chronogram and Super-Regions

**Table 2:**
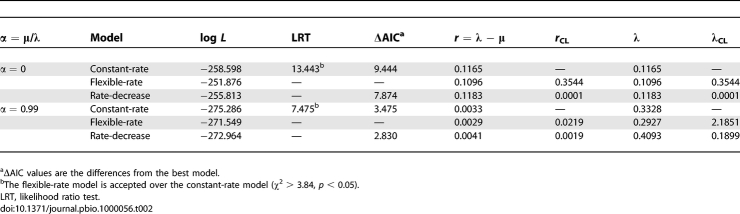
Diversification Rate Estimates and Fit under Constant, Flexible, and Rate Decrease Model in Poison Frogs

**Table 3:**
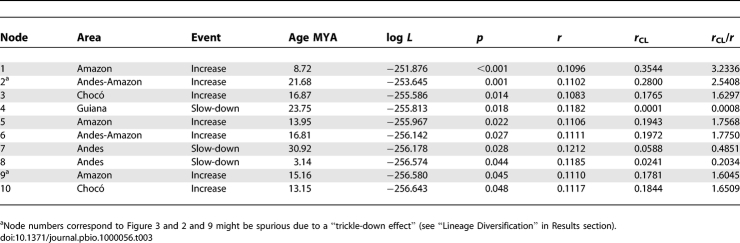
Significant Shifts in Diversification Rate under the Flexible-Rate Model with the Lowest Extinction Fraction (α = 0)

### Historical Biogeography of the Poison Frogs

The patterns of spatial and temporal distribution of poison frogs were inferred using dispersal-extinction-cladogenesis (DEC) modeling [26]. We compared three DEC models (i.e., a null and two alternatives) for ten areas (Figures 1 and S6). The null model (SM0) assumes that spatial structure has no effect on biogeographic patterns of evolution. The alternative models either favor the Amazon Basin as a center-of-origin (SM1), or patterns of dispersal/vicariance that reflect the spatial arrangement or connectivity of biogeographic areas (a stepping-stone model; SM2). Our results strongly support the SM2 model over SM1 (large phylogeny Δ*ln*[*L*] = 140.5, *p <* 0.001; chronogram Δ*ln*[*L*] = 48.6, *p <* 0.001) and SM0 (large phylogeny Δ*ln*[*L*] = 17.7, *p <* 0.001; chronogram Δ*ln*[*L*] = 0.5^NS^, *p* > 0.05). The nonsignificant comparison of SM2 and SM0 for the chronogram alone is likely due to its reduced taxon sample.

The chronogram and a summary of the significant biogeographic events with confidence limits (Tables S8, S9A, and S9B) from the stepping-stone model are superimposed on the four major clades (Figure 2). The most recent ancestor of Dendrobatidae was distributed in regions that correspond to the current Venezuelan Highlands and Northern Oriental Andes at 40.9 ± 5.4 million years ago (MYA). This ancestral range split into a Venezuelan Highlands ancestor of clade A and an Andean ancestor (clade B + C + D). The most recent ancestor of each major clade occurred during the Oligocene at 34.9 ± 5.4 MYA (clade A), 30.9 ± 3.9 MYA (clade B), 27.1 ± 3.2 MYA (clade C), and 29.9 ± 4.0 MYA (clade D). We inferred 14 dispersals into and 18 from the Amazon Basin to adjacent areas, including three major radiations and a single lineage extinction within Amazonia. We also found five cross-Andean dispersals, five dispersals from Northern to Central Andes, six dispersals from Northern Andes to Chocó, four dispersals from Chocó to the Andes, and three temporal phases of lineage dispersal with two interleaved periods of vicariant events between the Chocó and Central America (Figure 2 and Table S12).

The diversity of Amazonian poison frogs (>70 species) resulted from 14 separate dispersals into this region, in three phases (Figure 2). First, the two oldest dispersals originated independently from the Guiana Shield (23.8 MYA) and from the developing Andes (21.1 MYA), just before and during the existence of the Amazonian Miocene floodbasin. Second, a single dispersal from the Guiana Shield occurred 15.5 MYA, during a low sea-level period associated with reduction of the Miocene floodbasin system. Third, the 11 remaining dispersals from the Andes took place between (1.6–7.3 MYA) during the formation of the modern Amazon Basin river system. Ancestral area reconstructions using a Bayesian multistate procedure similarly support the recent multiple dispersals to the Amazon Basin (Figure 2). Thus, our results suggest that much of the extant Amazonian biodiversity results from relatively recent immigration of distinct lineages followed by in situ radiation during the last 10 MYA.

At least 18 dispersals from the Amazon Basin to other areas were found in three temporal phases. First, the earliest dispersals to the developing Chocoan lowlands (21.8 MYA) and the Andes (15.2 MYA) occurred during the establishment of the Miocene floodbasin system. Interestingly, for the present Chocoan lineage of Dendrobates pumilio (Figure 2), our results suggest a Miocene overwater dispersal from Chocó to the developing Central America archipelago and the extinction of the Amazonian lineage ancestor at ∼19.5 MYA during the formation of the Miocene floodbasin system. Second, dispersals to the Guiana Shield (1), the Venezuela Highlands (1), and the Andes (1) took place after the Miocene floodbasin system receded (8.8–10.8 MYA). Third, the 12 remaining dispersals were very recent (0.7–6.0 MYA), to the Guiana Shield (7), Andes (4), and Brazilian Shield (1). Thus, 16 out of 18 occurred <11 MYA, after the establishment of the current Amazonian geomorphology (Figure 2).

At least four major diversifications occurred within Amazonia: (1) the Allobates trilineatus complex (26 species) is the oldest (14.0–15.1 MYA); the three remaining are more recent (5.4–8.7 MYA), (2) the Amazonian *Ameerega* (19 species); (3) the Dendrobates ventrimaculatus complex (15 species); and (4) the Allobates femoralis complex (nine species). Moreover, all four lineages entered the Guiana Shield area in the Pliocene (Figure 2).

Species diversity in the Andes (71 species) resulted from a continuous diversification process since the late Eocene (Figure 2). However, several Andean events were contemporaneous with establishment of the Amazon Basin. Five cross-Andean dispersals from Oriental to Occidental Andes (2.0–25.4 MYA), five dispersals from Northern to Central Andes (14.9–30.9 MYA), and six dispersals from the Northern Andes to the Chocoan lowlands (8.3–29.9 MYA) (Figure 2) took place before the establishment of the Andes as a major geographic barrier during the Miocene–Pliocene boundary [27]. We also found five dispersals from the Chocó to North and Central Andes (1.1–6.6 MYA) (Figure 2) that took place mostly in the Pliocene when the Andes were already established as a barrier. Our results indicate that lower montane transition zones between Andean and lowland environments (Chocó and Amazonia) promote diversification, as exemplified by the Amazonian *Ameerega* [28] and the Chocoan *Epipedobates*.

Central American and Chocoan species (>40) also show a complex pattern of diversification at the end of the Miocene. Ten dispersals from Chocó to Central America suggest a pattern of recurrent colonization and isolation in three phases (Figure 2). First, the two oldest dispersals (8.3–12.1 MYA) from the Chocó overlap with a proposed earlier exchange of faunas during the late Miocene [29]. A single vicariant event at 6.8 MYA isolated the first wave of immigrants (i.e., ancestors of *Phyllobates* and *Colostethus* 1). Interestingly, the contemporaneous divergence of the Trinidad and Tobago species (Mannophryne trinitatis and M. olmonae) from Venezuelan relatives at 8.3 MYA suggests a global period of high sea level. Second, six Pliocene dispersals from South America (3.2–5.4 MYA), immediately followed a proposed low sea-level period after 6.0 MYA [11]. Six vicariant events in the middle Pliocene (1.1–3.6 MYA) are concomitant with a second high sea-level period (1.5–3.0 MYA) that separated Central America from the Chocó [11]. Third, two dispersals in the late Pleistocene (0.5–2.2 MYA) are contemporaneous with the Great Faunal Interchange at 1.2 MYA [11]. Likewise, the endemic poison frog of Gorgona Island (Epipedobates boulengeri), located 50 km off the Pacific coast, was derived from a Chocoan ancestor 2.4 MYA during the same period. Our results strongly support the repeated dispersal of poison frogs into Central America across the PLB before its final Pliocene closure.

Similar results for the ancestral area reconstruction were obtained by dispersal-vicariance analysis (DIVA) [30]. However, DIVA provided unrealistic ancestral reconstructions for basal nodes (Figure S7), and a large number (i.e., ∼16 × 10^6^) of equally parsimonious reconstructions (see Material and Methods). Therefore, DIVA analyses were considered exploratory due to its algorithmic limitations [26,31].

### Lineage Diversification

We estimated diversification rates of the chronogram (i.e., dendrobatid family clade) using the adjusted γ statistic to account for incomplete taxon sampling [32,33]. The γ statistic compares the relative position of the nodes in a chronogram to that expected under the pure birth model, and different values of γ characterize whether the diversification rate has increased (γ > 0), decreased (γ < 0), or remained constant (γ = 0) over time [32]. However, we emphasize that the γ statistic is an indirect estimation of the rate of diversification [32], it should only be applied to monophyletic groups, and our inferences from the γ statistic should be considered relative measures of the diversification. Our result from the chronogram of the adjusted γ statistic is −0.662, and we failed to reject (*p* = 0.508^NS^) the null of the pure birth expectation of exponential growth, γ = 0. Simulations have suggested that significant positive values of γ have been associated with two alternatives, either increasing diversification or high extinction through time [34]. The general absence of Tertiary frog fossils from the lowland Neotropics is intriguing [35], but it does not provide evidence for or against increases in extinction/diversification, as suggested by our estimated γ statistic for the dendrobatid family clade. However, our results of the ancestral area reconstructions strongly suggest that the bulk of recent diversification in poison frogs might be due to rapid radiations in the Amazon Basin and the Central American-Chocó super-regions in the late Miocene. Therefore, our inference of recent dispersals to Amazonia and the recent geological origin of the modern Neotropical rainforest [18] might weigh in favor of a recent increase in diversification. Additional data from other Neotropical biota might be crucial to validate our inferences.

We further evaluated our claim of a significant increase in diversification in the Amazon Basin and the Central America-Chocó super-regions. We tested for significant changes in diversification rate at the genus-supraspecific level (GSPF chronogram and Figure 3; see Material and Methods) under incomplete taxon sampling using ML approach [36] with two extreme values of the extinction/speciation ratio (i.e., extinction rate fraction α = μ/λ, α = 0, and α = 0.99) (Table 2). The GSPF chronogram rejected the constant-rate model (all lineages with equal diversification rate) in favor of a variable rate model (at least one lineage has a significant higher or lower diversification rate) (Table 2). Additionally, the GSPF chronogram favored the variable-rate model with diversification rate change in one or more lineages (Figure 3; Tables 3, S13 and S14) over an alternative of retained elevated ancestral diversification rate (Table 2). However, we found possible spurious significant rate increases (i.e., nodes 2 and 9 of Figure 3 and Table 2) that might be dependent on more inclusive lower nodes (i.e., 1 and 5, respectively). This “trickle-down effect” artifact can be explained by a significant rate increase detected in daughter clade being carried over to the adjacent parent clade.

The diversification rate increase within *Ameerega* is 3.23-fold (α = 0) to 7.55-fold (α = 0.99) higher than the rest of the GSPF chronogram (Figure 3; Tables 3, S13 and S14). Interestingly, *Ameerega* corresponds the most recent (i.e., 8.7 MYA) widespread radiation of poison frogs in the Amazon Basin after dispersal from the Andes (Figures 2 and 3) in the late Miocene. Other significant increases in the diversification rate include two in Amazonia, two in the Chocoan region, and one in the Andes (Figure 3 and Table 3). Significant decreases in the rate of diversification correspond to the rare Guiana Shield endemic *Allobates* (0.0008-fold reduction) and the mostly Andean endemic Clade B (0.4851-fold reduction) (Figure 3; Tables 3, S13, and S14). Therefore, we found that Amazonian and Central American-Chocoan lineages significantly increased their diversification rate since the late Miocene, while the diversification rate in the Andean and Guianan-Venezuelan-Brazilian Shield lineages have been near constant with a tendency to slow down since the Oligocene. However, we acknowledge that these super-regions (i.e., the Andes and the Guiana-Venezuela-Brazilian Shield) might be undersampled (Table 1) and conclusions about their near-constant rate of diversification need further validation.

## Discussion

The unstable coexistence of lineages within a large community for extended periods of time has been hypothesized as a cause of Neotropical diversity [7]. However, our results suggest that such a model is incomplete; rather, the complex pattern of diversification is strongly intertwined with paleogeographic events. Our inferences about the past history of the poison frogs using ancestral area reconstructions and diversification analyses provide new insights on speciation and extinction patterns in the Neotropics. Three species richness patterns are potential explanations for the extant diversity differences among regions of the Neotropics: (1) high immigration into one region after suitable geoclimatic conditions are established; (2) gradual in situ diversification of old endemic clades, regardless of the geoclimatic conditions, promoting species accumulation; or (3) rapid in situ diversification of endemic clades after favorable geoclimatic conditions are established. We found that all three patterns might apply to different areas depending on historical context.

All extant Amazonian species descended from 14 lineages that dispersed into the Amazon Basin, mostly after the Miocene floodbasin system receded. The recurrent immigrations that originated mostly in the adjacent Andes (species-richness pattern 1), combined with an increased rate of diversification, explain the high α–diversity of Amazonia. Later, from the Miocene-Pliocene boundary to the present, a rapid in situ diversification (pattern 3) gave rise to the extant Amazonian endemic biota. Therefore, most species in Amazonia originated in the last 10 MY. Moreover, lineages immigrating into Amazonia at <8.0 MYA radiated rapidly, resulting in widespread species and young clades (e.g., *Ameerega*, *Allobates trilineatus*, A. femoralis, and Dendrobates ventrimaculatus groups).

The diversity in the Chocoan-Central American super-region derived from scattered immigrations (pattern 1) from Andes to the early Chocoan rainforest during the late Miocene. However, starting at the Miocene-Pliocene boundary, significant orogenic events gave rise to the Central American archipelago [11,37] followed by sea level fluctuations [38], which provided the conditions for repeated dispersal and vicariance events in pre-PLB islands. Evidence of rapid in situ diversification (pattern 3) is supported by the high genetic diversity observed among poison frogs and other lineages especially between Western and Eastern Panamá [12,39,40]. Interestingly, our results might explain the high β–diversity of other endemic clades within the Chocó-Central America super-region [8] as originating initially from long-distance dispersals between disconnected islands, with diversification later during isolation by high sea levels.

The Andes have undergone extended in situ diversification (pattern 2) since the late Eocene. However, our analyses also provided evidence of decline in the diversification rate since the middle Oligocene, which has important implications for history and conservation of the endemic Andean fauna. First, the Andes uplift at the Miocene–Pliocene boundary caused significant changes in the rate of diversification in the lowland transition zone. We found that several poison frog lineages distributed on one or both sides of the Andes had dispersed repeatedly before the Miocene uplift (i.e., five cross-Andean and five Northern to Central Andes migrations). Paleogeological evidence supports introgression of shallow seas across the northern Andes during the Miocene [14], suggesting a historical connection between the Amazon Basin and the Chocó. Second, the Pliocene Andean uplift (>2,000 m above sea level) [27] formed a significant barrier to dispersal, because no other cross-Andean dispersals were found. The uplift also was associated with dramatic ecological changes [27] and a decrease in diversification rates. These results suggest a role for niche conservatism [41,42], in that some lineages may have gone extinct because of failure to adapt. Alternatively, despite greater sampling effort in the Andes region than in other areas, we failed to find some previously common Andean species (e.g., Hyloxalus jacobuspetersi and the Ecuadorian H. lehmanni). Consequently, it is difficult to separate a natural decrease in diversification rates from the current trend of amphibian species extinctions at high altitudes due to anthropogenic habitat alteration [43], increased UV radiation [44], climate change [45], or pandemic infection [46]. In contrast, the montane transition zones of the Andes and adjacent lowlands (Chocó and Amazonia) have become centers of rapid cladogenesis (pattern 3), and species richness in these transition zones might be underestimated because many Neotropical lineages have been shown to contain several cryptic species [47]. Therefore, dispersals within or across the Andes diminished during the Pliocene, but diversification has intensified in the Andes-lowlands interface.

Although some of the oldest lineages of poison frogs originated in the Guiana Shield and the Venezuelan Highlands (>30 species), our results suggest extended in situ diversification (pattern 2) followed by a decline in the rate of diversification of endemic clades in both areas since the early Miocene. Along the same lines, the Guiana Shield has high poison frog endemism, which is mostly restricted to the summits of the sandstone tepuis [48], while recent Amazonian poison frog immigrants occupy lowlands adjacent to the tepuis. Our results suggest that the decline of endemic Guianan diversity might be associated with ecological changes in habitat due to the collapse of the ancient tepuis [4] and repeated dispersals from Amazonian lineages since the Pliocene. However, the diversity of poison frogs in the Guiana Shield is only beginning to be revealed [48]. In contrast, diversification in the Venezuelan region most likely reflects the oldest vicariant event in Dendrobatidae, at 40.9 MYA. The costal ranges of Mérida, Cordillera de la Costa, and Paria peninsula are species rich but their total area is less than 5% of that of the Amazon Basin. No lineage of this endemic fauna has dispersed out to other regions since the early radiation of the poison frog family in the late Eocene. However, Eocene floristic paleoecological reconstruction of the Venezuelan Highlands area showed that it was more diverse than at present [49], suggesting that the ancestral habitat of the first poison frogs might have been lowland. The depauperate dendrobatid fauna of the Venezuelan llanos and Brazilian Shield plateau is puzzling, but might be related to Holocene aridity [50].

The recurring dispersals to Amazonia suggests that a large part of dendrobatid diversity results from repeated immigration waves at <10.0 MYA, followed by a rapid in situ diversification after geoclimatic conditions suitable for a rainforest ecosystem were present. The biota of Amazonia was not isolated during the process of diversification, but finely intertwined with the development and export of biodiversity across the entire Neotropical realm. Poison frog diversity in the Chocoan-Central American super-region was significantly associated with formation of the PLB in the Pliocene. Repeated dispersals between disconnected islands followed vicariance by cyclic high sea-level periods, promoted rapid in situ diversification and endemism of poison frog lineages. The extant Andean, Guianan, and Venezuelan Highlands fauna most likely originated after prolongated in situ diversification since the origin of the poison frog clade, but the pace of species formation within these areas has slowed down. Phylogenetic analyses on tropical biota such as birds [51] and the species-rich genus *Inga* [52] as well as models of diversification [3], have argued that the Amazon might accumulate older lineages; however, the origin of those lineages is not clear. Our results are the first to provide evidence, to our knowledge, of the major involvement of the Andes as a source of diversity of both the Amazon and the Chocó–Central America region. Because 23.5% of endemic Amazonian amphibian species are dendrobatids (i.e., ∼70 of 298) [53], our results may generalize to other Neotropical terrestrial biota with similar distribution. Moreover, these results provide a crucial broad spatiotemporal framework that, coupled with realistic phylogeny-based explanations of the current richness in Neotropics, explains why species occur where they do and how they came to get there. Thus, the major patterns of dispersals and radiations in the Neotropics were already set by the Miocene–Pliocene boundary, but the ongoing process of Neotropical radiation is occurring now, in the Chocó–Central America region and especially in the Amazonian rainforest.

## Materials and Methods

### Data collection and species sampled.

Because there are no fossil poison frogs, a large phylogeny of amphibians was constructed to calibrate the age of the root of the dendrobatid tree. We used a total of 89 terminals including 80 species of anurans (30 families), three species of salamanders (three families), three species of caecilians (three families), and three outgroups (lungfish, human, and chicken) (Figures S1 and S2; Table S2). The amphibian classification partially follows that of Frost et al. [54]. Conflicts with Frost et al. [54] are indicated as paraphyletic families (e.g., Dicroglossidae 1 and 2).

Molecular data include the mitochondrial rRNA genes (12S and 16S sequences; ∼2,400 bp) and the nuclear protein-coding gene RAG-1 (approximately 495 bp). Sequences were retrieved from GenBank (74 terminals) or sequenced (15 terminals) from total genomic DNA (Table S2). The primers and protocols for amplification, purification, and sequencing of PCR products are provided in previous studies [22,55,56]. PCR products were sequenced in both directions and compared to GenBank sequences using BLAST (http://www.ncbi.nlm.nih.gov/BLAST/). By this procedure, we were also able to validate sequences in GenBank and exclude contaminated or mislabeled submissions. GenBank accession numbers are given in Table S2.

A total of 406 individuals for described (137) and undescribed (34–89) species of poison frogs and 12 outgroups (from Hyloidea) were used to estimate the phylogeny (Figure S3A–S3D; Table S3). The estimate of undescribed species (34–89 spp.) corresponds to the estimated minimum and maximum number of new species. Therefore, the described diversity of poison frogs (264 species) plus the diversity discovered by our analysis yields a maximum of 353 (264 known + 89 maximum number of undescribed species), which is a better estimate of the true extant diversity. Of the 127 described poison frog species not sampled (Table S11), we were able to identify their closest relative or species group in 92.1% of the cases (117 species). Thus, we are confidant that we have not missed any crucial lineage and that our conclusions will hold as more data are incorporated. Furthermore, the conservation status of the unsampled species is based on the Global Amphibian Assessment [57] is as follows (Table S11): 51.3% are data deficient, 16.6% have been described recently (no category), 28.9% are in one of four “threatened” categories, and 3.2% are of least concern.

The classification here partially follows that of Grant et al. [23]. Species placements that conflict with this taxonomy are indicated as paraphyletic genera (e.g., *Colostethus* 1 and 2). Proposed taxonomic changes and corrections are explained in “Corrections to Taxonomy” (Text S1). Our taxon sample included species from throughout the distribution of dendrobatid frogs, with regions of higher diversity sampled more extensively (Table S3) and the 117 species that were not sampled were assigned to a taxonomic group (Tables S11 and S13) and included in the Materials and Methods section “Speciation and extinction patterns under incomplete taxon sampling.” Molecular data were generated using the same protocols indicated in the first paragraph of Materials and Methods. We included only the mitochondrial rRNA genes (12S and 16S sequences; ∼2,400 bp), from which 374 individuals (121 species) have complete sequences. GenBank sequence accession numbers are given in Table S3; because some outgroup sequences will appear in other papers currently under review, their numbers are not listed. Although sequences from other genes are available in GenBank, these were not used because data from the data are highly incomplete; complete data for only 80 species were available. Moreover, simulation studies have indicated that large phylogenetic analyses including many terminals with incomplete sequences might bias branch length estimation, and cause topological inconsistencies due to unrealistic estimates of the rate of evolution [58].

### Sequence alignment, data partitioning, and model testing.

Contigs were assembled using Sequencher 4.7 [59]. Sequences were initially aligned using ClustalX 1.81 [60] and manually adjusted to minimize informative sites using MacClade 4.08 [61]. The final matrices included 2,688 characters (i.e., 2,192 from mitochondrial genes, 495 from RAG-1) for the Amphibian Tree Matrix (ATM) and 2,380 characters (mitochondrial genes) for the Poison Frog Tree Matrix (PFTM). A total of 228 (ATM) and 113 (PGTM) ambiguously aligned characters were excluded. For the ATM, we divided the data into mitochondrial gene and RAG-1 partitions. For the PFTM, we used 12S rRNA, tRNA-Valine, and 16S rRNA partitions. The best model of nucleotide substitution for each partition was determined using ModelTest 3.7 [62,63]. For the ATM, GTR + Γ + I and TrN + Γ + I were selected for the mitochondrial genes and RAG-1 segments, respectively. For the PFTM, GTR + Γ + I was selected for the 12S and 16S rRNA segments and the GTR + Γ for the tRNA-valine segment.

### Phylogenetic analysis.

ATM and PFTM were analyzed with ML methods under a genetic algorithm in GARLI 0.951 [64] and with Bayesian sampling of tree space with MrBayes 3.1.2 [65,66]. For the ATM analyses, the amphibian species were constrained to be monophyletic. For the GARLI analyses, a total of 40 independent runs were used to infer the best tree and 200 nonparametric bootstrap searches were used to estimate support for the nodes. For the MrBayes analyses, tree topology estimation, branch lengths, and Bayesian posterior probabilities (PP) were determined from five independent runs of four incrementally heated chains. Runs were performed for 35–45 × PFTM 10^6^ generations under partitioned models using default settings as priors; the sampling frequency was 1 in 1,000 generations. The convergence of the runs and the optimal burn-in was determined to be 1.238 × 10^6^ (ATM) and 4.282 × 10^6^ (PFTM) generations using MrConverge [58]. This program estimates the point where the likelihood score becomes stationary and the overall precision of the bipartition posterior probability is maximized.

### Determination of divergence times.

For molecular dating analyses the strict molecular clock model was rejected from both ATM (χ^2^ = 4,762.6, df = 87, *p <* 0.001) and PFTM (χ^2^ = 3822.6, df = 404, *p <* 0.001) datasets using a likelihood ratio test (LRT) that compared the best unconstrained GARLI trees to those estimated under a strict molecular clock. Therefore, a relaxed molecular clock Bayesian method in MULTIDIVTIME [21] was used to estimate chronograms for the Amphibian Tree and the Poison Frog Tree.

For chronogram estimation, all taxa in the ATM dataset (Amphibian Chronogram) and a pruned PFTM dataset (Poison Frog Chronogram) were used. The pruned PFTM dataset excluded multiple individuals of the same species to improve computational efficiency. For each analysis (ATM and pruned PFTM), the aligned matrix and the rooted ML topologies without branch lengths were input into MULTIDIVTIME [21]. Branch length estimates under the F84 + Γ model of molecular evolution and variance/covariance matrices were calculated using the BASEML and ESTBRANCHES components of PAML 3.15 [67] and MULTIDIVTIME [21], respectively. Calibration points (Figures S4 and S5; Tables S4–S6), relaxed-clock model priors, and variance/covariance matrices were then input into MULTIDIVTIME [21].

For the Amphibian Chronogram, three different sets of time constraints were used to assess the robustness of the dating estimates; these were based on paleogeography, vertebrate fossils, and amphibian fossil records (Table S4). The ingroup tip-to-root distances needed for the estimation of the MULTIDIVTIME rtrate and rtratesd priors [21] were calculated using TreeStat v1.1 [68]. The relaxed-clock model priors were 344 MYA for the expected age between tip and root (rttm) and 20 MYA for its standard deviation (rttmsd). The rttm prior corresponds to the divergence of Amniota and Amphibia and is based on fossil and molecular analyses [69–72]. The expected molecular evolution rate at the ingroup root node (rtrate) prior and its standard deviation (rtratesd) were estimated at 0.00345 substitutions/site/MY by dividing the median of ingroup tip-to-root distances by the rttm prior as suggested by the MULTIDIVTIME documentation [21]. The priors for the expected value of the Brownian motion constant υ (nu) (brownmean) and its standard deviation (brownsd) were estimated to be 0.058 by setting rttm * brownmean equal to 2.0 (on-line suggestion of Frank Rutschmann [73]). The bigtime parameter was set to twice the estimated time divergence of Amniota and Amphibia (i.e., 700 MYA). Markov chain (newk, othk, thek) and beta (minab) priors were set to default values. Each MCMC chain was run for 1 × 10^6^ generations with sampling frequency of 1 per 100 generations and burn-in of the first 100,000 generations. All analyses were run twice to ensure convergence of the time estimates. The divergence time estimated for each node of the amphibian chronogram was described by its mean age and 95% confidence interval (CI) (Table S7).

The Poison Frog Chronogram was estimated from the pruned PFTM dataset that included 224 individuals from 157 poison frog species (131 described and 26 undescribed) and 12 outgroups. The fossil record of Tertiary Neotropical frogs is minimal and no fossils of poison frogs have been found [35]. For this reason, we used a three-part strategy to date the Poison Frog Chronogram. First, the expected ages and 95% CIs of the split of the Dendrobatidae from other Hyloidea and the age of the most recent common ancestor of this clade were estimated from the Amphibian Chronogram. Second, a list of geological time constraints (Table S5) was developed based on paleogeological evidence (Table S6). Third, ancestral area reconstruction was inferred and explained in the Materials and Methods section “Ancestral area reconstruction”. To test the overall accuracy of these approaches, three sets of progressively less inclusive time constraints were used (Table S5). The relaxed-clock model priors were 71.4 MYA for the expected age between tip and root (rttm) and 18.6 MYA for its standard deviation (rttmsd). This value, estimated from the Amphibian Chronogram, corresponds to the mean age of the split between Dendrobatidae and its sister clade. The expected value (rtrate) and standard deviation (rtratesd) priors were set to 0.0056 substitutions/site/MY. The priors for the expected value (brownmean) and its standard deviation (brownsd) of the Brownian motion constant υ (nu) were set to 0.2. These priors (rtrate, rtratesd, brownmean, and brownsd) were obtained similarly as in the Amphibian Chronogram (see above). The bigtime parameter was set to three times the estimated age of the Dendrobatidae node from the Amphibian Chronogram (40 × 3 = 120 MYA). Markov chain priors (newk, othk, thek), beta prior (minab), and MCMC chain parameters were the same as for the Amphibian Chronogram. All analyses were run twice to ensure convergence of the time estimates. The divergence time estimated for each node of the Poison Frog Chronogram was described by its mean age and 95% CI (Table S8) and major taxonomic events (Table S9).

We assessed the robustness of the calibrations (Table S5) with three approaches. First, we recalculated the chronogram by using penalized likelihood approach (PL) [74] implemented in r8s [75]. Because the penalized likelihood method requires at least one fixed node age, the nodes were fixed for root of the poison frog tree at 42.9 MYA or for the major split between clades B + C + D and A at 36.3 MYA. These values correspond to the mean age for each node obtained by averaging the ages from the three estimates of the Amphibian Chronogram (Table S7). We calculated the Poison Frog Chronogram with all constraints and then by removing one calibration constraint at a time (Tables S9 and S10). We use five different smoothing parameters (1, 10, 100, 500, and 1,000), an additive scale for rate penalty, and 20 random starts to estimate the chronogram. The average of all runs without the respective constraint was used as the estimate of node age (Table S9B). Second, we recalculated the chronogram using the same parameters of the relaxed-clock Bayesian method but removing one calibration constraint at a time (Table S9A). Finally, we recalculated the Poison Frog Chronogram to determine the effect of the combination of bigtime and rttmsd priors, which have been shown to have the substantial impact [76], by changing the estimates of nodes to make them older or younger. We used the same rttmsd prior (18.6 MYA) and two bigtime prior values of 60 and 80 (Table S9A). Based on all tests, the estimates of 36 exemplar nodes that correspond to major phylogenetic events in the family (Table S9A and S9B) and the differences in MYA from the Poison Frog Chronogram node mean are provided (Table S10). We found that all time estimates from each test were within two standard deviations of the mean of poison frog chronogram (Table S10). The time estimates obtained by using the penalized likelihood method mostly were within one standard deviation from those estimated in the Poison Frog Chronogram. In the case of the tests done using the relaxed molecular clock Bayesian method, the removal of constraint A (2.4–15.0 MYA, which was assigned to nodes that correspond to dispersals between South America and Central America) was found to provide older time estimates for all nodes, especially those close to the root. The effect of changing the rttmsd/bigtime priors combination was negligible.

### Ancestral area reconstruction.

The reconstruction of ancestral areas of the poison frog clade was determined by three methods: a maximum-likelihood inference of geographic range evolution [26,31,77], DIVA [30], and Bayesian analysis of ancestral areas [78–80]. Ten areas (designated by letters) were delimited on the basis of geological barriers, areas of endemism [81–83], and distribution maps [57] (Figure 1; Table S3). The Andes were divided into four adjacent regions: Central Occidental Andes (H), Central Oriental Andes (G), North Oriental Andes (E), and North Occidental Andes (F). Northern and Central Andes are divided perpendicular to the southern limit of Carnegie Ridge (parallel 2 °S) in southern Ecuador [84,85]. The Oriental and Occidental Andes are divided along the Interandean valleys that separate both parallel mountain chains. The Guiana Shield (B) and Brazilian Shield (K) regions are located in the eastern shoulder and center of South America, respectively. Both regions are ancient Precambrian plateaus with lowland tropical rainforest, dry forest, and cloud forests from 200–2,800 m. The Venezuelan Highlands (D) region includes the current Caribbean costal cordilleras of Venezuela (Mérida, Cordillera de la Costa, and Paria peninsula) and Trinidad and Tobago Islands. Paleontological and stratigraphical evidence suggests that Venezuelan Highlands region had strong similarities to the western Amazonian Tertiary fossil fauna [86–89]. However, the Venezuelan Highlands biogeographic distinctiveness is evidenced by the Miocene uplift [90], episodic Miocene floodings, and the formation of the Llanos [91], the separation from the Guiana Shield region by the current Orinoco river drainage and from the northern Andes by the Táchira depression. The Amazon Basin (C) region includes the river drainage and its extensive lowland tropical rainforest <300 m. The Central America (J) region corresponds to the lowlands and highlands of western side of the PLB to southern Nicaragua (northernmost distribution of poison frogs). The Chocó (I) region includes the eastern side of the PLB and the costal lowland tropical forest below 500 m of the Pacific Coast of Ecuador and Colombia on the western side of Andes. The Magdalena river drainage and the Gorgona Island were also included in the Chocó region based on the paleontological and biotic resemblance to the Chocó [92–94].

Ancestral area reconstructions, time of diversification, and rate of diversification estimates require a fully bifurcate tree [95–98]. We used the best GARLI tree to reconstruct the ancestral areas. Each species in the phylogeny was assigned to one or more regions based on distribution (Table S3). For the first method, we estimated a DEC model using Lagrange package [26,77]. The DEC model is a continuous-time stochastic model for geographic range evolution in discrete areas, with ML parameters estimated for rates of dispersal between areas (range expansion) and local extinction within areas (range contraction). The DEC model considers geographic scenarios of lineage divergence (including scenarios involving within-area speciation), allowing a widespread ancestral range to persist through a cladogenesis event as ancestral states at internal nodes on a phylogeny with observed species ranges at the tips. In all cases, ancestral ranges were assumed to include no more than two areas, the maximum observed for extant species. Moreover, spatial and temporal constraints (e.g., area distances, dates of geological origin) may be imposed in the DEC model estimation, providing a more accurate estimation of the ancestral areas and hypothesis testing of specific geographic scenarios.

We tested three biogeographic scenarios based on the hypothesized origin of the group (Figure S6). First, the SM0 null model has all areas as equiprobable ancestral ranges and assigns them to be adjacent pairs (i.e., separated by one step in the matrix), plus the individual areas themselves. Second, the SM1 (center-of-origin model) favors an Amazon Basin origin of the poison frog clade and assigns all non-Amazonian areas to be adjacent to the Amazon Basin (i.e., separated by one step in the matrix) and nonadjacent to each other (i.e., separated by two steps). However, the one exception was to make the Chocó and Central America adjacent, because it is not possible to reach Central America without passing through the Chocó. Third, the SM2 model (stepping-stone model) assigns the rates of dispersal between areas to be inversely scaled by their relative distance and connectivity (e.g., the distance between Guiana Shield and Central America is four steps, so the rate of dispersal was constrained to be 0.25). Each analysis estimated the global rate of dispersal and local extinction on the phylogeny with species ranges at the tips, considering all possible range inheritance scenarios (ancestral states) at internal nodes without conditioning on any particular values. Then, the global rates were used to calculate and rank the likelihoods of all ancestral states at every internal node on the phylogeny. The ranking is done by calculating the likelihood at the root of the tree, given the global rates, with one node fixed at one ancestral range scenario, and all other nodes free to vary. The ML scores for all nodes are compared to the overall ML score of each geographic scenario under a “global” method of ML ancestral state reconstruction [99]. The likelihood of each model was optimized against the observed ranges of species and their phylogenetic relationships, with differences greater than 2 log-likelihood units considered significant; the reconstruction with the worst score was rejected [99]. Tests of the three hypotheses (SM0, SM1, and SM2) were repeated in both the complete ML phylogeny and chronogram (reduced number of taxa). All tests were repeated in both large ML phylogeny and chronogram including and excluding Allobates alagoanus whose phylogenetic position at the base of clade A is not well supported (Figure S3A). In all cases similar results were found, so the results excluding A. alagoanus are presented.

For the second method, we performed ancestral area reconstruction by DIVA using DIVA 1.1 [30]. DIVA assumes that speciation occurs as a consequence of vicariance and reconstructs these events at no cost. Therefore, the ancestral geographic distributions are reconstructed by minimizing the number of dispersal or extinction events necessary to explain the actual distribution pattern. Because we assigned all nine regions to favor dispersals equally, no single solution for the large tree was found (∼16 × 10^6^ possible reconstructions). Hence, this analysis is considered exploratory due to its limitations. DIVA, which is parsimony-based, only optimizes historical events on cladograms without regard to relative or absolute time (i.e., branch lengths), and is less flexible about geographic lineage divergence scenarios (e.g., widespread ancestors are assumed to undergo vicariance); specific biogeographic hypotheses cannot be tested. In consequence, the strict consensus of the dispersal/vicariance events on >75% of all possible reconstructions was used as the best solution and mapped on the ML poison frog phylogeny (Figure S7).

For the third method, estimates of the values of states at ancestral nodes were derived by point estimates (log-likelihoods) using an ML approach in BAYESMULTISTATE, a component of BAYESTRAITS [79,100]. The reduced chronogram (236 terminals) described in the Materials and Methods section “Determination of divergence times” was used for the analysis. We coded all terminal distributions under two alternatives, Amazonian Basin (C) origin, and non-Amazonian Basin origin (all other areas). We calculated the proportion of likelihood of both alternatives at each node with 10,000 samples under default parameters. The results were mapped onto the chronogram (Figure 2). We also tested whether an Amazonian origin was present at each node by constraining the node to this state using the option “fossilizing” in BAYESMULTISTATE [100]. The likelihoods of the constrained and unconstrained reconstructions were compared, and a difference of ≥2 log-likelihood units was considered significant; the model with the worse score was rejected [99].

### Speciation and extinction patterns under incomplete taxon sampling.

We calculated the tree imbalance of the ML poison frog phylogeny, the reduced-taxon chronogram, and the tree of each super-regions: the Amazon Basin (region C), the Andes (regions E–H), Chocó-Central America (regions I and J), and Guiana Shield-Venezuela-Brazilian Shield (regions B, D, and K). We used conservative imbalance metrics [25,101]: *I_C_* [102], *I_S_* [103], and *s* [104,105]. All standardized indices and probability of rejecting the null model of each branch having the equal probability of splitting (Equal Markov Rate or ERM) were calculated using functions colless.test, sackin.test, and likelihood.test, implemented in the R package apTreeshape [106].

An indirect estimate of diversification rates assuming incomplete taxon sampling was explored using the γ statistic [32] and adjusted for actual phylogenies by excluding the distance between the most recent node to the present (i.e., *g*_n_ node of a phylogeny of size *n*), which does not come from the same distribution [33]. The adjusted γ statistic value was obtained from the Poison Frog Chronogram (family level).

The adjusted γ statistic [32,33] was calculated using following functions of the R package LASER [107]: (1) the g_n_ node was excluded from the chronogram (i.e., entire family and major region subtrees) by using truncateTree function; (2) the γ statistic of the truncated tree was calculated using gamStat function; (3) a null-distribution of γ under a pure birth model was obtained by 10,000 Monte Carlo replicates using mccrTest.Rd. This function generates full size trees at the species level and prunes randomly terminals to the actual size of the empirical tree (i.e., simulates incomplete taxon sampling); (4) the empirical γ statistic was adjusted by subtracting the mean value of the simulated null-distribution of γ, which is expected to be 0 [33]; and (5) the *p*-values of the adjusted γ statistics were computed from a normal distribution; values outside the ±1.96 standard deviation boundaries are significantly different (alpha = 0.05) from the null pure birth expectation.

We tested for significant changes in diversification rate within the poison frog clade using a ML methodology under the assumption of incomplete taxon sampling [36]. First, we produced a GSPF level chronogram by pruning all but a single lineage per taxonomic group (Figures 3 and S8; Table S13) from the Poison Frog Chronogram, yielding a tree of 78 terminals. The total species richness per taxonomic group was assigned to each terminal based on previous taxonomic and phylogenetic studies [22,23,108–112]. Second, we estimated a constant diversification rate *r* (i.e., the difference between speciation λ and extinction μ rates) across the phylogeny using a ML estimator that incorporates both known taxonomic diversity and phylogenetic data [36]. We calculated the constant-rate model fit statistics (log likelihood and AIC score) and *r* using the fitNDR_1rate.Rd function of LASER [107]. Third, we tested for shifts in diversification rate within the poison frog phylogeny by comparing likelihood of the GSPF chronogram under constant and rate-flexible diversification models [36]. Two alternative hypotheses for rate-flexible model may explain the shifts in rate of diversification: (1) an increase within a particular clade (*r*_CL_) from the ancestral diversification rate *r* (flexible-rate model) or (2) a clade-specific decrease (*r*_CL_) from the ancestral diversification rate *r* (rate-decrease model) [36]. We calculated both rate-flexible alternative model fit statistics, *r* and *r*_CL_ values using the fitNDR_2rate.Rd function of LASER [107]. The best fitting model was determined using a likelihood ratio test (LRT) between the constant-rate and the flexible-rate models (nested), and by ΔAIC scores between the flexible-rate and rate-decrease models (not nested). All analyses were performed under two extremes of the relative extinction rate (α = μ/λ, α = 0 and α = 0.99) as a fixed parameter to determine the robustness of the results to variation in the extinction fraction [113].

### Sequence accession numbers.

GenBank (http://www.ncbi.nlm.nih.gov/Genbank) sequence accession numbers mentioned in this paper are EU342502–EU342745 (see Tables S2 and S3).

## Supporting Information

Figure S1 Molecular Phylogeny of Amphibians Including 80 Species of Anurans (30 Families), Three Species of Salamanders (Three Families), Three Species of Caecilians (Three Families), and Three Outgroups (Lungfish, Human, and Chicken) The phylogram was produced under a genetic algorithm in GARLI 0.951 [64]. We also inferred the tree topology and branch lengths using a Bayesian sampling of tree space with MrBayes 3.1.2 [65,66]. Support values from the nodes were constructed with 200 nonparametric bootstrap replicates (above branch) and Bayesian posterior probabilities (below branch). An asterisk indicates a support value of 100.
(5.20 MB JPG)

Figure S2 Molecular Chronogram of the Amphibians (80 Anuran, Three Salamander, and Three Caecilian Species) Used to Date the Origin and Divergence of Poison Frogs (Dendrobatidae) (a) Relaxed clock chronogram estimated from the ML tree using time constraints (date from seven fossils and one paleogeographic date constraints as in Table S4); support values for the nodes were constructed with 200 bootstrap replicates (ML) using GARLI; Bayesian posterior probabilities (PP) were estimated using MrBayes; gray bars are 95% CI of the estimated node age. Six major geological events are indicated by red squares: (1) rifting of Pangaea; (2) Gondwana break-up; (3) separation of South America from Africa; (4) Cretaceous–Tertiary mass extinction; (5) initial orogeny of the Andes; (6) formation of the modern Amazon Basin; (7) Stem Dendrobatidae; and (8) Dendrobatidae.
(4.53 MB JPG)

Figure S3 Molecular Phylogeny of the Poison Frogs Inferred from 394 Individuals of 137 Described and 34–89 Undescribed Species (A–D) The phylogram is the ML methods under a genetic algorithm in GARLI 0.951 [64]. We also inferred the tree topology and branch lengths using a Bayesian sampling of tree space with MrBayes 3.1.2 [65,66]. Support values from the nodes were constructed with 200 nonparametric bootstrap replicates (above branch) and Bayesian posterior probabilities (below branch). (*) indicates that the support value was 100. The coding of the individual terminals includes the generic, species, locality, country code, biogeographic area, and museum, field series, or GenBank accession numbers (see Table S3).
(2.55 MB PDF)

Figure S4 Node Numbers and Constraints of the Amphibian Chronogram Used in the Molecular Dating Analyses See Table S4.
(4.38 MB JPG)

Figure S5 Node Numbers and Constraints of the Poison Frog Phylogeny Used in the Molecular Dating Analyses (A–D) The nodes preceded by “N” correspond to the ML phylogeny and those that were preceded by “#” correspond to the chronogram. See Table S5.
(2.36 MB PDF)

Figure S6 Geographic Scenarios for Diversification of Poison Frogs (1) SM0, the null model, assumes no spatial structure, with equal rates of dispersal among all areas and no constraints on ancestral range composition; (2) alternative center of origin model SM1 models the dispersal rate into and out of the Amazonia as twice the rate between all other areas, with widespread ancestral ranges constrained to include the Amazon Basin; and (3) a stepping-stone model SM2 accounts for area adjacency, scaling rates of dispersal between areas inversely by relative distance, and constraining widespread ancestors to spatially adjacent areas. Each hypothesis test was repeated in the large ML phylogeny and in the chronogram. The area codes correspond to Guiana Shield (B), Amazon Basin (C), Venezuela Highlands and Trinidad and Tobago Islands (D), Northern Andean Cordillera Oriental (E), Northern Andean Cordillera Occidental (F), Central Andean Cordillera Oriental (G), Central Andean Cordillera Occidental (H), Chocó, Magdalena Valley, and Gorgona Island (I), Central America (J), and Brazilian Shield (K).
(814 KB JPG)

Figure S7 The Ancestral Areas Inferred from DIVA and DEC Model using Lagrange, Mapped Over the Complete ML Tree Reconstruction The “?” corresponds to *Allobates alagoanus,* whose phylogenetic relationships are uncertain [26,30,77].
(7.57 MB JPG)

Figure S8 The GSPF Level Chronogram with the Species Groups Used for the Diversification Analyses Node numbers correspond to those provided in Figure S5A–S5D that match the ML phylogeny.
(5.04 MB JPG)

Table S1 Tree Imbalance Indices (Colless *I_C_*, Sackin *I_S_*, and Likelihood Shape *s*) Estimated for the Poison Frog Chronogram, the Trees for Each Super-Region and the Corresponding Probability of Rejecting the Null Equal Markov Rate An asterisk indicates significance at α = 0.05.
(49 KB PDF)

Table S2 Taxa Used to Infer the Age of the Root of the Poison Frog Tree Including GenBank Accession Numbers of Genes Used (82 KB PDF)

Table S3 Taxa Used to Infer the Phylogeny and the Chronogram of the Poison Frogs “Use” identifies which samples were used for the phylogeny (P), chronogram (C), and Langrage (L) analyses. Other columns provide GenBank accession numbers, locality of collection, and biogeographic region of the Neotropics.
(305 KB PDF)

Table S4 Time Constraints Used to Infer the Age of the Root of the Poison Frog Tree (94 KB PDF)

Table S5 Poison Frog Time Constraints Node numbers are indicated in the Figure S5A–S5D.
(107 KB PDF)

Table S6 Published Evidence Supporting the Paleogeographic Constraints Used in Inferring the Poison Frog Chronogram (111 KB PDF)

Table S7 Amphibian Tree estimated Divergence Times for MULTIDIVTIME Nodes from Figure S4A–S4D.
(74 KB PDF)

Table S8 Poison Frog Tree Estimated Divergence Times, Events (Vicariance and Dispersals), Lagrange Ancestral Area Reconstructions, and BayesTraits Probability of Amazon Basin Area at the Chronogram Node Node numbers form Figure S5A–S5D.
(124 KB PDF)

Table S9 Poison Frog Tree Estimated Divergence Times of Taxonomic Events and Results of the Chronogram Calibration Robustness and Constraint Reliability Analysis (A) corresponds to the node age estimations using MULTIDIVTIME constraint jackknifing and “bigtime” prior variation.
(B) corresponds to the node age estimations using penalized likelihood approach (r8s) [71] constraint jackknifing. Node numbers form Figure S5A–S5D.
(142 KB PDF)

Table S10 Node Time Differences between the Chronogram Age Mean and Those of the Jackknifed Constraint Estimated Chronograms for Both r8s and MULTIDIVTIME Bold indicate difference (V), that is 2 SD > V > 1 SD of the TimeSet 1 chronogram. Node numbers form Figure S5A–S5D.
(72 KB PDF)

Table S11 Species of Poison Frogs Not Included in the Analyses with Information about the Author of the Species Description, Distribution, Range, Area of Distribution, Phylogenetic Group, and Conservation Status from Global Amphibian Assessment (GAA) (196 KB PDF)

Table S12 Poison Frog Tree Dispersal Events Indicated in Figure 2 with Their Estimated Divergence Times and Node Number from the ML Phylogeny Node numbers form Figure S5A–S5D.
(65 KB PDF)

Table S13 Poison Frog Species Group and Taxonomic Diversity Assigned to the Diversification Rate Analyses and Figure 3
The species sampled for the analyses are indicated by “*”.
(62 KB PDF)

Table S14 Diversification Rate Estimates under the Flexible-Rate Model with the Lowest Extinction Fraction (*a* = 0 and *a* = 0.99) Node numbers correspond to Figure S8.
(94 KB PDF)

Text S1 Corrections to the Poison Frog Taxonomy and Supporting Literature for Tables S4–S6
(52 KB DOC)

## Abbreviations

ATM: Amphibian Tree Matrix
CI: confidence interval
DEC: dispersal-extinction-cladogenesis modeling
DIVA: dispersal-vicariance analysis
GSPF: genus-supraspecific poison frog chronogram
ML: maximum likelihood: MY, million years
MYA: million years ago
PLB: Panamanian Land Bridge
PFTM: Poison Frog Tree Matrix
SM0: no spatial structure biogeographical null model
SM1: Amazon Basin as a center-of-origin biogeographical alternative model
SM2: stepping-stone biogeographical alternative model

## References

[pbio-1000056-b001] Wilson EO, Wilson EO (1988). Biodiversity.

[pbio-1000056-b002] Myers N, Mittermeier RA, Mittermeier CG, da Fonseca AB, Kent J (2000). Biodiversity hotspots for conservation priorities. Nature.

[pbio-1000056-b003] Moritz C, Patton JL, Schneider CJ, Smith TB (2000). Diversification of rainforest faunas: an integrated molecular approach. Ann Rev Ecol Syst.

[pbio-1000056-b004] Burnham RJ, Graham A (1999). The history of neotropical vegetation: new developments and status. Ann Mo Bot Gard.

[pbio-1000056-b005] Pitman NCA, Terborgh JW, Silman MR, Nunez P, Neill DA (2001). Dominance and distribution of tree species in upper Amazonian terra firme forests. Ecology.

[pbio-1000056-b006] Bush MB (1994). Amazonian speciation - a necessarily complex model. J Biogeogr.

[pbio-1000056-b007] Hubbell SP, Levin SA, Horn HS (2001). The unified neutral theory of biodiversity and biogeography.

[pbio-1000056-b008] Condit R, Pitman N, Leigh EG, Chave J, Terborgh J (2002). Beta-diversity in tropical forest trees. Science.

[pbio-1000056-b009] Wiens JJ, Donoghue MJ (2004). Historical biogeography, ecology and species richness. Trends Ecol Evol.

[pbio-1000056-b010] Patton JL, da Silva MNF, Howard DJ, Berlocher SH (1998). Rivers, refuges and ridges: the geography of speciation of Amazonian mammals.

[pbio-1000056-b011] Coates AG, Obando JA, Jackson JBC, Budd AF, Coates AG (1996). The geological evolution of the Central American Isthmus.

[pbio-1000056-b012] Bermingham E, Martin AP (1998). Comparative mtDNA phylogeography of neotropical freshwater fishes: testing shared history to infer the evolutionary landscape of lower Central America. Mol Ecol.

[pbio-1000056-b013] Knowlton N, Weigt LA (1998). New dates and new rates for divergence across the Isthmus of Panama. Proc R Soc Lond B.

[pbio-1000056-b014] Hoorn C (1994). An environmental reconstruction of the paleo-Amazon River system (Middle-Late Miocene, NW Amazonia). Palaeogeog Palaeoclim Palaeoecol.

[pbio-1000056-b015] Latrubesse EM, Bocquentin J, Santos CR, Ramonell CG (1997). Paleoenvironmental model for the late Cenozoic southwestern Amazonia: paleontology and geology. Acta Amazonica 27.

[pbio-1000056-b016] Lovejoy NR, Albert JS, Crampton WGR (2006). Miocene marine incursions and marine/freshwater transitions: Evidence from Neotropical fishes. J South Am Earth Sci.

[pbio-1000056-b017] Kaandorp RJG, Wesselingh FP, Vonhof HB (2006). Ecological implications from geochemical records of Miocene Western Amazonian bivalves. J S Am Earth Sci.

[pbio-1000056-b018] Hoorn C (2006). The birth of the mighty Amazon. Sci Am.

[pbio-1000056-b019] Sepkoski JJ (1997). Biodiversity: past, present, and future. J Paleontol.

[pbio-1000056-b020] Alroy J (2002). How many named species are valid. Proc Natl Acad Sci U S A.

[pbio-1000056-b021] Thorne JL, Kishino H (2002). Divergence time and evolutionary rate estimation with multilocus data. Syst Biol.

[pbio-1000056-b022] Santos J, Coloma LA, Cannatella DC (2003). Multiple, recurring origins of aposematism and diet specialization in poison frogs. Proc Nat Acad Sci USA.

[pbio-1000056-b023] Grant T, Frost DR, Caldwell JP, Gagliardo R, Haddad CFB (2006). Phylogenetic systematics of dart-poison frogs and their relatives (Anura: Athesphatanura: Dendrobatidae). Bull Amer Mus Nat Hist.

[pbio-1000056-b024] Heath TA, Zwickl DJ, Kim J, Hillis DM (2008). Taxon sampling affects inferences of macroevolutionary processes from phylogenetic trees. Syst Biol.

[pbio-1000056-b025] Blum MGB, Francois O (2006). Which random processes describe the tree of life? A large-scale study of phylogenetic tree imbalance. Syst Biol.

[pbio-1000056-b026] Ree RH, Smith SA (2008). Maximum-likelihood inference of geographic range evolution by dispersal, local extinction, and cladogenesis. Syst Biol.

[pbio-1000056-b027] Gregory-Wodzicki KM (2000). Uplift history of the Central and Northern Andes: a review. Geol Soc Am Bull.

[pbio-1000056-b028] Roberts JL, Brown JL, von May R, Arizabal W, Schulte R (2006). Genetic divergence and speciation in lowland and montane peruvian poison frogs. Mol Phylogenet Evol.

[pbio-1000056-b029] Webb SD, Stehli FG, Webb SD (1985). Late Cenozoic mammal dispersal between the Americas. The great American biotic interchange.

[pbio-1000056-b030] Ronquist F (1997). Dispersal-vicariance analysis: a new approach to the quantification of historical biogeography. Syst Biol.

[pbio-1000056-b031] Ree RH, Moore BR, Webb CO, Donoghue MJ (2005). A likelihood framework for inferring the evolution of geographic range on phylogenetic trees. Evolution.

[pbio-1000056-b032] Pybus OG, Harvey PH (2000). Testing macro-evolutionary models using incomplete molecular phylogenies. Proc R Soc Lond, B, Biol Sci.

[pbio-1000056-b033] Weir JT (2006). Divergent timing and patterns of species accumulation in lowland and highland neotropical birds. Evolution.

[pbio-1000056-b034] Rabosky DL, Lovette IJ (2008). Explosive evolutionary radiations: decreasing speciation or increasing extinction throught time. Evolution.

[pbio-1000056-b035] Sanchíz B, Wellnhofer P (1998). Salientia.

[pbio-1000056-b036] Rabosky DL, Donnellan SC, Talaba AL, Lovette IJ (2007). Exceptional among-lineage variation in diversification rates during the radiation of Australia's most diverse vertebrate clade. Proc R Soc Lond B Biol Sci.

[pbio-1000056-b037] Coates AG, Collins LS, Aubry MP, Berggren WA (2004). The geology of the Darien, Panama, and the late Miocene-Pliocene collision of the Panama arc with northwestern South America. Geol Soc Am Bull.

[pbio-1000056-b038] Zachos J, Pagani M, Sloan L, Thomas E, Billups K (2001). Trends, rhythms, and aberrations in global climate 65 Ma to present. Science.

[pbio-1000056-b039] Zamudio KR, Green HW (1997). Phylogeography of the bushmaster (Lachesis muta: Viperidae): implications for neotropical biogeography, systematics, and conservation. Biol J Linn Soc Lond.

[pbio-1000056-b040] Wang I, Crawford A, Bermingham E (2008). Phylogeography of the Pygmy Rain Frog (Pristimantis ridens) across the lowland wet forests of isthmian Central America. Mol Phylogenet Evol.

[pbio-1000056-b041] Kozak KH, Wiens JJ (2007). Climatic zonation drives latitudinal variation in speciation mechanisms. Proc R Soc Lond B Biol Sci.

[pbio-1000056-b042] Wiens JJ, Graham CH (2005). Niche conservatism: integrating evolution, ecology, and conservation biology. Annu Rev Ecol Syst.

[pbio-1000056-b043] Pechmann JHK, Scott DE, Semlitsch RD, Caldwell JP, Vitt LJ (1991). Declining amphibian populations: the problem of seperating human impacts from natural fluctuations. Science.

[pbio-1000056-b044] Kiesecker J, Blaustein A, Belden L (2001). Complex causes of amphibian population declines. Nature.

[pbio-1000056-b045] Pounds J, Bustamante M, Coloma L, Consuegra J, Fogden M (2006). Widespread amphibian extinctions from epidemic disease driven by global warming. Nature.

[pbio-1000056-b046] Lips K, Diffendorfer J, Mendelson J (2008). Riding the wave: reconciling the roles of disease and climate change in amphibian declines. PLoS Biol.

[pbio-1000056-b047] Hebert P, Penton E, Burns J, Janzen D, Hallwachs W (2004). Ten species in one: DNA barcoding reveals cryptic species in the neotropical skipper butterfly Astraptes fulgerator. Proc Natl Acad Sci U S A.

[pbio-1000056-b048] Señaris JC, MacCulloch R, Hollowell T, Reynolds RP (2005). Amphibians. Checklist of the terrestrial vertebrates of the Guiana Shield.

[pbio-1000056-b049] Jaramillo C, Rueda MJ, Mora G (2006). Cenozoic plant diversity in the Neotropics. Science.

[pbio-1000056-b050] Marchant R, Hooghiemstra H (2004). Rapid environmental change in African and South American tropics around 4,000 years before present: a review. Earth Sci Rev.

[pbio-1000056-b051] Fjeldså J (1994). Geographical patterns for relict and young species of birds in Africa and South America and implications for conservation priorities. Biodivers Conserv.

[pbio-1000056-b052] Richardson JE, Pennington RT, Pennington TD, Hollingsworth PM (2001). Rapid diversification of a species-rich genus of neotropical rain forest trees. Science.

[pbio-1000056-b053] Duellman WE (2005). Biogeography. Cusco Amazonico: the lives of amphibians and reptiles in an Amazonian Rainforest.

[pbio-1000056-b054] Frost DR, Grant T, Faivovich J, Bain RH, Haas A (2006). The amphibian tree of life. Bull Am Mus Nat Hist.

[pbio-1000056-b055] Darst CR, Cannatella DC (2004). Novel relationships among hyloid frogs inferred from 12S and 16S mitochondrial DNA sequences. Mol Phylogenet Evol.

[pbio-1000056-b056] Bossuyt F, Brown RM, Hillis DM, Cannatella DC, Milinkovitch MC (2006). Phylogeny and biogeography of a cosmopolitan frog radiation: lLate Cretaceous diversification resulted in continent-scale endemism in the family Ranidae. Syst Biol.

[pbio-1000056-b057] IUCN, International C, NatureServe (2006). Global amphibian assessment..

[pbio-1000056-b058] Lemmon AR, Brown JM, Stanger-Hall K, Moriarty-Lemmon EC (2008). The effect of missing data on phylogenetic estimates obtained by maximum-likelihood and bayesian inference. Syst Biol.

[pbio-1000056-b059] GeneCodes (2006). Sequencher. 4.7 ed.

[pbio-1000056-b060] Thompson JD, Gibson TJ, Plewniak F, Jeanmougin F, Higgins D (1997). The Clustal X window interface: flexible strategies for multiple sequence alignment aided by quality analysis tools. Nucleic Acids Res.

[pbio-1000056-b061] Maddison WP, Maddison DR (2001). MacClade 4: analysis of phylogeny and character evolution..

[pbio-1000056-b062] Posada D, Buckley TR (2004). Model selection and model averaging in phylogenetics: advantages of the AIC and Bayesian approaches over likelihood ratio tests. Syst Biol.

[pbio-1000056-b063] Posada D, Crandall KA (1998). Modeltest: testing the model of DNA substitution. Bioinformatics.

[pbio-1000056-b064] Zwickl D (2006). Genetic algorithm approaches for the phylogenetic analysis of large biological sequence datasets under the maximum likelihood criterion.

[pbio-1000056-b065] Huelsenbeck JP, Ronquist FP (2001). MRBAYES: Bayesian inference of phylogenetic trees. Bioinformatics.

[pbio-1000056-b066] Ronquist F, Huelsenbeck JP (2003). MRBAYES 3: Bayesian phylogenetic inference under mixed models. Bioinformatics.

[pbio-1000056-b067] Yang Z (2003). PAML: phylogenetic analysis by maximum likelihood. 3.14 ed..

[pbio-1000056-b068] Rambaut A, Drummond AJ (2007). TreeStat v1.1..

[pbio-1000056-b069] Roelants K, Gower DJ, Wilkinson M, Loader SP, Biju SD (2007). Global patterns of diversification in the history of modern amphibians. Proc Nat Acad Sci USA.

[pbio-1000056-b070] Ruta M, Coates M, Ibarra-Vidal H, Donoghue PCJ, Smith MP (2003). Bones, molecules and crown-tetrapod origins. Telling the evolutionary time: molecular clocks and the fossil record.

[pbio-1000056-b071] Ruta M, Coates MI, Quicke DLJ (2003). Early tetrapod relationships revisited. Biol Rev Camb Philos Soc.

[pbio-1000056-b072] Zhang P, Zhou H, Chen YQ, Liu YF, Qu LH (2005). Mitogenomic perspectives on the origin and phylogeny of living amphibians. Syst Biol.

[pbio-1000056-b073] Rutschmann F (2005). Bayesian dating step-by-step manual. Version 1.5, July 2005..

[pbio-1000056-b074] Sanderson MJ (2002). Estimating absolute rates of molecular evolution and divergence times: a penalized likelihood approach. Mol Biol Evol.

[pbio-1000056-b075] Sanderson MJ (2003). r8s: inferring absolute rates of molecular evolution and divergence times in the absence of a molecular clock. Bioinformatics.

[pbio-1000056-b076] Vieites DR, Min MS, Wake DB (2007). Rapid diversification and dispersal during periods of global warming by plethodontid salamanders. Proc Natl Acad Sci U S A.

[pbio-1000056-b077] Ree RH, Smith SA (2007). Lagrange: software for likelihood analysis of geographic range evolution..

[pbio-1000056-b078] Brumfield RT, Edwards SV (2007). Evolution into and out of the Andes: a Bayesian analysis of historical diversification in *Thamnophilus* antshrikes. Evolution.

[pbio-1000056-b079] Pagel M, Meade A, Barker D (2004). Bayesian estimation of ancestral character states on phylogenies. Syst Biol.

[pbio-1000056-b080] Ronquist F (2004). Bayesian inference of character evolution. Trends Ecol Evol (Amst).

[pbio-1000056-b081] Duellman WE, Duellman WE (1999). Distribution patterns of amphibians in South America. Patterns of distribution of amphibians: a global perspective.

[pbio-1000056-b082] Gentry AH (1992). Tropical forest biodiversity - distributional patterns and their conservational significance. Oikos.

[pbio-1000056-b083] Stattersfield AJ, Crosby MJ, Long AJ, Wege DC (1998). Endemic bird areas of the world.

[pbio-1000056-b084] Gutscher MA, Malavielle J, Lallemand S, Collot JY (1999). Tectonic segmentation of the North Andean margin: impact of the Carnegie Ridge collision. Earth Planet Sci Lett.

[pbio-1000056-b085] Hungerbuhler D, Steinmann M, Winkler W, Seward D, Eguez A (2002). Neogene stratigraphy and Andean geodynamics of southern Ecuador. Earth-Sci Rev.

[pbio-1000056-b086] Cozzuol MA (2006). The Acre vertebrate fauna: age, diversity, and geography. J S Am Earth Sci.

[pbio-1000056-b087] Aguilera O, Rodrigues de Aguilera D, Sanchez-Villagra MR, Clark JA (2004). New Miocene otolith-based sciaenid species (Pisces, Perciformes) from Venezuela. Fossils from the Miocene Castillo Formation, Venezuela: contributions in neotropical paleontology special papers in paleontology.

[pbio-1000056-b088] Aguilera O, Rodrigues de Aguilera D, Sanchez-Villagra MR, Clark JA (2004). Amphi-American Neogene sea catfishes (Siluriformes, Ariidae) from northern South America. Fossils from the Miocene Castillo formation, Venezuela: contributions in neotropical paleontology special papers in paleontology.

[pbio-1000056-b089] Brochu C (1997). Morphology, fossils, divergence timing, and the phylogenetic relationships of Gavialis. Syst Biol.

[pbio-1000056-b090] Diaz de Gamero ML (1995). The changing course of the Orinoco River during the Neogene: a review. Palaeogeog Palaeoclim Palaeoecol.

[pbio-1000056-b091] Cooper MA, Addison FT, Alvarez R M. C, Graham RH (1995). Basin development and tectonic history of the Llanos basin, Eastern Cordillera, and middle Magdalena Valley, Colombia. Am Assoc Pet Geol Bull.

[pbio-1000056-b092] Forero E (1988). Botanical exploration and phytogeography of Colombia: past, present, and future. Taxon.

[pbio-1000056-b093] Guerrero J, Kay RF, Madden RH, Cifelli RL, Flynn JJ (1997). Stratigraphy, sedimentary environments, and the Miocene uplift of the Colombian Andes.

[pbio-1000056-b094] Kerr AC (2005). La Isla de Gorgona, Colombia: a petrological enigma. Lithos.

[pbio-1000056-b095] Magallon S, Sanderson MJ (2001). Absolute diversification rates in angiosperm clades. Evolution.

[pbio-1000056-b096] Nee S (2001). Inferring speciation rates from phylogenies. Evolution.

[pbio-1000056-b097] Paradis E (1997). Assessing temporal variations in diversification rates from phylogenies: estimation and hypothesis testing. Proc R Soc Lond, B, Biol Sci.

[pbio-1000056-b098] Sanderson MJ, Thorne JL, Wikstrom N, Bremer K (2004). Molecular evidence on plant divergence times. Am J Bot.

[pbio-1000056-b099] Pagel M (1999). The maximum likelihood approach to reconstructing ancestral character states of discrete characters on phylogenies. Syst Biol.

[pbio-1000056-b100] Pagel M, Meade A (2006). Bayesian analysis of correlated evolution of discrete characters by reversible-jump Markov chain Monte Carlo. Am Nat.

[pbio-1000056-b101] Agapow PM, Purvis A (2002). Power of eight tree shape statistics to detect nonrandom diversification: a comparison by simulation of two models of cladogenesis. Syst Biol.

[pbio-1000056-b102] Colless DH (1982). Review of phylogentics: phylogenetics: the theory and practice of phylogenetic systematics, by E. O. Wiley. Syst Zool.

[pbio-1000056-b103] Mooers AO, Heard SB (1997). Evolutionary process from phylogenetic tree shape. Q Rev Biol.

[pbio-1000056-b104] Semple C, Steel M (2003). Phylogenetics.

[pbio-1000056-b105] Fill JA (1996). On the distribution of binary search trees under the random permutation model. Random Struct Algor.

[pbio-1000056-b106] Bortolussi N, Durand E, Blum M, Francois O (2006). apTreeshape: statistical analysis of phylogenetic tree shape. Bioinformatics.

[pbio-1000056-b107] Rabosky DL (2006). LASER: a maximum likelihood toolkit for detecting temporal shifts in diversification rates. Evol Bioinform Online.

[pbio-1000056-b108] Coloma LA (1995). Ecuadorian frogs of the genus *Colostethus* (Anura:Dendrobatidae). Misc Publ Mus Nat Hist Univ Kansas.

[pbio-1000056-b109] Morales VR (2002 [2000]). Sistematica y biogeografia del grupo *trilineatus* (Amphibia, Anura, Dendrobatidae, *Colostethus*) con descricion de once nuevas especies. Publicaciones de la Asociacion de Amigos de Doñana.

[pbio-1000056-b110] La Marca E (1995 [1994]). Taxonomy of the frogs of the genus Mannophryne (Amphibia; Anura: Dendrobatidae). Publ Asoc Amigos Donana.

[pbio-1000056-b111] Rivero JA (1990 [1988]). Sobre las relaciones de las especies del genero *Colostethus* (Amphibia, Dendrobatidae). Mem Soc Cien Nat La Salle.

[pbio-1000056-b112] Duellman WE (2004). Frogs of the genus *Colostethus* (Anura; Dendrobatidae) in the Andes of Northern Peru. Scientific papers. Lawrence (Kansas): Natural History Museum, The University of Kansas.

[pbio-1000056-b113] Raup DM (1985). Mathematical-models of cladogenesis. Paleobiology.

