# Correction: Amazonian Amphibian Diversity Is Primarily Derived from Late Miocene Andean Lineages

**DOI:** 10.1371/annotation/18e722e3-05db-4e40-86a0-bedfc4934a5a

**Published:** 2010-09-13

**Authors:** Juan C Santos, Luis A Coloma, Kyle Summers, Janalee P Caldwell, Richard Ree, David C Cannatella

In Text S1, some taxonomic names were incorrectly specified. The taxonomic corrections of Text S1 are based on the phylogeny of Figure S3. Please view the correct Text S1 here: 
