# Supplementary material for: Amazonian Amphibian Diversity Is Primarily Derived from Late Miocene Andean Lineages
Source: PLoS Biol. 2009 Mar 10;7(3):e1000056. doi: 10.1371/journal.pbio.1000056 (PMC2653552; doi:10.1371/journal.pbio.1000056)
Supplement: Supplementary file 2 [file pbio.1000056.sg005.pdf]

Fig. S5A

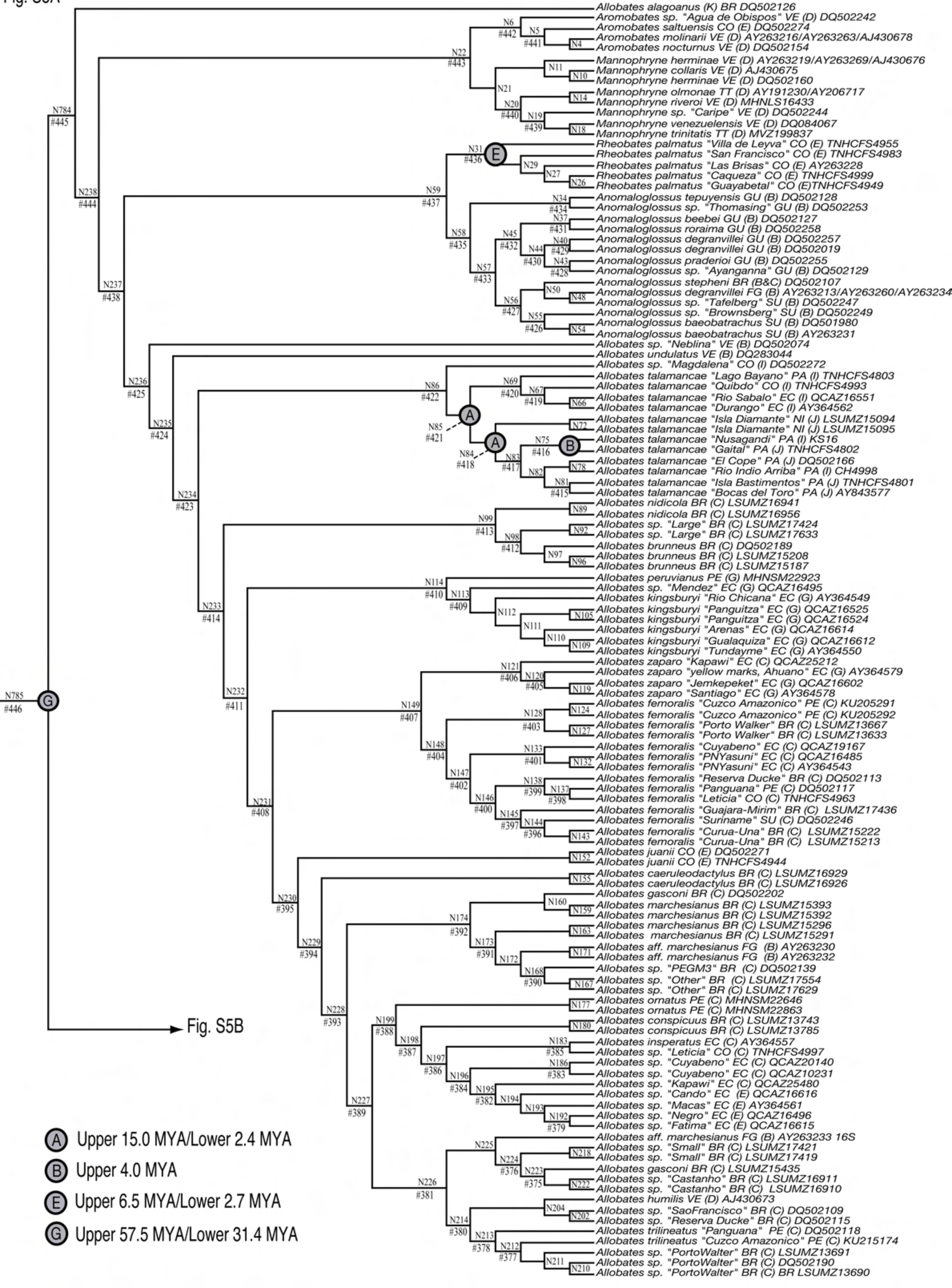

Allobates alagoanus (K) BR DQ502126
Aromobates sp. "Agua de Obispos" VE (D) DQ502242
Aromobates saltuensis CO (E) DQ502274
Aromobates molinarii VE (D) AY263216/AY263263/AJ430678
Aromobates nocturnus VE (D) DQ502154
Mannophryne herminae VE (D) AY263219/AY263269/AJ430676
Mannophryne collaris VE (D) AJ430675
Mannophryne herminae VE (D) DQ502160
Mannophryne olmonae TT (D) AY191230/AY206717
Mannophryne riveroi VE (D) MHNLS16433
Mannophryne sp. "Caripe" VE (D) DQ502244
Mannophryne venezuelensis VE (D) DQ084067
Mannophryne trinitatis TT (D) MVZ199837
Rheobates palmatus "Villa de Leyva" CO (E) TNHCFS4955
Rheobates palmatus "San Francisco" CO (E) TNHCFS4983
Rheobates palmatus "Las Brisas" CO (E) AY263228
Rheobates palmatus "Caqueza" CO (E) TNHCFS4999
Rheobates palmatus "Guayabetal" CO (E)TNHCFS4949
Anomaloglossus tepuyensis GU (B) DQ502128
Anomaloglossus sp. "Thomasing" GU (B) DQ502253
Anomaloglossus beebei GU (B) DQ502127
Anomaloglossus roraima GU (B) DQ502258
Anomaloglossus degranvillei GU (B) DQ502257
Anomaloglossus degranvillei GU (B) DQ502019
Anomaloglossus praderioi GU (B) DQ502255
Anomaloglossus sp. "Ayanganna" GU (B) DQ502129
Anomaloglossus stepheni BR (B&C) DQ502107
Anomaloglossus degranvillei FG (B) AY263213/AY263260/AY263234
Anomaloglossus sp. "Tafelberg" SU (B) DQ502247
Anomaloglossus sp. "Brownsberg" SU (B) DQ502249
Anomaloglossus baeobatrachus SU (B) DQ501980
Anomaloglossus baeobatrachus SU (B) AY263231
Allobates sp. "Neblina" VE (B) DQ502074
Allobates undulatus VE (B) DQ283044
Allobates sp. "Magdalena" CO (I) DQ502272
Allobates talamancae "Lago Bayano" PA (I) TNHCFS4803
Allobates talamancae "Quibdo" CO (I) TNHCFS4993
Allobates talamancae "Rio Sabalo" EC (I) QCAZ16551
Allobates talamancae "Durango" EC (I) AY364562
Allobates talamancae "Isla Diamante" NI (J) LSUMZ15094
Allobates talamancae "Isla Diamante" NI (J) LSUMZ15095
Allobates talamancae "Nusagandi" PA (I) KS16
Allobates talamancae "Gaital" PA (J) TNHCFS4802
Allobates talamancae "El Cope" PA (J) DQ502166
Allobates talamancae "Rio Indio Arriba" PA (I) CH4998
Allobates talamancae "Isla Bastimentos" PA (J) TNHCFS4801
Allobates talamancae "Bocas del Toro" PA (J) AY843577
Allobates nidicola BR (C) LSUMZ16941
Allobates nidicola BR (C) LSUMZ16956
Allobates sp. "Large" BR (C) LSUMZ17424
Allobates sp. "Large" BR (C) LSUMZ17633
Allobates brunneus BR (C) DQ502189
Allobates brunneus BR (C) LSUMZ15208
Allobates brunneus BR (C) LSUMZ15187
Allobates peruvianus PE (G) MHNSM22923
Allobates sp. "Mendez" EC (G) QCAZ16495
Allobates kingsburyi "Rio Chicana" EC (G) AY364549
Allobates kingsburyi "Panguitza" EC (G) QCAZ16525
Allobates kingsburyi "Panguitza" EC (G) QCAZ16524
Allobates kingsburyi "Arenas" EC (G) QCAZ16614
Allobates kingsburyi "Gualaquiza" EC (G) QCAZ16612
Allobates kingsburyi "Tundayme" EC (G) AY364550
Allobates zaparo "Kapawi" EC (C) QCAZ25212
Allobates zaparo "yellow marks, Ahuano" EC (G) AY364579
Allobates zaparo "Jemkepeket" EC (G) QCAZ16602
Allobates zaparo "Santiago" EC (G) AY364578
Allobates femoralis "Cuzco Amazonico" PE (C) KU205291
Allobates femoralis "Cuzco Amazonico" PE (C) KU205292
Allobates femoralis "Porto Walker" BR (C) LSUMZ13667
Allobates femoralis "Porto Walker" BR (C) LSUMZ13633
Allobates femoralis "Cuyabeno" EC (C) QCAZ19167
Allobates femoralis "PNYasuni" EC (C) QCAZ16485
Allobates femoralis "PNYasuni" EC (C) AY364543
Allobates femoralis "Reserva Ducke" BR (C) DQ502113
Allobates femoralis "Panguana" PE (C) DQ502117
Allobates femoralis "Leticia" CO (C) TNHCFS4963
Allobates femoralis "Guajara-Mirim" BR (C) LSUMZ17436
Allobates femoralis "Suriname" SU (C) DQ502246
Allobates femoralis "Curua-Una" BR (C) LSUMZ15222
Allobates femoralis "Curua-Una" BR (C) LSUMZ15213
Allobates juanii CO (E) DQ502271
Allobates juanii CO (E) TNHCFS4944
Allobates caeruleodactylus BR (C) LSUMZ16929
Allobates caeruleodactylus BR (C) LSUMZ16926
Allobates gasconi BR (C) DQ502202
Allobates marchesianus BR (C) LSUMZ15393
Allobates marchesianus BR (C) LSUMZ15392
Allobates marchesianus BR (C) LSUMZ15296
Allobates marchesianus BR (C) LSUMZ15291
Allobates aff. marchesianus FG (B) AY263230
Allobates aff. marchesianus FG (B) AY263232
Allobates sp. "PEGM3" BR (C) DQ502139
Allobates sp. "Other" BR (C) LSUMZ17554
Allobates sp. "Other" BR (C) LSUMZ17629
Allobates ornatus PE (C) MHNSM22646
Allobates ornatus PE (C) MHNSM22863
Allobates conspicuus BR (C) LSUMZ13743
Allobates conspicuus BR (C) LSUMZ13785
Allobates insperatus EC (C) AY364557
Allobates sp. "Leticia" CO (C) TNHCFS4997
Allobates sp. "Cuyabeno" EC (C) QCAZ20140
Allobates sp. "Cuyabeno" EC (C) QCAZ10231
Allobates sp. "Kapawi" EC (C) QCAZ25480
Allobates sp. "Cando" EC (E) QCAZ16616
Allobates sp. "Macas" EC (E) AY364561
Allobates sp. "Negro" EC (E) QCAZ16496
Allobates sp. "Fatima" EC (E) QCAZ16615
Allobates aff. marchesianus FG (B) AY263233 16S
Allobates sp. "Small" BR (C) LSUMZ17421
Allobates sp. "Small" BR (C) LSUMZ17419
Allobates gasconi BR (C) LSUMZ15435
Allobates sp. "Castanho" BR (C) LSUMZ16911
Allobates sp. "Castanho" BR (C) LSUMZ16910
Allobates humilis VE (D) AJ430673
Allobates sp. "SaoFrancisco" BR (C) DQ502109
Allobates sp. "Reserva Ducke" BR (C) DQ502115
Allobates trilineatus "Panguana" PE (C) DQ502118
Allobates trilineatus "Cuzco Amazonico" PE (C) KU215174
Allobates sp. "PortoWalter" BR (C) LSUMZ13691
Allobates sp. "PortoWalter" BR (C) DQ502190
Allobates sp. "PortoWalter" BR (C) BR LSUMZ13690
Fig. S5B
A Upper 15.0 MYA/Lower 2.4 MYA
B Upper 4.0 MYA
E Upper 6.5 MYA/Lower 2.7 MYA
G Upper 57.5 MYA/Lower 31.4 MYA

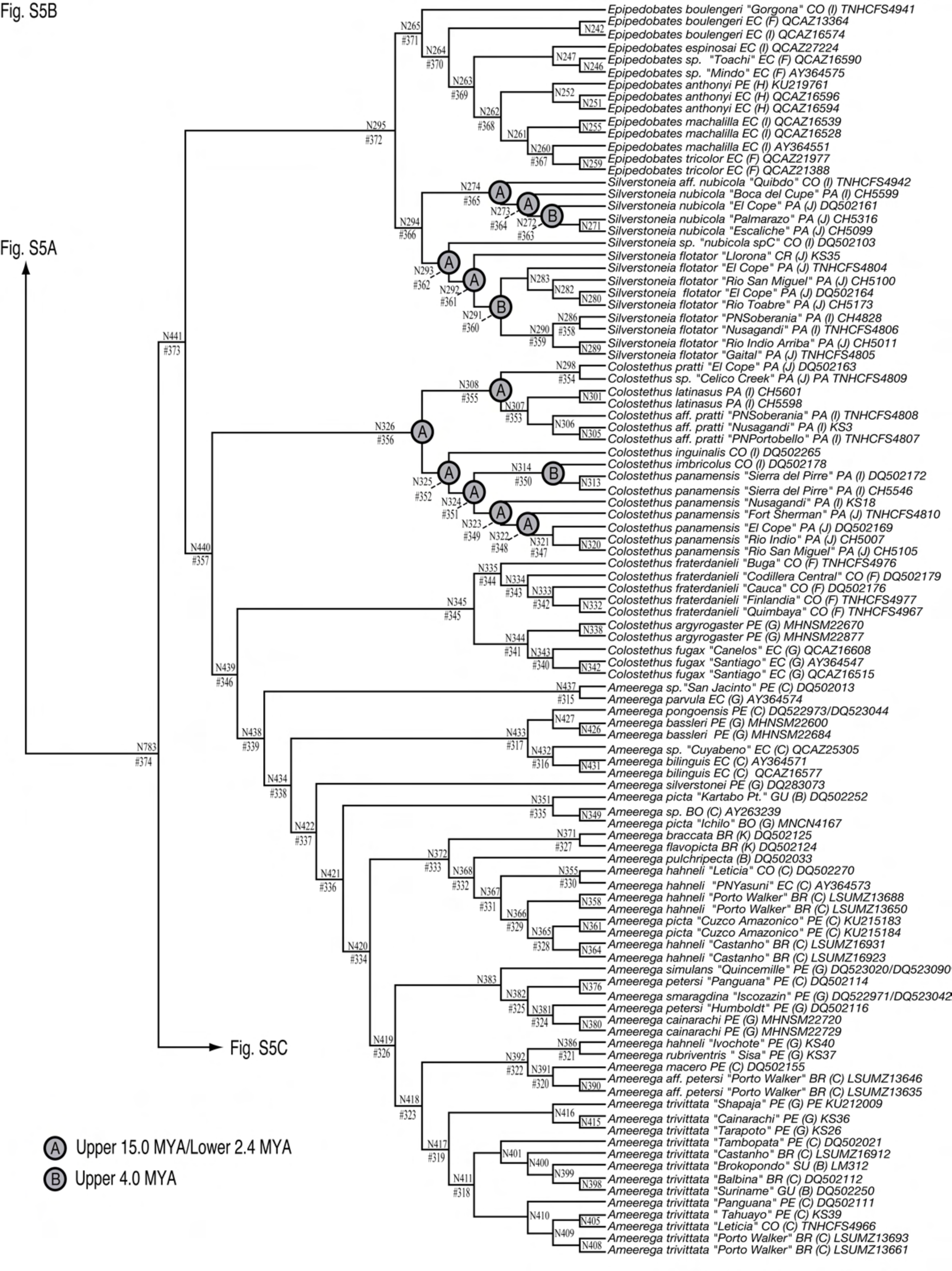
Fig. S5B
Fig. S5A
Fig. S5C
Epipedobates boulengeri "Gorgona" CO (I) TNHCFS4941
Epipedobates boulengeri EC (F) QCAZ13364
Epipedobates boulengeri EC (I) QCAZ16574
Epipedobates espinosai EC (I) QCAZ27224
Epipedobates sp. "Toachi" EC (F) QCAZ16590
Epipedobates sp. "Mindo" EC (F) AY364575
Epipedobates anthonyi PE (H) KU219761
Epipedobates anthonyi EC (H) QCAZ16596
Epipedobates anthonyi EC (H) QCAZ16594
Epipedobates machalilla EC (I) QCAZ16539
Epipedobates machalilla EC (I) QCAZ16528
Epipedobates machalilla EC (I) AY364551
Epipedobates tricolor EC (F) QCAZ21977
Epipedobates tricolor EC (F) QCAZ21388
Silverstoneia aff. nubicola "Quibdo" CO (I) TNHCFS4942
Silverstoneia nubicola "Boca del Cupe" PA (I) CH5599
Silverstoneia nubicola "El Cope" PA (J) DQ502161
Silverstoneia nubicola "Palmarazo" PA (J) CH5316
Silverstoneia nubicola "Escaliche" PA (J) CH5099
Silverstoneia sp. "nubicola spC" CO (I) DQ502103
Silverstoneia flotator "Llorona" CR (J) KS35
Silverstoneia flotator "El Cope" PA (J) TNHCFS4804
Silverstoneia flotator "Rio San Miguel" PA (J) CH5100
Silverstoneia flotator "El Cope" PA (J) DQ502164
Silverstoneia flotator "Rio Toabre" PA (J) CH5173
Silverstoneia flotator "PNSoberania" PA (I) CH4828
Silverstoneia flotator "Nusagandi" PA (I) TNHCFS4806
Silverstoneia flotator "Rio Indio Arriba" PA (J) CH5011
Silverstoneia flotator "Gaital" PA (J) TNHCFS4805
Colostethus pratti "El Cope" PA (J) DQ502163
Colostethus sp. "Celico Creek" PA (J) PA TNHCFS4809
Colostethus latinasus PA (I) CH5601
Colostethus latinasus PA (I) CH5598
Colostethus aff. pratti "PNSoberania" PA (I) TNHCFS4808
Colostethus aff. pratti "Nusagandi" PA (I) KS3
Colostethus aff. pratti "PNPortobello" PA (I) TNHCFS4807
Colostethus inguinalis CO (I) DQ502265
Colostethus imbricolus CO (I) DQ502178
Colostethus panamensis "Sierra del Pirre" PA (I) DQ502172
Colostethus panamensis "Sierra del Pirre" PA (I) CH5546
Colostethus panamensis "Nusagandi" PA (I) KS18
Colostethus panamensis "Fort Sherman" PA (J) TNHCFS4810
Colostethus panamensis "El Cope" PA (J) DQ502169
Colostethus panamensis "Rio Indio" PA (J) CH5007
Colostethus panamensis "Rio San Miguel" PA (J) CH5105
Colostethus fraterdanieli "Buga" CO (F) TNHCFS4976
Colostethus fraterdanieli "Codillera Central" CO (F) DQ502179
Colostethus fraterdanieli "Cauca" CO (F) DQ502176
Colostethus fraterdanieli "Finlandia" CO (F) TNHCFS4977
Colostethus fraterdanieli "Quimbaya" CO (F) TNHCFS4967
Colostethus argyrogaster PE (G) MHNSM22670
Colostethus argyrogaster PE (G) MHNSM22877
Colostethus fugax "Canelos" EC (G) QCAZ16608
Colostethus fugax "Santiago" EC (G) AY364547
Colostethus fugax "Santiago" EC (G) QCAZ16515
Ameerega sp."San Jacinto" PE (C) DQ502013
Ameerega parvula EC (G) AY364574
Ameerega pongoensis PE (C) DQ522973/DQ523044
Ameerega bassleri PE (G) MHNSM22600
Ameerega bassleri PE (G) MHNSM22684
Ameerega sp. "Cuyabeno" EC (C) QCAZ25305
Ameerega bilinguis EC (C) AY364571
Ameerega bilinguis EC (C) QCAZ16577
Ameerega silverstonei PE (G) DQ283073
Ameerega picta "Kartabo Pt." GU (B) DQ502252
Ameerega sp. BO (C) AY263239
Ameerega picta "Ichilo" BO (G) MNCN4167
Ameerega braccata BR (K) DQ502125
Ameerega flavopicta BR (K) DQ502124
Ameerega pulchripecta (B) DQ502033
Ameerega hahneli "Leticia" CO (C) DQ502270
Ameerega hahneli "PNYasuni" EC (C) AY364573
Ameerega hahneli "Porto Walker" BR (C) LSUMZ13688
Ameerega hahneli "Porto Walker" BR (C) LSUMZ13650
Ameerega picta "Cuzco Amazonico" PE (C) KU215183
Ameerega picta "Cuzco Amazonico" PE (C) KU215184
Ameerega hahneli "Castanho" BR (C) LSUMZ16931
Ameerega hahneli "Castanho" BR (C) LSUMZ16923
Ameerega simulans "Quincemille" PE (G) DQ523020/DQ523090
Ameerega petersi "Panguana" PE (C) DQ502114
Ameerega smaragdina "Iscozazin" PE (G) DQ522971/DQ523042
Ameerega petersi "Humboldt" PE (G) DQ502116
Ameerega cainarachi PE (G) MHNSM22720
Ameerega cainarachi PE (G) MHNSM22729
Ameerega hahneli "Ivochote" PE (G) KS40
Ameerega rubriventris " Sisa" PE (G) KS37
Ameerega macero PE (C) DQ502155
Ameerega aff. petersi "Porto Walker" BR (C) LSUMZ13646
Ameerega aff. petersi "Porto Walker" BR (C) LSUMZ13635
Ameerega trivittata "Shapaja" PE (G) PE KU212009
Ameerega trivittata "Cainarachi" PE (G) KS36
Ameerega trivittata "Tarapoto" PE (G) KS26
Ameerega trivittata "Tambopata" PE (C) DQ502021
Ameerega trivittata "Castanho" BR (C) LSUMZ16912
Ameerega trivittata "Brokopondo" SU (B) LM312
Ameerega trivittata "Balbina" BR (C) DQ502112
Ameerega trivittata "Suriname" GU (B) DQ502250
Ameerega trivittata "Panguana" PE (C) DQ502111
Ameerega trivittata " Tahuayo" PE (C) KS39
Ameerega trivittata "Leticia" CO (C) TNHCFS4966
Ameerega trivittata "Porto Walker" BR (C) LSUMZ13693
Ameerega trivittata "Porto Walker" BR (C) LSUMZ13661
A Upper 15.0 MYA/Lower 2.4 MYA
B Upper 4.0 MYA

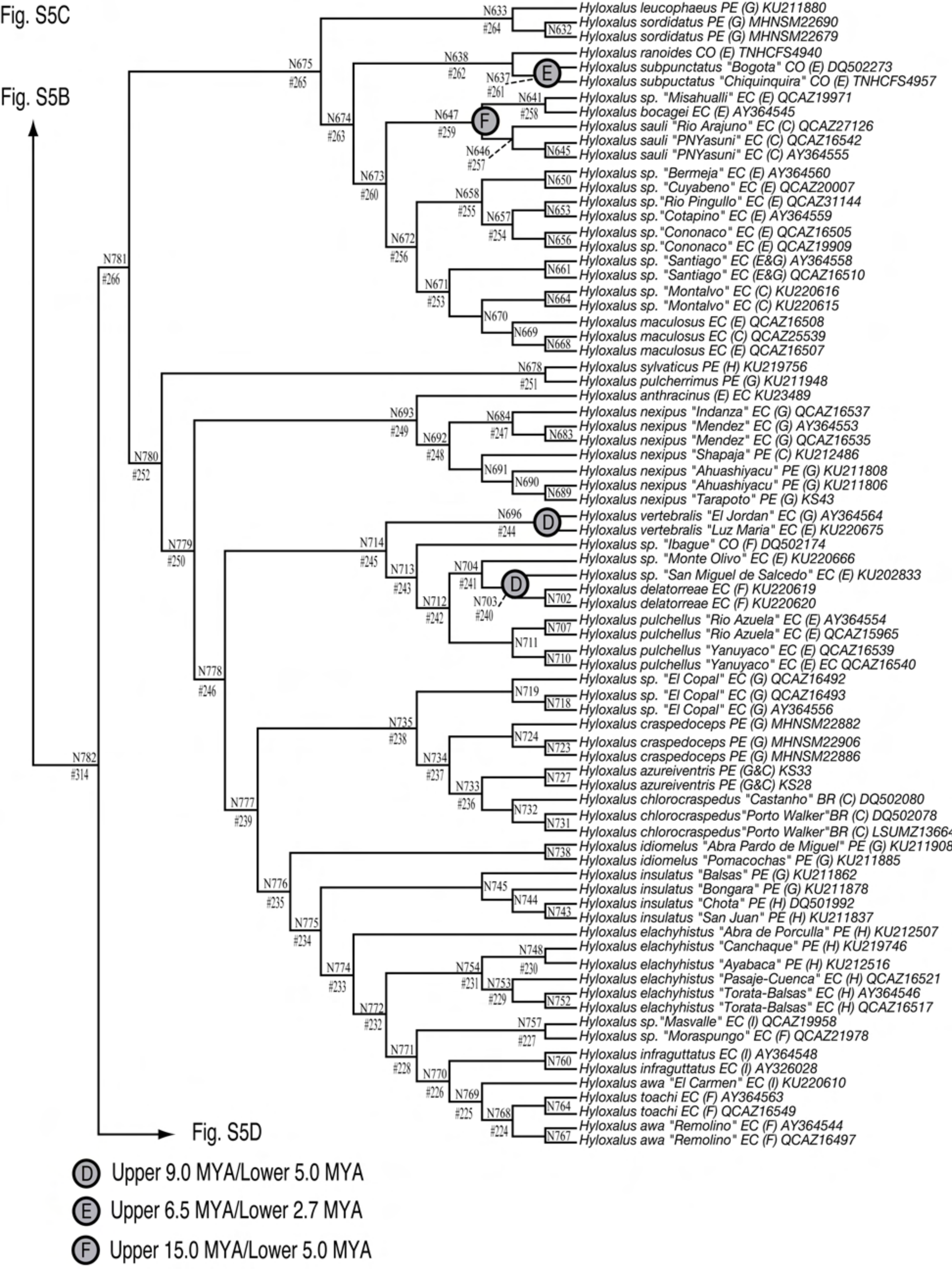
Fig. S5C
Fig. S5B
Fig. S5D
Hyloxalus leucophaeus PE (G) KU211880
Hyloxalus sordidatus PE (G) MHNSM22690
Hyloxalus sordidatus PE (G) MHNSM22679
Hyloxalus ranoides CO (E) TNHCFS4940
Hyloxalus subpunctatus "Bogota" CO (E) DQ502273
Hyloxalus subpuctatus "Chiquinquira" CO (E) TNHCFS4957
Hyloxalus sp. "Misahualli" EC (E) QCAZ19971
Hyloxalus bocagei EC (E) AY364545
Hyloxalus sauli "Rio Arajuno" EC (C) QCAZ27126
Hyloxalus sauli "PNYasuni" EC (C) QCAZ16542
Hyloxalus sauli "PNYasuni" EC (C) AY364555
Hyloxalus sp. "Bermeja" EC (E) AY364560
Hyloxalus sp. "Cuyabeno" EC (E) QCAZ20007
Hyloxalus sp."Rio Pingullo" EC (E) QCAZ31144
Hyloxalus sp."Cotapino" EC (E) AY364559
Hyloxalus sp."Cononaco" EC (E) QCAZ16505
Hyloxalus sp."Cononaco" EC (E) QCAZ19909
Hyloxalus sp. "Santiago" EC (E&G) AY364558
Hyloxalus sp. "Santiago" EC (E&G) QCAZ16510
Hyloxalus sp. "Montalvo" EC (C) KU220616
Hyloxalus sp. "Montalvo" EC (C) KU220615
Hyloxalus maculosus EC (E) QCAZ16508
Hyloxalus maculosus EC (C) QCAZ25539
Hyloxalus maculosus EC (E) QCAZ16507
Hyloxalus sylvaticus PE (H) KU219756
Hyloxalus pulcherrimus PE (G) KU211948
Hyloxalus anthracinus (E) EC KU23489
Hyloxalus nexipus "Indanza" EC (G) QCAZ16537
Hyloxalus nexipus "Mendez" EC (G) AY364553
Hyloxalus nexipus "Mendez" EC (G) QCAZ16535
Hyloxalus nexipus "Shapaja" PE (C) KU212486
Hyloxalus nexipus "Ahuashiyacu" PE (G) KU211808
Hyloxalus nexipus "Ahuashiyacu" PE (G) KU211806
Hyloxalus nexipus "Tarapoto" PE (G) KS43
Hyloxalus vertebralis "El Jordan" EC (G) AY364564
Hyloxalus vertebralis "Luz Maria" EC (E) KU220675
Hyloxalus sp. "Ibague" CO (F) DQ502174
Hyloxalus sp. "Monte Olivo" EC (E) KU220666
Hyloxalus sp. "San Miguel de Salcedo" EC (E) KU202833
Hyloxalus delatorreae EC (F) KU220619
Hyloxalus delatorreae EC (F) KU220620
Hyloxalus pulchellus "Rio Azuela" EC (E) AY364554
Hyloxalus pulchellus "Rio Azuela" EC (E) QCAZ15965
Hyloxalus pulchellus "Yanuyaco" EC (E) QCAZ16539
Hyloxalus pulchellus "Yanuyaco" EC (E) EC QCAZ16540
Hyloxalus sp. "El Copal" EC (G) QCAZ16492
Hyloxalus sp. "El Copal" EC (G) QCAZ16493
Hyloxalus sp. "El Copal" EC (G) AY364556
Hyloxalus craspedoceps PE (G) MHNSM22882
Hyloxalus craspedoceps PE (G) MHNSM22906
Hyloxalus craspedoceps PE (G) MHNSM22886
Hyloxalus azureiventris PE (G&C) KS33
Hyloxalus azureiventris PE (G&C) KS28
Hyloxalus chlorocraspedus "Castanho" BR (C) DQ502080
Hyloxalus chlorocraspedus"Porto Walker"BR (C) DQ502078
Hyloxalus chlorocraspedus"Porto Walker"BR (C) LSUMZ13664
Hyloxalus idiomelus "Abra Pardo de Miguel" PE (G) KU211908
Hyloxalus idiomelus "Pomacochas" PE (G) KU211885
Hyloxalus insulatus "Balsas" PE (G) KU211862
Hyloxalus insulatus "Bongara" PE (G) KU211878
Hyloxalus insulatus "Chota" PE (H) DQ501992
Hyloxalus insulatus "San Juan" PE (H) KU211837
Hyloxalus elachyhistus "Abra de Porculla" PE (H) KU212507
Hyloxalus elachyhistus "Canchaque" PE (H) KU219746
Hyloxalus elachyhistus "Ayabaca" PE (H) KU212516
Hyloxalus elachyhistus "Pasaje-Cuenca" EC (H) QCAZ16521
Hyloxalus elachyhistus "Torata-Balsas" EC (H) AY364546
Hyloxalus elachyhistus "Torata-Balsas" EC (H) QCAZ16517
Hyloxalus sp."Masvalle" EC (I) QCAZ19958
Hyloxalus sp. "Moraspungo" EC (F) QCAZ21978
Hyloxalus infraguttatus EC (I) AY364548
Hyloxalus infraguttatus EC (I) AY326028
Hyloxalus awa "El Carmen" EC (I) KU220610
Hyloxalus toachi EC (F) AY364563
Hyloxalus toachi EC (F) QCAZ16549
Hyloxalus awa "Remolino" EC (F) AY364544
Hyloxalus awa "Remolino" EC (F) QCAZ16497
D Upper 9.0 MYA/Lower 5.0 MYA
E Upper 6.5 MYA/Lower 2.7 MYA
F Upper 15.0 MYA/Lower 5.0 MYA

Fig. S5D

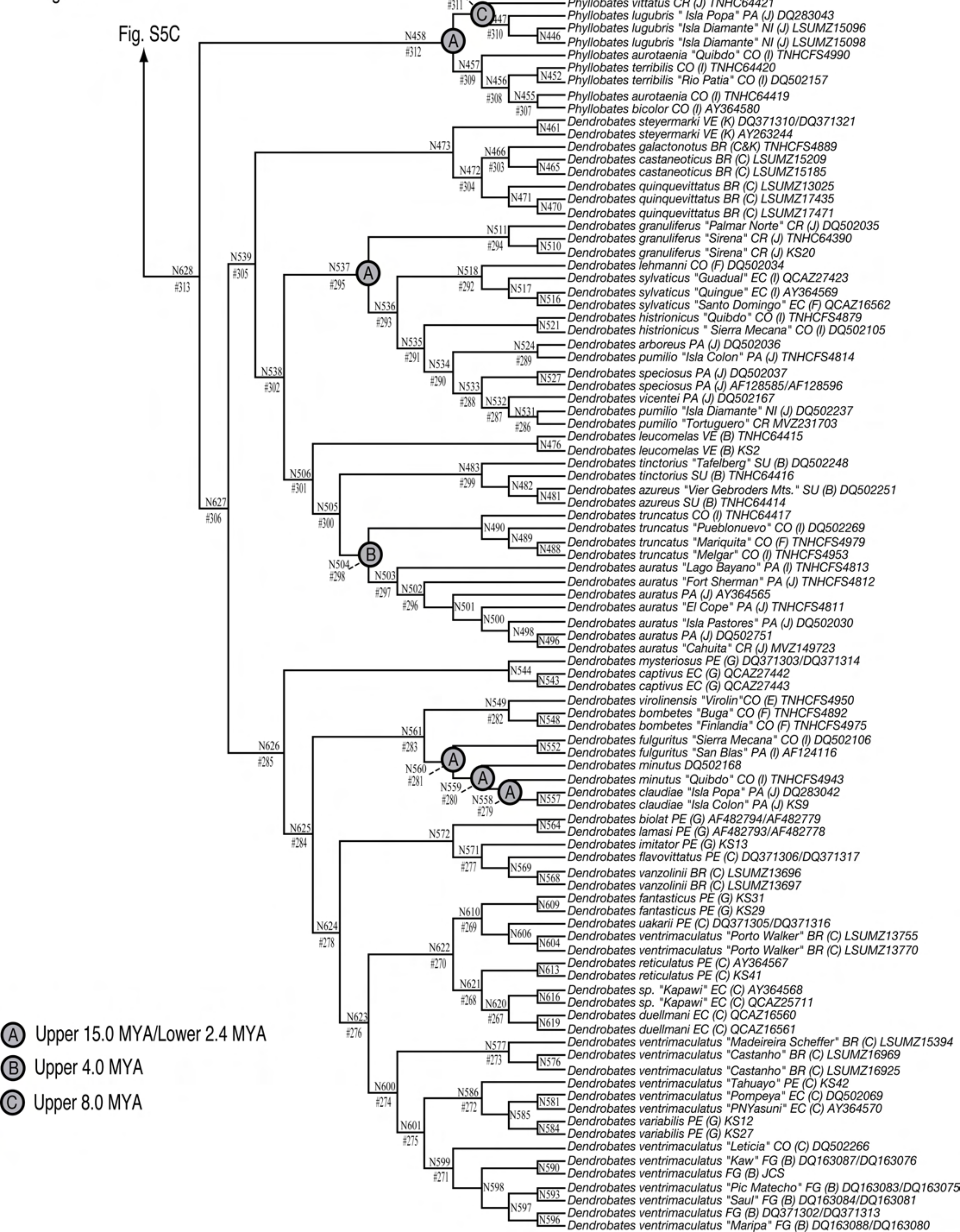

Fig. S5C
Phyllobates vittatus CR (J) TNHC64421
Phyllobates lugubris " Isla Popa" PA (J) DQ283043
Phyllobates lugubris "Isla Diamante" NI (J) LSUMZ15096
Phyllobates lugubris "Isla Diamante" NI (J) LSUMZ15098
Phyllobates aurotaenia "Quibdo" CO (I) TNHCFS4990
Phyllobates terribilis CO (I) TNHC64420
Phyllobates terribilis "Rio Patia" CO (I) DQ502157
Phyllobates aurotaenia CO (I) TNHC64419
Phyllobates bicolor CO (I) AY364580
Dendrobates steyermarki VE (K) DQ371310/DQ371321
Dendrobates steyermarki VE (K) AY263244
Dendrobates galactonotus BR (C&K) TNHCFS4889
Dendrobates castaneoticus BR (C) LSUMZ15209
Dendrobates castaneoticus BR (C) LSUMZ15185
Dendrobates quinquevittatus BR (C) LSUMZ13025
Dendrobates quinquevittatus BR (C) LSUMZ17435
Dendrobates quinquevittatus BR (C) LSUMZ17471
Dendrobates granuliferus "Palmar Norte" CR (J) DQ502035
Dendrobates granuliferus "Sirena" CR (J) TNHC64390
Dendrobates granuliferus "Sirena" CR (J) KS20
Dendrobates lehmanni CO (F) DQ502034
Dendrobates sylvaticus "Guadual" EC (I) QCAZ27423
Dendrobates sylvaticus "Quingue" EC (I) AY364569
Dendrobates sylvaticus "Santo Domingo" EC (F) QCAZ16562
Dendrobates histrionicus "Quibdo" CO (I) TNHCFS4879
Dendrobates histrionicus " Sierra Mecana" CO (I) DQ502105
Dendrobates arboreus PA (J) DQ502036
Dendrobates pumilio "Isla Colon" PA (J) TNHCFS4814
Dendrobates speciosus PA (J) DQ502037
Dendrobates speciosus PA (J) AF128585/AF128596
Dendrobates vicentei PA (J) DQ502167
Dendrobates pumilio "Isla Diamante" NI (J) DQ502237
Dendrobates pumilio "Tortuguero" CR MVZ231703
Dendrobates leucomelas VE (B) TNHC64415
Dendrobates leucomelas VE (B) KS2
Dendrobates tinctorius "Tafelberg" SU (B) DQ502248
Dendrobates tinctorius SU (B) TNHC64416
Dendrobates azureus "Vier Gebroders Mts." SU (B) DQ502251
Dendrobates azureus SU (B) TNHC64414
Dendrobates truncatus CO (I) TNHC64417
Dendrobates truncatus "Pueblonuevo" CO (I) DQ502269
Dendrobates truncatus "Mariquita" CO (F) TNHCFS4979
Dendrobates truncatus "Melgar" CO (I) TNHCFS4953
Dendrobates auratus "Lago Bayano" PA (I) TNHCFS4813
Dendrobates auratus "Fort Sherman" PA (J) TNHCFS4812
Dendrobates auratus PA (J) AY364565
Dendrobates auratus "El Cope" PA (J) TNHCFS4811
Dendrobates auratus "Isla Pastores" PA (J) DQ502030
Dendrobates auratus PA (J) DQ502751
Dendrobates auratus "Cahuita" CR (J) MVZ149723
Dendrobates mysteriosus PE (G) DQ371303/DQ371314
Dendrobates captivus EC (G) QCAZ27442
Dendrobates captivus EC (G) QCAZ27443
Dendrobates virolinensis "Virolin"CO (E) TNHCFS4950
Dendrobates bombetes "Buga" CO (F) TNHCFS4892
Dendrobates bombetes "Finlandia" CO (F) TNHCFS4975
Dendrobates fulguritus "Sierra Mecana" CO (I) DQ502106
Dendrobates fulguritus "San Blas" PA (I) AF124116
Dendrobates minutus DQ502168
Dendrobates minutus "Quibdo" CO (I) TNHCFS4943
Dendrobates claudiae "Isla Popa" PA (J) DQ283042
Dendrobates claudiae "Isla Colon" PA (J) KS9
Dendrobates biolat PE (G) AF482794/AF482779
Dendrobates lamasi PE (G) AF482793/AF482778
Dendrobates imitator PE (G) KS13
Dendrobates flavovittatus PE (C) DQ371306/DQ371317
Dendrobates vanzolinii BR (C) LSUMZ13696
Dendrobates vanzolinii BR (C) LSUMZ13697
Dendrobates fantasticus PE (G) KS31
Dendrobates fantasticus PE (G) KS29
Dendrobates uakarii PE (C) DQ371305/DQ371316
Dendrobates ventrimaculatus "Porto Walker" BR (C) LSUMZ13755
Dendrobates ventrimaculatus "Porto Walker" BR (C) LSUMZ13770
Dendrobates reticulatus PE (C) AY364567
Dendrobates reticulatus PE (C) KS41
Dendrobates sp. "Kapawi" EC (C) AY364568
Dendrobates sp. "Kapawi" EC (C) QCAZ25711
Dendrobates duellmani EC (C) QCAZ16560
Dendrobates duellmani EC (C) QCAZ16561
Dendrobates ventrimaculatus "Madeireira Scheffer" BR (C) LSUMZ15394
Dendrobates ventrimaculatus "Castanho" BR (C) LSUMZ16969
Dendrobates ventrimaculatus "Castanho" BR (C) LSUMZ16925
Dendrobates ventrimaculatus "Tahuayo" PE (C) KS42
Dendrobates ventrimaculatus "Pompeya" EC (C) DQ502069
Dendrobates ventrimaculatus "PNYasuni" EC (C) AY364570
Dendrobates variabilis PE (G) KS12
Dendrobates variabilis PE (G) KS27
Dendrobates ventrimaculatus "Leticia" CO (C) DQ502266
Dendrobates ventrimaculatus "Kaw" FG (B) DQ163087/DQ163076
Dendrobates ventrimaculatus FG (B) JCS
Dendrobates ventrimaculatus "Pic Matecho" FG (B) DQ163083/DQ163075
Dendrobates ventrimaculatus "Saul" FG (B) DQ163084/DQ163081
Dendrobates ventrimaculatus FG (B) DQ371302/DQ371313
Dendrobates ventrimaculatus "Maripa" FG (B) DQ163088/DQ163080
A Upper 15.0 MYA/Lower 2.4 MYA
B Upper 4.0 MYA
C Upper 8.0 MYA
