# Supplementary material for: Amazonian Amphibian Diversity Is Primarily Derived from Late Miocene Andean Lineages
Source: PLoS Biol. 2009 Mar 10;7(3):e1000056. doi: 10.1371/journal.pbio.1000056 (PMC2653552; doi:10.1371/journal.pbio.1000056)
Supplement: Supplementary file 3 [file pbio.1000056.st001.pdf]

Table S1. Tree imbalance indices (Colless I_C, Sackin I_S, and likelihood shape *s*) estimated for the poison frog chronogram, the trees for each super-region and the corresponding probability of rejecting the null Equal Markov Rate. An asterisk indicates significance at $\alpha = 0.05$.

Super-Region	I_C	*p*	I_S	*p*	*s*	*p*
Amazon Basin	0.235	0.342	0.329	0.061	1.175	0.120
Andes	0.949	0.109	0.511	0.041*	1.486	0.069
Central America-Chocó	-0.060	0.489	-0.118	0.161	0.449	0.327
Guiana-Venezuela-Brazilian Shield	0.148	0.382	0.043	0.092	0.059	0.476
Entire Chronogram	0.431	0.268	0.345	0.069	1.914	0.028*
Entire ML Phylogeny	0.575	0.215	0.489	0.054	1.789	0.037*
