# Supplementary material for: Amazonian Amphibian Diversity Is Primarily Derived from Late Miocene Andean Lineages
Source: PLoS Biol. 2009 Mar 10;7(3):e1000056. doi: 10.1371/journal.pbio.1000056 (PMC2653552; doi:10.1371/journal.pbio.1000056)
Supplement: Supplementary file 4 [file pbio.1000056.st002.pdf]

Table S2. Taxa used to infer the age of the root of the poison frog tree

Tree #	Order	Family	Genus	Species	1216S	RAG
17	Anura	Alytidae	*Alytes*	*obstetricans*	NC006688	AY364200
18	Anura	Alytidae	*Discoglossus*	*galganoi*	NC006690	AY583338
19	Anura	Alytidae	*Discoglossus*	*pictus*	AY523761 DQ283435	AY364202
68	Anura	Arthroleptidae	*Arthroleptis*	*variabilis*	DQ283081	AY571642
69	Anura	Arthroleptidae	*Trichobatrachus*	*robustus*	AY843773	AY364212
25	Anura	Batrachophrynidae	*Caudiverbera*	*caudiverbera*	DQ283439	AY583337
16	Anura	Bombinatoridae	*Bombina*	*orientalis*	AY957562	AY583335
67	Anura	Brevicipitidae	*Callulina*	*kreffti*	AY326068	DQ347281
55	Anura	Bufonidae	*Atelopus*	*peruensis*	AY819329 DQ158419	DQ158345
58	Anura	Bufonidae	*Bufo*	*marinus*	AY325994	DQ158392
59	Anura	Bufonidae	*Bufo*	*boreas*	AY325983	DQ158360
60	Anura	Bufonidae	*Bufo*	*haematiticus*	AY680270	DQ158383
61	Anura	Bufonidae	*Bufo*	*bufo*	AY325988	AY583336
56	Anura	Bufonidae	*Dendrophryniscus*	*minutus*	AY843582	DQ158346
57	Anura	Bufonidae	*Melanophryniscus*	*stelzneri*	AY325999	DQ158347
36	Anura	Centrolenidae	*Centrolene*	*prosoblepon*	AY843574	AY364223
71	Anura	Ceratobatrachidae	*Ceratobatrachus*	*guentheri*	DQ283198	DQ019496
70	Anura	Ceratobatrachidae	*Ingerana*	*baluensis*	DQ283142	DQ347270
49	Anura	Ceratophryidae	*Atelognathus*	*patagonicus*	AY843571	*
34	Anura	Ceratophryidae	*Ceratophrys*	*ornata*	AY326013	AY364218
43	Anura	Ceratophryidae	*Telmatobius*	*niger*	AY326015	DQ347275
52	Anura	Cycloramphidae	*Alsodes*	*monticola*	AY326016	*
53	Anura	Cycloramphidae	*Odontophrynus*	*americanus*	AY843704	*
35	Anura	Cycloramphidae	*Rhinoderma*	*darwinii*	DQ283324	AY364222
50	Anura	Cycloramphidae	*Thoropa*	*miliaris*	DQ283331	*
44	Anura	Dendrobatidae	*Allobates*	*femoralis*	AY364543	*
45	Anura	Dendrobatidae	*Colostethus*	*awa*	AY364544	*
46	Anura	Dendrobatidae	*Colostethus*	*fugax*	AY364547	*
48	Anura	Dendrobatidae	*Dendrobates*	*auratus*	AY326036	AY364214
47	Anura	Dendrobatidae	*Phyllobates*	*vittatus*	DQ502152	*
83	Anura	Dicroglossidae	*Fejervarya*	*nicobariensis*	AY326062	DQ347274
73	Anura	Dicroglossidae	*Hoplobatrachus*	*occipitalis*	DQ283059	AY571650
72	Anura	Dicroglossidae	*Limnonectes*	*magnus*	AY313706	DQ347252
26	Anura	Heleophrynidae	*Heleophryne*	*purcelli*	AY326072	AY364221
66	Anura	Hemisotidae	*Hemisus*	*marmoratum*	AY326070	AY364216
29	Anura	Hylidae	*Agalychnis*	*callidryas*	AY843563	AY323765
32	Anura	Hylidae	*Hyla*	*cinerea*	AY549327	AY323766
33	Anura	Hylidae	*Hyla*	*arenicolor*	AY843603	AY364220
28	Anura	Hylidae	*Litoria*	*caerulea*	AY843692	AY323767
31	Anura	Hylidae	*Pseudis*	*paradoxa*	AY326032	AY323773
30	Anura	Hylidae	*Trachycephalus*	*venulosa*	AY326048	AY364215
10	Anura	Leiopelmatidae	*Ascaphus*	*truei*	AJ871087	AY323754
11	Anura	Leiopelmatidae	*Leiopelma*	*archeyi*	DQ283216 DQ283215 AY523785	AY523748
37	Anura	Leptodactylidae	*Adenomera*	*andreae*	AY364538	*
41	Anura	Leptodactylidae	*Edalorhina*	*perezi*	AY843585	*
42	Anura	Leptodactylidae	*Engystomops*	*pustulosus*	DQ337241	*
39	Anura	Leptodactylidae	*Leptodactylus*	*ocellatus*	DQ158417	DQ158343

* Please, request this sequence to David Cannatella <catfish@mail.utexas.edu>

Table S2 (Cont). Taxa used to infer the age of the root of the poison frog tree

Tree #	Order	Family	Genus	Species	1216S	RAG
40	Anura	Leptodactylidae	*Leptodactylus*	*fuscus*	DQ283404	AY323770
38	Anura	Leptodactylidae	*Lithodytes*	*lineatus*	AY843690	*
51	Anura	Leptodactylidae	*Pleurodema*	*brachyops*	AY843733	*
54	Anura	Leptodactylidae	*Pseudopaludicola*	*falcipes*	AY843741	*
27	Anura	Limnodynastidae	*Limnodynastes*	*salminii*	AY326071	AY364219
78	Anura	Mantellidae	*Aglyptodactylus*	*madagascariensis*	DQ283056	AY571640
81	Anura	Mantellidae	*Boophis*	*tephraeomystax*	DQ283032	DQ347234
79	Anura	Mantellidae	*Laliostoma*	*labrosum*	DQ283057	AY571652
80	Anura	Mantellidae	*Mantella*	*nigricans*	DQ283034	AY723527
21	Anura	Megophryidae	*Megophrys*	*montana*	*	AY323760
63	Anura	Microhylidae	*Elachistocleis*	*ovalis*	DQ283405	DQ347282
64	Anura	Microhylidae	*Gastrophryne*	*olivacea*	AY326066	DQ347280
65	Anura	Microhylidae	*Kaloula*	*pulchra*	NC006405	AY323772
62	Anura	Microhylidae	*Scaphiophryne*	*marmorata*	AY843751	AY364205
22	Anura	Pelobatidae	*Pelobates*	*cultripes*	NC008144	AY364201
20	Anura	Pelodytidae	*Pelodytes*	*punctatus*	DQ283111	AY364203
82	Anura	Phrynobatrachidae	*Phrynobatrachus*	*natalensis*	DQ283414	DQ019507
14	Anura	Pipidae	*Hymenochirus*	*boettgeri*	AY581623	AY583340
15	Anura	Pipidae	*Pipa*	*pipa*	AY581621	AY874303
13	Anura	Pipidae	*Silurana*	*tropicalis*	NC006839	AY874307
74	Anura	Pyxicephalidae	*Afrana*	*fuscigula*	DQ283069	DQ347290
75	Anura	Pyxicephalidae	*Strongylopus*	*grayii*	DQ283068	DQ347256
84	Anura	Ranidae	*Rana*	*galamensis*	DQ283058	DQ347260
85	Anura	Ranidae	*Rana*	*temporaria*	AY326063	DQ347231
86	Anura	Ranidae	*Rana*	*aurora*	DQ283189	DQ019509
87	Anura	Ranidae	*Rana*	*clamitans*	DQ283185	DQ347262
88	Anura	Ranidae	*Rana*	*palmipes*	DQ283384	DQ347263
89	Anura	Ranidae	*Rana*	*pipiens*	Y10945	DQ347265
76	Anura	Rhacophoridae	*Buergeria*	*buergeri*	AB127977	DQ347226
77	Anura	Rhacophoridae	*Chirixalus*	*doriae*	DQ283135	DQ347261
12	Anura	Rhinophrynidae	*Rhinophrynus*	*dorsalis*	DQ283109	AY874302
23	Anura	Scaphiopodidae	*Scaphiopus*	*hurteri*	AY581619	AY874301
24	Anura	Scaphiopodidae	*Scaphiopus*	*couchii*	DQ283150	AY323759
9	Caudata	Ambystomatidae	*Ambystoma*	*mexicanum*	NC005797	AY323752
7	Caudata	Hynobiidae	*Hynobius*	*formosanus*	NC008084	DQ347285
8	Caudata	Plethodontidae	*Eurycea*	*bislineata*	AY728217	AY691706
5	Gymnophiona	Caeciliidae	*Gegeneophis*	*ramaswamii*	NC006301	AY456255
6	Gymnophiona	Rhinatrematidae	*Rhinatrema*	*bivittatum*	NC006303	AY456257
4	Gymnophiona	Typhlonectidae	*Typhlonectes*	*natans*	NC002471	AY456260
2	Galliformes	Phasianidae	*Gallus*	*gallus*	AP003319	AF143730
1	Lepidosireniformes	Lepidosirenidae	*Lepidosiren*	*paradoxa*	NC003342	AY442926
3	Primates	Hominidae	*Homo*	*sapiens*	AC000021	NM000448

* Please, request this sequence to David Cannatella <catfish@mail.utexas.edu>
