# Supplementary material for: Amazonian Amphibian Diversity Is Primarily Derived from Late Miocene Andean Lineages
Source: PLoS Biol. 2009 Mar 10;7(3):e1000056. doi: 10.1371/journal.pbio.1000056 (PMC2653552; doi:10.1371/journal.pbio.1000056)
Supplement: Supplementary file 5 [file pbio.1000056.st003.pdf]

Table S3. Taxa used to infer the phylogeny and the chronogram of the poison frogs. 'Use' identifies which samples were used for the phylogeny (P), chronogram (C), and Langrage (L) analyses.

12S	16S beginning	16S end	12S-16S	Genera	Species	Use	Museum	Country	Code	Locality	Altitude (m)	Area	Code	Long	Lat
			AY326015	*Telmatobius*	*niger*	P, C	TNHC62493	Ecuador	EC	Azuay: 48.8 km WNW Cuenca	3380	Outgroup	A	-78.727	-2.774
			AY326014	*Ceratophrys*	*cornuta*	P, C	KU202561	Peru	PE	Madre de Dios: Cusco Amazonico	200	Outgroup	A	-70.08	-12.60
			AY843579	*Crossodactylus*	*schmidti*	P, C	MLPA 1414	Argentina	AR	Misiones:Aristobulo del Valle, Balneario Cunapiru	450	Outgroup	A	-54.9167	-27.116
			DQ283096	*Hylodes*	*phyllodes*	P, C	CFBH-T 249	Brazil	BR	Sao Paulo: Picinguaba, Ubatuba	50	Outgroup	A	-45.0333	-23.45
			DQ283072	*Megaelosia*	*goeldii*	P, C	MZUSP95879	Brazil	BR	Rio de Janeiro: Teresopolis, Rio Beija-Flor	910	Outgroup	A	-43.150	-22.399
			AY364540	*Centrolene*	*grandisonae*	P, C	QCAZ16512	Ecuador	EC	Pichincha: Manuel Cornejo A (Tandapi), vía Atenas	950	Outgroup	A	-78.783	-0.4167
			DQ283331	*Thoropa*	*militaris*	P, C	CFBH 3239	Brazil	BR	Sao Paulo: Picinguaba, Ubatuba	50	Outgroup	A	-45.0333	-23.45
			EU342502	*Vanzolinius*	*discodactylus*	P, C	QCAZ16625	Ecuador	EC	Francisco de Orellana: Parque Nacional Yasuni-Estacion PUCE	230	Outgroup	A	-76.50	-0.633
			DQ337246	*Engystomops*	*pustulosus*	P, C	LW1067	El Salvador	EL	El Salvador: San Miguel	140	Outgroup	A	-88.2833	13.5333
			AY325999	*Melanophryniscus*	*stelzneri*	P, C	AG87	n.d.	n.d.	n.d.	n.d.	Outgroup	A	n.d.	n.d.
			AY325996	*Atelopus*	*varius*	P, C	MVZ 223279	Costa Rica	CR	Puntarenas: 29 km NE San Vito, Las Alturas	1500	Outgroup	A	-82.9667	8.8333
			AY364538	*Adenomera*	*sp.*	P, C	QCAZ15998	Ecuador	EC	Zamora Chinchipe: near Zamora	980	Outgroup	A	-78.956	-4.068
			DQ502126	*Colostethus*	*alagoanus*	P, C	MRT 6031	Brazil	BR	Bahia: Sao Jose da Vitoria, Fazenda Unacau	630	Brazilian Shield	K	-39.3	-15.15
			DQ502242	*Nephelobates*	*sp.*	P, C, L	WES 626	Venezuela	VE	Trujillo: carretera Humocaro Bajo-Agua de Obispos	2400	Venezuela	D	-70.43	9.09
			DQ502274	*Colostethus*	*saltuensis*	P, C, L	MUJ 3726	Colombia	CO	Boyaca: Cubara, Fatima, Quebrada Gralanday	1560	North Oriental Andes	E	-72.1083	7.0019
AY263216	AY263263	AJ430678		*Nephelobates*	*molinarii*	P, L	ULABG4497-4502	Venezuela	VE	Merida: near Bailadores	1920	Venezuela	D	-71.1408	8.2208
			DQ502154	*Aromobates*	*nocturnus*	P, C, L	AMNH A130041	Venezuela	VE	Trujillo: 2 km (airline) ESE Agua de Obispos	2250	Venezuela	D	-70.1072222	9.7038889
AY263219	AY263269	AJ430676		*Mannophryne*	*herminae*	P, L	ULABG4506-4508	Venezuela	VE	Carabobo: La Entrada (near Valencia)	782	Venezuela	D	-68.04472	10.3042
		AJ430675		*Mannophryne*	*collaris*	P, L	ULABG4248	Venezuela	VE	Merida: Ejido	1096	Venezuela	D	-71.2375	8.5514
			DQ502160	*Mannophryne*	*herminae*	P, C, L	none	Venezuela	VE	Trujillo: 2 km (airline) W La Pena	1920	Venezuela	D	-70.1622	9.7172
AY191230		AY206717		*Mannophryne*	*olmonae*	P, L	none	Trinidad & Tobago	TT	Tobago: North Tobago	200	Venezuela	D	-60.68	11.28

Table S3 (Cont) Taxa used to infer the phylogeny and the chronogram of the poison frogs. 'Use' identifies which samples were used for the phylogeny (P), chronogram (C), and Langrage (L) analyses

12S	16S beginning	16S end	12S-16S	Genera	Species	Use	Museum	Country	Code	Locality	altitude (m)	Area	Code	Long	Lat
			EU342503	*Mannophryne*	*riveroi*	P, C, L	MHNLS16433	Venezuela	VE	Sucre: Paria Peninsula, base Cerro Humo	950	Venezuela	D	-62.6	10.683
			DQ502244	*Mannophryne*	*sp.*	P, C, L	WES1035	Venezuela	VE	Monagas: Cueva del Guacharo, near Caripe	1400	Venezuela	D	-63.49	10.17
			DQ084067	*Mannophryne*	*venezuelensis*	P, L	ULABG6706	Venezuela	VE	Sucre: San Juan de Las Galdonas-San Juan de Unare Road	180	Venezuela	D	-62.85	10.69
			EU342504	*Mannophryne*	*trinitatis*	P, C, L	MVZ199837	Trinidad & Tobago	TT	Trinidad: Caroni, Charuma Ward, Tamana Cave	64	Venezuela	D	-61.2	10.3833
			EU342505	*Colostethus*	*palmatus*	P, C, L	TNHCFS4955	Colombia	CO	Boyaca: Villa de Leiva	2118	North Oriental Andes	E	-73.5283	5.6386
			EU342506	*Colostethus*	*palmatus*	P, C, L	TNHCFS4983	Colombia	CO	Cundinamarca: San Francisco, Finca La Galia	1760	North Oriental Andes	E	-74.283	4.951
		AY263228		*Colostethus*	*palmatus*	P, L	none	Colombia	CO	Cundinamarca: Las Brisas	2005	North Oriental Andes	E	- 73.918	4.433
			EU342507	*Colostethus*	*palmatus*	P, L	TNHCFS4999	Colombia	CO	Cundinamarca: Las Brisas (Caqueza)	2005	North Oriental Andes	E	- 73.918	4.433
			EU342508	*Colostethus*	*palmatus*	P, L	TNHCFS4949	Colombia	CO	Meta: Guyabetal-Villavicencio road	1280	North Oriental Andes	E	-73.779	4.199
			DQ502128	*Colostethus*	*tepuyensis*	P, C, L	ROM 39637	Guyana	GU	Mazaruni-Potoro: Mt. Ayanganna, northeast plateau	1490	Guiana Shield	B	-59.95	5.4
			DQ502253	*Colostethus*	*sp. "Thomasing"*	P, C, L	UTA A56709	Guyana	GU	Mazaruni-Potaro: Mt. Thomasing (~2 km N Imbaimadai)	508	Guiana Shield	B	-60.29758	5.7396
			DQ502127	*Colostethus*	*beebei*	P, C, L	ROM 39631	Guyana	GU	Mazaruni-Potoro: Mt. Ayanganna, northeast plateau	1520	Guiana Shield	B	-59.95	5.4
			DQ502258	*Colostethus*	*roraima*	P, C, L	CPI 10216	Guyana	GU	Mazaruni-Potoro: Mt. Roraima	2150	Guiana Shield	B	-60.7333	5.2
			DQ502257	*Colostethus*	*degranvillei*	P, C, L	CPI 10209	Guyana	GU	Mazaruni-Potoro: Mt. Roraima	1075	Guiana Shield	B	-60.7333	5.2
			DQ502019	*Colostethus*	*degranvillei*	P, C, L	none	Guyana	GU	Mazaruni-Potoro: Mereme Mountains	n.d.	Guiana Shield	B	n.d.	n.d.
			DQ502255	*Colostethus*	*praderioi*	P, C, L	CPI 10198	Guyana	GU	Mazaruni-Potoro: Mt. Roraima	1310	Guiana Shield	B	-60.7333	5.2
			DQ502129	*Colostethus*	*sp. "Ayanganna"*	P, C, L	ROM 39639	Guyana	GU	Mazaruni-Potoro: Mt. Ayanganna, northeast plateau	1520	Guiana Shield	B	-59.95	5.4
			DQ502107	*Colostethus*	*stepheni*	P, C, L	MJH 3928	Brazil	BR	Amazonas: 25 km NE Manaus, Reserva Forestal Adolpho Ducke	80	Amazon/Guiana Shield	BC	-59.95	-2.933
AY263213	AY263260	AY263234		*Colostethus*	*degranvillei*	P, L	MNHN 2000-655	French Guiana	FG	Departement de la Guyane: Saul	230	Guiana Shield	B	-53.2	3.6167
			DQ502247	*Colostethus*	*sp."Tafelberg"*	P, L	UTA A55758	Surinam	SU	Sipaliwini: ca. 4.0 km N of Tafelberg airstrip	300	Guiana Shield	B	-56.15	3.7833

Table S3 (Cont) Taxa used to infer the phylogeny and the chronogram of the poison frogs. 'Use' identifies which samples were used for the phylogeny (P), chronogram (C), and Langrage (L) analyses

12S	16S beginning	16S end	12S-16S	Genera	Species	Use	Museum	Country	Code	Locality	altitude (m)	Area	Code	Long	Lat
			DQ502249	*Colostethus*	*sp."Brownsberg"*	P, C, L	UTA A56469	Surinam	SU	Brokopondo: Brownsweg Nature Park	75	Guiana Shield	B	-55.15	5.0
			DQ501980	*Colostethus*	*baeobatrachus*	P, C, L	PK-437-1	French Guiana	FG	Departement de la Guyane:Pic Matecho lake	590	Guiana Shield	B	-53.033	3.750
		AY263231		*Colostethus*	*baeobatrachus*	P, L	MNHN 2000-654	French Guiana	FG	Departement de la Guyane: Saul	230	Guiana Shield	B	-53.2	3.6167
			DQ502074	*Colostethus*	*sp. "Neblina"*	P, C, L	AMCC 106112	Venezuela	VE	Amazonas: Rio Negro, Neblina Base Camp on Rio Mawarinuma	140	Guiana Shield	B	-66.167	0.833
			DQ283044	*Colostethus*	*undulatus*	P, C, L	AMNHA159139	Venezuela	VE	Amazonas: Cerro Yutaje	1700	Guiana Shield	B	-66.133	5.767
			DQ502272	*Colostethus*	*sp."Magdalena"*	P, C, L	MUJ 3520	Colombia	CO	Caldas: La Dorada, San Roque, Reserva Natural Privada Riomanso	280	Choco	I	-74.767	5.667
			EU342509	*Colostethus*	*talamancae*	P, C, L	TNHCFS4803	Panama	PA	Panama: Lago Bayano	60	Choco	I	-78.5833	9.1333
			EU342510	*Colostethus*	*talamancae*	P, C, L	TNHCFS4993	Colombia	CO	Choco: Quibdo, La Troje	50	Choco	I	-76.591	5.728
			EU342511	*Colostethus*	*talamancae*	P, L	QCAZ16551	Ecuador	EC	Pichincha: Río Sabalo, ca. Pedro Vicente Maldonado	191	Choco	I	-79.4167	0.0667
			AY364562	*Colostethus*	*talamancae*	P, C, L	QCAZ15562	Ecuador	EC	Esmeraldas: A 3 Km de Durango, en el cruce de la vía a San Lorenzo	253	Choco	I	-79.4167	0.0667
			EU342512	*Colostethus*	*talamancae*	P, C, L	LSUMZ15094	Nicaragua	NI	Rio San Juan: Isla de Diamante (ca. 15 km SE El Castillo on Rio San Juan)	40	Central America	J	-84.300	10.933
			EU342513	*Colostethus*	*talamancae*	P, L	LSUMZ15095	Nicaragua	NI	Rio San Juan: Isla de Diamante (ca. 15 km SE El Castillo on Rio San Juan)	40	Central America	J	-84.300	10.933
			EU342514	*Colostethus*	*talamancae*	P, C, L	KS16	Panama	PA	San Blas: Nusagandi	478	Choco	I	-78.987	9.2994
			EU342515	*Colostethus*	*talamancae*	P, C, L	TNHCFS4802	Panama	PA	Cocle: Cerro Gaital (CTF)	886	Central America	J	-80.11603	8.62873
			DQ502166	*Colostethus*	*talamancae*	P, L	SIUC 7667	Panama	PA	Cocle: El Cope, Parque Nacional General de Division 'Omar Torrijos Herrera	782	Central America	J	-80.592	8.667
			EU342516	*Colostethus*	*talamancae*	P, L	CH4998	Panama	PA	Panama: Rio Indio Arriba	385	Choco	I	-80.133	8.75
			EU342517	*Colostethus*	*talamancae*	P, C, L	TNHCFS4801	Panama	PA	Bocas del Toro: Isla Bastimientos, Red frog beach, 15 m (CTR)	15	Central America	J	-82.1797	9.3445
			AY843577	*Colostethus*	*talamancae*	P, C, L	USNM-FS 52055	Panama	PA	Bocas del Toro	n.d.	Central America	J	n.d.	n.d.

Table S3 (Cont) Taxa used to infer the phylogeny and the chronogram of the poison frogs. 'Use' identifies which samples were used for the phylogeny (P), chronogram (C), and Langrage (L) analyses

12S	16S beginning	16S end	12S-16S	Genera	Species	Use	Museum	Country	Code	Locality	altitude (m)	Area	Code	Long	Lat
			EU342518	*Colostethus*	*nidicola*	P, C, L	LSUMZ16941	Brazil	BR	Amazonas: Castanho, ca. 40 km S Manaus	40	Amazon	C	-60.455	-3.6196
			EU342519	*Colostethus*	*nidicola*	P, L	LSUMZ16956	Brazil	BR	Amazonas: Castanho, ca. 40 km S Manaus	40	Amazon	C	-60.455	-3.6196
			EU342520	*Colostethus*	*spLarge*	P, C, L	LSUMZ17633	Brazil	BR	Rondonia: Rio Formoso, Parque Estadual Guajara-Mirim, 90 km N Nova Mamore	180	Amazon	C	-64.5633	-10.3214
			EU342521	*Colostethus*	*spLarge*	P, L	LSUMZ17424	Brazil	BR	Rondonia: Rio Formoso, Parque Estadual Guajara-Mirim, 90 km N Nova Mamore	180	Amazon	C	-64.5633	-10.321
			DQ502189	*Colostethus*	*brunneus*	P, C, L	MPEG 11921	Brazil	BR	Para: 101 km S, 15 km E Santarem CEMEX (near Rio Curua-Una)	120	Amazon	C	-54.833	-3.15
			EU342522	*Colostethus*	*brunneus*	P, L	LSUMZ15208	Brazil	BR	Para: 101 km S, 15 km E Santarem CEMEX (near Rio Curua-Una)	120	Amazon	C	-54.833	-3.15
			EU342523	*Colostethus*	*brunneus*	P, L	LSUMZ15187	Brazil	BR	Para: 101 km S, 15 km E Santarem CEMEX (near Rio Curua-Una)	120	Amazon	C	-54.833	-3.15
			EU342524	*Colostethus*	*peruvianus*	P, C, L	MHNSM22923	Peru	PE	San Martin: road to San Jose de Sisa	509	Central Oriental Andes	G	-76.37298	-6.38022
			EU342525	*Colostethus*	*sp*	P, C, L	QCAZ16495	Ecuador	EC	Morona Santiago: conjuction of Rio Paute-Negro	540	Central Oriental Andes	G	-78.3034	-2.7454
			AY364549	*Colostethus*	*kingsburyi*	P, C, L	QCAZ16523	Ecuador	EC	Zamora Chinchipe: Rio Chicana	1085	Central Oriental Andes	G	-78.7433	-3.7242
			EU342526	*Colostethus*	*kingsburyi*	P, L	QCAZ16525	Ecuador	EC	Zamora Chinchipe: near Panguitza	870	Central Oriental Andes	G	-78.8125	-3.8986
			EU342527	*Colostethus*	*kingsburyi*	P, L	QCAZ16524	Ecuador	EC	Zamora Chinchipe: near Panguitza	870	Central Oriental Andes	G	-78.8125	-3.8986
			EU342528	*Colostethus*	*kingsburyi*	P, L	QCAZ16614	Ecuador	EC	Zamora Chinchipe: near Arenas	1124	Central Oriental Andes	G	-78.82	-3.73
			EU342529	*Colostethus*	*kingsburyi*	P, L	QCAZ16612	Ecuador	EC	Morona Santiago: 10 km S Gualaquiza	1250	Central Oriental Andes	G	-78.55	-3.40
			AY364550	*Colostethus*	*kingsburyi*	P, L	QCAZ16613	Ecuador	EC	Zamora Chinchipe: near Tundayme, Condor Cordillera	1010	Central Oriental Andes	G	-78.4903	-3.5764
			EU342530	*Allobates*	*zaparo*	P, C, L	QCAZ25212	Ecuador	EC	Pastaza: Kapawi Lodge	239	Amazon	C	-76.830812	-2.540804

Table S3 (Cont) Taxa used to infer the phylogeny and the chronogram of the poison frogs. 'Use' identifies which samples were used for the phylogeny (P), chronogram (C), and Langrage (L) analyses

12S	16S beginning	16S end	12S-16S	Genera	Species	Use	Museum	Country	Code	Locality	altitude (m)	Area	Code	Long	Lat
			AY364579	*Allobates*	*zaparo*	P, C, L	QCAZ16604	Ecuador	EC	Napo: Jatun Sacha, vía Ahuano	390	Central Oriental Andes	G	-77.5167	-1.0667
			EU342531	*Allobates*	*zaparo*	P, C, L	QCAZ16602	Ecuador	EC	Morona Santiago: Río Yaupi, Jemkepeket	473	Central Oriental Andes	G	-77.8644	-2.9750
			AY364578	*Allobates*	*zaparo*	P, L	QCAZ16601	Ecuador	EC	Morona Santiago: 2 km E Santiago	495	Central Oriental Andes	G	-78.0408	-3.0383
			AY326026	*Allobates*	*femoralis*	P, C, L	KU 205291	Peru	PE	Madre de Dios: Cuzco Amazonico, 15 km E Puerto Maldonado	200	Amazon	C	-70.08	-12.60
			AY326027	*Allobates*	*femoralis*	P, L	KU 205292	Peru	PE	Madre de Dios: Cuzco Amazonico, 15 km E Puerto Maldonado	200	Amazon	C	-70.08	-12.60
			EU342532	*Allobates*	*femoralis*	P, C, L	LSUMZ13667	Brazil	BR	Acre: near 5 km N Porto Walker, inland from the Rio Jurua	200	Amazon	C	-72.777	-8.259
			EU342533	*Allobates*	*femoralis*	P, L	LSUMZ13633	Brazil	BR	Acre: near 5 km N Porto Walker, inland from the Rio Jurua	200	Amazon	C	-72.777	-8.259
			EU342534	*Allobates*	*femoralis*	P, C, L	QCAZ19167	Ecuador	EC	Sucumbios: Laguna Grande, Reserva de Producción Faunística Cuyabeno	260	Amazon	C	-76.216667	0
			EU342535	*Allobates*	*femoralis*	P, C, L	QCAZ16485	Ecuador	EC	Francisco de Orellana: Parque Nacional Yasuni-Estacion PUCE	230	Amazon	C	-76.50	-0.633
			AY364543	*Allobates*	*femoralis*	P, L	QCAZ16484	Ecuador	EC	Francisco de Orellana: Parque Nacional Yasuni-Estacion PUCE	230	Amazon	C	-76.50	-0.633
			DQ502113	*Allobates*	*femoralis*	P, C, L	MJH 3976	Brazil	BR	Amazonas: 25 km NE Manaus, Reserva Forestal Adolpho Ducke	80	Amazon	C	-59.95	-2.933
			DQ502117	*Allobates*	*femoralis*	P, C, L	MJH 7354	Peru	PE	Huanuco: Rio Llullapichis, Panguana	270	Amazon	C	-74.93	-9.62
			EU342536	*Allobates*	*femoralis*	P, C, L	TNHCFS4963	Colombia	CO	Amazonas: Leticia, Cerca Viva	83	Amazon	C	-69.9406	-4.2153
			EU342537	*Allobates*	*femoralis*	P, C, L	LSUMZ17436	Brazil	BR	Rondonia: Rio Formoso, Parque Estadual Guajara-Mirim, 90 km N Nova Mamore	180	Amazon	C	-64.563	-10.321
			DQ502246	*Allobates*	*femoralis*	P, C, L	UTA A56478	Surinam	SU	Sipaliwini: in the vicinity of Kayser airstrip	260	Guiana Shield	B	-56.467	3.10

Table S3 (Cont) Taxa used to infer the phylogeny and the chronogram of the poison frogs. 'Use' identifies which samples were used for the phylogeny (P), chronogram (C), and Langrage (L) analyses

12S	16S beginning	16S end	12S-16S	Genera	Species	Use	Museum	Country	Code	Locality	altitude (m)	Area	Code	Long	Lat
			EU342538	*Allobates*	*femoralis*	P, L	LSUMZ15222	Brazil	BR	Para: 101 km S, 15 km E Santarem CEMEX (near Rio Curua-Una)	120	Amazon	C	-54.833	-3.15
			EU342539	*Allobates*	*femoralis*	P, C, L	LSUMZ15213	Brazil	BR	Para: 101 km S, 15 km E Santarem CEMEX (near Rio Curua-Una)	120	Amazon	C	-54.833	-3.15
			DQ502271	*Colostethus*	*juanii*	P, L	ARA 2394	Colombia	CO	Meta: Villavicencio, Pozo Azul	560	North Oriental Andes	E	-73.635	4.1533
			EU342540	*Colostethus*	*juanii*	P, C, L	TNHCFS4944	Colombia	CO	Meta: Villavicencio, Villavicencio-Restrepo road	411	North Oriental Andes	E	-73.603	4.1903
			EU342541	*Colostethus*	*caeruleodactylus*	P, C, L	LSUMZ16926	Brazil	BR	Amazonas: Castanho, ca. 40 km S Manaus, at km 12 on road to Autazes	40	Amazon	C	-60.455	-3.6195
			EU342542	*Colostethus*	*caeruleodactylus*	P, L	LSUMZ16929	Brazil	BR	Amazonas: Castanho, ca. 40 km S Manaus, at km 12 on road to Autazes	40	Amazon	C	-60.455	-3.6195
			DQ502202	*Colostethus*	*gasconi*	P, L	OMNH 36637	Brazil	BR	Amazonas: Rio Ituxi (Scheffer-Madeireira)	110	Amazon	C	-65.717	-8.4794
			EU342543	*Colostethus*	*marchesianus*	P, L	LSUMZ15393	Brazil	BR	Amazonas: Rio Ituxi at the Madeireira Scheffer; left bank	110	Amazon	C	-65.7166	-8.4794
			EU342544	*Colostethus*	*marchesianus*	P, C, L	LSUMZ15392	Brazil	BR	Amazonas: Rio Ituxi at the Madeireira Scheffer; left bank	110	Amazon	C	-65.7166	-8.4794
			EU342545	*Colostethus*	*marchesianus*	P, L	LSUMZ15296	Brazil	BR	Para: 101 km S, 15 km E Santarem CEMEX (near Rio Curua-Una)	120	Amazon	C	-54.833	-3.15
			EU342546	*Colostethus*	*marchesianus*	P, C, L	LSUMZ15291	Brazil	BR	Para: 101 km S, 15 km E Santarem CEMEX (near Rio Curua-Una)	120	Amazon	C	-54.833	-3.15
		AY263230		*Colostethus*	*aff marchesianus*	P, L	MNHN 2000-651	French Guiana	FG	Departement de la Guyane: Saul	230	Guiana Shield	B	-53.2	3.6167
		AY263232		*Colostethus*	*aff marchesianus*	P, L	MNHN 2000-653	French Guiana	FG	Departement de la Guyane: Saul	230	Guiana Shield	B	-53.2	3.6167
			DQ502139	*Colostethus*	*sp. "PEG-M3"*	P, C, L	MPEG 13386	Brazil	BR	Rondonia: Rio Formoso, Parque Estadual Guajara-Mirim, 90 km N Nova Mamore	180	Amazon	C	-64.563	-10.321
			EU342547	*Colostethus*	*spOther*	P, C, L	LSUMZ17554	Brazil	BR	Rondonia: Rio Formoso, Parque Estadual Guajara-Mirim, 90 km N Nova Mamore	180	Amazon	C	-64.563	-10.321

Table S3 (Cont) Taxa used to infer the phylogeny and the chronogram of the poison frogs. 'Use' identifies which samples were used for the phylogeny (P), chronogram (C), and Langrage (L) analyses

12S	16S beginning	16S end	12S-16S	Genera	Species	Use	Museum	Country	Code	Locality	altitude (m)	Area	Code	Long	Lat
			EU342548	*Colostethus*	*spOther*	P, L	LSUMZ17629	Brazil	BR	Rondonia: Rio Formoso, Parque Estadual Guajara-Mirim, 90 km N Nova Mamore	180	Amazon	C	-64.563	-10.321
			EU342549	*Colostethus*	*ornatus*	P, C, L	MHNSM22646	Peru	PE	San Martin: 17km W from Tarapoto to Moyobamba	253	Amazon	C	-76.5125	-6.47889
			EU342550	*Colostethus*	*ornatus*	P, L	MHNSM22863	Peru	PE	San Martin: 17km W from Tarapoto to Moyobamba	253	Amazon	C	-76.5125	-6.47889
			EU342551	*Colostethus*	*spConspicuous*	P, C, L	LSUMZ13743	Brazil	BR	Acre: near 5 km N Porto Walker, inland from the Rio Jurua	210	Amazon	C	-72.777	-8.259
			EU342552	*Colostethus*	*spConspicuous*	P, L	LSUMZ13785	Brazil	BR	Acre: near 5 km N Porto Walker, inland from the Rio Jurua	210	Amazon	C	-72.777	-8.259
			AY364557	*Colostethus*	*insperatus*	P, C, L	QCAZ16533	Ecuador	EC	Francisco de Orellana: Parque Nacional Yasuni-Estacion PUCE	230	Amazon	C	-76.50	-0.633
			EU342553	*Colostethus*	*sp.*	P, C, L	TNHCFS4997	Colombia	CO	Amazonas: Leticia, Cerca Viva	83	Amazon	C	-69.9406	-4.2153
			EU342554	*Colostethus*	*sp*	P, C, L	QCAZ20140	Ecuador	EC	Sucumbios: Laguna Grande-Neotropic, Reserva de Producción Faunística Cuyabeno	260	Amazon	C	-76.217	0
			EU342555	*Colostethus*	*sp*	P, C, L	QCAZ10231	Ecuador	EC	Sucumbios: Laguna Grande-Neotropic, Reserva de Producción Faunística Cuyabeno	260	Amazon	C	-76.217	0
			EU342556	*Colostethus*	*sp*	P, C, L	QCAZ25480	Ecuador	EC	Pastaza: Kapawi Lodge	239	Amazon	C	-76.831	-2.541
			EU342557	*Colostethus*	*sp*	P, L	QCAZ16616	Ecuador	EC	Napo: near Comunidad Cando	760	North Oriental Andes	E	-77.944	-1.072
			AY364561	*Colostethus*	*spG3*	P, L	QCAZ16609	Ecuador	EC	Pastaza: Puyo-Macas road 43 km	850	North Oriental Andes	E	-77.841	-1.6611
			EU342558	*Colostethus*	*sp*	P, L	QCAZ16496	Ecuador	EC	Morona Santiago: conjuction of Rio Paute and Rio Negro	540	North Oriental Andes	E	-78.3034	-2.7454
			EU342559	*Colostethus*	*sp*	P, C, L	QCAZ16615	Ecuador	EC	Pastaza: Fatima Zoo	1050	North Oriental Andes	E	-77.9833	-1.4333
		AY263233		*Colostethus*	*aff marchesianus*	P, L	none	French Guiana	FG	Departement de la Guyane: Saul	230	Guiana Shield	B	-53.2	3.6167

Table S3 (Cont) Taxa used to infer the phylogeny and the chronogram of the poison frogs. 'Use' identifies which samples were used for the phylogeny (P), chronogram (C), and Langrage (L) analyses

12S	16S beginning	16S end	12S-16S	Genera	Species	Use	Museum	Country	Code	Locality	altitude (m)	Area	Code	Long	Lat
			EU342563	*Colostethus*	*spSmall*	P, C, L	LSUMZ17421	Brazil	BR	Rondonia: Rio Formoso, Parque Estadual Guajara-Mirim, 90 km N Nova Mamore	180	Amazon	C	-64.563	-10.321
			EU342564	*Colostethus*	*spSmall*	P, L	LSUMZ17419	Brazil	BR	Rondonia: Rio Formoso, Parque Estadual Guajara-Mirim, 90 km N Nova Mamore	180	Amazon	C	-64.563	-10.321
			EU342565	*Colostethus*	*gasconi*	P, C, L	LSUMZ15435	Brazil	BR	Amazonas: Rio Ituxi at the Madeireira Scheffer; left bank	110	Amazon	C	-65.717	-8.479
			EU342566	*Colostethus*	*sp1*	P, C, L	LSUMZ16911	Brazil	BR	Amazonas: Castanho, ca. 40 km S Manaus at km 12 on road to Autazes	40	Amazon	C	-60.455	-3.620
			EU342567	*Colostethus*	*sp1*	P, L	LSUMZ16910	Brazil	BR	Amazonas: Castanho, ca. 40 km S Manaus at km 12 on road to Autazes	40	Amazon	C	-60.455	-3.620
		AJ430673		*Colostethus*	*humilis*	P, L	ULABG4477	Venezuela	VE	Monagas: Bocono, North Orinoco Riverline	79	Venezuela	D	-63.3	9.7833
			DQ502109	*Colostethus*	*sp. "SaoFrancisco"*	P, C, L	MJH 3909	Brazil	BR	Amazonas: Facenda Sao Francisco, 2 km N km 49 on Manaus-Manacapuru road	80	Amazon	C	-60.0002	-2.8207
			DQ502115	*Colostethus*	*sp. "ReservaDucke"*	P, C, L	MJH 3988	Brazil	BR	Amazonas: 25 km NE Manaus, Reserva Forestal Adolpho Ducke	80	Amazon	C	-59.95	-2.933
			DQ502118	*Colostethus*	*trilineatus*	P, C, L	MJH 7477	Peru	PE	Huanuco: Rio Llullapichis, Panguana	270	Amazon	C	-74.93	-9.62
			EU342560	*Colostethus*	*trilineatus*	P, C, L	KU215174	Peru	PE	Madre de Dios: Cuzco Amazonico, 15 km E Puerto Maldonado	200	Amazon	C	-70.08	-12.60
			EU342561	*Colostethus*	*gasconi*	P, C, L	LSUMZ13691	Brazil	BR	Acre: near 5 km N Porto Walker, inland from the Rio Jurua	210	Amazon	C	-72.776972	-8.258667
			DQ502190	*Colostethus*	*sp. "PortoWalter2"*	P, L	MPEG 12360	Brazil	BR	Acre: near 5 km N Porto Walker, inland from the Rio Jurua	210	Amazon	C	-72.776972	-8.258667
			EU342562	*Colostethus*	*gasconi*	P, L	LSUMZ13690	Brazil	BR	Acre: near 5 km N Porto Walker, inland from the Rio Jurua	210	Amazon	C	-72.776972	-8.258667
			EU342568	*Epipedobates*	*boulengeri*	P, C, L	TNHCFS4941	Colombia	CO	Cauca: Isla Gorgona	12	Choco	I	-78.2	3

Table S3 (Cont) Taxa used to infer the phylogeny and the chronogram of the poison frogs. 'Use' identifies which samples were used for the phylogeny (P), chronogram (C), and Langrage (L) analyses

12S	16S beginning	16S end	12S-16S	Genera	Species	Use	Museum	Country	Code	Locality	altitude (m)	Area	Code	Long	Lat
			EU342569	*Epipedobates*	*boulengeri*	P, C, L	QCAZ16574	Ecuador	EC	Esmeraldas: A 3 Km de Durango, road to San Lorenzo	253	Choco	I	-78.70	0.867
			EU342570	*Epipedobates*	*boulengeri*	P, L	QCAZ13364	Ecuador	EC	Carchi: 2 km S El Chical, Rio Blanco	1298	North Occidental Andes	F	-78.2333	1.0333
			EU342571	*Epipedobates*	*espinosai*	P, C, L	QCAZ27224	Ecuador	EC	Los Rios: Reserva Rio Palenque, 2 km SSE Patricia Pilar	189	Choco	I	-79.350	-0.5833
			EU342572	*Epipedobates*	*spF*	P, L	QCAZ16590	Ecuador	EC	Pichincha: Union del Toachi	694	North Occidental Andes	F	-78.95	-0.316
			AY364575	*Epipedobates*	*spF*	P, L	QCAZ16589	Ecuador	EC	Pihincha: Mindo	1525	North Occidental Andes	F	-78.80	-0.0333
			EU342573	*Epipedobates*	*anthonyi*	P, C, L	KU219761	Peru	PE	Piura: 8.5 km W Canchaque	1120	Central Occidental Andes	H	-79.6003	-5.3731
			AY364577	*Epipedobates*	*anthonyi*	P, L	QCAZ16596	Ecuador	EC	Loja: Macará-Catacocha	1135	Central Occidental Andes	H	-79.816667	-4.05
			EU342574	*Epipedobates*	*anthonyi*	P, L	QCAZ16594	Ecuador	EC	Azuay: Pasaje-Girón	1512	Central Occidental Andes	H	-79.35	-3.30
			EU342575	*Colostethus*	*machalilla*	P, C, L	QCAZ16529	Ecuador	EC	Cañar: Manta Real	465	Choco	I	-79.366667	-2.55
			EU342576	*Colostethus*	*machalilla*	P, L	QCAZ16528	Ecuador	EC	Cañar: Manta Real	465	Choco	I	-79.366667	-2.55
			AY364551	*Colostethus*	*machalilla*	P, L	QCAZ16526	Ecuador	EC	Manabi: Rio Ayampe	70	Choco	I	-80.783333	-1.666667
			EU342577	*Epipedobates*	*tricolor*	P, C, L	QCAZ21977	Ecuador	EC	Cotopaxi: Corazón-Moraspungo	1250	North Occidental Andes	F	-79.12	-1.18
			EU342578	*Epipedobates*	*tricolor*	P, L	QCAZ21388	Ecuador	EC	Bolívar: 13.4 km E Echeandía	879	North Occidental Andes	F	-79.266	-1.433
			EU342579	*Colostethus*	*aff nubicola*	P, C, L	TNHCFS4942	Colombia	CO	Choco: Quibdo, La Troje	50	Choco	I	-76.591	5.728
			EU342580	*Colostethus*	*nubicola*	P, C, L	CH5599	Panama	PA	Darien: Sendero Boca de Cupe	71	Choco	I	-77.5833	8.05
			DQ502161	*Colostethus*	*nubicola*	P, C, L	SIUC 7652	Panama	PA	Cocle: El Cope, Parque Nacional General de Division 'Omar Torrijos Herrera	782	Central America	J	-80.592	8.667
			EU342581	*Colostethus*	*nubicola*	P, C, L	CH5316	Panama	PA	Cocle: Quebrada Guabito, Palmarazo	138	Central America	J	-80.6667	8.7333
			EU342582	*Colostethus*	*nubicola*	P, L	CH5099	Panama	PA	Cocle: Escaliche, Penome, Quebrada Escaliche	70	Central America	J	-80.366	8.516
			DQ502103	*Colostethus*	*nubicola "spC"*	P, C, L	MHNUC 320	Colombia	CO	Choco: Bahia Solano, Quebrada Tebada	165	Choco	I	-77.35	6.483

Table S3 (Cont) Taxa used to infer the phylogeny and the chronogram of the poison frogs. 'Use' identifies which samples were used for the phylogeny (P), chronogram (C), and Langrage (L) analyses

12S	16S beginning	16S end	12S-16S	Genera	Species	Use	Museum	Country	Code	Locality	altitude (m)	Area	Code	Long	Lat
			EU342583	*Colostethus*	*flotator*	P, C, L	KS35	Costa Rica	CR	Puntarenas: Llorona, Peninsula Osa	30	Central America	J	-83.732	8.597
			EU342584	*Colostethus*	*flotator*	P, C, L	TNHCFS4804	Panama	PA	Cocle: El Cope, Parque Nacional General de Division 'Omar Torrijos Herrera	782	Central America	J	-80.592	8.667
			EU342585	*Colostethus*	*flotator*	P, L	CH5100	Panama	PA	Cocle: Rio San Miguel	750	Central America	J	-80.3167	8.7833
			DQ502164	*Colostethus*	*flotator*	P, L	SIUC 7664	Panama	PA	Cocle: El Cope, Parque Nacional General de Division 'Omar Torrijos Herrera	782	Central America	J	-80.592	8.667
			EU342586	*Colostethus*	*flotator*	P, L	CH5173	Panama	PA	Cocle: Rio Toabre, La Sargenta	860	Central America	J	-80.55	8.9333
			EU342587	*Colostethus*	*flotator*	P, C, L	CH4828	Panama	PA	Panama: Parque Nacional Soberania, Pipeline road	25	Choco	I	-79.72997	9.14778
			EU342588	*Colostethus*	*flotator*	P, C, L	TNHCFS4806	Panama	PA	San Blas: Nusagandi	478	Choco	I	-78.98692	9.2994
			EU342589	*Colostethus*	*flotator*	P, L	CH5011	Panama	PA	Cocle: Quebrada Amarilla, Rio Indio Arriba	700	Central America	J	-80.1333	8.7667
			EU342590	*Colostethus*	*flotator*	P, C, L	TNHCFS4805	Panama	PA	Cocle: Cerro Gaital	886	Central America	J	-80.11603	8.62873
			DQ502163	*Colostethus*	*pratti*	P, C, L	SIUC 7658	Panama	PA	Cocle: El Cope, Parque Nacional General de Division 'Omar Torrijos Herrera	782	Central America	J	-80.592	8.667
			EU342591	*Colostethus*	*sp.*	P, C, L	TNHCFS4809	Panama	PA	Bocas del Toro: near Cilico Creek	39	Central America	J	-82.2833	9.0667
			EU342592	*Colostethus*	*latinasus*	P, C, L	CH5601	Panama	PA	Darien: Cana	450	Choco	I	-77.7167	7.9333
			EU342593	*Colostethus*	*latinasus*	P, L	CH5598	Panama	PA	Darien: La Mina de Cana	450	Choco	I	-77.7167	7.9333
			EU342594	*Colostethus*	*cf. pratti*	P, C, L	TNHCFS4808	Panama	PA	Panama: Parque Nacional Soberania, Pipeline road (CTA)	25	Choco	I	-79.72997	9.14778
			EU342595	*Colostethus*	*pratti*	P, L	KS3	Panama	PA	San Blas: Nusagandi	478	Choco	I	-78.98692	9.2994
			EU342596	*Colostethus*	*cf. pratti*	P, L	TNHCFS4807	Panama	PA	Colon: Parque Nacional Portobello (CTC)	50	Choco	I	-79.655553	9.52823
			DQ502265	*Colostethus*	*inguinalis*	P, C, L	MUJ 3247	Colombia	CO	Caldas: La Dorada, San Roque, Reserva Natural Privada Riomanso	280	Choco	I	-74.767	5.667
			DQ502178	*Colostethus*	*imbricolus*	P, C, L	MHNUC 257	Colombia	CO	Choco: Bahia Solano, Quebrada Nabuga	15	Choco	I	-77.333	6.366

Table S3 (Cont) Taxa used to infer the phylogeny and the chronogram of the poison frogs. 'Use' identifies which samples were used for the phylogeny (P), chronogram (C), and Langrage (L) analyses

12S	16S beginning	16S end	12S-16S	Genera	Species	Use	Museum	Country	Code	Locality	altitude (m)	Area	Code	Long	Lat
			DQ502172	*Colostethus*	*panamensis*	P, L	CH 5546	Panama	PA	Darien: headwaters of Rio Cana, Sierra del Pirre	450	Choco	I	-77.7167	7.9333
			EU342597	*Colostethus*	*inguinalis*	P, C, L	CH5546	Panama	PA	Darien: headwaters of Rio Cana, Sierra del Pirre	450	Choco	I	-77.7167	7.9333
			EU342598	*Colostethus*	*inguinalis*	P, C, L	KS18	Panama	PA	San Blas: Nusagandi	478	Choco	I	-78.98692	9.2994
			EU342599	*Colostethus*	*panamensis*	P, C, L	TNHCFS4810	Panama	PA	Colon: Fort Sherman (Ci4)	189	Central America	J	-79.91540	9.28016
			DQ502169	*Colostethus*	*panamensis*	P, C, L	SIUC 7666	Panama	PA	Cocle: El Cope, Parque Nacional General de Division 'Omar Torrijos Herrera	782	Central America	J	-80.592	8.667
			EU342600	*Colostethus*	*inguinalis*	P, L	CH5007	Panama	PA	Cocle: Quebrada Amarilla, Rio Indio	700	Central America	J	-80.1333	8.7667
			EU342601	*Colostethus*	*inguinalis*	P, C, L	CH5105	Panama	PA	Cocle: Quebrada Oscura, Rio San Miguel	750	Central America	J	-80.3167	8.7833
			EU342602	*Colostethus*	*fraterdanieli*	P, C, L	TNHCFS4976	Colombia	CO	Valle del Cauca: Buga, Buga-Buenaventura road	1610	North Occidental Andes	F	-76.435	3.876
			DQ502179	*Colostethus*	*fraterdanieli*	P, C, L	none	Colombia	CO	Caldas: Cordillera Central, W slope	2400	North Occidental Andes	F	n.d.	n.d.
			DQ502176	*Colostethus*	*fraterdanieli*	P, C, L	MHNUC 361	Colombia	CO	Cauca: Popayan, Hacienda La Paz	1700	North Occidental Andes	F	-76.6	2.483
			EU342603	*Colostethus*	*fraterdanieli*	P, C, L	TNHCFS4977	Colombia	CO	Quindio: Barbas, Finlandia, Hacienda Lusitania	1958	North Occidental Andes	F	-75.63525	4.689778
			EU342604	*Colostethus*	*fraterdanieli*	P, L	TNHCFS4967	Colombia	CO	Quindio: Quimbaya, Finca EL Cortijo	1085	North Occidental Andes	F	-75.84363	4.642972
			EU342605	*Colostethus*	*argyrogaster*	P, C, L	MHNSM22670	Peru	PE	San Martin: Tarapoto to Yurimaguas (34 km)	576	Central Oriental Andes	G	-76.2913	-6.42091
			EU342606	*Colostethus*	*argyrogaster*	P, L	MHNSM22877	Peru	PE	San Martin: Tarapoto to Yurimaguas (34 km)	576	Central Oriental Andes	G	-76.2913	-6.42091
			EU342607	*Colostethus*	*fugax*	P, C, L	QCAZ16608	Ecuador	EC	Pastaza: Canelos	631	Central Oriental Andes	G	-77.750	-1.5833
			AY364547	*Colostethus*	*fugax*	P, C, L	QCAZ16513	Ecuador	EC	Morona Santiago: 2 km E Santiago	495	Central Oriental Andes	G	-78.0408	-3.0383
			EU342608	*Colostethus*	*fugax*	P, L	QCAZ16515	Ecuador	EC	Morona Santiago: 2 km E Santiago	495	Central Oriental Andes	G	-78.0408	-3.0383
			DQ502013	*Epipedobates*	*sp. "San Jacinto"*	P, C, L	KU 221841	Peru	PE	Loreto: San Jacinto	175	Amazon	C	-75.750	-2.4167

Table S3 (Cont) Taxa used to infer the phylogeny and the chronogram of the poison frogs. 'Use' identifies which samples were used for the phylogeny (P), chronogram (C), and Langrage (L) analyses

12S	16S beginning	16S end	12S-16S	Genera	Species	Use	Museum	Country	Code	Locality	altitude (m)	Area	Code	Long	Lat
			AY364574	*Epipedobates*	*parvulus*	P, C, L	QCAZ16583	Ecuador	EC	Morona Santiago: near Mendez	550	Central Oriental Andes	G	-78.3034	-2.7454
DQ522973		DQ523044		*Epipedobates*	*pongoensis*	P, L	none	Peru	PE	San Martin: Tarapoto, Convento	200	Amazon	C	-76.31459	-6.25107
			EU342609	*Epipedobates*	*bassleri*	P, C, L	MHNSM22600	Peru	PE	San Martin: 19.5 Km E Tarapoto, road to Yurimaguas	991	Central Oriental Andes	G	-76.2958	-6.4466
			EU342610	*Epipedobates*	*bassleri*	P, L	MHNSM22684	Peru	PE	San Martin: 19.5 Km E Tarapoto, road to Yurimaguas	991	Central Oriental Andes	G	-76.2958	-6.4466
			EU342611	*Epipedobates*	*sp.*	P, C, L	QCAZ25305	Ecuador	EC	Sucumbios: Laguna Grande-Neotropic, Reserva de Producción Faunística Cuyabeno	260	Amazon	C	-76.216667	0
			AY364571	*Epipedobates*	*bilinguis*	P, C, L	QCAZ16576	Ecuador	EC	Francisco de Orellana: Parque Nacional Yasuni-Estacion PUCE	230	Amazon	C	-76.50	-0.633
			EU342612	*Epipedobates*	*bilinguis*	P, L	QCAZ16577	Ecuador	EC	Francisco de Orellana: Cotapino	609	Central Oriental Andes	G	-77.4667	-0.70
			DQ283073	*Epipedobates*	*silverstonei*	P, C, L	none	Peru	PE	San Martin-Hanuco	n.d.	Central Oriental Andes	G	n.d.	n.d.
			DQ502252	*Epipedobates*	*pictus*	P, C, L	BPN 1074	Guyana	GU	Mazaruni-Potaro: Kartabo Pt. (confluence of Cyuni & Mazaruni Rivers)	30	Guiana Shield	B	-58.667	6.5
		AY263239		*Epipedobates*	*sp*	P, L	CBF3900	Bolivia	BO	Bolivia	n.d.	Amazon	C	n.d.	n.d.
			EU342613	*Epipedobates*	*pictus*	P, C, L	MNCN4167	Bolivia	BO	Santa Cruz: Ichilo, Parque Natural Amboro, Campamento La Chonta	480	Central Oriental Andes	G	-62.6333	-19.7167
			DQ502125	*Epipedobates*	*braccatus*	P, C, L	MRT 5603	Brazil	BR	Mato Grosso: APM Manso	250	Brazilian Shield	K	-58.733	-15.45
			DQ502124	*Epipedobates*	*flavopictus*	P, C, L	MZUSP 111790	Brazil	BR	Tocantins: Parana	400	Brazilian Shield	K	-47.866	-12.6
			DQ502033	*Epipedobates*	*pulchripectus*	P, C, L	CWM 19053	Brazil	BR	Amapa: Serra do Navio	140	Guiana Shield	B	-52.05	0.9833
			DQ502270	*Epipedobates*	*hahneli*	P, C, L	ICN 50410	Colombia	CO	Amazona:, Leticia, Lago Yahuarcaca	90	Amazon	C	-70	-4.166
			AY364573	*Epipedobates*	*hahneli*	P, C, L	QCAZ13325	Ecuador	EC	Francisco de Orellana: Parque Nacional Yasuni-Estacion PUCE	230	Amazon	C	-76.50	-0.633

Table S3 (Cont) Taxa used to infer the phylogeny and the chronogram of the poison frogs. 'Use' identifies which samples were used for the phylogeny (P), chronogram (C), and Langrage (L) analyses

12S	16S beginning	16S end	12S-16S	Genera	Species	Use	Museum	Country	Code	Locality	altitude (m)	Area	Code	Long	Lat
			EU342614	*Epipedobates*	*hahneli*	P, L	LSUMZ13688	Brazil	BR	Acre: near 5 km N Porto Walker, inland from the Rio Jurua	200	Amazon	C	-72.776972	-8.258667
			EU342615	*Epipedobates*	*hahneli*	P, C, L	LSUMZ13650	Brazil	BR	Acre: near 5 km N Porto Walker, inland from the Rio Jurua	200	Amazon	C	-72.776972	-8.258667
			EU342616	*Epipedobates*	*pictus*	P, L	KU215183	Peru	PE	Madre de Dios: Cuzco Amazonico, 15 km E Puerto Maldonado	200	Amazon	C	-70.08	-12.60
			EU342617	*Epipedobates*	*pictus*	P, C, L	KU215184	Peru	PE	Madre de Dios: Cuzco Amazonico, 15 km E Puerto Maldonado	200	Amazon	C	-70.08	-12.60
			EU342618	*Epipedobates*	*hahneli*	P, L	LSUMZ16931	Brazil	BR	Amazonas: Castanho, ca. 40 km S Manaus at km 12 on road to Autazes	40	Amazon	C	-60.455111	-3.619556
			EU342619	*Epipedobates*	*hahneli*	P, C, L	LSUMZ16923	Brazil	BR	Amazonas: Castanho, ca. 40 km S Manaus at km 12 on road to Autazes	40	Amazon	C	-60.455111	-3.619556
DQ523020		DQ523090		*Epipedobates*	*simulans*	P, L	none	Peru	PE	Cuzco: Quincemille	490	Central Oriental Andes	G	-70.63308	-13.18396
			DQ502114	*Epipedobates*	*petersi*	P, C, L	MJH 7041	Peru	PE	Huanuco: Rio Llullapichis, Panguana	270	Amazon	C	-74.93	-9.62
DQ522971		DQ523042		*Epipedobates*	*smaragdinus*	P, L	none	Peru	PE	Pasco: Iscozazin	350	Central Oriental Andes	G	-75.16052	-10.18879
			DQ502116	*Epipedobates*	*petersi*	P, C, L	MJH 3715	Peru	PE	Peru: Humboldt	n.d.	Central Oriental Andes	G	n.d.	n.d.
			EU342620	*Epipedobates*	*cainarachi*	P, C, L	MHNSM22720	Peru	PE	San Martin: Shapaja-Chazuta road 15.5 km	347	Central Oriental Andes	G	-76.17211	-6.61735
			EU342621	*Epipedobates*	*cainarachi*	P, L	MHNSM22729	Peru	PE	San Martin: Shapaja-Chazuta road 15.5 km	347	Central Oriental Andes	G	-76.17211	-6.61735
			EU342622	*Epipedobates*	*hahneli*	P, C, L	KS40	Peru	PE	Cuzco: Ivochote	620	Central Oriental Andes	G	-72.99385	-12.47086
			EU342623	*Epipedobates*	*rubriventris*	P, C, L	KS37	Peru	PE	San Martin: road to San Jose de Sisa	509	Central Oriental Andes	G	-76.37298	-6.380222
			DQ502155	*Epipedobates*	*macero*	P, C, L	LR 742	Peru	PE	Madre de Dios: Parque Nacional del Manu	295	Amazon	C	-70.90	-12.25

Table S3 (Cont) Taxa used to infer the phylogeny and the chronogram of the poison frogs. 'Use' identifies which samples were used for the phylogeny (P), chronogram (C), and Langrage (L) analyses

12S	16S beginning	16S end	12S-16S	Genera	Species	Use	Museum	Country	Code	Locality	altitude (m)	Area	Code	Long	Lat
			EU342624	*Epipedobates*	*sp1cfpetersi*	P, C, L	LSUMZ13646	Brazil	BR	Acre: near 5 km N Porto Walker, inland from the Rio Jurua	200	Amazon	C	-72.776972	-8.258667
			EU342625	*Epipedobates*	*sp1cfpetersi*	P, L	LSUMZ13635	Brazil	BR	Acre: near 5 km N Porto Walker, inland from the Rio Jurua	200	Amazon	C	-72.776972	-8.258667
			EU342626	*Epipedobates*	*trivittatus*	P, C, L	KU212009	Peru	PE	San Martin: 14km ESE Shapaja	310	Central Oriental Andes	G	-76.2833	-6.6
			EU342627	*Epipedobates*	*trivittatus*	P, L	KS36	Peru	PE	San Martin: Cainarachi Valley	350	Central Oriental Andes	G	-76.31758	-6.45043
			EU342628	*Epipedobates*	*trivittatus*	P, L	KS26	Peru	PE	San Martin: Tarapoto	540	Central Oriental Andes	G	-76.29034	-6.43066
			DQ502021	*Epipedobates*	*trivittatus*	P, L	USNM 268846	Peru	PE	Madre de Dios: Puerto Maldonado Explorer's Inn, 30 km (airline) SSW of Tambopata Reserve	230	Amazon	C	-69.165	-12.88
			EU342629	*Epipedobates*	*trivittatus*	P, L	LSUMZ16912	Brazil	BR	Amazonas: Castanho, ca. 40 km S Manaus at km 12 on road to Autazes	40	Amazon	C	-60.455111	-3.619556
			EU342630	*Epipedobates*	*trivittatus*	P, C, L	LM312	Surinam	SU	Brokopondo: Brokopondo	72	Guiana Shield	B	-54.9667	5.0667
			DQ502112	*Epipedobates*	*trivittatus*	P, L	MJH 3907	Brazil	BR	Amazonas: Base 2 island in reservoir of Uatuma river, 8 km NW represa de Balbina	140	Amazon	C	-59.466	-1.8833
			DQ502250	*Epipedobates*	*trivittatus*	P, L	BPN 910	Guyana	GU	Suriname: Para, Paramaribo-Apura road	20	Guiana Shield	B	-55.11	5.525
			DQ502111	*Epipedobates*	*trivittatus*	P, L	MJH 7483	Peru	PE	Huanuco: Rio Llullapichis, Panguana	270	Amazon	C	-74.93	-9.62
			EU342631	*Epipedobates*	*trivittatus*	P, L	KS39	Peru	PE	Loreto: Tahuayo	140	Amazon	C	-73.10457	-4.18707
			EU342632	*Epipedobates*	*trivittatus*	P, C, L	TNHCFS4966	Colombia	CO	Amazonas: Leticia, Cerca Viva	83	Amazon	C	-69.9406	-4.2153
			EU342633	*Epipedobates*	*trivittatus*	P, L	LSUMZ13693	Brazil	BR	Acre: near 5 km N Porto Walker, inland from the Rio Jurua	200	Amazon	C	-72.776972	-8.258667
			EU342634	*Epipedobates*	*trivittatus*	P, L	LSUMZ13661	Brazil	BR	Acre: near 5 km N Porto Walker, inland from the Rio Jurua	200	Amazon	C	-72.776972	-8.258667
			EU342689	*Colostethus*	*leucophaeus*	P, C, L	KU211880	Peru	PE	Amazonas: Molinopampa	2400	Central Oriental Andes	G	-77.63333	-6.1833

Table S3 (Cont) Taxa used to infer the phylogeny and the chronogram of the poison frogs. 'Use' identifies which samples were used for the phylogeny (P), chronogram (C), and Langrage (L) analyses

12S	16S beginning	16S end	12S-16S	Genera	Species	Use	Museum	Country	Code	Locality	altitude (m)	Area	Code	Long	Lat
			EU342690	*Colostethus*	*sordidatus*	P, C, L	MHNSM22690	Peru	PE	San Martin: road to San Jose de Sisa	509	Central Oriental Andes	G	-76.37298	-6.380222
			EU342691	*Colostethus*	*sordidatus*	P, L	MHNSM22679	Peru	PE	San Martin: road to San Jose de Sisa	509	Central Oriental Andes	G	-76.37298	-6.380222
			EU342692	*Colostethus*	*ranoides*	P, L	TNHCFS4940	Colombia	CO	Casanare: Agua Azul	336	North Oriental Andes	E	-72.580306	5.19
			DQ502273	*Colostethus*	*subpunctatus*	P, C, L	none	Colombia	CO	Cundinamarca: Bogota D.C.	2550	North Oriental Andes	E	-74.104	4.684
			EU342693	*Colostethus*	*subpunctatus*	P, C, L	TNHCFS4957	Colombia	CO	Boyaca: Chiquinquira	2575	North Oriental Andes	E	-73.811139	5.6215
			EU342694	*Colostethus*	*sp*	P, C, L	QCAZ19971	Ecuador	EC	Napo: Puerto Misahualli- vía Pununo,jardin Aleman	550	North Oriental Andes	E	-77.6667	-1.0333
			AY364545	*Colostethus*	*bocagei*	P, C, L	QCAZ20826	Ecuador	EC	Sucumbios: La Libertad road to La Virgen	1680	North Oriental Andes	E	-77.586	-0.07
			EU342695	*Colostethus*	*sauli*	P, C, L	QCAZ27126	Ecuador	EC	Napo: Río Arajuno, Sendero 1	295	Amazon	C	-77.5333	-1.0833
			EU342696	*Colostethus*	*sauli*	P, C, L	QCAZ16542	Ecuador	EC	Francisco de Orellana: Parque Nacional Yasuni-Estacion PUCE	230	Amazon	C	-76.50	-0.633
			AY364555	*Colostethus*	*sauli*	P, L	QCAZ16541	Ecuador	EC	Francisco de Orellana: Parque Nacional Yasuni-Estacion PUCE	230	Amazon	C	-76.50	-0.633
			AY364560	*Colostethus*	*spE*	P, C, L	QCAZ16503	Ecuador	EC	Sucumbios: Bermeja, 2 km NO Rio Bermejo	521	North Oriental Andes	E	-77.0794	0.2831
			EU342697	*Colostethus*	*spE*	P, L	QCAZ20007	Ecuador	EC	Sucumbios: Laguna Grande-Neotropic, Reserva de Producción Faunística Cuyabeno	260	Amazon	C	-76.216667	0
			EU342698	*Colostethus*	*sp.*	P, L	QCAZ31144	Ecuador	EC	Napo: Archidona, road to Pinguyo waterfall, Wawa Sumaco	1095	North Oriental Andes	E	-77.56972	-0.72898
			EU342699	*Colostethus*	*spD1*	P, C, L	QCAZ16505	Ecuador	EC	Francisco de Orellana: Cotapino	609	North Oriental Andes	E	-77.4667	-0.7
			AY364559	*Colostethus*	*spD2*	P, L	QCAZ16504	Ecuador	EC	Francisco de Orellana: Cononaco, Pozo 16	340	North Oriental Andes	E	-77.042	-0.833
			EU342700	*Colostethus*	*spD2*	P, C, L	QCAZ19909	Ecuador	EC	Francisco de Orellana: Cononaco, Pozo 16	340	North Oriental Andes	E	-77.042	-0.833

Table S3 (Cont) Taxa used to infer the phylogeny and the chronogram of the poison frogs. 'Use' identifies which samples were used for the phylogeny (P), chronogram (C), and Langrage (L) analyses

12S	16S beginning	16S end	12S-16S	Genera	Species	Use	Museum	Country	Code	Locality	altitude (m)	Area	Code	Long	Lat
			AY364558	*Colostethus*	*spC*	P, L	QCAZ16511	Ecuador	EC	Morona Santiago: 2 km E Santiago	495	North Oriental Andes	E	-78.0408	-3.0383
			EU342701	*Colostethus*	*spC*	P, C, L	QCAZ16510	Ecuador	EC	Morona Santiago: 2 km E Santiago	495	North Oriental Andes	E	-78.0408	-3.0383
			EU342702	*Colostethus*	*spF*	P, L	KU220616	Ecuador	EC	Pastaza: Locacion Petrolera Garza 1, NE Motalvo	266	Amazon	C	-76.966	-2.0667
			EU342703	*Colostethus*	*spF*	P, L	KU220615	Ecuador	EC	Pastaza: Locacion Petrolera Garza 1, NE Motalvo	266	Amazon	C	-76.966	-2.0667
			AY364552	*Colostethus*	*maculosus*	P, C, L	QCAZ16508	Ecuador	EC	Pastaza: El Porvenir (to Palora), near Río Puyo	1050	North Oriental Andes	E	-77.842	-1.636
			EU342705	*Colostethus*	*maculosus*	P, L	QCAZ25539	Ecuador	EC	Pastaza: Kapawi Lodge	239	Amazon	C	-76.830812	-2.540804
			EU342704	*Colostethus*	*maculosus*	P, L	QCAZ16507	Ecuador	EC	Pastaza: El Porvenir (to Palora), near Río Puyo	1050	North Oriental Andes	E	-77.842	-1.636
			EU342706	*Colostethus*	*sylvaticus*	P, C, L	KU219756	Peru	PE	Piura: 12.7 km E El Tambo	2770	Central Occidental Andes	H	-79.55	-5.35
			EU342707	*Colostethus*	*pulcherrimus*	P, C, L	KU211948	Peru	PE	Cajamarca: Cutervo	2620	Central Oriental Andes	G	-78.816667	-6.366667
			EU342708	*Colostethus*	*anthracinus*	P, C, L	KU23489	Ecuador	EC	Azuay: 10 km S Cutchil	2674	North Oriental Andes	E	-78.80	-3.0667
			EU342709	*Colostethus*	*nexipus*	P, C, L	QCAZ16537	Ecuador	EC	Morona Santiago: Indanza-San Miguel del Conchay	855	Central Oriental Andes	G	-78.466667	-3.066667
			AY364553	*Colostethus*	*nexipus*	P, C, L	QCAZ16534	Ecuador	EC	Morona Santiago: near Mendez	550	Central Oriental Andes	G	-78.3034	-2.7454
			EU342710	*Colostethus*	*nexipus*	P, L	QCAZ16535	Ecuador	EC	Morona Santiago: near Mendez	550	Central Oriental Andes	G	-78.3034	-2.7454
			EU342711	*Colostethus*	*nexipus*	P, C, L	KU212486	Peru	PE	San Martín:6 km ESE Shapaja	310	Central Oriental Andes	G	- 76.2833	- 6.60
			EU342712	*Colostethus*	*nexipus*	P, L	KU211808	Peru	PE	San Martin: Cataratas Ahuashiyacu, 14 km NE Tarapoto	730	Central Oriental Andes	G	-76.3333	-6.50
			EU342713	*Colostethus*	*nexipus*	P, L	KU211806	Peru	PE	San Martin: Cataratas Ahuashiyacu, 14 km NE Tarapoto	730	Central Oriental Andes	G	-76.3333	-6.50
			EU342714	*Colostethus*	*nexipus*	P, L	KS43	Peru	PE	San Martin: Tarapoto	540	Central Oriental Andes	G	-76.29034	-6.43066

Table S3 (Cont) Taxa used to infer the phylogeny and the chronogram of the poison frogs. 'Use' identifies which samples were used for the phylogeny (P), chronogram (C), and Langrage (L) analyses

12S	16S beginning	16S end	12S-16S	Genera	Species	Use	Museum	Country	Code	Locality	altitude (m)	Area	Code	Long	Lat
			AY364564	*Colostethus*	*vertebralis*	P, C, L	QCAZ16554	Ecuador	EC	Azuay: El Jordán (cerca a Paguancay)	2424	Central Oriental Andes	G	-78.6	-2.633333
			EU342715	*Colostethus*	*vertebralis*	P, C, L	KU220675	Ecuador	EC	Azuay: 10-15 km E Luz Maria	2450	Central Occidental Andes	E	-79.331	-2.666
			DQ502174	*Colostethus*	*sp. "Ibague"*	P, C, L	MUJ 3564	Colombia	CO	Tolima: Ibague, El Totumo, finca La Magnolia, Quebrada El Cural	1047	North Occidental Andes	F	-75.18	4.378
			EU342716	*Colostethus*	*cf. puchellus*	P, C, L	KU220666	Ecuador	EC	Carchi: Monte Olivo	2713	North Oriental Andes	E	-77.8833	0.400
			EU342717	*Colostethus*	*cf. puchellus*	P, C, L	KU202833	Ecuador	EC	Sucumbios: 60 km E San Miguel de Salcedo	2550	North Oriental Andes	E	-77.563	0.451
			EU342718	*Colostethus*	*delatorreae*	P, C, L	KU220619	Ecuador	EC	Carchi: 18.5 km E Maldonado	2420	North Occidental Andes	F	-78.01	0.904
			EU342719	*Colostethus*	*delatorreae*	P, L	KU220620	Ecuador	EC	Carchi: 18.5 km E Maldonado	2420	North Occidental Andes	F	-78.01	0.904
			AY364554	*Colostethus*	*pulchellus*	P, C, L	QCAZ15964	Ecuador	EC	Sucumbios: Rio Azuela	1680	North Oriental Andes	E	-77.65	-0.1667
			EU342720	*Colostethus*	*pulchellus*	P, L	QCAZ15965	Ecuador	EC	Sucumbios: Rio Azuela	1680	North Oriental Andes	E	-77.65	-0.1667
			EU342721	*Colostethus*	*pulchellus*	P, L	QCAZ16539	Ecuador	EC	Napo: Yanuyaco near Cosanga	2215	North Oriental Andes	E	-77.866	-0.6
			EU342722	*Colostethus*	*pulchellus*	P, L	QCAZ16540	Ecuador	EC	Napo: Yanuyaco near Cosanga	2215	North Oriental Andes	E	-77.866	-0.6
			EU342723	*Colostethus*	*spA*	P, C, L	QCAZ16492	Ecuador	EC	Morona Santiago: near El Copal, 27 Km S Méndez-Cuenca	1036	Central Oriental Andes	G	-78.383333	-2.716667
			EU342724	*Colostethus*	*spA*	P, L	QCAZ16493	Ecuador	EC	Morona Santiago: near El Copal, 27 Km S Méndez-Cuenca	1036	Central Oriental Andes	G	-78.383333	-2.716667
			AY364556	*Colostethus*	*spA*	P, L	QCAZ16490	Ecuador	EC	Morona Santiago: conjuction of Rio Paute and Rio Negro	540	Central Oriental Andes	G	-78.3034	-2.7454
			EU342725	*Colostethus*	*craspedoceps*	P, C, L	MHNSM22882	Peru	PE	San Martin: road to San Jose de Sisa	509	Central Oriental Andes	G	-76.37298	-6.380222
			EU342726	*Colostethus*	*craspedoceps*	P, L	MHNSM22906	Peru	PE	San Martin: road to San Jose de Sisa	509	Central Oriental Andes	G	-76.37298	-6.380222
			EU342727	*Colostethus*	*craspedoceps*	P, L	MHNSM22886	Peru	PE	San Martin: road to San Jose de Sisa	509	Central Oriental Andes	G	-76.37298	-6.380222

Table S3 (Cont) Taxa used to infer the phylogeny and the chronogram of the poison frogs. 'Use' identifies which samples were used for the phylogeny (P), chronogram (C), and Langrage (L) analyses

12S	16S beginning	16S end	12S-16S	Genera	Species	Use	Museum	Country	Code	Locality	altitude (m)	Area	Code	Long	Lat
			EU342728	*Cryptophyllobates*	*azureiventris*	P, C, L	KS33	Peru	PE	San Martin: Cainarachi Valley	350	Central Oriental Andes	G	-76.31758	-6.45043
			EU342729	*Cryptophyllobates*	*azureiventris*	P, L	KS28	Peru	PE	San Martin: Bonilla	200	Amazon	C	-76.27226	-6.21007
			DQ502080	*Cryptophyllobates*	*chlorocraspedus*	P, C, L	MPEG 13853	Brazil	BR	Amazonas: Castanho, ca. 40 km S Manaus, at km 12 on road to Autazes	40	Amazon	C	-60.455111	-3.619556
			DQ502078	*Cryptophyllobates*	*chlorocraspedus*	P, L	OMNH 36170	Brazil	BR	Acre: near 5 km N Porto Walker, inland from the Rio Jurua	200	Amazon	C	-72.776972	-8.258667
			EU342730	*Cryptophyllobates*	*chlorocraspedus*	P, L	LSUMZ13664	Brazil	BR	Acre: near 5 km N Porto Walker, inland from the Rio Jurua	200	Amazon	C	-72.776972	-8.258667
			EU342731	*Colostethus*	*idiomelus*	P, C, L	KU211908	Peru	PE	San Martín: E slope Abra Pardo de Miguel	2180	Central Oriental Andes	G	-77.7	-5.766667
			EU342732	*Colostethus*	*idiomelus*	P, L	KU211885	Peru	PE	Amazonas: Pomacochas	2150	Central Oriental Andes	G	-77.9167	-5.8333
			EU342733	*Colostethus*	*insulatus*	P, C, L	KU211862	Peru	PE	Amazonas: 17 km ENE Balsas	1090	Central Oriental Andes	G	-77.983333	-6.833333
			EU342734	*Colostethus*	*insulatus*	P, L	KU211878	Peru	PE	Amazonas: Bongara, 6 km W Pedro Ruiz Gallo	1260	Central Oriental Andes	G	-77.95	-5.416667
			DQ501992	*Colostethus*	*insulatus*	P, L	KU 212522	Peru	PE	Cajamarca: Chota, 4 km W Llama	2638	Central Occidental Andes	H	-79.1203	-6.5144
			EU342735	*Colostethus*	*insulatus*	P, L	KU211837	Peru	PE	Cajamarca: 2 km NW San Juan	2290	Central Occidental Andes	H	-78.5	-7.25
			EU342736	*Colostethus*	*elachyhistus*	P, C, L	KU212507	Peru	PE	Piura: NE slope Abra de Porculla	2200	Central Occidental Andes	H	-79.5	-5.8333
			EU342737	*Colostethus*	*elachyhistus*	P, C, L	KU219746	Peru	PE	Piura: 8.5 km W Canchaque	620	Central Occidental Andes	H	-79.6	-5.373
			EU342738	*Colostethus*	*elachyhistus*	P, C, L	KU212516	Peru	PE	Piura: Ayabaca	2750	Central Occidental Andes	H	-79.7167	-4.6333
			EU342739	*Colostethus*	*elachyhistus*	P, C, L	QCAZ16521	Ecuador	EC	El Oro: Pasaje-Cuenca road, 23.5 km	1200	Central Occidental Andes	H	-79.6	-3.303
			AY364546	*Colostethus*	*elachyhistus*	P, L	QCAZ16518	Ecuador	EC	El Oro: Torata-Balsas road	640	Central Occidental Andes	H	-79.85	-3.716667
			EU342740	*Colostethus*	*elachyhistus*	P, C, L	QCAZ16517	Ecuador	EC	El Oro: Torata-Balsas road	640	Central Occidental Andes	H	-79.85	-3.716667
			EU342741	*Colostethus*	*infraguttatus*	P, L	QCAZ19958	Ecuador	EC	Guayas: Cerro Masvalle	92	Choco	I	-79.642	-2.394

Table S3 (Cont) Taxa used to infer the phylogeny and the chronogram of the poison frogs. 'Use' identifies which samples were used for the phylogeny (P), chronogram (C), and Langrage (L) analyses

12S	16S beginning	16S end	12S-16S	Genera	Species	Use	Museum	Country	Code	Locality	altitude (m)	Area	Code	Long	Lat
			EU342742	*Colostethus*	*spG*	P, C, L	QCAZ21978	Ecuador	EC	Cotopaxi: Corazón-Moraspungo	1250	North Occidental Andes	F	-79.12	-1.18
			AY364548	*Colostethus*	*infraguttatus*	P, C, L	QCAZ16516	Ecuador	EC	Manabi: road Portoviejo - Jipijapa at 6 Km from Puerto Cayo	167	Choco	I	-80.708	-1.324
			AY326028	*Colostethus*	*infraguttatus*	P, C, L	QCAZ10812	Ecuador	EC	Manabi: road Portoviejo - Jipijapa at 6 Km from Puerto Cayo	167	Choco	I	-80.708	-1.324
			EU342743	*Colostethus*	*awa*	P, C, L	KU220610	Ecuador	EC	Manabi: 38 km NW El Carmen, road El Carmen-Pedernales	330	Choco	I	-79.761	-0.196
			AY364563	*Colostethus*	*toachi*	P, C, L	QCAZ16548	Ecuador	EC	Carchi: Rio Baboso near Lita	534	North Occidental Andes	F	-78.4667	0.8333
			EU342744	*Colostethus*	*toachi*	P, L	QCAZ16549	Ecuador	EC	Carchi: Rio Baboso near Lita	534	North Occidental Andes	F	-78.4667	0.8333
			AY364544	*Colostethus*	*awa*	P, L	QCAZ16498	Ecuador	EC	Pichincha: Remolino, Rio Guayabamba	900	North Occidental Andes	F	-78.67	0.1247
			EU342745	*Colostethus*	*awa*	P, C, L	QCAZ16497	Ecuador	EC	Imbabura: Rio Verde (Remolino-Sahuangal)	890	North Occidental Andes	F	-78.68	0.22
			EU342635	*Phyllobates*	*vittatus*	P, C, L	TNHC64421	Costa Rica	CR	Puntarenas	n.d.	Central America	J	n.d.	n.d.
			DQ283043	*Phyllobates*	*lugubris*	P, C, L	USNM-FS 195116	Panama	PA	Bocas del Toro: S end of Isla Popa, 1 km E Sumwood Channel	6	Central America	J	-82.1167	9.1833
			EU342636	*Phyllobates*	*lugubris*	P, C, L	LSUMZ15096	Nicaragua	NI	Rio San Juan: Rio San Juan, Near Isla de Diamante (ca. 15 km SE El Castillo on Rio San Juan)	80	Central America	J	-84.30	10.9333
			EU342637	*Phyllobates*	*lugubris*	P, L	LSUMZ15098	Nicaragua	NI	Rio San Juan: Rio San Juan, Near Isla de Diamante (ca. 15 km SE El Castillo on Rio San Juan)	80	Central America	J	-84.30	10.9333
			EU342638	*Phyllobates*	*aurotaenia*	P, C, L	TNHCFS4990	Colombia	CO	Choco: Quibdo road to Pacuritas	50	Choco	I	-76.61086	5.683944
			EU342639	*Phyllobates*	*terribilis*	P, C, L	TNHC64420	Colombia	CO	Pet Trade	n.d.	Choco	I	n.d.	n.d.
			DQ502157	*Phyllobates*	*terribilis*	P, L	AMNH A118566	Colombia	CO	Cauca: Quebrada Guangui, 0.5 km above Rio Patia (upper Saija drainage)	150	Choco	I	-77.4667	2.8
			EU342640	*Phyllobates*	*aurotaenia*	P, C, L	TNHC64419	Colombia	CO	Pet Trade	n.d.	Choco	I	n.d.	n.d.
			AY364580	*Phyllobates*	*bicolor*	P, C, L	TNHC62488	Colombia	CO	Pet Trade	n.d.	Choco	I	n.d.	n.d.
DQ371310		DQ371321		*Dendrobates*	*steyermarki*	P, L	none	Venezuela	VE	Amazonas: Cerro Yapacana	1050	Guiana Shield	B	-66.783	3.686

Table S3 (Cont) Taxa used to infer the phylogeny and the chronogram of the poison frogs. 'Use' identifies which samples were used for the phylogeny (P), chronogram (C), and Langrage (L) analyses

12S	16S beginning	16S end	12S-16S	Genera	Species	Use	Museum	Country	Code	Locality	altitude (m)	Area	Code	Long	Lat
		AY263244		*Dendrobates*	*steyermarki*	P, L	none	Venezuela	VE	Amazonas: Cerro Yapacana	1050	Guiana Shield	B	-66.783	3.686
			EU342641	*Dendrobates*	*galactonotus*	P, C, L	TNHCFS4889	Brazil	BR	Amazonas	n.d.	Amazon/Brazilian Shield	CK	n.d.	n.d.
			EU342642	*Dendrobates*	*castaneoticus*	P, C, L	LSUMZ15209	Brazil	BR	Para: 101 km S, 15 km E Santarem CEMEX (near Rio Curua-Una)	120	Amazon	C	-54.833	-3.15
			EU342643	*Dendrobates*	*castaneoticus*	P, L	LSUMZ15185	Brazil	BR	Para: 101 km S, 15 km E Santarem CEMEX (near Rio Curua-Una)	120	Amazon	C	-54.833	-3.15
			EU342644	*Dendrobates*	*quinquevittatus*	P, C, L	LSUMZ17435	Brazil	BR	Rondonia: Rio Formoso, Parque Estadual Guajara-Mirim, 90 km N Nova Mamore	180	Amazon	C	-64.563306	-10.321
			EU342645	*Dendrobates*	*quinquevittatus*	P, L	LSUMZ13025	Brazil	BR	Amazonas: Rio Ituxi at the Madeireira Scheffer; left bank	110	Amazon	C	-65.716556	-8.479389
			EU342646	*Dendrobates*	*quinquevittatus*	P, L	LSUMZ17471	Brazil	BR	Rondonia: Rio Formoso, Parque Estadual Guajara-Mirim, 90 km N Nova Mamore	180	Amazon	C	-64.563306	-10.321
			DQ502035	*Dendrobates*	*granuliferus*	P, C, L	CWM 19044	Costa Rica	CR	Puntarenas: about 6 km airline E Palmar Norte, stream draining into Rio Granada de Terraba	90	Central America	J	-80.404	8.964
			EU342658	*Dendrobates*	*granuliferus*	P, L	TNHC64390	Costa Rica	CR	Puntarenas: Peninsula de la Osa, Sirena	80	Central America	J	-53.588	8.48
			EU342659	*Dendrobates*	*granuliferus*	P, C, L	KS20	Costa Rica	CR	Puntarenas: Peninsula de la Osa, Sirena	80	Central America	J	-53.588	8.48
			DQ502034	*Dendrobates*	*lehmanni*	P, C, L	CWM 19050	Colombia	CO	Valle del Cauca: Anchicaya Valley near Dagua (type locality)	1488	North Occidental Andes	F	-76.6928	3.6603
			EU342660	*Dendrobates*	*sylvaticus*	P, C, L	QCAZ27423	Ecuador	EC	Esmeraldas: Guadual	187	Choco	I	-79.1667	0.8
			AY364569	*Dendrobates*	*sylvaticus*	P, L	QCAZ16563	Ecuador	EC	Esmeraldas: near Quingue	306	Choco	I	-80.0833	0.7167
			EU342661	*Dendrobates*	*sylvaticus*	P, L	QCAZ16562	Ecuador	EC	Pichincha: near Santo Domingo de los Colorados	604	North Occidental Andes	F	-79.15	-0.25
			EU342662	*Dendrobates*	*histrionicus*	P, C, L	TNHCFS4879	Colombia	CO	Choco: Quibdo, La Troje	50	Choco	I	-76.591	5.728
			DQ502105	*Dendrobates*	*histrionicus*	P, L	MHNUC 344	Colombia	CO	Choco: Bahia Solano, Sierra Mecana	330	Choco	I	- 77.35	6.266667

Table S3 (Cont) Taxa used to infer the phylogeny and the chronogram of the poison frogs. 'Use' identifies which samples were used for the phylogeny (P), chronogram (C), and Langrage (L) analyses

12S	16S beginning	16S end	12S-16S	Genera	Species	Use	Museum	Country	Code	Locality	altitude (m)	Area	Code	Long	Lat
			DQ502036	*Dendrobates*	*arboreus*	P, C, L	CWM 18636	Panama	PA	Chiriqui: Continental divide above upper Quebrada de Arena	1120	Central America	J	-82.6833	8.7167
			EU342663	*Dendrobates*	*pumilio*	P, C, L	TNHCFS4814	Panama	PA	Bocas del Toro: Isla Colon, Bocas del Drago (Dragomar)	11	Central America	J	-82.32038	9.42479
			DQ502037	*Dendrobates*	*speciosus*	P, C, L	CWM 17826(D)	Panama	PA	Chiriqui: Continental divide above upper Quebrada de Arena	1350	Central America	J	-82.6833	8.7167
AF128585		AF128596		*Dendrobates*	*speciosus*	P, L	none	Panama	PA	Chiriqui: Fortuna	1188	Central America	J	-82.2667	8.7167
			DQ502167	*Dendrobates*	*vicentei*	P, C, L	KRL 789	Panama	PA	Cocle: El Cope, Parque Nacional General de Division 'Omar Torrijos Herrera	782	Central America	J	-80.592	8.667
			DQ502237	*Dendrobates*	*pumilio*	P, C, L	OMNH 33300	Nicaragua	NI	Rio San Juan: Rio San Juan, Near Isla de Diamante (ca. 15 km SE El Castillo on Rio San Juan)	80	Central America	J	-84.30	10.9333
			EU342664	*Dendrobates*	*pumilio*	P, C, L	MVZ231703	Costa Rica	CR	Limon: Tortuguero, Green Turtle Station, Atlantic Cost (NE Costa Rica, near Nicaraguan border)	0	Central America	J	-83.5167	10.5833
			EU342647	*Dendrobates*	*leucomelas*	P, C, L	TNHC64415	Venezuela	VE	Amazonas	n.d.	Guiana Shield	B	n.d.	n.d.
			EU342648	*Dendrobates*	*leucomelas*	P, L	KS2	Venezuela	VE	Amazonas: Tobogan	630	Guiana Shield	B	-63.75	2.2333
			DQ502248	*Dendrobates*	*tinctorius*	P, C, L	UTA A56495	Surinam	SU	Sipaliwini: ca. 4.0 km N of Tafelberg airstrip	300	Guiana Shield	B	-56.15	3.7833
			EU342649	*Dendrobates*	*tinctorius*	P, L	TNHC64416	Surinam	SU	Sipaliwini	n.d.	Guiana Shield	B	n.d.	n.d.
			DQ502251	*Dendrobates*	*azureus*	P, L	BPN 977	Surinam	SU	Sipaliwini: forest island on W slope of Vier Gebroders Mts., Sipaliwini savannah	310	Guiana Shield	B	-56.1	2.033
			EU342650	*Dendrobates*	*azureus*	P, C, L	TNHC64414	Surinam	SU	Sipaliwini	n.d.	Guiana Shield	B	n.d.	n.d.
			EU342651	*Dendrobates*	*truncatus*	P, L	TNHC64417	Colombia	CO	Magdalena Valley	n.d.	Choco	I	n.d.	n.d.
			DQ502269	*Dendrobates*	*truncatus*	P, L	ICN 48477	Colombia	CO	Cordoba: Pueblonuevo, finca Embajada	40	Choco	I	-75.356111	8.418611
			EU342652	*Dendrobates*	*truncatus*	P, L	TNHCFS4979	Colombia	CO	Tolima: Mariquita, vereda Malabares	587	North Occidental Andes	F	-74.894333	5.259417
			EU342653	*Dendrobates*	*truncatus*	P, C, L	TNHCFS4953	Colombia	CO	Tolima: Melgar	336	Choco	I	-74.6456	4.2072

Table S3 (Cont) Taxa used to infer the phylogeny and the chronogram of the poison frogs. 'Use' identifies which samples were used for the phylogeny (P), chronogram (C), and Langrage (L) analyses

12S	16S beginning	16S end	12S-16S	Genera	Species	Use	Museum	Country	Code	Locality	altitude (m)	Area	Code	Long	Lat
			EU342654	*Dendrobates*	*auratus*	P, C, L	TNHCFS4813	Panama	PA	Panama: Lago Bayano	115	Choco	I	-78.77941	9.17556
			EU342655	*Dendrobates*	*auratus*	P, C, L	TNHCFS4812	Panama	PA	Colon: Fort Sherman	189	Central America	J	-79.91540	9.28016
			AY364565	*Dendrobates*	*auratus*	P, L	TNHC62487	Panama	PA	Panama	n.d.	Central America	J	n.d.	n.d.
			EU342656	*Dendrobates*	*auratus*	P, L	TNHCFS4811	Panama	PA	Cocle: El Cope, Parque Nacional General de Division 'Omar Torrijos Herrera	782	Central America	J	-80.592	8.667
			DQ502030	*Dendrobates*	*auratus*	P, L	CWM 17698(A)	Panama	PA	Bocas del Toro: North side Isla Pastores	5	Central America	J	-82.3333	9.2333
			AY843581	*Dendrobates*	*auratus*	P, L	USNM 31318	Panama	PA	Bocas del Toro	n.d.	Central America	J	n.d.	n.d.
			EU342657	*Dendrobates*	*auratus*	P, C, L	MVZ149723	Costa Rica	CR	Limon: 1 km NW Cahuita, Atlantic cost	54	Central America	J	-82.85	9.7333
DQ371303		DQ371314		*Dendrobates*	*mysteriosus*	P, L	none	Peru	PE	Cajamarca: near Santa Rosa	900	Central Oriental Andes	G	-78.6833	-5.3667
			EU342665	*Dendrobates*	*captivus*	P, C, L	QCAZ27442	Ecuador	EC	Zamora Chinchipe: near Panguitza	870	Central Oriental Andes	G	-78.8125	-3.8986
			EU342666	*Dendrobates*	*captivus*	P, L	QCAZ27443	Ecuador	EC	Zamora Chinchipe: near Panguitza	870	Central Oriental Andes	G	-78.8125	-3.8986
			EU342667	*Dendrobates*	*virolinensis*	P, C, L	TNHCFS4950	Colombia	CO	Santander: Virolin, Costilla de Fara	1767	North Oriental Andes	E	-73.196667	6.113306
			EU342668	*Dendrobates*	*bombetes*	P, C, L	TNHCFS4892	Colombia	CO	Valle del Cauca: Buga, Buga-Buenaventura road	1610	North Occidental Andes	F	-76.435	3.876
			EU342669	*Dendrobates*	*bombetes*	P, L	TNHCFS4975	Colombia	CO	Quindio: Barbas, Finlandia, Hacienda Lusitania	1958	North Occidental Andes	F	-75.63525	4.689778
			DQ502106	*Dendrobates*	*fulguritus*	P, C, L	MHNUC 340	Colombia	CO	Choco: Bahia Solano, Sierra Mecana	260	Choco	I	-77.35555	6.25694
		AF124116		*Dendrobates*	*fulguritus*	P, L	none	Panama	PA	San Blas	n.d.	Choco	I	n.d.	n.d.
			DQ502168	*Dendrobates*	*minutus*	P, C, L	KRL 790	Panama	PA	Cocle: El Cope, Parque Nacional General de Division 'Omar Torrijos Herrera	782	Choco/Central America	IJ	-80.592	8.667
			EU342670	*Dendrobates*	*aff. minutus*	P, C, L	TNHCFS4943	Colombia	CO	Choco: Quibdo, La Troje	50	Choco	I	-76.591	5.728
			DQ283042	*Dendrobates*	*claudiae*	P, L	USNM-FS 59980	Panama	PA	Bocas del Toro: S end of Isla Popa, 1 km E Sumwood Channel	6	Central America	J	-82.1167	9.1833
			EU342671	*Dendrobates*	*claudiae*	P, C, L	KS9	Panama	PA	Bocas del Toro: Isla Colon, Bocas del Drago	11	Central America	J	-82.32038	9.42479

Table S3 (Cont) Taxa used to infer the phylogeny and the chronogram of the poison frogs. 'Use' identifies which samples were used for the phylogeny (P), chronogram (C), and Langrage (L) analyses

12S	16S beginning	16S end	12S-16S	Genera	Species	Use	Museum	Country	Code	Locality	altitude (m)	Area	Code	Long	Lat
AF482794		AF482779		*Dendrobates*	*biolat*	P, L	none	Peru	PE	Madre de Dios: Tambopata	310	Central Oriental Andes	G	-69.183	-12.733
AF482793		AF482778		*Dendrobates*	*lamasi*	P, L	none	Peru	PE	Hanuco: Tingo Maria	705	Central Oriental Andes	G	-75.9833	-9.30
			EU342672	*Dendrobates*	*imitator*	P, C, L	KS13	Peru	PE	San Martin: Tarapoto	550	Central Oriental Andes	G	-76.29034	-6.43066
DQ371306		DQ371317		*Dendrobates*	*flavovittatus*	P, L	none	Peru	PE	Loreto	n.d.	Amazon	C	n.d.	n.d.
			EU342673	*Dendrobates*	*vanzolinii*	P, C, L	LSUMZ13696	Brazil	BR	Acre: near 5 km N Porto Walker, inland from the Rio Jurua	200	Amazon	C	-72.776972	-8.258667
			EU342674	*Dendrobates*	*vanzolinii*	P, L	LSUMZ13697	Brazil	BR	Acre: near 5 km N Porto Walker, inland from the Rio Jurua	200	Amazon	C	-72.776972	-8.258667
			EU342682	*Dendrobates*	*fantasticus*	P, C, L	KS31	Peru	PE	San Martin: Tarapoto	540	Central Oriental Andes	G	-76.29034	-6.43066
			EU342683	*Dendrobates*	*fantasticus*	P, L	KS29	Peru	PE	San Martin: Cainarachi Valley	350	Central Oriental Andes	G	-76.31758	-6.45043
DQ371305		DQ371316		*Dendrobates*	*uakarii*	P, L	none	Peru	PE	Loreto	n.d.	Amazon	C	n.d.	n.d.
			EU342684	*Dendrobates*	*ventrimaculatus*	P, C, L	LSUMZ13755	Brazil	BR	Acre: near 5 km N Porto Walker, inland from the Rio Jurua	200	Amazon	C	-72.776972	-8.258667
			EU342685	*Dendrobates*	*ventrimaculatus*	P, L	LSUMZ13770	Brazil	BR	Acre: near 5 km N Porto Walker, inland from the Rio Jurua	200	Amazon	C	-72.776972	-8.258667
			AY364567	*Dendrobates*	*reticulatus*	P, C, L	TNHC61143	Peru	PE	Loreto	n.d.	Amazon	C	n.d.	n.d.
			EU342686	*Dendrobates*	*reticulatus*	P, L	KS41	Peru	PE	Loreto: Puerto Almendras, 20 km WSW of Iquitos	100	Amazon	C	-73.32	-4.1
			AY364568	*Dendrobates*	*sp*	P, C, L	QCAZ16558	Ecuador	EC	Pastaza: Nuevo Corrientes	294	Amazon	C	-76.776667	-2.0075
			EU342687	*Dendrobates*	*sp*	P, L	QCAZ25711	Ecuador	EC	Pastaza: Kapawi Lodge	239	Amazon	C	-76.830812	-2.540804
			EU342688	*Dendrobates*	*duellmani*	P, C, L	QCAZ16560	Ecuador	EC	Francisco de Orellana: Parque Nacional Yasuni-Estacion PUCE	230	Amazon	C	-76.50	-0.633
			AY364566	*Dendrobates*	*duellmani*	P, L	QCAZ16561	Ecuador	EC	Francisco de Orellana: Parque Nacional Yasuni-Estacion PUCE	230	Amazon	C	-76.50	-0.633
			EU342675	*Dendrobates*	*ventrimaculatus*	P, C, L	LSUMZ15394	Brazil	BR	Amazonas: Rio Ituxi at the Madeireira Scheffer; left bank	110	Amazon	C	-65.716556	-8.479389

Table S3 (Cont) Taxa used to infer the phylogeny and the chronogram of the poison frogs. 'Use' identifies which samples were used for the phylogeny (P), chronogram (C), and Langrage (L) analyses

12S	16S beginning	16S end	12S-16S	Genera	Species	Use	Museum	Country	Code	Locality	altitude (m)	Area	Code	Long	Lat
			EU342676	*Dendrobates*	*ventrimaculatus*	P, L	LSUMZ16969	Brazil	BR	Amazonas: Castanho, ca. 40 km S Manaus at km 12 on road to Autazes	40	Amazon	C	-60.455111	-3.619556
			EU342677	*Dendrobates*	*ventrimaculatus*	P, C, L	LSUMZ16925	Brazil	BR	Amazonas: Castanho, ca. 40 km S Manaus at km 12 on road to Autazes	40	Amazon	C	-60.455111	-3.619556
			EU342678	*Dendrobates*	*ventrimaculatus*	P, C, L	KS42	Peru	PE	Loreto: Tahuayo	140	Amazon	C	-73.10457	-4.18707
			DQ502069	*Dendrobates*	*ventrimaculatus*	P, L	OMNH 34091	Ecuador	EC	Sucumbios, Estacion Cientifica de Universidad Catolica near Reserva Faunistica Cuyabeno	220	Amazon	C	-76.16667	0
			AY364570	*Dendrobates*	*ventrimaculatus*	P, L	QCAZ16566	Ecuador	EC	Francisco de Orellana: Parque Nacional Yasuni-Estacion PUCE	230	Amazon	C	-76.50	-0.633
			EU342679	*Dendrobates*	*variabilis*	P, C, L	KS12	Peru	PE	San Martin: Tarapoto	425	Central Oriental Andes	G	-76.3656	-6.5014
			EU342680	*Dendrobates*	*variabilis*	P, L	KS27	Peru	PE	San Martin: Tarapoto	425	Central Oriental Andes	G	-76.3656	-6.5014
			DQ502266	*Dendrobates*	*ventrimaculatus*	P, C, L	JDL 24489	Colombia	CO	Amazonas: Leticia, Km 11 (Leticia-Tarapaca)	80	Amazon	C	-69.941	-4.215
DQ163087		DQ163076		*Dendrobates*	*ventrimaculatus*	P, L	BPN737	French Guiana	FG	Departement de la Guyane: Kaw	71	Guiana Shield	B	-52.0333	4.4833
			EU342681	*Dendrobates*	*ventrimaculatus*	P, C, L	TNHC64418	French Guiana	FG	Departement de la Guyane	n.d.	Guiana Shield	B	n.d.	n.d.
DQ163083		DQ163075		*Dendrobates*	*ventrimaculatus*	P, L	BPN664	French Guiana	FG	Departement de la Guyane: Pic Matecho lake	590	Guiana Shield	B	-53.033	3.750
DQ163084		DQ163081		*Dendrobates*	*ventrimaculatus*	P, L	BPN762	French Guiana	FG	Departement de la Guyane: Saul	230	Guiana Shield	B	-53.2	3.6167
DQ371302		DQ371313		*Dendrobates*	*ventrimaculatus*	P, L	none	French Guiana	FG	Departement de la Guyane	n.d.	Guiana Shield	B	n.d.	n.d.
DQ163088		DQ163080		*Dendrobates*	*ventrimaculatus*	P, L	BPN744	French Guiana	FG	Departement de la Guyane: Maripa	26	Guiana Shield	B	-51.8833	3.80
