# Supplementary material for: Amazonian Amphibian Diversity Is Primarily Derived from Late Miocene Andean Lineages
Source: PLoS Biol. 2009 Mar 10;7(3):e1000056. doi: 10.1371/journal.pbio.1000056 (PMC2653552; doi:10.1371/journal.pbio.1000056)
Supplement: Supplementary file 6 [file pbio.1000056.st004.pdf]

Table S4. Time constraints used to infer the age of the root of the poison frog tree.

Figure S4 code	Node	Time Interval (MYA)	Event	Stages age ICS (MYA)	Evidence
1	#173	306.1-332.3	Diapsida (Aves/Reptilia) and Synapsida (Mammalia) divergence	Visean-Moscovian (345.3-306.5)	Amniote fossil record and avian molecular clocks [119-121]
2	#169	245.0	Anura and Caudata divergence	Induan-Olenekian (245.0-251.0)	†*Triadobatrachus massinoti* fossil date estimate [122]
3	#158	65.5	Pelodytoidea	Maastrichtian (65.5-70.6)	†*Eopelobates* sp. fossil date estimate [123,124]
4	#150	65.5	Hyloidea	Santonian-Maastrichtian (65.5-85.8)	†*Baurubatrachus pricei* fossil date estimate [125]
5	#162	150.8	Stem Rhinophrynidae	Kimmeridgian (150.8-155.7)	†*Rhadinosteus parvus* fossil date estimate [126]
6	#160	86.0	*Hymenochirus* and *Pipa* divergence	-	Final separation of Africa and South America [127,128]
7	#90	164.7	Stem Discoglossoidea	Bathonian (164.7-167.7)	†*Eodiscoglossus oxoniensis* fossil date estimate [129,130]
**Node constraint sets**					
All constraints			#173, #169, #158, #150, #150, #162, #160, #90		
Constraints within Anura			#169, #158, #150, #150, #162, #160, #90		
Fossil Anuran constraints			#169, #158, #150, #150, #162, #90		
