# Supplementary material for: Amazonian Amphibian Diversity Is Primarily Derived from Late Miocene Andean Lineages
Source: PLoS Biol. 2009 Mar 10;7(3):e1000056. doi: 10.1371/journal.pbio.1000056 (PMC2653552; doi:10.1371/journal.pbio.1000056)
Supplement: Supplementary file 7 [file pbio.1000056.st005.pdf]

Table S5. Poison frog time constraints (node numbers are indicated in the Figure S5 A-D).

Figure S5 code	Node	Time Interval (MYA)	Rationale
A	#279, #280, #281, #295, #312, #348, #349, #351, #352, #355, #356, #361, #362, #364, #365, #418, #421	2.4-15.0	These nodes were given this time constraint because their ancestors dispersed from South America to Central America as indicated in the section on determination of divergence times.
B	#298, #350, #360, #363, #416	4.0	The nodes correspond to second or third wave of dispersals from South to Central America of members of the *Allobates talamancae* (#416), *Colostethus inguinalis* (#350), *Dendrobates auratus* (#298), *Silverstoneia flotator* (#360) and *Silverstoneia nubicola* (#363) clades. All of these clades are widespread in Central America (Western and Eastern Panamá) and most Chocó (Northwestern Colombia and Ecuador). Their distributional patterns were revealed by ancestral area reconstruction and it is consistent with a rapid dispersal into and across South America after the uplift of the Isthmus of Panamá. The upper time boundary of 4.0 MYA corresponds to the oldest estimate of the establishment of the Panamá Land Bridge (PLB) in the Pliocene [131].
C	#311	8.0	This node corresponds to the most recent common ancestor of the sister species *Phyllobates lugubris* (Atlantic lowland tropical rainforest) and *P. vitattus* (Pacific lowland tropical rainforest) before the rise of the Talamanca Cordillera in Central America. The vicariant event was revealed by the ancestral area reconstruction analyses. The upper time boundary of 8.0 MYA corresponds to the oldest estimate of the beginning of the rise of the Talamanca Cordillera in the late Miocene [11].
D	#240, #244	5.0-9.0	Both nodes correspond to the most recent common ancestors of the sister taxa distributed in both North Oriental and Occidental Andes: *Hyloxalus delatorreae-H. pulchellus* (#240) and *H. vertebralis* clade (#244). The dispersal event was revelead by the ancestral area reconstruction analyses. The dispersals between both ranges of the Andean Cordillera were most likely to have occurred at the final uplift of the Northern Andes.

Table S5 (Cont). Poison frog time constraints (node numbers are indicated in the Figure S5 A-D).

Figure S5 code	Node	Time Interval (MYA)	Rationale
E	#261, #436	2.7-6.5	These nodes correspond to the most recent common ancestors of the *Rheobates palmatus* (#436) and *Hyloxalus subpuctatus* (#261) clades endemic to the North Oriental Andes of Colombia. The diversification and origin of these clades are associated to the uplift of the youngest branch of Andes in Colombia, which started in the Pliocene [27,132].
F	#259	5.0-15.0	This node corresponds to the most recent common ancestor of the sister species *Hyloxalus bocagei* and *H. sauli*. *Hyloxalus bocagei* is endemic to the eastern slopes of the North Oriental Andes of Ecuador and *H. sauli* is a lowland western Amazonian species. The vicariant event and isolation of *Hyloxalus bocagei* was most likely associated to the uplift of the Andes in Ecuador during the late Miocene.
G	#446	57.5-31.4	This node corresponds to the most recent common ancestor of the extant taxa of poison frogs (Dendrobatidae). The dating of this node came from the 95% CI limits of our Amphibian tree chronogram.

**Constraint Set**	***n***	**A**	**B**	**C**	**D**	**E**	**F**	**G**
**1**	48	UL	U	U	UL	UL	UL	UL
	Nodes Excluded	-	-	-	-	-	-	-
**2**	29	U	-	U	U	U	U	UL
	Nodes Excluded	-	All	-	-	-	-	-
**3**	23	U	-	U	U	-	U	UL
	Nodes Excluded	-	All	-	-	All	-	-

Node constrained for upper (U) and lower (L) time boundaries.
