# Supplementary material for: Amazonian Amphibian Diversity Is Primarily Derived from Late Miocene Andean Lineages
Source: PLoS Biol. 2009 Mar 10;7(3):e1000056. doi: 10.1371/journal.pbio.1000056 (PMC2653552; doi:10.1371/journal.pbio.1000056)
Supplement: Supplementary file 8 [file pbio.1000056.st006.pdf]

Table S6. Published evidence supporting the paleogeographic constraints used in inferring the poison frog chronogram.

Event	Time (MYA)	Constraint concordance	Evidence
Formation of the Panamá Land Bridge (PLB)	3.0	A and B	Paleogeological evidence [133]
Characteristic ichnofauna from the northwestern coast of South America	3.2-3.6	A and B	Fossil ichnofauna of northwestern coast of Ecuador [134,135]
Final closure of the Isthmus of Panamá	2.8-3.1	A and B	Paleogeological and stratigraphic dating [11]
Final closure of the Isthmus of Panamá	2.4-2.8	A and B	Characteristic Paleoceanographic patterns in the Caribbean and North Atlantic Ocean [136]
Major biotic interchange of terrestrial animals between North and South America	2.47	A and B	Neotropical mammal fossil record [137]
Major biotic interchange of terrestrial animals between North and South America	2.5-3.6	A and B	Paleomagnetism of fossiliferous sediments [138,139]
Major biotic interchange of terrestrial animals between North and South America	3.3-4.0	A and B	Paleomagnetism of fossiliferous sediments (Inchasi, Bolivia) [131]
Upper boundary date before the formation of the Central American Archipelago	15.0	A	Biostratigraphic dating using planktic foraminifera and calcareous nanoplankton [11,37,140]
Gradual uplift of the Panamá Land Bridge	Middle Miocene-Pliocene	A	Central and South American tonnoidean gastropods fossil record [141]
Pacific lowland of Ecuador and Colombia was permanently covered by the sea	Paleocene to Pliocene	A	Paleogegraphic and stratigraphic evidence of the Pacific coast of northern South America [142,143]
Corridor between Chocó and Central America	Pliocene	A and B	Terrestrial species similarities [144]
Divergence of Central and South American clades before the formation of Panamá Land bridge	6.0-18.0	A	*Lachesis* clade (bushmaster snakes) molecular divergence [39]

Table S6 (Cont). Published evidence supporting the paleogeographic constraints used to infer the poison frog chronogram.

Event	Time (MYA)	Constraint concordance	Evidence Reference
Dispersal and colonization of South America before the formation of Panamá Land bridge	15.0	A	Neotropical widespread *Symphonia globulifera* tree phylogeographic structure [145]
Divergence of Central and South American lineages before the formation of Panamá Land bridge	6.0-10.0	A	Divergence of Central and South American lineages of *Physalaemus pustulosus* [146]
Divergence of Central and South American lineages before the formation of Panamá Land bridge	2.9-5.3	A and B	Divergence of Central and South American lineages of harlequin beetle-riding pseudoscorpion, *Cordylochernes scorpioides* [147]
Divergence of Central and South American lineages before the formation of Panamá Land bridge	3.1-15.0	A and B	Sea urchin (Arcidae) pair of species divergence from tropical western Atlantic and eastern Pacific oceans of Panamá [148]
Multiple dispersals of freshwater fishes lineages from Chocó (northwestern Colombia) to Central America	Late Miocene-Pleistocene	A and B	Three distinct waves of invasion into Central America from putative northwestern South America source populations of freshwater fishes (*Roeboides*, *Hypopomus*, *Pimelodella*) [12]
Uplift of Talamanca Cordillera	5.0-8.0	C	Paleogeological evidence [11,149]
Uplift of Talamanca Cordillera	3.5-5.5	C	Subduction of the Cocos Ridge plate in southeast Costa Rica accelerated the rise of the Talamanca Cordillera [150]
Dispersal into South America after the uplift of the Isthmus of Panamá	1.1	B	*Crotalus durissus* phylogeographic analysis [151]
Dispersal into South America after the uplift of the Isthmus of Panamá	1.47-1.93	B	Rapid diversification of *Lupinus* legume genus in Andes [152]
Divergence of Central and South American lineages of *Phyllobates* poison frogs before the formation of Panamá Land bridge	5.0-5.5	A	Dating inferred from immunological distance between *Phyllobates terribilis* and *P. vittatus* [153]

Table S6 (Cont). Published evidence supporting the paleogeographic constraints used in inferring the poison frog chronogram.

Event	Time (MYA)	Constraint concordance	Evidence Reference
Final uplift of Northern Oriental Andes Cordillera	2.7-5.0	D, E, and F	Paleobotanical, paleoclimatological, paleogeological, geomorphological, and landscape developmental data timing of the Cenozoic history of the Northern and Central Andes. [27,154]
Rise of Northern Oriental Andes Cordillera and the Venezuelan Highlands	Late Miocene	F	Sedimentary record, paleogeography of the western Venezuelan Basins, and Caribbean neotectonics [90,155,156]
Rise of Northern Oriental Andes Cordillera	6.0-9.0	F	Miocene history of the Ecuadorian Andes reveled by using stratigraphical and fission-track dating approachs. [85]
Uplift of the Central Oriental Andes	11.0-12.9	F	Mammalian and fish fossil records, sendimentary environments dating. [93,157]
Diversification as consequence of the uplift of the Andes	5.0-2.0	E	Time estimated for cis- and trans-Andean avian taxa diversification. [158,159]
Magdalena drainage system formation during the uplift of the North Oriental Andes Cordillera	9.0-12.9	D and F	Rise of the North Oriental Andes Cordillera of Colombia from statigraphic, sedimentary, and aquatic biota fossil record. [91,93,160]
Rise of the Central Andes	8.0-15.0	D, E, and F	Quechua I and II tectonic phases of orogenic formation of the Andes from palaeogeological evidence [161]
Rise of the Central Andes	12.5-20.0	D, E, and F	Quechua I tectonic phases of orogenic formation of the Andes from palaeogeological evidence [162-164] [165]
