# Supplementary material for: Amazonian Amphibian Diversity Is Primarily Derived from Late Miocene Andean Lineages
Source: PLoS Biol. 2009 Mar 10;7(3):e1000056. doi: 10.1371/journal.pbio.1000056 (PMC2653552; doi:10.1371/journal.pbio.1000056)
Supplement: Supplementary file 9 [file pbio.1000056.st007.pdf]

Table S7. Amphibian tree estimated divergence times for MULTIDIVTIME (nodes from Supplementary Figure 4)

		All Constraints (MYA) n=7				Constraints within Anura (MYA) n=6				Fossil Anuran constraints (MYA) n=5			
node	Event	mean	s.d.	L 95CI	U 95CI	mean	s.d.	L 95CI	U 95CI	mean	s.d.	L 95CI	U 95CI
88		67.509	18.785	35.546	107.816	64.846	18.219	33.623	103.836	65.182	18.347	34.196	104.426
89	stem Alytidae	209.809	19.062	171.961	246.483	202.794	19.832	165.383	243.444	203.167	20.009	165.462	244.170
90	Bombinatoridae and Alytidae split	231.846	18.377	194.918	267.475	224.291	19.725	187.642	264.655	224.672	19.967	186.891	265.031
91		41.476	7.182	29.064	57.144	40.106	7.032	28.026	55.754	40.299	7.129	28.279	55.910
92		59.717	8.856	44.129	78.662	57.767	8.739	42.566	76.950	57.964	8.917	42.824	77.378
93		71.811	9.764	54.384	92.798	69.489	9.596	53.196	90.617	69.704	9.811	53.225	91.003
94		28.713	5.463	19.336	40.721	27.744	5.292	18.916	39.375	27.838	5.391	18.939	39.924
95		16.307	3.585	10.383	24.306	15.782	3.495	10.125	23.589	15.810	3.512	10.137	23.589
96		30.748	5.559	21.287	42.655	29.705	5.459	20.517	41.810	29.797	5.527	20.608	41.939
97		34.574	6.061	24.118	47.661	33.411	5.910	23.449	46.575	33.531	6.018	23.441	46.578
98		37.945	6.608	26.379	51.905	36.683	6.471	25.848	51.165	36.832	6.585	25.596	51.223
99	node Ranidae	50.465	7.746	36.835	67.063	48.790	7.591	35.922	65.427	48.984	7.770	35.853	65.867
100		31.951	5.809	21.995	44.610	30.985	5.777	21.224	43.878	31.110	5.854	21.204	44.076
101		43.539	7.067	31.117	58.685	42.180	6.945	30.398	57.485	42.332	7.065	30.401	57.967
102		49.819	7.664	36.416	66.745	48.226	7.508	35.404	64.634	48.393	7.629	35.459	65.048
103		50.874	7.957	36.925	67.897	49.252	7.750	36.212	66.293	49.417	7.896	36.100	66.923
104		66.125	9.172	50.005	85.530	63.988	9.043	48.660	83.787	64.176	9.225	48.517	84.314
105		44.773	7.300	32.055	60.676	43.230	7.108	31.186	58.973	43.377	7.213	30.941	58.987
106		69.535	9.499	52.755	89.746	67.274	9.367	51.526	87.602	67.471	9.566	51.414	88.400
107		72.645	9.786	55.463	93.379	70.288	9.653	54.012	91.447	70.497	9.861	53.789	92.027
108		74.921	9.997	57.140	96.402	72.503	9.845	55.764	94.172	72.715	10.067	55.647	94.660
109	node Natatanura	84.425	10.999	64.853	107.683	81.721	10.840	63.345	105.226	81.945	11.135	63.010	105.707
110		57.722	9.175	41.228	77.281	55.945	8.856	40.500	75.467	56.018	9.087	40.456	75.659
111		67.015	10.077	48.994	88.326	64.912	9.846	48.068	86.362	65.046	10.066	47.541	86.828
112		82.805	10.947	63.409	105.818	80.245	10.801	61.430	103.388	80.458	11.144	61.205	104.459
113		36.923	7.346	24.246	53.058	35.723	7.113	23.749	51.531	35.763	7.199	23.493	51.546
114		49.727	8.551	34.890	68.312	48.153	8.398	33.816	66.577	48.217	8.565	33.568	66.848
115		66.379	9.853	48.843	87.265	64.327	9.695	47.866	85.499	64.402	9.895	47.410	86.007
116	stem Microhylidae	90.463	11.387	70.581	114.354	87.656	11.232	68.043	111.958	87.881	11.550	67.823	112.865
117	stem Natatanura	98.225	11.909	76.995	122.924	95.136	11.729	75.276	120.469	95.397	12.071	74.626	121.332

Table S7 (Cont). Amphibian tree estimated divergence times for MULTIDIVTIME (nodes from Supplementary Figure 4)

		All Constraints (MYA) n=7				Constraints within Anura (MYA) n=6				Fossil Anuran constraints (MYA) n=5			
node	Event	mean	s.d.	L 95CI	U 95CI	mean	s.d.	L 95CI	U 95CI	mean	s.d.	L 95CI	U 95CI
118		54.481	8.806	39.391	73.037	52.888	8.534	38.642	71.765	53.221	8.757	38.687	72.440
119		73.218	10.029	56.216	94.772	71.096	9.831	55.237	92.591	71.448	10.092	55.147	93.717
120		42.921	7.468	30.278	59.112	41.686	7.273	29.436	57.781	41.884	7.388	29.638	58.475
121		49.487	8.324	35.285	67.704	48.040	8.156	34.324	66.637	48.229	8.229	34.536	66.363
122		67.157	9.559	50.795	87.752	65.198	9.399	49.872	86.438	65.495	9.547	49.774	86.582
123		20.020	3.993	13.308	28.976	19.528	3.963	12.999	28.349	19.576	4.046	12.848	28.592
124		22.047	4.302	14.810	31.706	21.481	4.236	14.495	30.882	21.533	4.338	14.315	31.204
125		28.089	5.116	19.380	39.520	27.280	5.022	18.996	38.450	27.367	5.143	18.790	38.689
126		40.801	6.706	29.403	55.377	39.658	6.540	28.663	54.191	39.854	6.722	28.561	54.606
127		46.756	7.289	34.222	62.412	45.488	7.098	33.596	61.219	45.735	7.317	33.569	61.775
128	node Bufonidae	60.526	8.559	45.786	79.213	58.824	8.358	44.974	77.521	59.179	8.549	45.297	78.060
129		70.674	9.350	54.784	90.820	68.589	9.135	53.823	88.539	68.964	9.403	53.714	89.708
130	stem *Dendrobates* and *Phyllobates*	28.100	5.148	19.238	39.410	27.250	4.959	19.026	38.469	27.379	5.057	19.018	38.638
131		31.648	5.569	22.300	43.964	30.711	5.396	21.805	42.682	30.855	5.502	21.756	43.051
132		36.958	6.128	26.631	50.348	35.824	5.915	25.982	48.924	35.994	6.050	25.990	49.434
**133**	**Dendrobatidae**	**43.713**	**6.902**	**32.001**	**58.702**	**42.401**	**6.666**	**31.373**	**57.485**	**42.619**	**6.839**	**31.382**	**57.883**
134		45.596	7.248	32.983	61.119	44.211	7.106	32.308	59.785	44.470	7.314	32.431	60.839
135		52.074	7.632	39.038	68.608	50.535	7.487	38.093	66.933	50.808	7.683	38.129	67.988
136		48.648	7.409	35.917	64.758	47.144	7.286	34.905	63.171	47.437	7.497	34.945	63.954
137		56.952	8.015	43.202	74.347	55.230	7.839	42.490	72.569	55.553	8.054	42.364	73.097
138		60.819	8.369	46.593	79.036	58.965	8.170	45.643	76.899	59.303	8.407	45.697	77.753
139		15.577	3.318	10.172	23.068	15.110	3.176	9.795	22.141	15.195	3.247	9.847	22.528
140		46.557	7.317	34.031	62.556	45.065	7.067	33.051	60.441	45.331	7.268	32.993	61.259
141		52.607	7.683	39.474	69.161	50.953	7.434	38.576	67.332	51.249	7.649	38.557	68.036
142		48.572	7.621	35.561	65.477	47.085	7.352	34.819	63.108	47.357	7.559	34.759	64.147
143		60.949	8.464	46.485	79.250	59.100	8.194	45.741	77.448	59.460	8.468	45.582	78.003
144		65.257	8.820	50.254	84.365	63.277	8.617	49.539	82.338	63.641	8.853	49.219	82.961
145		68.190	9.070	52.909	87.939	66.137	8.837	51.888	85.766	66.494	9.115	51.712	86.772
**146**	**stem Dendrobatidae**	**71.075**	**9.365**	**55.260**	**91.168**	**68.961**	**9.149**	**54.187**	**89.082**	**69.341**	**9.429**	**54.126**	**89.921**
147		74.111	9.648	57.812	94.937	71.927	9.434	56.738	92.463	72.319	9.717	56.785	93.666

Table S7 (Cont). Amphibian tree estimated divergence times for MULTIDIVTIME (nodes from Supplementary Figure 4)

		All Constraints (MYA) n=7				Constraints within Anura (MYA) n=6				Fossil Anuran constraints (MYA) n=5			
node	Event	mean	s.d.	L 95CI	U 95CI	mean	s.d.	L 95CI	U 95CI	mean	s.d.	L 95CI	U 95CI
148		77.442	10.048	60.349	99.001	75.194	9.802	59.474	96.655	75.568	10.086	59.394	97.736
149		82.476	10.458	64.542	104.722	80.052	10.231	63.711	102.586	80.463	10.502	63.840	103.677
150	node Hyloidea	86.313	10.744	68.021	109.213	83.779	10.507	67.192	106.620	84.196	10.812	67.187	107.798
151	stem Hyloidea	127.219	13.911	102.251	156.087	123.201	13.790	99.585	152.483	123.622	14.265	98.770	154.196
152		107.499	13.561	83.331	135.964	103.875	13.270	80.752	132.013	104.238	13.738	80.194	133.711
153		138.611	14.658	111.787	168.882	134.157	14.522	108.954	164.845	134.636	14.998	108.248	166.193
154	node Neobatrachia (without Sooglosidae)	147.093	15.181	119.316	178.197	142.292	15.078	115.829	174.087	142.745	15.551	115.294	175.012
155		36.485	11.689	18.781	63.987	35.075	11.171	18.221	61.411	35.277	11.390	18.377	62.232
156		111.703	17.099	80.054	146.374	107.847	16.590	77.197	142.511	108.067	16.816	76.810	142.949
157		147.646	17.655	113.730	183.265	142.743	17.490	110.425	178.530	143.148	17.803	110.512	180.450
158	node Scaphiopodidae	165.373	17.703	131.755	200.544	159.950	17.914	126.669	196.866	160.271	18.138	127.085	197.634
159		210.146	17.567	175.766	245.081	203.360	18.414	169.311	241.604	203.761	18.835	168.827	242.636
160		144.489	18.889	107.963	181.960	139.439	18.847	102.913	177.386	139.734	19.077	103.860	178.398
161	stem Pipidae	167.667	18.493	132.280	203.643	161.943	18.895	126.218	200.013	162.207	19.258	126.678	201.285
162	Rhinophrynidae and Pipidae split	210.342	17.807	175.561	244.958	203.328	18.893	168.368	242.134	203.582	19.286	168.060	243.475
163		236.990	17.702	201.877	271.291	229.278	19.339	193.374	268.431	229.640	19.614	192.920	269.835
164		245.918	17.594	210.684	279.761	237.890	19.562	201.030	277.725	238.254	19.822	200.708	278.387
165	stem Leiopelmatidae	237.811	19.097	199.887	273.942	229.935	20.825	190.688	272.625	230.068	21.114	190.017	272.016
166	node Anura	268.058	17.043	234.011	300.306	259.401	19.813	221.274	299.231	259.583	20.093	221.438	300.024
167	*Hynobius* and *Ambystoma* split	208.854	24.589	156.215	253.321	201.483	26.141	149.187	251.355	201.323	26.192	147.923	251.067
168		247.841	22.400	201.544	288.896	239.105	24.682	188.888	287.158	239.031	24.873	189.133	286.048
169	Caudata and Anura split	337.433	14.551	310.002	366.697	327.171	19.770	290.076	367.169	327.124	19.884	289.059	367.335
170		186.994	23.974	137.243	231.694	178.839	25.363	127.539	226.378	178.917	25.569	128.634	228.212
171		244.142	20.540	201.527	281.651	234.398	23.326	186.367	278.950	234.340	23.705	187.090	279.681
172	Root of Amphibia	349.338	13.777	323.764	377.036	338.833	19.444	302.382	378.358	338.740	19.663	301.618	379.045
173		317.169	7.320	306.495	331.095	300.772	22.200	256.939	344.520	300.710	22.572	257.068	345.475
174		355.434	13.030	331.720	382.379	344.478	19.312	308.064	384.034	344.371	19.467	307.174	383.811
