# Supplementary material for: Amazonian Amphibian Diversity Is Primarily Derived from Late Miocene Andean Lineages
Source: PLoS Biol. 2009 Mar 10;7(3):e1000056. doi: 10.1371/journal.pbio.1000056 (PMC2653552; doi:10.1371/journal.pbio.1000056)
Supplement: Supplementary file 10 [file pbio.1000056.st008.pdf]

Table S8. Poison frog tree estimated divergence times, events (vicariance and dispersals), Lagrange ancestral area reconstructions, and BayesTraits probability of Amazon Basin area at the chronogram node (node numbers form Supplementary Figure S5 A-D).

Entire Phylogeny LaGrange				Chrono. LaGrange			Btrait	Time Constratins Set 1				Time Constratins Set 2				Time Constratins Set 3			
Node	Event	split	p	Node	split	p	p	mean	s.d.	L 95CI	U 95CI	mean	s.d.	L 95CI	U 95CI	mean	s.d.	L 95CI	U 95CI
4		[D|D]	0.985																
5	D → E	[E|D]<br>[D|D]	0.475<br>0.387	441	[E|D]<br>[D|D]	0.513<br>0.284	0.065	10.660	2.891	5.955	17.245	9.998	2.750	5.585	16.177	9.264	2.510	5.233	15.117
6		[D|D]<br>[D|DE]	0.814<br>0.152	442	[D|D]<br>[D|DE]	0.703<br>0.225	0.062	16.960	3.664	10.782	24.975	15.750	3.532	10.043	23.853	14.572	3.209	9.379	22.055
10		[D|D]	0.995																
11		[D|D]	0.990																
14		[D|D]	0.998																
18		[D|D]	0.998																
19		[D|D]	0.995	439	[D|D]	0.987		8.300	2.365	4.474	13.648	7.694	2.218	4.170	12.873	7.134	2.056	3.960	11.848
20		[D|D]	0.996	440	[D|D]	0.952	0.021	11.101	2.790	6.508	17.465	10.255	2.659	6.060	16.330	9.484	2.415	5.641	14.912
21		[D|D]	0.983																
22		[D|D]	0.886	443	[D|D]	0.793	0.110	26.992	4.758	18.696	37.320	25.089	4.642	17.563	35.499	23.234	4.187	16.654	33.144
26		[E|E]	1.000																
27		[E|E]	1.000																
29		[E|E]	0.995																
31	D → E	[E|E]	0.924	436	[E|E]	0.880		3.135	1.047	1.474	5.584	2.905	1.013	1.353	5.333	2.730	0.996	1.261	5.121
34		[B|B]	0.993	434	[B|B]	0.980		3.770	1.316	1.762	6.865	3.453	1.204	1.659	6.323	3.196	1.112	1.536	5.841
37		[B|B]	0.999	431	[B|B]	0.997		3.962	1.264	1.996	6.859	3.670	1.198	1.863	6.475	3.406	1.108	1.715	6.008
40		[B|B]	0.999	429	[B|B]	0.999		1.094	0.487	0.349	2.235	1.021	0.455	0.321	2.083	0.954	0.428	0.299	1.977
43		[B|B]	1.000	428	[B|B]	0.999		5.518	1.560	3.001	9.020	5.130	1.499	2.795	8.612	4.770	1.394	2.592	8.007
44		[B|B]	0.999	430	[B|B]	0.998		6.294	1.711	3.514	10.119	5.859	1.657	3.300	9.710	5.449	1.533	3.038	9.031
45		[B|B]	0.996	432	[B|B]	0.993	0.002	7.187	1.894	4.064	11.395	6.688	1.833	3.821	10.894	6.215	1.699	3.573	10.134
48		[B|B]	0.994																
50		[B|B]	0.952																
54		[B|B]	1.000																
55		[B|B]	0.995	426	[B|B]	0.990		10.793	2.682	6.311	16.610	9.986	2.548	5.855	15.794	9.252	2.345	5.529	14.640
56	B → C	[B|B]	0.983	427	[B|B]	0.913	0.140	15.525	3.387	9.763	22.980	14.407	3.267	9.063	21.809	13.367	3.005	8.511	20.221
57		[B|B]	0.971	433	[B|B]	0.932	0.062	18.522	3.657	12.249	26.497	17.200	3.572	11.380	25.410	15.949	3.258	10.696	23.436
58		[B|B]	0.895	435	[B|B]	0.789	0.022	19.726	3.804	13.166	28.023	18.285	3.724	12.162	26.826	16.955	3.391	11.522	24.702
59	D|B	[D|B]	0.544	437	[D|B]	0.342	0.133	28.720	4.736	20.516	39.018	26.721	4.733	19.048	37.679	24.777	4.244	18.172	34.810
66		[I|I]	1.000																
67		[I|I]	0.999	419	[I|I]	0.998		2.670	0.787	1.428	4.548	2.106	0.773	0.941	3.915	1.964	0.719	0.915	3.680
69		[I|I]	0.998	420	[I|I]	0.994	0.001	3.575	0.946	2.152	5.876	2.809	0.960	1.391	5.087	2.624	0.887	1.326	4.708
72		[J|J]	1.000																

Table S8 (Cont). Poison frog tree estimated divergence times, events (vicariance and dispersals), Lagrange ancestral area reconstructions, and BayesTraits probability of Amazon Basin area at the chronogram node (node numbers form Supplementary Figure S5 A-D).

Entire Phylogeny LaGrange				Chrono. LaGrange			Btrait	Time Constratins Set 1				Time Constratins Set 2				Time Constratins Set 3			
Node	Event	split	p	Node	split	p	p	mean	s.d.	L 95CI	U 95CI	mean	s.d.	L 95CI	U 95CI	mean	s.d.	L 95CI	U 95CI
75	I|J	[I|J]	0.975	416	[I|J]	0.962	0.000	1.071	0.398	0.416	2.002	0.802	0.379	0.252	1.718	0.746	0.343	0.237	1.567
78		[J|I]	0.997																
81	J → I	[J|J]	1.000	415	[J|J]	1.000	0.000	0.916	0.382	0.286	1.808	0.691	0.342	0.188	1.514	0.645	0.322	0.171	1.416
82		[J|J]<br>[IJ|J]	0.568<br>0.430																
83	I → J	[IJ|J]	0.585	417	[IJ|J]<br>[J|J]	0.765<br>0.224	0.000	2.153	0.586	1.286	3.591	1.610	0.624	0.708	3.136	1.503	0.568	0.684	2.868
84	I → J	[J|IJ]	0.879	418	[J|IJ]<br>[J|J]	0.731<br>0.254	0.001	3.172	0.702	2.426	5.058	2.347	0.848	1.109	4.405	2.190	0.780	1.075	4.081
85		[I|IJ]<br>[I|I]	0.564<br>0.319	421	[I|IJ]<br>[I|I]<br>[I|J]	0.416<br>0.324<br>0.249	0.000	4.429	1.041	2.967	7.018	3.440	1.133	1.758	6.153	3.210	1.042	1.674	5.678
86	E → I	[I|I]<br>[EI|I]	0.518<br>0.285	422	[I|I]<br>[EI|I]	0.432<br>0.351	0.069	13.485	2.818	8.913	19.922	11.486	2.906	6.836	18.045	10.705	2.681	6.540	16.864
89		[C|C]	1.000																
92		[C|C]	0.999																
96		[C|C]	1.000																
97		[C|C]	0.999																
98		[C|C]	0.966	412	[C|C]	0.969	0.989	8.303	2.151	4.846	13.161	7.448	2.046	4.202	12.099	6.954	1.908	4.047	11.383
99		[C|C]	0.951	413	[C|C]	0.953	0.977	15.059	3.201	9.772	22.215	13.564	3.146	8.427	20.791	12.647	2.906	8.039	19.390
105		[G|G]	1.000																
109		[G|G]	1.000																
110		[G|G]	1.000																
111		[G|G]	1.000																
112		[G|G]	1.000																
113		[G|G]	0.941	409	[G|G]	0.938		9.745	2.379	5.867	14.952	8.765	2.233	5.182	13.876	8.182	2.084	4.933	13.070
114	C → G	[G|G]	0.789	410	[G|G]<br>[CG|G]	0.738<br>0.161	0.025	10.622	2.512	6.519	16.133	9.550	2.379	5.731	14.962	8.914	2.209	5.486	14.193
119		[G|G]	1.000																
120		[G|G]	0.997	405	[G|G]	0.997	0.000	0.557	0.267	0.153	1.190	0.497	0.244	0.134	1.085	0.467	0.228	0.127	1.015
121	C → G	[C|G]	0.987	406	[C|G]	0.984		0.772	0.323	0.298	1.559	0.688	0.290	0.264	1.397	0.648	0.274	0.249	1.302
124		[C|C]	1.000																
127		[C|C]	1.000																
128		[C|C]	0.998	403	[C|C]	0.999		1.090	0.449	0.417	2.153	0.985	0.408	0.383	1.968	0.924	0.378	0.365	1.828
132		[C|C]	1.000																

Table S8 (Cont). Poison frog tree estimated divergence times, events (vicariance and dispersals), Lagrange ancestral area reconstructions, and BayesTraits probability of Amazon Basin area at the chronogram node (node numbers form Supplementary Figure S5 A-D).

Entire Phylogeny LaGrange				Chrono. LaGrange			Btrait	Time Constratins Set 1				Time Constratins Set 2				Time Constratins Set 3			
Node	Event	split	p	Node	split	p	p	mean	s.d.	L 95CI	U 95CI	mean	s.d.	L 95CI	U 95CI	mean	s.d.	L 95CI	U 95CI
133		[C|C]	0.999	401	[C|C]	1.000		0.493	0.241	0.123	1.060	0.443	0.222	0.111	0.975	0.416	0.209	0.104	0.910
137		[C|C]	1.000	398	[C|C]	1.000		0.586	0.258	0.192	1.207	0.524	0.238	0.170	1.091	0.495	0.225	0.161	1.039
138		[C|C]	1.000	399	[C|C]	1.000		1.265	0.465	0.575	2.388	1.135	0.428	0.515	2.174	1.070	0.399	0.483	2.017
143		[C|C]	0.999																
144	C → B	[B|C]	0.877	396	[B|C]	0.885	0.698	1.098	0.426	0.448	2.118	0.988	0.385	0.402	1.879	0.936	0.372	0.376	1.805
145		[C|C]<br>[C|BC]	0.790<br>0.208	397	[C|C]<br>[C|BC]	0.799<br>0.200	0.992	1.475	0.517	0.695	2.706	1.330	0.471	0.630	2.436	1.258	0.450	0.578	2.331
146		[C|C]	0.914	400	[C|C]	0.920	1.000	1.852	0.620	0.915	3.344	1.667	0.571	0.811	3.011	1.574	0.535	0.764	2.838
147		[C|C]	0.974	402	[C|C]	0.976	0.999	2.864	0.917	1.453	5.033	2.582	0.847	1.299	4.566	2.430	0.794	1.242	4.257
148		[C|C]	0.991	404	[C|C]	0.994	0.998	4.756	1.401	2.572	8.045	4.288	1.311	2.270	7.362	4.027	1.214	2.155	6.892
149		[CG|C]<br>[C|C]	0.604<br>0.352	407	[C|C]<br>[CG|C]	0.506<br>0.460	0.983	5.432	1.557	2.969	9.057	4.895	1.468	2.621	8.328	4.601	1.360	2.527	7.828
152		[E|E]	0.997																
155		[C|C]	0.998																
159		[C|C]	1.000																
160		[C|C]	0.999																
163		[C|C]	1.000																
167		[C|C]	1.000																
168		[C|C]	0.991	390	[C|C]	0.998		1.500	0.564	0.640	2.836	1.340	0.511	0.569	2.563	1.253	0.478	0.538	2.422
171		[B|B]	1.000																
172		[C|B]<br>[C|C]	0.673<br>0.248																
173	C → B	[C|C]<br>[C|BC]	0.762<br>0.224	391	[C|C]	0.993		5.143	1.441	2.869	8.517	4.626	1.351	2.494	7.810	4.322	1.264	2.388	7.318
174		[C|C]	0.908	392	[C|C]	0.987	0.994	8.009	2.054	4.758	12.751	7.232	1.925	4.155	11.635	6.757	1.815	3.972	11.028
177		[C|C]	1.000																
180		[C|C]	1.000																
183		[C|C]	0.996	385	[C|C]	0.998		1.629	0.586	0.748	3.027	1.478	0.531	0.669	2.716	1.393	0.510	0.649	2.605
186		[C|C]	0.999	383	[C|C]	1.000		0.770	0.332	0.266	1.557	0.704	0.298	0.251	1.419	0.660	0.286	0.230	1.341
192		[E|E]	1.000	379	[C|C]	0.995		7.481	1.921	4.354	11.904	6.766	1.794	3.877	10.852	6.324	1.695	3.723	10.418
193		[E|E]	0.991																
194		[E|E]	0.988																
195	C → E	[C|E]	0.940	382	[C|E]	0.936	0.607	1.164	0.444	0.514	2.230	1.050	0.394	0.463	2.007	0.985	0.374	0.432	1.899

Table S8 (Cont). Poison frog tree estimated divergence times, events (vicariance and dispersals), Lagrange ancestral area reconstructions, and BayesTraits probability of Amazon Basin area at the chronogram node (node numbers form Supplementary Figure S5 A-D).

Entire Phylogeny LaGrange				Chrono. LaGrange			Btrait	Time Constratins Set 1				Time Constratins Set 2				Time Constratins Set 3			
Node	Event	split	p	Node	split	p	p	mean	s.d.	L 95CI	U 95CI	mean	s.d.	L 95CI	U 95CI	mean	s.d.	L 95CI	U 95CI
196		[C|C]<br>[C|CE]	0.631<br>0.361	384	[C|C]<br>[C|CE]	0.732<br>0.263	0.975	2.287	0.762	1.144	4.110	2.074	0.678	1.034	3.692	1.947	0.650	0.979	3.492
197		[C|C]<br>[C|CE]	0.799<br>0.189	386	[C|C]	0.884	0.998	3.660	1.118	1.958	6.314	3.333	1.018	1.773	5.672	3.125	0.977	1.683	5.450
198		[C|C]	0.960	387	[C|C]	0.983	0.984	8.279	2.130	4.858	13.217	7.521	1.989	4.338	12.094	7.049	1.887	4.125	11.477
199		[C|C]	0.986	388	[C|C]	0.994	0.988	10.375	2.469	6.362	15.880	9.401	2.335	5.573	14.677	8.800	2.208	5.371	14.045
202		[C|C]	0.986																
204		[C|C]<br>[C|D]	0.773<br>0.174																
210		[C|C]	1.000																
211		[C|C]	1.000																
212		[C|C]	0.999	377	[C|C]	0.999		0.980	0.388	0.384	1.885	0.887	0.361	0.335	1.754	0.828	0.334	0.325	1.643
213		[C|C]	0.993	378	[C|C]	0.995	0.999	2.270	0.735	1.128	3.970	2.048	0.686	1.001	3.639	1.913	0.640	0.964	3.448
214	C → D	[C|C]	0.951	380	[C|C]	0.997		8.833	2.135	5.386	13.722	7.981	2.024	4.716	12.493	7.470	1.913	4.501	11.983
218		[C|C]	1.000																
222		[C|C]	1.000																
223		[C|C]	0.996	375	[C|C]	0.998		2.548	0.813	1.294	4.428	2.291	0.739	1.148	4.020	2.147	0.698	1.082	3.809
224	C → B	[C|C]	0.985	376	[C|C]	0.993		3.873	1.125	2.104	6.439	3.478	1.032	1.879	5.897	3.262	0.975	1.780	5.580
225		[C|C]<br>[B|C]	0.607<br>0.298																
226		[C|C]	0.907	381	[C|C]	0.996	0.998	9.887	2.334	6.076	15.233	8.935	2.218	5.326	13.959	8.364	2.094	5.117	13.338
227		[C|C]	0.986	389	[C|C]	0.998	0.999	11.766	2.673	7.384	17.749	10.639	2.532	6.462	16.240	9.961	2.402	6.203	15.717
228		[C|C]	0.985	393	[C|C]	0.996	0.998	12.839	2.852	8.101	19.253	11.621	2.718	7.155	17.726	10.869	2.576	6.825	16.973
229		[C|C]	0.974	394	[C|C]	0.983	0.994	13.953	3.023	8.924	20.817	12.626	2.906	7.844	19.117	11.794	2.737	7.514	18.252
230	C → E	[C|C]	0.783	395	[C|C]<br>[E|C]	0.757<br>0.159	0.937	15.159	3.202	9.820	22.365	13.692	3.108	8.575	20.594	12.802	2.908	8.257	19.635
231		[C|C]<br>[CG|C]	0.689<br>0.185	408	[C|C]<br>[CG|C]	0.742<br>0.154	0.975	16.772	3.436	11.007	24.483	15.139	3.367	9.604	22.763	14.139	3.123	9.204	21.544
232		[C|C]<br>[G|C]<br>[G|CG]	0.418<br>0.252<br>0.217	411	[C|C]<br>[G|C]<br>[G|CG]	0.469<br>0.237<br>0.179	0.841	17.745	3.558	11.743	25.715	16.016	3.501	10.198	23.900	14.951	3.237	9.831	22.636
233		[C|C]<br>[C|CG]	0.679<br>0.208	414	[C|C]	0.754	0.986	19.008	3.704	12.816	27.224	17.139	3.673	11.089	25.428	15.992	3.400	10.637	23.964

Table S8 (Cont). Poison frog tree estimated divergence times, events (vicariance and dispersals), Lagrange ancestral area reconstructions, and BayesTraits probability of Amazon Basin area at the chronogram node (node numbers form Supplementary Figure S5 A-D).

Entire Phylogeny LaGrange				Chrono. LaGrange			Btrait	Time Constratins Set 1				Time Constratins Set 2				Time Constratins Set 3			
Node	Event	split	p	Node	split	p	p	mean	s.d.	L 95CI	U 95CI	mean	s.d.	L 95CI	U 95CI	mean	s.d.	L 95CI	U 95CI
234	C → E	[E|C]<br>[C|C]	0.621<br>0.168	423	[E|C]<br>[C|C]	0.610<br>0.202	0.839	21.836	3.942	15.241	30.623	19.582	3.996	13.011	28.513	18.239	3.686	12.410	26.913
235	B → C	[B|C]	0.647	424	[B|C]<br>[BC|C]<br>[C|C]	0.506<br>0.178<br>0.150	0.639	23.753	4.179	16.638	32.958	21.502	4.218	14.638	31.125	20.014	3.878	13.921	29.106
236		[B|B]<br>[B|BC]	0.485<br>0.284	425	[B|B]<br>[B|BC]	0.359<br>0.290	0.462	25.699	4.471	18.040	35.391	23.354	4.491	16.063	33.535	21.711	4.106	15.279	31.317
237	D → B	[BD|B]<br>[D|D]	0.572<br>0.175	438	[BD|B]<br>[B|B]	0.349<br>0.168	0.156	31.247	4.973	22.625	42.240	29.051	4.939	21.160	40.304	26.935	4.407	20.325	37.377
238		[D|D]	0.718	444	[D|D]<br>[D|BD]	0.300<br>0.171	0.057	32.851	5.154	24.001	44.279	30.595	5.102	22.482	42.178	28.371	4.552	21.623	39.523
242		[I|F]	0.989																
246		[F|F]	0.999																
247		[I|F]	0.986																
251		[H|H]	0.999																
252		[H|H]	0.988																
255		[I|I]	1.000																
259		[F|F]	0.989																
260	I → F	[I|F]	0.914	367	[I|F]	0.819	0.000	1.125	0.473	0.376	2.237	1.051	0.448	0.346	2.117	1.067	0.463	0.355	2.170
261		[I|I]<br>[I|FI]	0.750<br>0.247																
262	I → H	[H|I]	0.884	368	[H|I]	0.843	0.000	1.415	0.547	0.573	2.711	1.322	0.519	0.516	2.569	1.345	0.543	0.534	2.617
263	I → F	[I|I]<br>[FI|I]	0.596<br>0.242	369	[I|I]<br>[I|HI]	0.817<br>0.161	0.001	2.723	0.885	1.337	4.804	2.537	0.832	1.249	4.458	2.588	0.872	1.255	4.683
264		[I|I]<br>[FI|I]	0.429<br>0.302	370	[I|I]	0.896	0.001	3.475	1.035	1.856	5.925	3.236	0.987	1.722	5.532	3.296	1.035	1.715	5.775
265		[I|I]<br>[I|FI]	0.701<br>0.271	371	[I|I]	0.923	0.004	5.837	1.514	3.402	9.323	5.452	1.480	3.096	8.808	5.537	1.527	3.057	9.034
271		[J|J]	0.999																
272	I|J	[J|J]	0.965	363	[J|J]	0.974	0.014	3.330	0.489	2.201	3.973	3.263	0.515	2.070	3.969	4.020	1.104	2.184	6.467
273	I → J	[I|J]	0.897	364	[I|J]	0.892	0.006	4.519	0.822	3.013	6.302	4.356	0.819	2.844	6.082	5.022	1.253	2.877	7.727
274		[I|I]	0.837	365	[I|I]<br>[I|IJ]	0.819<br>0.153	0.036	10.485	1.731	7.242	13.945	9.983	1.776	6.718	13.691	10.476	2.002	6.767	14.348
280		[J|J]	1.000																
282		[J|J]	1.000																

Table S8 (Cont). Poison frog tree estimated divergence times, events (vicariance and dispersals), Lagrange ancestral area reconstructions, and BayesTraits probability of Amazon Basin area at the chronogram node (node numbers form Supplementary Figure S5 A-D).

Entire Phylogeny LaGrange				Chrono. LaGrange			Btrait	Time Constratins Set 1				Time Constratins Set 2				Time Constratins Set 3			
Node	Event	split	p	Node	split	p	p	mean	s.d.	L 95CI	U 95CI	mean	s.d.	L 95CI	U 95CI	mean	s.d.	L 95CI	U 95CI
283		[J|J]	1.000																
286		[I|I]	0.996	358	[I|I]	0.995	0.000	0.911	0.361	0.312	1.729	0.852	0.348	0.279	1.650	0.881	0.375	0.276	1.758
289		[J|J]	1.000																
290	I|J	[I|J]	0.983	359	[I|J]	0.971	0.000	1.315	0.432	0.611	2.289	1.226	0.423	0.548	2.202	1.270	0.463	0.553	2.364
291	I|J	[J|IJ]	0.920	360	[J|IJ]	0.883	0.001	2.330	0.635	1.245	3.695	2.180	0.638	1.102	3.567	2.259	0.730	1.125	3.956
292	I → J	[J|IJ]	0.876	361	[J|IJ]	0.845	0.004	4.149	0.988	2.593	6.356	3.869	1.030	2.126	6.129	3.979	1.121	2.146	6.445
293		[I|I]<br>[I|IJ]	0.716<br>0.178	362	[I|I]	0.725	0.033	10.068	1.823	6.737	13.855	9.475	1.894	6.058	13.471	9.678	1.989	6.107	13.810
294		[I|I]	0.913	366	[I|I]	0.915	0.025	14.884	2.214	10.748	19.322	14.073	2.323	9.842	18.896	14.383	2.480	9.791	19.422
295		[I|I]	0.872	372	[I|I]	0.893	0.020	16.874	2.422	12.491	21.937	15.913	2.551	11.276	21.222	16.097	2.663	11.218	21.541
298		[J|J]	0.986	354	[J|J]	0.973	0.004	2.937	0.747	1.653	4.576	2.812	0.744	1.538	4.446	2.774	0.759	1.517	4.456
301		[I|I]	1.000																
305		[I|I]	1.000																
306		[I|I]	0.999																
307		[I|I]	0.982	353	[I|I]	0.979	0.011	3.981	0.937	2.351	6.035	3.811	0.954	2.171	5.906	3.745	0.963	2.087	5.825
308	I → J	[J|I]	0.931	355	[J|I]	0.888	0.003	5.388	1.086	3.447	7.706	5.155	1.111	3.193	7.516	5.080	1.138	3.082	7.492
313		[I|I]	1.000																
314		[I|I]	0.999	350	[I|I]	0.996		2.087	0.592	1.073	3.381	1.961	0.592	0.958	3.278	1.950	0.623	0.920	3.362
320		[J|J]	1.000																
321		[J|J]	0.992	347	[J|J]	0.989		2.202	0.583	1.244	3.515	2.005	0.594	1.026	3.333	1.968	0.598	0.978	3.339
322	I → J	[J|J]	0.950	348	[J|J]	0.919	0.002	3.370	0.679	2.455	4.964	3.049	0.780	1.718	4.748	3.001	0.793	1.666	4.749
323	I|J	[I|J]	0.757	349	[I|J]<br>[I|I]	0.670<br>0.213	0.015	6.778	1.160	4.713	9.215	6.326	1.267	3.989	8.996	6.235	1.300	3.889	8.909
324		[I|I]<br>[I|IJ]	0.716<br>0.270	351	[I|I]<br>[I|IJ]	0.690<br>0.291	0.007	8.076	1.240	5.783	10.604	7.564	1.373	5.009	10.349	7.460	1.409	4.839	10.327
325		[I|I]	0.930	352	[I|I]	0.906	0.023	11.388	1.403	8.489	13.952	10.830	1.611	7.540	13.720	10.654	1.640	7.402	13.662
326		[I|I]<br>[IJ|I]	0.729<br>0.186	356	[I|I]<br>[IJ|I]	0.691<br>0.193	0.013	13.146	1.324	10.092	14.921	12.561	1.618	9.045	14.875	12.366	1.690	8.760	14.871
332		[F|F]	1.000																
333		[F|F]	0.998	342	[F|F]	0.998		1.253	0.555	0.385	2.583	1.174	0.531	0.360	2.438	1.150	0.520	0.353	2.355
334		[F|F]	0.981	343	[F|F]	0.982		3.009	1.002	1.463	5.389	2.810	0.949	1.372	4.979	2.761	0.927	1.365	4.943
335	E → F	[F|F]	0.936	344	[F|F]	0.902	0.001	3.466	1.090	1.788	6.022	3.238	1.035	1.664	5.588	3.181	1.006	1.667	5.530
338		[G|G]	1.000																
342		[G|G]	1.000																
343		[G|G]	0.993	340	[G|G]	0.988		2.499	0.833	1.180	4.397	2.355	0.796	1.119	4.179	2.307	0.782	1.096	4.126

Table S8 (Cont). Poison frog tree estimated divergence times, events (vicariance and dispersals), Lagrange ancestral area reconstructions, and BayesTraits probability of Amazon Basin area at the chronogram node (node numbers form Supplementary Figure S5 A-D).

Entire Phylogeny LaGrange				Chrono. LaGrange			Btrait	Time Constratins Set 1				Time Constratins Set 2				Time Constratins Set 3			
Node	Event	split	p	Node	split	p	p	mean	s.d.	L 95CI	U 95CI	mean	s.d.	L 95CI	U 95CI	mean	s.d.	L 95CI	U 95CI
344		[G|G]	0.915	341	[G|G]	0.853	0.019	7.678	1.811	4.622	11.615	7.241	1.769	4.293	11.176	7.076	1.738	4.183	10.928
345	E|G	[E|G]	0.573	345	[E|G]<br>[E|E]	0.453<br>0.220	0.077	18.807	2.914	13.439	24.967	17.738	2.998	12.356	24.116	17.301	2.911	12.218	23.602
349	C → G	[C|G]	0.962																
351	C → B	[C|B]	0.807	335	[C|C]	0.995		2.427	0.804	1.170	4.287	2.257	0.755	1.081	3.992	2.212	0.734	1.054	3.950
355		[C|C]	0.999	330	[K|K]	0.945		1.705	0.596	0.768	3.091	1.595	0.551	0.720	2.900	1.561	0.545	0.702	2.838
358		[C|C]	1.000																
361		[C|C]	1.000																
364		[C|C]	1.000																
365		[C|C]	1.000	328	[G|G]	0.988		1.193	0.443	0.501	2.239	1.106	0.412	0.463	2.073	1.081	0.397	0.455	1.998
366		[C|C]	1.000	329	[C|G]	0.958		1.587	0.529	0.763	2.841	1.475	0.497	0.709	2.631	1.445	0.483	0.699	2.576
367		[C|C]	0.997	331	[K|C]	0.853		3.228	0.920	1.747	5.304	3.019	0.867	1.640	5.015	2.960	0.853	1.596	4.912
368	C → B	[C|C]<br>[B|C]	0.741<br>0.229	332	[C|C]	0.900		5.963	1.406	3.639	9.082	5.566	1.359	3.358	8.668	5.447	1.323	3.292	8.425
371	C → K	[K|K]<br>[CK|K]<br>[K|CK]	0.538<br>0.247<br>0.206	327	[G|G]	0.839		3.818	1.067	2.077	6.256	3.586	1.041	1.945	6.002	3.509	1.013	1.880	5.867
372		[C|C]	0.985	333	[C|G]<br>[C|C]	0.601<br>0.292		6.510	1.473	4.057	9.814	6.082	1.434	3.759	9.374	5.951	1.393	3.684	9.041
376		[C|G]	0.986																
380		[G|G]	1.000																
381		[G|G]	1.000	324	[C|B]	0.910		1.536	0.543	0.691	2.807	1.423	0.508	0.635	2.608	1.399	0.505	0.607	2.580
382		[G|G]<br>[CG|G]	0.616<br>0.380	325	[C|C]<br>[C|BC]	0.565<br>0.428		2.441	0.740	1.297	4.163	2.267	0.701	1.165	3.883	2.228	0.694	1.133	3.837
383		[G|G]	0.885																
386		[G|G]	0.975	321	[C|G]<br>[C|C]	0.656<br>0.237		3.832	1.051	2.129	6.179	3.591	1.008	1.987	5.879	3.515	0.990	1.943	5.819
390		[C|C]	1.000																
391		[C|C]	0.980	320	[G|C]	0.814		2.918	0.848	1.553	4.878	2.728	0.820	1.437	4.598	2.675	0.802	1.407	4.517
392		[G|C]	0.918	322	[C|C]	0.625		4.851	1.219	2.876	7.585	4.537	1.182	2.663	7.180	4.442	1.156	2.588	7.127
398		[C|B]	0.999																
399		[B|BC]	0.998																
400		[C|C]<br>[C|BC]	0.758<br>0.240																
401		[C|C]	0.993																

Table S8 (Cont). Poison frog tree estimated divergence times, events (vicariance and dispersals), Lagrange ancestral area reconstructions, and BayesTraits probability of Amazon Basin area at the chronogram node (node numbers form Supplementary Figure S5 A-D).

Entire Phylogeny LaGrange				Chrono. LaGrange			Btrait	Time Constratins Set 1				Time Constratins Set 2				Time Constratins Set 3			
Node	Event	split	p	Node	split	p	p	mean	s.d.	L 95CI	U 95CI	mean	s.d.	L 95CI	U 95CI	mean	s.d.	L 95CI	U 95CI
401		[C|C]	0.993																
405		[C|C]	1.000																
408		[C|C]	1.000																
409		[C|C]	1.000																
410		[C|C]	1.000																
411	C → B	[C|C]	0.998	318	[C|G]	0.917		1.263	0.456	0.533	2.324	1.183	0.432	0.505	2.171	1.157	0.422	0.495	2.149
415		[G|G]	1.000																
416		[G|G]	1.000																
417	G|C	[C|G]	0.985	319	[CG|C]<br>[C|C]	0.752<br>0.245		1.502	0.511	0.698	2.681	1.408	0.483	0.663	2.519	1.380	0.471	0.651	2.472
418	G → C	[CG|G]<br>[CG|C]<br>[G|G]	0.332<br>0.312<br>0.160	323	[C|C]	0.928		6.110	1.407	3.767	9.226	5.705	1.368	3.478	8.830	5.583	1.331	3.411	8.589
419		[G|CG]	0.747	326	[C|C]	0.954		6.363	1.456	3.942	9.607	5.942	1.409	3.654	9.174	5.818	1.375	3.575	8.935
420		[C|CG]	0.809	334	[CG|C]<br>[C|C]	0.528<br>0.441		6.786	1.524	4.232	10.195	6.335	1.482	3.917	9.742	6.203	1.447	3.837	9.476
421	G → C	[C|CG]	0.823	336	[C|CG]<br>[C|C]	0.507<br>0.484		7.237	1.620	4.522	10.811	6.750	1.572	4.180	10.342	6.610	1.537	4.098	10.113
422		[G|G]<br>[G|CG]	0.757<br>0.191	337	[G|CG]<br>[C|C]<br>[G|C]	0.481<br>0.294<br>0.174		7.904	1.761	4.971	11.856	7.354	1.701	4.556	11.186	7.213	1.675	4.458	11.079
426		[G|G]	0.999																
427		[C|G]<br>[G|G]	0.553<br>0.354																
431		[C|G]	0.992																
432		[C|CG]<br>[C|C]	0.813<br>0.179	316	[C|C]	0.991		1.961	0.717	0.850	3.613	1.818	0.676	0.763	3.412	1.784	0.675	0.734	3.355
433	G → C	[G|G]<br>[CG|C]	0.516<br>0.222	317	[C|C]<br>[B|C]	0.477<br>0.440		6.745	1.613	4.101	10.406	6.265	1.553	3.776	9.785	6.143	1.519	3.689	9.571
434	E|G	[G|G]<br>[G|CG]	0.593<br>0.187	338	[CG|C]<br>[C|C]	0.546<br>0.385		8.186	1.818	5.121	12.297	7.615	1.756	4.728	11.552	7.467	1.727	4.622	11.401
437	G → C	[C|G]	0.918	315	[C|G]<br>[B|C]	0.633<br>0.293	0.630	3.109	0.993	1.535	5.395	2.900	0.944	1.436	5.103	2.837	0.934	1.411	5.015

Table S8 (Cont). Poison frog tree estimated divergence times, events (vicariance and dispersals), Lagrange ancestral area reconstructions, and BayesTraits probability of Amazon Basin area at the chronogram node (node numbers form Supplementary Figure S5 A-D).

Entire Phylogeny LaGrange				Chrono. LaGrange			Btrait	Time Constratins Set 1				Time Constratins Set 2				Time Constratins Set 3			
Node	Event	split	p	Node	split	p	p	mean	s.d.	L 95CI	U 95CI	mean	s.d.	L 95CI	U 95CI	mean	s.d.	L 95CI	U 95CI
438		[G|G]<br>[G|CG]	0.351<br>0.298	339	[CG|C]<br>[C|C]<br>[C|CG]<br>[CG|G]	0.312<br>0.200<br>0.180<br>0.162	0.055	8.722	1.936	5.444	12.992	8.109	1.867	5.021	12.266	7.954	1.843	4.894	12.079
439	E → G	[EG|G]	0.593	346	[EG|G]<br>[E|E]	0.451<br>0.231	0.061	21.681	3.007	16.087	27.900	20.480	3.132	14.910	27.155	19.954	3.061	14.536	26.417
440	I|E	[I|E]	0.721	357	[I|E]	0.699	0.036	24.412	2.971	18.811	30.485	23.070	3.161	17.234	29.615	22.494	3.082	16.894	28.979
441	E → I	[I|EI]	0.713	373	[I|EI]	0.749	0.032	27.090	3.197	21.248	33.546	25.590	3.432	19.310	32.832	24.966	3.331	19.068	32.169
446		[J|J]	1.000																
447		[J|J]	0.997	310	[J|J]	0.994		2.421	0.889	1.070	4.548	2.213	0.841	0.956	4.239	2.092	0.797	0.916	3.963
448		[J|J]	0.983	311	[J|J]	0.963	0.001	3.168	1.051	1.567	5.675	2.892	0.993	1.416	5.322	2.735	0.946	1.350	5.019
452		[I|I]	1.000																
455		[I|I]	1.000	307	[I|I]	0.999		2.513	0.896	1.175	4.673	2.297	0.838	1.053	4.287	2.174	0.785	1.003	4.051
456		[I|I]	0.998	308	[I|I]	0.996		3.246	1.069	1.629	5.812	2.973	1.004	1.475	5.365	2.807	0.948	1.418	5.135
457		[I|I]	0.989	309	[I|I]	0.977	0.002	4.185	1.297	2.217	7.292	3.830	1.237	2.003	6.806	3.614	1.166	1.908	6.442
458	I → J	[J|I]	0.764	312	[J|I]	0.626	0.029	12.060	2.753	7.427	18.132	11.081	2.753	6.660	17.260	10.433	2.566	6.351	16.313
461		[B|B]	0.939																
465		[C|C]	1.000																
466		[C|C]	0.836	303	[C|C]	0.870	0.859	8.477	2.132	4.976	13.308	8.094	2.128	4.605	12.806	7.660	2.019	4.391	12.309
470		[C|C]	1.000																
471		[C|C]	0.999																
472	C → B	[C|C]	0.861	304	[C|C]	0.731	0.973	10.803	2.451	6.692	16.236	10.307	2.490	6.165	15.921	9.750	2.379	5.936	15.193
473		[C|C]<br>[B|C]	0.508<br>0.270																
476		[B|B]	0.996																
481		[B|B]	1.000																
482		[B|B]	0.999																
483		[B|B]	0.959	299	[B|B]	0.921	0.001	1.260	0.497	0.462	2.424	1.230	0.516	0.450	2.486	1.167	0.489	0.415	2.375
488		[F|I]	0.992																
489		[I|I]	0.915																
490		[I|I]	0.942																
496		[J|J]	1.000																
498		[J|J]	1.000																
500		[J|J]	1.000																
501		[J|J]	1.000																

Table S8 (Cont). Poison frog tree estimated divergence times, events (vicariance and dispersals), Lagrange ancestral area reconstructions, and BayesTraits probability of Amazon Basin area at the chronogram node (node numbers form Supplementary Figure S5 A-D).

<table>
<tr><th colspan="4">Entire Phylogeny LaGrange</th><th colspan="3">Chrono. LaGrange</th><th>Btrait</th><th colspan="4">Time Constratins Set 1</th><th colspan="4">Time Constratins Set 2</th><th colspan="4">Time Constratins Set 3</th></tr>
<tr><th>Node</th><th>Event</th><th>split</th><th>p</th><th>Node</th><th>split</th><th>p</th><th>p</th><th>mean</th><th>s.d.</th><th>L 95CI</th><th>U 95CI</th><th>mean</th><th>s.d.</th><th>L 95CI</th><th>U 95CI</th><th>mean</th><th>s.d.</th><th>L 95CI</th><th>U 95CI</th></tr>
<tr><td>502</td><td>I → J</td><td>[J|J]</td><td>0.993</td><td>296</td><td>[J|J]</td><td>0.987</td><td>0.000</td><td>0.578</td><td>0.254</td><td>0.136</td><td>1.126</td><td>0.620</td><td>0.326</td><td>0.133</td><td>1.398</td><td>0.586</td><td>0.309</td><td>0.127</td><td>1.326</td></tr>
<tr><td>503</td><td></td><td>[I|J]</td><td>0.965</td><td>297</td><td>[I|J]</td><td>0.946</td><td>0.000</td><td>1.223</td><td>0.366</td><td>0.558</td><td>1.975</td><td>1.327</td><td>0.541</td><td>0.539</td><td>2.637</td><td>1.260</td><td>0.521</td><td>0.511</td><td>2.547</td></tr>
<tr><td>504</td><td></td><td>[I|I]
[I|IJ]</td><td>0.605
0.330</td><td>298</td><td>[I|I]
[I|IJ]</td><td>0.533
0.403</td><td>0.000</td><td>1.772</td><td>0.385</td><td>0.972</td><td>2.366</td><td>1.971</td><td>0.717</td><td>0.927</td><td>3.710</td><td>1.870</td><td>0.689</td><td>0.865</td><td>3.547</td></tr>
<tr><td>505</td><td>C|B</td><td>[B|C]
[C|E]
[D|E]</td><td>0.374
0.324
0.179</td><td>300</td><td>[C|E]
[D|E]</td><td>0.389
0.344</td><td>0.030</td><td>9.739</td><td>1.949</td><td>6.330</td><td>13.966</td><td>9.549</td><td>2.315</td><td>5.735</td><td>14.803</td><td>9.032</td><td>2.178</td><td>5.523</td><td>13.962</td></tr>
<tr><td>506</td><td>C → B</td><td>[B|BC]
[C|CE]
[D|DE]</td><td>0.406
0.284
0.178</td><td>301</td><td>[C|CE]
[D|DE]</td><td>0.392
0.356</td><td>0.009</td><td>10.542</td><td>2.056</td><td>6.913</td><td>15.005</td><td>10.294</td><td>2.429</td><td>6.282</td><td>15.676</td><td>9.734</td><td>2.280</td><td>6.049</td><td>14.916</td></tr>
<tr><td>510</td><td></td><td>[J|J]</td><td>1.000</td><td></td><td></td><td></td><td></td><td></td><td></td><td></td><td></td><td></td><td></td><td></td><td></td><td></td><td></td><td></td><td></td></tr>
<tr><td>511</td><td></td><td>[J|J]</td><td>0.997</td><td>294</td><td>[J|J]</td><td>0.994</td><td>0.001</td><td>1.396</td><td>0.555</td><td>0.549</td><td>2.676</td><td>1.305</td><td>0.524</td><td>0.503</td><td>2.526</td><td>1.233</td><td>0.490</td><td>0.483</td><td>2.393</td></tr>
<tr><td>516</td><td></td><td>[I|F]</td><td>1.000</td><td></td><td></td><td></td><td></td><td></td><td></td><td></td><td></td><td></td><td></td><td></td><td></td><td></td><td></td><td></td><td></td></tr>
<tr><td>517</td><td></td><td>[I|FI]</td><td>0.993</td><td></td><td></td><td></td><td></td><td></td><td></td><td></td><td></td><td></td><td></td><td></td><td></td><td></td><td></td><td></td><td></td></tr>
<tr><td>518</td><td>I → F</td><td>[F|FI]</td><td>0.976</td><td>292</td><td>[F|I]
[I|I]</td><td>0.787
0.157</td><td>0.002</td><td>2.490</td><td>0.814</td><td>1.221</td><td>4.383</td><td>2.322</td><td>0.800</td><td>1.114</td><td>4.238</td><td>2.188</td><td>0.752</td><td>1.055</td><td>3.997</td></tr>
<tr><td>521</td><td></td><td>[I|I]</td><td>1.000</td><td></td><td></td><td></td><td></td><td></td><td></td><td></td><td></td><td></td><td></td><td></td><td></td><td></td><td></td><td></td><td></td></tr>
<tr><td>524</td><td></td><td>[J|J]</td><td>1.000</td><td>289</td><td>[J|J]</td><td>0.999</td><td></td><td>1.096</td><td>0.451</td><td>0.405</td><td>2.134</td><td>1.035</td><td>0.438</td><td>0.379</td><td>2.069</td><td>0.977</td><td>0.417</td><td>0.355</td><td>1.964</td></tr>
<tr><td>527</td><td></td><td>[J|J]</td><td>1.000</td><td></td><td></td><td></td><td></td><td></td><td></td><td></td><td></td><td></td><td></td><td></td><td></td><td></td><td></td><td></td><td></td></tr>
<tr><td>531</td><td></td><td>[J|J]</td><td>1.000</td><td>286</td><td>[J|J]</td><td>1.000</td><td></td><td>1.473</td><td>0.535</td><td>0.631</td><td>2.717</td><td>1.395</td><td>0.537</td><td>0.590</td><td>2.697</td><td>1.314</td><td>0.502</td><td>0.557</td><td>2.523</td></tr>
<tr><td>532</td><td></td><td>[J|J]</td><td>1.000</td><td>287</td><td>[J|J]</td><td>1.000</td><td></td><td>1.943</td><td>0.638</td><td>0.947</td><td>3.430</td><td>1.834</td><td>0.644</td><td>0.882</td><td>3.420</td><td>1.731</td><td>0.600</td><td>0.843</td><td>3.176</td></tr>
<tr><td>533</td><td></td><td>[J|J]</td><td>0.999</td><td>288</td><td>[J|J]</td><td>0.998</td><td></td><td>2.430</td><td>0.745</td><td>1.273</td><td>4.150</td><td>2.288</td><td>0.755</td><td>1.147</td><td>4.092</td><td>2.161</td><td>0.707</td><td>1.098</td><td>3.873</td></tr>
<tr><td>534</td><td></td><td>[J|J]</td><td>0.995</td><td>290</td><td>[J|J]</td><td>0.991</td><td>0.001</td><td>3.018</td><td>0.890</td><td>1.628</td><td>5.066</td><td>2.830</td><td>0.892</td><td>1.484</td><td>4.940</td><td>2.668</td><td>0.828</td><td>1.422</td><td>4.697</td></tr>
<tr><td>535</td><td>I|J</td><td>[I|J]</td><td>0.966</td><td>291</td><td>[I|J]</td><td>0.936</td><td>0.000</td><td>3.314</td><td>0.956</td><td>1.817</td><td>5.510</td><td>3.106</td><td>0.962</td><td>1.652</td><td>5.339</td><td>2.930</td><td>0.898</td><td>1.579</td><td>5.134</td></tr>
<tr><td>536</td><td></td><td>[I|IJ]</td><td>0.941</td><td>293</td><td>[I|IJ]</td><td>0.932</td><td>0.000</td><td>3.706</td><td>1.047</td><td>2.037</td><td>6.077</td><td>3.474</td><td>1.065</td><td>1.860</td><td>5.941</td><td>3.278</td><td>0.993</td><td>1.781</td><td>5.724</td></tr>
<tr><td>537</td><td>E → IJ</td><td>[J|IJ]</td><td>0.876</td><td>295</td><td>[J|IJ]</td><td>0.849</td><td>0.017</td><td>8.331</td><td>1.992</td><td>4.962</td><td>12.728</td><td>7.838</td><td>2.045</td><td>4.512</td><td>12.521</td><td>7.383</td><td>1.915</td><td>4.333</td><td>11.820</td></tr>
<tr><td>538</td><td></td><td>[C|E]
[E|I]
[E|E]</td><td>0.437
0.172
0.158</td><td>302</td><td>[E|I]
[E|E]
[C|E]</td><td>0.413
0.229
0.155</td><td>0.070</td><td>19.549</td><td>3.157</td><td>13.904</td><td>26.075</td><td>18.568</td><td>3.539</td><td>12.543</td><td>26.295</td><td>17.508</td><td>3.300</td><td>11.936</td><td>24.885</td></tr>
<tr><td>539</td><td>E → C</td><td>[C|CE]
[C|E]</td><td>0.576
0.275</td><td>305</td><td>[E|E]
[C|CE]
[E|EI]
[C|E]</td><td>0.248
0.233
0.211
0.207</td><td>0.347</td><td>21.145</td><td>3.346</td><td>15.192</td><td>28.071</td><td>20.038</td><td>3.715</td><td>13.663</td><td>28.232</td><td>18.902</td><td>3.478</td><td>13.110</td><td>26.820</td></tr>
<tr><td>543</td><td></td><td>[G|G]</td><td>1.000</td><td></td><td></td><td></td><td></td><td></td><td></td><td></td><td></td><td></td><td></td><td></td><td></td><td></td><td></td><td></td><td></td></tr>
<tr><td>544</td><td></td><td>[G|G]</td><td>0.945</td><td></td><td></td><td></td><td></td><td></td><td></td><td></td><td></td><td></td><td></td><td></td><td></td><td></td><td></td><td></td><td></td></tr>
</table>

Table S8 (Cont). Poison frog tree estimated divergence times, events (vicariance and dispersals), Lagrange ancestral area reconstructions, and BayesTraits probability of Amazon Basin area at the chronogram node (node numbers form Supplementary Figure S5 A-D).

Entire Phylogeny LaGrange				Chrono. LaGrange			Btrait	Time Constratins Set 1				Time Constratins Set 2				Time Constratins Set 3			
Node	Event	split	p	Node	split	p	p	mean	s.d.	L 95CI	U 95CI	mean	s.d.	L 95CI	U 95CI	mean	s.d.	L 95CI	U 95CI
548		[F|F]	0.997																
549	E → F	[E|F]	0.940	282	[E|F]	0.927	0.001	2.010	0.724	0.873	3.677	1.791	0.693	0.734	3.456	1.690	0.675	0.690	3.341
552		[I|I]	0.988																
557		[J|J]	0.986																
558	I|J	[I|J]	0.804	279	[I|J]	0.764	0.006	3.584	0.872	2.456	5.740	3.109	0.967	1.585	5.336	2.923	0.926	1.485	5.066
559	I → J	[I|I]<br>[J|IJ]<br>[I|IJ]	0.366<br>0.243<br>0.228	280	[I|I]<br>[J|IJ]<br>[I|IJ]	0.389<br>0.230<br>0.211	0.003	4.732	1.115	3.044	7.388	4.186	1.207	2.301	6.912	3.932	1.139	2.143	6.592
560		[I|I]	0.869	281	[I|I]	0.906	0.036	9.312	1.866	6.140	13.409	8.399	2.035	4.960	12.920	7.899	1.945	4.716	12.403
561	E → I	[E|I]	0.857	283	[E|I]	0.852	0.006	10.085	1.971	6.785	14.477	9.107	2.139	5.513	13.826	8.572	2.057	5.203	13.370
564		[G|G]	0.993																
568		[C|C]	1.000																
569		[C|C]	0.983																
571	G → C	[C|G]	0.950	277	[C|C]	0.978	0.642	2.447	0.798	1.201	4.322	2.234	0.769	1.043	4.033	2.107	0.728	1.016	3.806
572		[G|G]<br>[G|CG]	0.601<br>0.373																
576		[C|C]	1.000																
577		[C|C]	0.997	273	[G|C]	0.854		4.390	1.145	2.505	6.982	4.059	1.137	2.237	6.676	3.826	1.076	2.139	6.334
581		[C|C]	0.999																
584		[G|G]	1.000																
585		[C|G]	0.964																
586		[C|C]<br>[C|CG]	0.550<br>0.444	272	[C|C]	0.998		1.494	0.526	0.678	2.737	1.382	0.501	0.616	2.563	1.299	0.475	0.574	2.430
590		[B|B]	0.999																
593		[B|B]	1.000																
596		[B|B]	1.000																
597		[B|B]	0.999																
598		[B|B]	0.987																
599	C|B	[C|B]	0.955	271	[G|C]<br>[C|B]	0.472<br>0.447		2.380	0.748	1.186	4.081	2.192	0.731	1.065	3.900	2.059	0.683	1.008	3.658
600	C → B	[C|C]	0.791	275	[C|C]	0.828	0.933	6.039	1.430	3.635	9.201	5.587	1.445	3.273	8.890	5.261	1.370	3.100	8.476
601		[C|C]	0.953	274	[C|C]	0.845	0.996	2.880	0.851	1.519	4.812	2.654	0.839	1.360	4.635	2.503	0.789	1.291	4.333
604		[C|C]	0.999																
606		[C|C]	0.951																
609		[G|G]	0.998																

Table S8 (Cont). Poison frog tree estimated divergence times, events (vicariance and dispersals), Lagrange ancestral area reconstructions, and BayesTraits probability of Amazon Basin area at the chronogram node (node numbers form Supplementary Figure S5 A-D).

<table>
<tr><th colspan="4">Entire Phylogeny LaGrange</th><th colspan="3">Chrono. LaGrange</th><th>Btrait</th><th colspan="4">Time Constratins Set 1</th><th colspan="4">Time Constratins Set 2</th><th colspan="4">Time Constratins Set 3</th></tr>
<tr><th>Node</th><th>Event</th><th>split</th><th>p</th><th>Node</th><th>split</th><th>p</th><th>p</th><th>mean</th><th>s.d.</th><th>L 95CI</th><th>U 95CI</th><th>mean</th><th>s.d.</th><th>L 95CI</th><th>U 95CI</th><th>mean</th><th>s.d.</th><th>L 95CI</th><th>U 95CI</th></tr>
<tr><td>610</td><td></td><td>[C|G]</td><td>0.846</td><td>269</td><td>[C|C]</td><td>0.998</td><td>0.695</td><td>3.434</td><td>0.962</td><td>1.862</td><td>5.595</td><td>3.173</td><td>0.942</td><td>1.675</td><td>5.372</td><td>2.990</td><td>0.897</td><td>1.585</td><td>5.092</td></tr>
<tr><td>613</td><td></td><td>[C|C]</td><td>1.000</td><td></td><td></td><td></td><td></td><td></td><td></td><td></td><td></td><td></td><td></td><td></td><td></td><td></td><td></td><td></td><td></td></tr>
<tr><td>616</td><td></td><td>[C|C]</td><td>1.000</td><td></td><td></td><td></td><td></td><td></td><td></td><td></td><td></td><td></td><td></td><td></td><td></td><td></td><td></td><td></td><td></td></tr>
<tr><td>619</td><td></td><td>[C|C]</td><td>1.000</td><td></td><td></td><td></td><td></td><td></td><td></td><td></td><td></td><td></td><td></td><td></td><td></td><td></td><td></td><td></td><td></td></tr>
<tr><td>620</td><td></td><td>[C|C]</td><td>0.999</td><td>267</td><td>[C|C]</td><td>0.991</td><td></td><td>2.017</td><td>0.655</td><td>0.991</td><td>3.521</td><td>1.863</td><td>0.628</td><td>0.879</td><td>3.320</td><td>1.751</td><td>0.592</td><td>0.831</td><td>3.102</td></tr>
<tr><td>621</td><td></td><td>[C|C]</td><td>0.999</td><td>268</td><td>[G|C]</td><td>0.840</td><td>1.000</td><td>2.805</td><td>0.808</td><td>1.513</td><td>4.660</td><td>2.590</td><td>0.791</td><td>1.357</td><td>4.482</td><td>2.437</td><td>0.754</td><td>1.288</td><td>4.244</td></tr>
<tr><td>622</td><td>C → G</td><td>[CG|C]
[C|C]</td><td>0.738
0.248</td><td>270</td><td>[C|C]
[C|CG]</td><td>0.717
0.274</td><td>0.991</td><td>4.436</td><td>1.149</td><td>2.556</td><td>7.075</td><td>4.104</td><td>1.139</td><td>2.272</td><td>6.760</td><td>3.864</td><td>1.085</td><td>2.180</td><td>6.377</td></tr>
<tr><td>623</td><td>G → C</td><td>[C|CG]
[C|C]</td><td>0.636
0.304</td><td>276</td><td>[C|C]</td><td>0.765</td><td>1.000</td><td>6.545</td><td>1.539</td><td>3.971</td><td>9.943</td><td>6.048</td><td>1.543</td><td>3.551</td><td>9.578</td><td>5.699</td><td>1.468</td><td>3.385</td><td>9.132</td></tr>
<tr><td>624</td><td></td><td>[G|G]
[CG|C]</td><td>0.522
0.182</td><td>278</td><td>[C|C]</td><td>0.723</td><td>0.967</td><td>10.320</td><td>2.190</td><td>6.614</td><td>15.168</td><td>9.470</td><td>2.259</td><td>5.721</td><td>14.500</td><td>8.914</td><td>2.129</td><td>5.501</td><td>13.840</td></tr>
<tr><td>625</td><td>E|G</td><td>[E|G]</td><td>0.810</td><td>284</td><td>[E|C]</td><td>0.622</td><td>0.504</td><td>13.674</td><td>2.480</td><td>9.426</td><td>19.120</td><td>12.506</td><td>2.698</td><td>7.932</td><td>18.576</td><td>11.774</td><td>2.550</td><td>7.474</td><td>17.545</td></tr>
<tr><td>626</td><td>E → G</td><td>[G|EG]</td><td>0.763</td><td>285</td><td>[G|E]
[E|E]</td><td>0.244
0.233</td><td>0.259</td><td>16.805</td><td>2.914</td><td>11.752</td><td>23.127</td><td>15.452</td><td>3.137</td><td>10.109</td><td>22.286</td><td>14.541</td><td>2.987</td><td>9.497</td><td>21.153</td></tr>
<tr><td>627</td><td></td><td>[E|E]</td><td>0.615</td><td>306</td><td>[E|E]</td><td>0.593</td><td>0.253</td><td>25.451</td><td>3.680</td><td>18.882</td><td>33.178</td><td>23.855</td><td>4.078</td><td>16.859</td><td>32.676</td><td>22.463</td><td>3.828</td><td>16.060</td><td>31.145</td></tr>
<tr><td>628</td><td>E → I</td><td>[I|E]
[E|E]</td><td>0.628
0.184</td><td>313</td><td>[I|E]
[E|E]
[I|EI]</td><td>0.396
0.325
0.164</td><td>0.171</td><td>29.914</td><td>4.038</td><td>22.662</td><td>38.326</td><td>27.843</td><td>4.450</td><td>20.315</td><td>37.681</td><td>26.193</td><td>4.150</td><td>19.556</td><td>35.980</td></tr>
<tr><td>632</td><td></td><td>[G|G]</td><td>1.000</td><td></td><td></td><td></td><td></td><td></td><td></td><td></td><td></td><td></td><td></td><td></td><td></td><td></td><td></td><td></td><td></td></tr>
<tr><td>633</td><td></td><td>[G|G]</td><td>0.928</td><td>264</td><td>[G|G]</td><td>0.883</td><td></td><td>7.183</td><td>1.909</td><td>4.099</td><td>11.531</td><td>6.410</td><td>1.832</td><td>3.529</td><td>10.673</td><td>6.011</td><td>1.721</td><td>3.350</td><td>10.033</td></tr>
<tr><td>637</td><td></td><td>[E|E]</td><td>0.992</td><td>261</td><td>[E|E]</td><td>0.966</td><td></td><td>4.018</td><td>0.906</td><td>2.770</td><td>6.098</td><td>3.388</td><td>1.071</td><td>1.641</td><td>5.799</td><td>3.231</td><td>1.128</td><td>1.547</td><td>5.921</td></tr>
<tr><td>638</td><td></td><td>[E|E]</td><td>0.962</td><td>262</td><td>[E|E]</td><td>0.925</td><td>0.101</td><td>20.574</td><td>3.318</td><td>14.732</td><td>27.670</td><td>18.390</td><td>3.585</td><td>12.317</td><td>26.184</td><td>17.300</td><td>3.371</td><td>11.670</td><td>25.107</td></tr>
<tr><td>641</td><td></td><td>[E|E]</td><td>0.987</td><td>258</td><td>[E|E]</td><td>0.981</td><td>0.003</td><td>2.910</td><td>0.896</td><td>1.529</td><td>5.039</td><td>2.516</td><td>0.864</td><td>1.193</td><td>4.525</td><td>2.360</td><td>0.808</td><td>1.135</td><td>4.250</td></tr>
<tr><td>645</td><td></td><td>[C|C]</td><td>0.999</td><td></td><td></td><td></td><td></td><td></td><td></td><td></td><td></td><td></td><td></td><td></td><td></td><td></td><td></td><td></td><td></td></tr>
<tr><td>646</td><td>E → C</td><td>[C|C]</td><td>0.983</td><td>257</td><td>[C|C]</td><td>0.967</td><td>1.000</td><td>1.593</td><td>0.587</td><td>0.682</td><td>2.977</td><td>1.375</td><td>0.540</td><td>0.544</td><td>2.633</td><td>1.299</td><td>0.512</td><td>0.509</td><td>2.501</td></tr>
<tr><td>647</td><td>E → C</td><td>[E|C]</td><td>0.822</td><td>259</td><td>[E|C]</td><td>0.728</td><td>0.656</td><td>7.355</td><td>1.658</td><td>5.128</td><td>11.369</td><td>6.351</td><td>1.732</td><td>3.544</td><td>10.288</td><td>5.975</td><td>1.648</td><td>3.394</td><td>9.827</td></tr>
<tr><td>650</td><td></td><td>[E|C]</td><td>0.986</td><td></td><td></td><td></td><td></td><td></td><td></td><td></td><td></td><td></td><td></td><td></td><td></td><td></td><td></td><td></td><td></td></tr>
<tr><td>653</td><td></td><td>[E|E]</td><td>1.000</td><td></td><td></td><td></td><td></td><td></td><td></td><td></td><td></td><td></td><td></td><td></td><td></td><td></td><td></td><td></td><td></td></tr>
<tr><td>656</td><td></td><td>[E|E]</td><td>1.000</td><td></td><td></td><td></td><td></td><td></td><td></td><td></td><td></td><td></td><td></td><td></td><td></td><td></td><td></td><td></td><td></td></tr>
<tr><td>657</td><td></td><td>[E|E]</td><td>0.999</td><td>254</td><td>[E|E]</td><td>0.998</td><td></td><td>4.403</td><td>1.257</td><td>2.422</td><td>7.268</td><td>3.875</td><td>1.161</td><td>2.053</td><td>6.601</td><td>3.639</td><td>1.085</td><td>1.937</td><td>6.189</td></tr>
<tr><td>658</td><td>E → C</td><td>[E|E]
[CE|E]</td><td>0.758
0.232</td><td>255</td><td>[E|E]</td><td>0.995</td><td>0.003</td><td>5.129</td><td>1.384</td><td>2.938</td><td>8.325</td><td>4.525</td><td>1.293</td><td>2.494</td><td>7.518</td><td>4.248</td><td>1.215</td><td>2.356</td><td>7.084</td></tr>
<tr><td>661</td><td></td><td>[E|E]</td><td>1.000</td><td></td><td></td><td></td><td></td><td></td><td></td><td></td><td></td><td></td><td></td><td></td><td></td><td></td><td></td><td></td><td></td></tr>
</table>

Table S8 (Cont). Poison frog tree estimated divergence times, events (vicariance and dispersals), Lagrange ancestral area reconstructions, and BayesTraits probability of Amazon Basin area at the chronogram node (node numbers form Supplementary Figure S5 A-D).

Entire Phylogeny LaGrange				Chrono. LaGrange			Btrait	Time Constratins Set 1				Time Constratins Set 2				Time Constratins Set 3			
Node	Event	split	p	Node	split	p	p	mean	s.d.	L 95CI	U 95CI	mean	s.d.	L 95CI	U 95CI	mean	s.d.	L 95CI	U 95CI
664		[C|C]	1.000																
668		[C|E]	0.998																
669		[E|CE]	1.000																
670		[C|CE]	0.979																
671	E → C	[E|E]<br>[E|CE]	0.749<br>0.230	253	[E|E]	0.993	0.102	4.735	1.363	2.596	7.849	4.183	1.282	2.217	7.144	3.938	1.203	2.074	6.794
672		[E|E]	0.728	256	[E|E]	0.986	0.009	7.164	1.776	4.295	11.240	6.332	1.698	3.646	10.273	5.959	1.590	3.466	9.654
673		[E|E]<br>[CE|E]	0.496<br>0.325	260	[E|E]<br>[CE|E]	0.652<br>0.293	0.225	13.198	2.599	9.081	19.098	11.619	2.658	7.225	17.475	10.918	2.503	6.876	16.650
674		[E|E]	0.920	263	[E|E]	0.908	0.106	25.274	3.535	19.052	32.795	22.736	3.922	16.148	31.382	21.345	3.634	15.451	29.719
675	E|G	[G|E]<br>[E|E]	0.550<br>0.252	265	[G|E]<br>[E|E]<br>[EG|E]	0.429<br>0.319<br>0.168	0.070	28.079	3.764	21.389	35.977	25.359	4.197	18.335	34.606	23.791	3.852	17.540	32.628
678		[H|G]	0.824	251	[H|G]<br>[G|G]	0.594<br>0.189	0.019	11.143	2.382	7.070	16.298	9.739	2.389	5.829	15.203	9.148	2.202	5.589	14.175
683		[G|G]	1.000																
684		[G|G]	0.995	247	[G|G]	0.995	0.008	3.469	1.113	1.727	6.000	3.048	1.003	1.529	5.399	2.876	0.939	1.443	5.092
689		[G|G]	1.000																
690		[G|G]	1.000																
691		[G|G]	1.000																
692		[G|G]	0.977	248	[G|G]	0.975	0.005	4.852	1.400	2.685	8.054	4.265	1.279	2.309	7.255	4.026	1.206	2.171	6.839
693	G → E	[G|G]<br>[E|G]	0.537<br>0.254	249	[G|G]<br>[E|G]	0.673<br>0.153	0.138	21.360	3.266	15.586	28.387	18.667	3.537	12.655	26.356	17.526	3.266	12.097	24.979
696	G|E	[G|E]	0.940	244	[G|E]	0.829	0.005	5.821	0.743	5.022	7.842	3.767	1.170	1.903	6.498	3.539	1.101	1.807	6.129
702		[F|F]	0.999																
703	E|F	[E|F]	0.949	240	[E|F]	0.914	0.001	2.224	0.762	0.998	3.962	1.808	0.670	0.788	3.383	1.695	0.625	0.741	3.162
704	E|F	[E|E]<br>[E|EF]	0.624<br>0.361	241	[E|E]<br>[E|EF]	0.716<br>0.264	0.009	6.445	1.495	3.894	9.764	5.257	1.450	2.970	8.591	4.943	1.356	2.814	8.057
707		[E|E]	1.000																
710		[E|E]	1.000																
711		[E|E]	1.000																
712		[E|E]<br>[EF|E]	0.651<br>0.342	242	[E|E]<br>[EF|E]	0.733<br>0.248	0.003	7.372	1.615	4.667	10.907	6.001	1.592	3.500	9.622	5.646	1.497	3.307	9.111

Table S8 (Cont). Poison frog tree estimated divergence times, events (vicariance and dispersals), Lagrange ancestral area reconstructions, and BayesTraits probability of Amazon Basin area at the chronogram node (node numbers form Supplementary Figure S5 A-D).

Entire Phylogeny LaGrange				Chrono. LaGrange			Btrait	Time Constratins Set 1				Time Constratins Set 2				Time Constratins Set 3			
Node	Event	split	p	Node	split	p	p	mean	s.d.	L 95CI	U 95CI	mean	s.d.	L 95CI	U 95CI	mean	s.d.	L 95CI	U 95CI
713	E → F	[F|E]	0.352	243	[E|E]	0.355	0.015	10.986	2.089	7.436	15.621	8.965	2.166	5.414	13.911	8.446	2.037	5.140	13.067
		[F|EF]	0.336		[F|E]	0.324													
		[E|E]	0.266		[F|EF]	0.248													
714	G → E	[EG|E]	0.869	245	[EG|E]	0.794	0.016	14.930	2.277	11.031	19.897	12.005	2.635	7.601	17.917	11.288	2.467	7.293	16.857
718		[G|G]	1.000																
719		[G|G]	0.999																
723		[G|G]	1.000																
724		[G|G]	1.000																
727		[G|C]	0.999																
731		[C|C]	1.000																
732		[C|C]	1.000																
733	G → C	[CG|C]	0.865	236	[G|C]	0.763	0.923	5.585	1.592	3.070	9.225	4.981	1.486	2.678	8.399	4.686	1.394	2.525	7.974
734		[G|G]	0.949	237	[G|G]	0.952	0.544	14.640	2.754	9.855	20.608	12.849	2.810	8.221	19.202	12.076	2.591	7.864	17.957
735		[G|G]	0.981	238	[G|G]	0.980	0.304	16.198	2.858	11.230	22.453	14.209	2.949	9.313	20.870	13.356	2.743	8.896	19.600
738		[G|G]	1.000																
743		[H|H]	0.998																
744		[G|H]	0.966																
745		[G|GH]	0.964																
748		[H|H]	0.999	230	[H|H]	0.999		1.518	0.579	0.603	2.856	1.313	0.520	0.526	2.568	1.239	0.492	0.500	2.404
752		[H|H]	1.000																
753		[H|H]	0.999	229	[H|H]	0.999		1.899	0.682	0.813	3.485	1.635	0.606	0.703	3.054	1.549	0.579	0.652	2.881
754		[H|H]	0.996	231	[H|H]	0.994	0.001	2.982	0.898	1.567	5.029	2.578	0.818	1.328	4.502	2.432	0.772	1.262	4.266
757	I|F	[I|F]	0.849	227	[F|I]	0.801	0.005	3.442	1.035	1.790	5.821	3.039	0.953	1.562	5.251	2.864	0.895	1.456	4.941
760		[I|I]	1.000																
764		[F|F]	1.000																
767		[F|F]	1.000																
768		[F|F]	0.993	224	[F|H]	0.948	0.001	1.257	0.501	0.469	2.416	1.108	0.449	0.410	2.163	1.052	0.433	0.388	2.073
769	I|F	[I|F]	0.981	225	[F|FH]	0.889	0.000	1.602	0.562	0.733	2.924	1.409	0.501	0.647	2.579	1.339	0.484	0.603	2.470
770		[I|I]	0.685	226	[I|H]	0.672	0.008	4.645	1.297	2.569	7.629	4.111	1.210	2.179	6.890	3.872	1.125	2.098	6.423
		[I|FI]	0.285		[I|F]	0.179													

Table S8 (Cont). Poison frog tree estimated divergence times, events (vicariance and dispersals), Lagrange ancestral area reconstructions, and BayesTraits probability of Amazon Basin area at the chronogram node (node numbers form Supplementary Figure S5 A-D).

Entire Phylogeny LaGrange				Chrono. LaGrange			Btrait	Time Constratins Set 1				Time Constratins Set 2				Time Constratins Set 3			
Node	Event	split	p	Node	split	p	p	mean	s.d.	L 95CI	U 95CI	mean	s.d.	L 95CI	U 95CI	mean	s.d.	L 95CI	U 95CI
771	I → F	[I|I]<br>[FI|I]	0.509<br>0.196	228	[I|HI]<br>[I|I]	0.631<br>0.201	0.005	6.640	1.620	3.954	10.321	5.852	1.538	3.378	9.357	5.515	1.445	3.206	8.834
772	H|I	[H|I]<br>[H|H]<br>[H|F]	0.453<br>0.317<br>0.175	232	[H|HI]<br>[H|I]<br>[H|H]	0.439<br>0.262<br>0.250	0.008	8.703	1.876	5.579	12.908	7.592	1.841	4.601	11.710	7.151	1.721	4.410	11.101
774	H → I	[H|H]<br>[HI|H]	0.743<br>0.181	233	[HI|I]<br>[H|H]	0.698<br>0.176	0.003	9.440	1.965	6.122	13.767	8.214	1.954	5.058	12.607	7.740	1.821	4.793	11.863
775	G → H	[GH|H]	0.825	234	[G|H]	0.791	0.008	11.465	2.248	7.595	16.372	9.955	2.273	6.209	15.072	9.373	2.115	5.902	14.190
776		[G|G]<br>[G|GH]	0.621<br>0.275	235	[G|G]<br>[G|GH]	0.661<br>0.290	0.011	13.690	2.500	9.360	19.069	11.914	2.572	7.592	17.601	11.208	2.386	7.316	16.632
777		[G|G]	0.921	239	[G|G]	0.937	0.051	18.457	2.961	13.229	24.782	16.136	3.151	10.860	23.091	15.178	2.934	10.389	21.956
778		[G|G]	0.804	246	[G|G]	0.799	0.032	22.389	3.101	16.967	28.963	19.344	3.525	13.269	27.193	18.176	3.271	12.880	25.508
779		[G|G]<br>[EG|G]	0.667<br>0.174	250	[G|G]	0.701	0.017	23.799	3.240	18.066	30.835	20.736	3.668	14.552	28.951	19.473	3.391	13.975	27.275
780	G → H	[G|G]<br>[G|EG]	0.612<br>0.271	252	[G|G]<br>[G|EG]	0.651<br>0.254	0.014	25.429	3.406	19.415	32.640	22.369	3.845	15.968	30.916	21.023	3.534	15.268	29.179
781	E → G	[EG|G]<br>[E|E]	0.633<br>0.197	266	[EG|G]<br>[E|E]	0.550<br>0.245	0.023	30.918	3.921	23.938	39.216	27.976	4.399	20.686	37.924	26.259	4.012	19.983	35.556
782		[E|E]<br>[EI|E]	0.666<br>0.187	314	[E|E]	0.732	0.059	34.633	4.235	27.054	43.452	32.046	4.695	24.428	42.543	30.084	4.273	23.535	40.224
783		[E|E]	0.481	374	[E|E]	0.735	0.044	37.037	4.422	29.126	46.276	34.558	4.828	26.774	45.267	32.541	4.343	26.037	43.075
785	D|E	[D|E]	0.540	446	[D|E]	0.590	0.057	40.931	5.360	31.387	52.584	38.704	5.457	30.640	51.290	35.968	4.748	30.309	48.050
784	--	--	--	445	--	--	0.075	34.921	5.373	25.516	46.683	32.644	5.335	24.272	44.778	30.283	4.744	23.306	41.944
