# Supplementary material for: Amazonian Amphibian Diversity Is Primarily Derived from Late Miocene Andean Lineages
Source: PLoS Biol. 2009 Mar 10;7(3):e1000056. doi: 10.1371/journal.pbio.1000056 (PMC2653552; doi:10.1371/journal.pbio.1000056)
Supplement: Supplementary file 11 [file pbio.1000056.st009.pdf]

Table S9A. Poison frog tree estimated divergence times and taxonomic events (node numbers form Supplementary Figure S5 A-D).

		MULTIDIVTIME									
node	Taxonomic Event	Time Set 1	"bigtime" 60	"bigtime" 80	Without A	Without B	Without C	Without D	Without E	Without F	Without G
733	*H. azureiventris*/*H. chlorocraspedus* split	5.585 ± 1.592 (3.070-9.225)	5.575 ± 1.576 (3.034-9.197)	5.575 ± 1.576 (3.034-9.197)	6.980 ± 1.955 (3.785-11.347)	6.689 ± 1.862 (3.718-10.930)	6.494 ± 1.800 (3.569-10.576)	5.166 ± 1.495 (2.788-8.545)	6.363 ± 1.778 (3.473-10.382)	6.394 ± 1.774 (3.516-10.411)	6.449 ± 1.811 (3.532-10.564)
673	*H. bocagei* complex	13.198 ± 2.599 (9.081-19.098)	13.145 ± 2.553 (8.994-18.923)	13.145 ± 2.553 (8.994-18.923)	15.906 ± 3.241 (10.387-22.960)	14.986 ± 2.967 (9.974-21.637)	14.585 ± 2.880 (9.756-20.840)	12.553 ± 2.383 (8.658-17.932)	14.455 ± 2.886 (9.631-20.849)	14.375 ± 3.026 (9.101-21.081)	14.681 ± 2.915 (9.798-21.034)
781	Clade B (Hyloxinae)	30.918 ± 3.921 (23.938-39.216)	30.916 ± 3.940 (23.959-39.460)	30.916 ± 3.940 (23.959-39.460)	38.447 ± 4.881 (29.163-47.898)	35.962 ± 4.146 (28.353-44.559)	35.068 ± 4.000 (27.710-43.277)	29.106 ± 3.797 (22.535-37.386)	34.591 ± 4.232 (26.982-43.590)	34.808 ± 4.136 (27.226-43.449)	35.132 ± 4.190 (27.488-43.813)
599	Dispersal of Epipedobates to Gorgona Island (50 km W of Pacific Coast of	2.380 ± 0.748 (1.186-4.081)	2.372 ± 0.743 (1.201-4.104)	2.372 ± 0.743 (1.201-4.104)	2.880 ± 0.969 (1.388-5.133)	2.666 ± 0.840 (1.336-4.592)	2.592 ± 0.817 (1.307-4.470)	2.335 ± 0.736 (1.182-4.028)	2.549 ± 0.805 (1.283-4.405)	2.563 ± 0.803 (1.295-4.419)	2.580 ± 0.814 (1.276-4.412)
623	*D. ventrimaculatus* complex (widespread Amazon Basin taxa)	6.545 ± 1.539 (3.971-9.943)	6.556 ± 1.531 (4.045-9.993)	6.556 ± 1.531 (4.045-9.993)	7.968 ± 2.037 (4.597-12.481)	7.371 ± 1.708 (4.529-11.161)	7.139 ± 1.639 (4.383-10.745)	6.445 ± 1.495 (4.038-9.869)	7.070 ± 1.636 (4.295-10.710)	7.089 ± 1.628 (4.371-10.699)	7.139 ± 1.661 (4.368-10.770)
560	*D. fulguritus* complex	9.312 ± 1.866 (6.140-13.409)	9.257 ± 1.857 (6.149-13.354)	9.257 ± 1.857 (6.149-13.354)	11.344 ± 2.704 (6.785-17.384)	10.346 ± 1.958 (6.827-14.287)	10.114 ± 1.939 (6.669-14.144)	9.036 ± 1.802 (6.045-13.124)	10.082 ± 1.947 (6.618-14.079)	10.085 ± 1.945 (6.649-14.142)	10.124 ± 1.962 (6.611-14.137)
534	*D. pumilio* complex	3.018 ± 0.890 (1.628-5.066)	3.024 ± 0.881 (1.627-5.062)	3.024 ± 0.881 (1.627-5.062)	3.881 ± 1.229 (2.001-6.810)	3.532 ± 0.995 (1.931-5.797)	3.399 ± 0.980 (1.850-5.606)	2.973 ± 0.864 (1.630-5.034)	3.384 ± 0.982 (1.795-5.636)	3.386 ± 0.969 (1.841-5.586)	3.431 ± 1.001 (1.831-5.701)
472	*D. quinquevittatus* complex (widespread Amazon Basin taxa)	10.803 ± 2.451 (6.692-16.236)	10.891 ± 2.490 (6.721-16.473)	10.891 ± 2.490 (6.721-16.473)	13.443 ± 3.132 (8.189-20.590)	12.548 ± 2.845 (7.709-18.911)	12.045 ± 2.698 (7.430-18.009)	10.761 ± 2.476 (6.646-16.288)	11.923 ± 2.710 (7.326-17.980)	11.997 ± 2.669 (7.450-17.783)	12.087 ± 2.723 (7.448-18.107)
627	*Dendrobates*	25.451 ± 3.680 (18.882-33.178)	25.515 ± 3.732 (18.821-33.390)	25.515 ± 3.732 (18.821-33.390)	31.557 ± 4.927 (22.405-41.669)	29.134 ± 3.959 (21.808-37.130)	28.172 ± 3.742 (21.306-35.792)	24.831 ± 3.614 (18.433-32.401)	27.907 ± 3.861 (20.824-35.668)	28.062 ± 3.815 (20.967-35.837)	28.284 ± 3.903 (21.054-36.296)
458	*Phyllobates*	12.060 ± 2.753 (7.427-18.132)	12.065 ± 2.787 (7.443-18.235)	12.065 ± 2.787 (7.443-18.235)	15.084 ± 3.417 (9.070-22.445)	12.336 ± 1.795 (8.396-14.872)	12.205 ± 1.797 (8.387-14.851)	11.011 ± 2.075 (7.013-14.669)	12.090 ± 1.866 (8.151-14.856)	12.150 ± 1.857 (8.126-14.863)	12.194 ± 1.842 (8.286-14.872)
628	Clade D (Dendrobatinae)	29.914 ± 4.038 (22.662-38.326)	29.940 ± 4.114 (22.701-38.689)	29.940 ± 4.114 (22.701-38.689)	37.010 ± 5.252 (27.200-47.366)	33.880 ± 4.092 (26.175-42.295)	32.948 ± 3.976 (25.460-41.116)	28.695 ± 3.815 (21.809-36.538)	32.518 ± 4.040 (25.028-40.861)	32.743 ± 4.001 (25.386-41.045)	32.968 ± 4.064 (25.461-41.431)
782	Clades B and D ancestor	34.633 ± 4.235 (27.054-43.452)	34.670 ± 4.318 (26.978-43.855)	34.670 ± 4.318 (26.978-43.855)	42.816 ± 5.361 (32.149-52.892)	39.745 ± 4.360 (31.495-48.397)	38.662 ± 4.225 (30.620-47.224)	33.048 ± 4.089 (25.865-41.669)	38.128 ± 4.454 (29.902-47.514)	38.434 ± 4.348 (30.263-47.370)	38.744 ± 4.473 (30.576-48.148)
417	*A. trivittatus* complex (widespread Amazon Basin species)	1.502 ± 0.511 (0.698-2.681)	1.495 ± 0.505 (0.695-2.687)	1.495 ± 0.505 (0.695-2.687)	1.877 ± 0.663 (0.849-3.454)	1.673 ± 0.559 (0.805-2.975)	1.583 ± 0.543 (0.750-2.846)	1.474 ± 0.493 (0.702-2.602)	1.603 ± 0.543 (0.744-2.869)	1.595 ± 0.540 (0.759-2.859)	1.605 ± 0.545 (0.749-2.865)
372	*A. pictus* complex (widespread Amazon Basin taxa)	6.510 ± 1.473 (4.057-9.814)	6.492 ± 1.458 (4.103-9.745)	6.492 ± 1.458 (4.103-9.745)	8.239 ± 2.022 (4.879-12.807)	7.226 ± 1.596 (4.532-10.746)	6.891 ± 1.562 (4.261-10.445)	6.339 ± 1.421 (3.997-9.484)	6.969 ± 1.562 (4.360-10.501)	6.980 ± 1.580 (4.363-10.591)	6.977 ± 1.574 (4.359-10.486)
421	Major radiation of *Ameerega* in the Amazon	7.237 ± 1.620 (4.522-10.811)	7.211 ± 1.601 (4.573-10.781)	7.211 ± 1.601 (4.573-10.781)	9.198 ± 2.233 (5.529-14.196)	8.039 ± 1.758 (5.080-11.910)	7.677 ± 1.709 (4.778-11.517)	7.044 ± 1.558 (4.461-10.492)	7.757 ± 1.722 (4.885-11.674)	7.773 ± 1.735 (4.876-11.647)	7.775 ± 1.728 (4.892-11.607)
438	*Ameerega*	8.722 ± 1.936 (5.444-12.992)	8.681 ± 1.895 (5.501-12.887)	8.681 ± 1.895 (5.501-12.887)	11.140 ± 2.676 (6.706-17.155)	9.704 ± 2.107 (6.148-14.298)	9.290 ± 2.052 (5.840-13.892)	8.467 ± 1.843 (5.394-12.570)	9.380 ± 2.061 (5.939-13.967)	9.386 ± 2.053 (5.954-13.919)	9.392 ± 2.062 (5.989-13.889)
345	*Colostethus* 2	18.807 ± 2.914 (13.439-24.967)	18.835 ± 2.894 (13.561-24.842)	18.835 ± 2.894 (13.561-24.842)	23.841 ± 4.234 (16.183-32.855)	20.902 ± 3.066 (15.364-27.385)	20.105 ± 2.983 (14.685-26.291)	18.394 ± 2.875 (13.243-24.375)	20.036 ± 3.017 (14.595-26.354)	20.112 ± 3.024 (14.560-26.424)	20.184 ± 3.026 (14.618-26.482)
326	*Colostethus* 1	13.146 ± 1.324 (10.092-14.921)	13.173 ± 1.299 (10.207-14.926)	13.173 ± 1.299 (10.207-14.926)	17.741 ± 3.406 (11.697-25.145)	13.621 ± 1.088 (10.925-14.954)	13.495 ± 1.154 (10.768-14.946)	12.999 ± 1.381 (9.831-14.910)	13.461 ± 1.175 (10.624-14.945)	13.483 ± 1.179 (10.603-14.951)	13.490 ± 1.166 (10.693-14.947)

Table S9A (Cont). Poison frog tree estimated divergence times and taxonomic events (node numbers form Supplementary Figure S5 A-D).

		MULTIDIVTIME									
node	Taxonomic Event	Time Set 1	"bigtime" 60	"bigtime" 80	Without A	Without B	Without C	Without D	Without E	Without F	Without G
294	*Silverstoneia*	14.884 ± 2.214 (10.748-19.322)	14.920 ± 2.220 (10.846-19.442)	14.920 ± 2.220 (10.846-19.442)	17.921 ± 3.143 (12.378-24.647)	17.000 ± 2.314 (12.492-21.476)	15.712 ± 2.287 (11.441-20.357)	14.581 ± 2.219 (10.536-19.197)	15.609 ± 2.273 (11.338-20.278)	15.685 ± 2.274 (11.445-20.291)	15.680 ± 2.285 (11.435-20.276)
265	*Epipedobates*	5.837 ± 1.514 (3.402-9.323)	5.883 ± 1.527 (3.352-9.324)	5.883 ± 1.527 (3.352-9.324)	7.353 ± 2.052 (4.029-12.053)	6.719 ± 1.707 (3.918-10.554)	6.314 ± 1.655 (3.594-10.046)	5.668 ± 1.490 (3.249-9.061)	6.289 ± 1.630 (3.627-9.960)	6.318 ± 1.640 (3.654-10.125)	6.285 ± 1.621 (3.643-9.998)
441	Clade C (Colostethinae)	27.090 ± 3.197 (21.248-33.546)	27.151 ± 3.175 (21.238-33.706)	27.151 ± 3.175 (21.238-33.706)	33.914 ± 4.852 (24.747-43.707)	30.301 ± 3.285 (24.194-37.018)	29.036 ± 3.228 (22.975-35.664)	26.467 ± 3.150 (20.718-33.115)	28.818 ± 3.238 (22.835-35.536)	28.988 ± 3.245 (23.003-35.672)	29.079 ± 3.261 (23.067-35.830)
783	Clade B, C and D ancestor	37.037 ± 4.422 (29.126-46.276)	37.088 ± 4.497 (29.006-46.602)	37.088 ± 4.497 (29.006-46.602)	45.604 ± 5.608 (34.278-55.823)	42.300 ± 4.557 (33.507-51.244)	41.076 ± 4.427 (32.721-49.998)	35.577 ± 4.247 (28.301-44.467)	40.487 ± 4.632 (31.897-50.307)	40.840 ± 4.509 (32.333-50.031)	41.164 ± 4.642 (32.681-50.871)
226	*A. trilineatus* complex (widespread Amazon Basin taxa)	9.887 ± 2.334 (6.076-15.233)	9.833 ± 2.279 (6.021-14.916)	9.833 ± 2.279 (6.021-14.916)	11.168 ± 2.676 (6.667-17.061)	11.027 ± 2.529 (6.859-16.648)	10.698 ± 2.469 (6.612-16.252)	9.829 ± 2.206 (6.173-14.643)	10.346 ± 2.400 (6.352-15.705)	10.612 ± 2.424 (6.628-16.074)	10.691 ± 2.477 (6.635-16.212)
174	*A. marchesianus* complex (widespread Amazon Basin taxa)	8.009 ± 2.054 (4.758-12.751)	7.977 ± 1.999 (4.694-12.407)	7.977 ± 1.999 (4.694-12.407)	9.083 ± 2.366 (5.232-14.365)	8.945 ± 2.222 (5.348-13.968)	8.721 ± 2.206 (5.107-13.743)	7.970 ± 1.965 (4.804-12.434)	8.414 ± 2.127 (4.954-13.335)	8.620 ± 2.154 (5.144-13.459)	8.707 ± 2.189 (5.090-13.682)
228	Radiation of *Allobates* in the Amazon Basin	12.839 ± 2.852 (8.101-19.253)	12.794 ± 2.770 (8.058-18.849)	12.794 ± 2.770 (8.058-18.849)	14.541 ± 3.286 (9.006-21.726)	14.343 ± 3.048 (9.277-20.985)	13.913 ± 2.984 (8.890-20.362)	12.779 ± 2.688 (8.269-18.710)	13.466 ± 2.927 (8.564-19.990)	13.805 ± 2.936 (8.969-20.282)	13.933 ± 3.022 (8.903-20.673)
149	*A. femoralis* complex (widespread Amazon Basin species)	5.432 ± 1.557 (2.969-9.057)	5.390 ± 1.518 (3.011-8.868)	5.390 ± 1.518 (3.011-8.868)	6.156 ± 1.825 (3.321-10.436)	6.077 ± 1.738 (3.356-10.102)	5.916 ± 1.676 (3.230-9.775)	5.439 ± 1.480 (3.056-8.847)	5.720 ± 1.639 (3.202-9.601)	5.874 ± 1.655 (3.262-9.693)	5.895 ± 1.682 (3.261-9.760)
99	*A. brunneus* group (widespread Amazon Basin taxa)	15.059 ± 3.201 (9.772-22.215)	15.016 ± 3.154 (9.705-21.980)	15.016 ± 3.154 (9.705-21.980)	17.104 ± 3.774 (10.723-25.393)	16.833 ± 3.496 (10.950-24.606)	16.442 ± 3.401 (10.662-24.011)	14.953 ± 3.024 (9.807-21.636)	15.917 ± 3.322 (10.293-23.441)	16.301 ± 3.346 (10.646-23.547)	16.445 ± 3.427 (10.731-24.004)
233	Radiation of *Allobates* in the Amazon Basin	19.008 ± 3.704 (12.816-27.224)	18.949 ± 3.629 (12.684-26.922)	18.949 ± 3.629 (12.684-26.922)	21.568 ± 4.358 (13.968-30.938)	21.255 ± 3.971 (14.507-29.974)	20.688 ± 3.897 (13.982-29.160)	18.894 ± 3.447 (13.016-26.496)	20.069 ± 3.836 (13.585-28.577)	20.549 ± 3.841 (14.050-28.900)	20.734 ± 3.949 (14.142-29.456)
86	*A. talamancae* complex	13.485 ± 2.818 (8.913-19.922)	13.437 ± 2.751 (8.942-19.521)	13.437 ± 2.751 (8.942-19.521)	14.707 ± 3.558 (8.712-22.544)	14.887 ± 3.097 (9.780-21.906)	14.577 ± 3.054 (9.561-21.527)	13.309 ± 2.625 (9.030-19.267)	14.197 ± 2.946 (9.377-20.884)	14.499 ± 2.979 (9.637-21.283)	14.629 ± 3.040 (9.616-21.670)
236	*Allobates* (without *A. alagoanus*)	25.699 ± 4.471 (18.040-35.391)	25.628 ± 4.392 (17.936-35.055)	25.628 ± 4.392 (17.936-35.055)	29.642 ± 5.290 (20.108-40.681)	28.905 ± 4.754 (20.585-39.203)	28.222 ± 4.646 (20.088-38.246)	25.416 ± 4.111 (18.417-34.457)	27.378 ± 4.669 (19.333-37.615)	28.034 ± 4.605 (20.129-37.995)	28.293 ± 4.721 (20.416-38.576)
58	*Anomaloglossus*	19.726 ± 3.804 (13.166-28.023)	19.671 ± 3.826 (13.071-27.990)	19.671 ± 3.826 (13.071-27.990)	23.527 ± 4.405 (15.590-32.785)	22.475 ± 4.061 (15.276-31.191)	21.969 ± 3.989 (14.936-30.626)	19.492 ± 3.540 (13.489-27.252)	21.278 ± 4.053 (14.298-30.072)	21.820 ± 3.987 (15.002-30.574)	22.077 ± 4.138 (15.040-31.269)
19	Disperals to Trinidad from Venezuela (*Mannophryne*)	8.300 ± 2.365 (4.474-13.648)	8.294 ± 2.396 (4.410-13.710)	8.294 ± 2.396 (4.410-13.710)	9.850 ± 2.821 (5.243-16.194)	9.459 ± 2.633 (5.127-15.455)	9.179 ± 2.550 (5.052-14.884)	8.161 ± 2.256 (4.408-13.288)	8.974 ± 2.601 (4.799-14.950)	9.184 ± 2.605 (4.849-14.995)	9.257 ± 2.603 (5.001-15.175)
20	*Mannophryne*	11.101 ± 2.790 (6.508-17.465)	11.076 ± 2.802 (6.529-17.561)	11.076 ± 2.802 (6.529-17.561)	13.217 ± 3.312 (7.720-20.536)	12.648 ± 3.093 (7.577-19.638)	12.317 ± 2.994 (7.374-18.915)	10.886 ± 2.664 (6.506-16.890)	12.010 ± 3.058 (6.995-18.816)	12.318 ± 3.072 (7.241-19.211)	12.410 ± 3.061 (7.353-19.225)
6	*Aromobates*	16.960 ± 3.664 (10.782-24.975)	16.949 ± 3.655 (10.868-24.934)	16.949 ± 3.655 (10.868-24.934)	20.165 ± 4.275 (12.668-29.287)	19.301 ± 3.986 (12.409-28.066)	18.804 ± 3.921 (12.069-27.381)	16.679 ± 3.469 (10.783-24.341)	18.289 ± 3.891 (11.616-26.814)	18.751 ± 3.843 (12.047-27.099)	18.897 ± 3.908 (12.106-27.541)
784	Clade A (Allobatinae)	34.921 ± 5.373 (25.516-46.683)	34.909 ± 5.387 (25.594-46.453)	34.909 ± 5.387 (25.594-46.453)	41.502 ± 6.041 (29.918-53.181)	39.686 ± 5.527 (29.615-51.041)	38.637 ± 5.369 (28.947-49.912)	34.404 ± 4.956 (26.120-45.208)	37.640 ± 5.571 (27.861-49.799)	38.446 ± 5.397 (28.727-49.792)	38.837 ± 5.600 (29.132-50.696)
785	Dendrobatidae	40.931 ± 5.360 (31.387-52.584)	40.997 ± 5.459 (31.353-53.089)	40.997 ± 5.459 (31.353-53.089)	49.444 ± 5.998 (37.074-59.262)	46.564 ± 5.381 (36.347-57.140)	45.275 ± 5.227 (35.290-55.953)	39.728 ± 5.042 (31.338-50.833)	44.467 ± 5.463 (34.595-56.091)	45.069 ± 5.384 (35.067-56.132)	45.502 ± 5.683 (35.384-57.555)

Table S9B. Poison frog tree estimated divergence times and taxonomic events (node numbers form Supplementary Figure S5A-D).

		MULTIDIVTIME	r8s							
node	Taxonomic Event	Time Set 1	With All	Without A	Without B	Without C	Without D	Without E	Without F	Without G
733	*H. azureiventris*/*H. chlorocraspedus* split	5.585 ± 1.592 (3.070-9.225)	5.502 ± 0.173 (5.800-5.370)	5.488 ± 0.177 (5.790-5.350)	5.502 ± 0.173 (5.800-5.370)	5.502 ± 0.173 (5.800-5.370)	5.068 ± 0.086 (5.140-4.920)	5.488 ± 0.177 (5.790-5.350)	5.502 ± 0.173 (5.800-5.370)	5.320 ± 0.138 (5.550-5.190)
673	*H. bocagei* complex	13.198 ± 2.599 (9.081-19.098)	11.274 ± 0.719 (12.270-10.690)	11.250 ± 0.733 (12.270-10.650)	11.274 ± 0.719 (12.270-10.690)	11.274 ± 0.719 (12.270-10.690)	10.714 ± 0.670 (11.750-10.210)	11.244 ± 0.739 (12.270-10.640)	11.274 ± 0.719 (12.270-10.690)	10.844 ± 0.478 (11.480-10.450)
781	Clade B (Hyloxinae)	30.918 ± 3.921 (23.938-39.216)	29.486 ± 1.715 (32.060-28.140)	29.438 ± 1.747 (32.060-28.070)	29.486 ± 1.715 (32.060-28.140)	29.486 ± 1.715 (32.060-28.140)	28.064 ± 1.560 (30.630-26.970)	29.426 ± 1.757 (32.060-28.050)	29.486 ± 1.715 (32.060-28.140)	28.210 ± 1.130 (29.920-27.340)
599	Dispersal of Epipedobates to Gorgona Island (50 km W of Pacific Coast of	2.380 ± 0.748 (1.186-4.081)	2.250 ± 0.119 (2.450-2.170)	2.246 ± 0.123 (2.450-2.160)	2.250 ± 0.119 (2.450-2.170)	2.250 ± 0.119 (2.450-2.170)	2.170 ± 0.148 (2.420-2.070)	2.246 ± 0.123 (2.450-2.160)	2.250 ± 0.119 (2.450-2.170)	2.176 ± 0.078 (2.310-2.120)
623	*D. ventrimaculatus* complex (widespread Amazon Basin taxa)	6.545 ± 1.539 (3.971-9.943)	6.184 ± 0.402 (6.420-5.480)	6.168 ± 0.392 (6.400-5.480)	6.184 ± 0.402 (6.420-5.480)	6.184 ± 0.402 (6.420-5.480)	5.946 ± 0.317 (6.130-5.390)	6.168 ± 0.392 (6.400-5.480)	6.184 ± 0.402 (6.420-5.480)	5.976 ± 0.475 (6.260-5.160)
560	*D. fulguritus* complex	9.312 ± 1.866 (6.140-13.409)	9.176 ± 0.295 (9.370-8.660)	9.154 ± 0.282 (9.340-8.660)	9.176 ± 0.295 (9.370-8.660)	9.176 ± 0.295 (9.370-8.660)	8.824 ± 0.184 (8.940-8.500)	9.156 ± 0.283 (9.340-8.660)	9.176 ± 0.295 (9.370-8.660)	8.846 ± 0.446 (9.120-8.090)
534	*D. pumilio* complex	3.018 ± 0.890 (1.628-5.066)	2.574 ± 0.082 (2.630-2.430)	2.568 ± 0.079 (2.620-2.430)	2.574 ± 0.082 (2.630-2.430)	2.574 ± 0.082 (2.630-2.430)	2.478 ± 0.050 (2.510-2.390)	2.568 ± 0.079 (2.620-2.430)	2.574 ± 0.082 (2.630-2.430)	2.486 ± 0.122 (2.560-2.280)
472	*D. quinquevittatus* complex (widespread Amazon Basin taxa)	10.803 ± 2.451 (6.692-16.236)	11.480 ± 0.671 (11.870-10.300)	11.456 ± 0.657 (11.840-10.300)	11.480 ± 0.671 (11.870-10.300)	11.480 ± 0.671 (11.870-10.300)	11.074 ± 0.536 (11.380-10.130)	11.458 ± 0.658 (11.840-10.300)	11.480 ± 0.671 (11.870-10.300)	11.058 ± 0.794 (11.530-9.690)
627	*Dendrobates*	25.451 ± 3.680 (18.882-33.178)	25.758 ± 0.373 (26.000-25.100)	25.716 ± 0.351 (25.940-25.100)	25.758 ± 0.373 (26.000-25.100)	25.758 ± 0.373 (26.000-25.100)	24.938 ± 0.170 (25.080-24.650)	25.716 ± 0.353 (25.950-25.100)	25.758 ± 0.373 (26.000-25.100)	24.642 ± 0.690 (25.050-23.450)
458	*Phyllobates*	12.060 ± 2.753 (7.427-18.132)	9.614 ± 0.159 (9.830-9.470)	9.594 ± 0.173 (9.830-9.440)	9.614 ± 0.159 (9.830-9.470)	9.614 ± 0.159 (9.830-9.470)	9.278 ± 0.233 (9.650-9.100)	9.596 ± 0.175 (9.830-9.440)	9.614 ± 0.159 (9.830-9.470)	9.222 ± 0.024 (9.250-9.190)
628	Clade D (Dendrobatinae)	29.914 ± 4.038 (22.662-38.326)	30.258 ± 0.179 (30.550-30.110)	30.214 ± 0.189 (30.520-30.050)	30.258 ± 0.179 (30.550-30.110)	30.258 ± 0.179 (30.550-30.110)	29.376 ± 0.213 (29.640-29.180)	30.214 ± 0.195 (30.530-30.040)	30.258 ± 0.179 (30.550-30.110)	28.846 ± 0.392 (29.080-28.160)
782	Clades B and D ancestor	34.633 ± 4.235 (27.054-43.452)	36.194 ± 0.585 (37.020-35.700)	36.156 ± 0.612 (37.020-35.640)	36.194 ± 0.585 (37.020-35.700)	36.194 ± 0.585 (37.020-35.700)	35.290 ± 0.652 (36.330-34.800)	36.152 ± 0.615 (37.020-35.630)	36.194 ± 0.585 (37.020-35.700)	34.328 ± 0.111 (34.480-34.230)
417	*A. trivittatus* complex (widespread Amazon Basin species)	1.502 ± 0.511 (0.698-2.681)	1.000 ± 0.067 (1.040-0.880)	0.998 ± 0.067 (1.040-0.880)	1.000 ± 0.067 (1.040-0.880)	1.000 ± 0.067 (1.040-0.880)	0.964 ± 0.053 (0.990-0.870)	1.000 ± 0.067 (1.040-0.880)	1.000 ± 0.067 (1.040-0.880)	0.968 ± 0.078 (1.010-0.830)
372	*A. pictus* complex (widespread Amazon Basin taxa)	6.510 ± 1.473 (4.057-9.814)	5.106 ± 0.217 (5.230-4.720)	5.092 ± 0.208 (5.210-4.720)	5.106 ± 0.217 (5.230-4.720)	5.106 ± 0.217 (5.230-4.720)	4.924 ± 0.144 (5.020-4.670)	5.096 ± 0.211 (5.220-4.720)	5.106 ± 0.217 (5.230-4.720)	4.944 ± 0.275 (5.100-4.460)
421	Major radiation of *Ameerega* in the Amazon	7.237 ± 1.620 (4.522-10.811)	5.626 ± 0.177 (5.730-5.310)	5.612 ± 0.169 (5.710-5.310)	5.626 ± 0.177 (5.730-5.310)	5.626 ± 0.177 (5.730-5.310)	5.426 ± 0.104 (5.520-5.250)	5.616 ± 0.172 (5.720-5.310)	5.626 ± 0.177 (5.730-5.310)	5.446 ± 0.248 (5.590-5.010)
438	*Ameerega*	8.722 ± 1.936 (5.444-12.992)	6.028 ± 0.156 (6.120-5.750)	6.014 ± 0.149 (6.100-5.750)	6.028 ± 0.156 (6.120-5.750)	6.028 ± 0.156 (6.120-5.750)	5.816 ± 0.084 (5.920-5.690)	6.014 ± 0.149 (6.100-5.750)	6.028 ± 0.156 (6.120-5.750)	5.830 ± 0.232 (5.960-5.420)
345	*Colostethus* 2	18.807 ± 2.914 (13.439-24.967)	16.262 ± 0.309 (16.690-15.990)	16.226 ± 0.330 (16.690-15.940)	16.262 ± 0.309 (16.690-15.990)	16.262 ± 0.309 (16.690-15.990)	15.732 ± 0.504 (16.510-15.340)	16.230 ± 0.331 (16.690-15.940)	16.262 ± 0.309 (16.690-15.990)	15.640 ± 0.056 (15.730-15.590)
326	*Colostethus* 1	13.146 ± 1.324 (10.092-14.921)	12.110 ± 0.282 (12.520-11.870)	12.084 ± 0.298 (12.520-11.830)	12.110 ± 0.282 (12.520-11.870)	12.110 ± 0.282 (12.520-11.870)	11.704 ± 0.432 (12.380-11.380)	12.086 ± 0.300 (12.520-11.830)	12.110 ± 0.282 (12.520-11.870)	11.668 ± 0.073 (11.750-11.600)

Table S9B (Cont). Poison frog tree estimated divergence times and taxonomic events (node numbers form Supplementary Figure S5A-D).

		MULTIDIVTIME	r8s							
node	Taxonomic Event	Time Set 1	With All	Without A	Without B	Without C	Without D	Without E	Without F	Without G
294	*Silverstoneia*	14.884 ± 2.214<br>(10.748-19.322)	13.142 ± 0.135<br>(13.360-13.020)	13.112 ± 0.141<br>(13.330-12.980)	13.142 ± 0.135<br>(13.360-13.020)	13.142 ± 0.135<br>(13.360-13.020)	12.706 ± 0.250<br>(13.010-12.490)	13.118 ± 0.148<br>(13.350-12.980)	13.142 ± 0.135<br>(13.360-13.020)	12.672 ± 0.166<br>(12.790-12.380)
265	*Epipedobates*	5.837 ± 1.514<br>(3.402-9.323)	4.032 ± 0.191<br>(4.350-3.900)	4.024 ± 0.195<br>(4.350-3.890)	4.032 ± 0.191<br>(4.350-3.900)	4.032 ± 0.191<br>(4.350-3.900)	3.892 ± 0.243<br>(4.300-3.730)	4.026 ± 0.196<br>(4.350-3.890)	4.032 ± 0.191<br>(4.350-3.900)	3.892 ± 0.116<br>(4.090-3.810)
441	Clade C (Colostethinae)	27.090 ± 3.197<br>(21.248-33.546)	25.428 ± 0.402<br>(25.970-25.070)	25.384 ± 0.426<br>(25.970-25.010)	25.428 ± 0.402<br>(25.970-25.070)	25.428 ± 0.402<br>(25.970-25.070)	24.712 ± 0.631<br>(25.680-24.220)	25.390 ± 0.429<br>(25.970-25.010)	25.428 ± 0.402<br>(25.970-25.070)	24.328 ± 0.069<br>(24.440-24.280)
783	Clade B, C and D ancestor	37.037 ± 4.422<br>(29.126-46.276)	38.382 ± 0.423<br>(38.970-38.020)	38.352 ± 0.443<br>(38.970-37.970)	38.382 ± 0.423<br>(38.970-38.020)	38.382 ± 0.423<br>(38.970-38.020)	37.706 ± 0.518<br>(38.520-37.310)	38.352 ± 0.445<br>(38.970-37.970)	38.382 ± 0.423<br>(38.970-38.020)	36.260 ± 0.000<br>(36.260-36.260)
226	*A. trilineatus* complex (widespread Amazon Basin taxa)	9.887 ± 2.334<br>(6.076-15.233)	11.782 ± 2.093<br>(13.370-8.520)	11.654 ± 2.218<br>(13.330-8.180)	11.782 ± 2.093<br>(13.370-8.520)	11.782 ± 2.093<br>(13.370-8.520)	11.462 ± 1.848<br>(12.820-8.520)	11.746 ± 2.088<br>(13.330-8.490)	11.782 ± 2.093<br>(13.370-8.520)	11.674 ± 1.930<br>(13.150-8.670)
174	*A. marchesianus* complex (widespread Amazon Basin taxa)	8.009 ± 2.054<br>(4.758-12.751)	8.950 ± 1.553<br>(10.130-6.530)	8.850 ± 1.645<br>(10.090-6.270)	8.950 ± 1.553<br>(10.130-6.530)	8.950 ± 1.553<br>(10.130-6.530)	8.708 ± 1.365<br>(9.710-6.530)	8.924 ± 1.548<br>(10.100-6.510)	8.950 ± 1.553<br>(10.130-6.530)	8.870 ± 1.427<br>(9.960-6.650)
228	Radiation of *Allobates* in the Amazon Basin	12.839 ± 2.852<br>(8.101-19.253)	15.874 ± 2.440<br>(17.710-12.040)	15.692 ± 2.615<br>(17.640-11.560)	15.874 ± 2.440<br>(17.710-12.040)	15.874 ± 2.440<br>(17.710-12.040)	15.458 ± 2.124<br>(16.990-12.040)	15.826 ± 2.433<br>(17.650-12.000)	15.874 ± 2.440<br>(17.710-12.040)	15.740 ± 2.221<br>(17.420-12.260)
149	*A. femoralis* complex (widespread Amazon Basin species)	5.432 ± 1.557<br>(2.969-9.057)	5.682 ± 0.939<br>(6.380-4.190)	5.620 ± 0.999<br>(6.360-4.030)	5.682 ± 0.939<br>(6.380-4.190)	5.682 ± 0.939<br>(6.380-4.190)	5.530 ± 0.824<br>(6.110-4.190)	5.664 ± 0.935<br>(6.360-4.180)	5.682 ± 0.939<br>(6.380-4.190)	5.632 ± 0.858<br>(6.270-4.270)
99	*A. brunneus* group (widespread Amazon Basin taxa)	15.059 ± 3.201<br>(9.772-22.215)	16.070 ± 1.830<br>(17.450-13.200)	15.874 ± 2.041<br>(17.390-12.640)	16.070 ± 1.830<br>(17.450-13.200)	16.070 ± 1.830<br>(17.450-13.200)	15.668 ± 1.526<br>(16.760-13.200)	16.020 ± 1.827<br>(17.390-13.150)	16.070 ± 1.830<br>(17.450-13.200)	15.950 ± 1.601<br>(17.170-13.460)
233	Radiation of *Allobates* in the Amazon Basin	19.008 ± 3.704<br>(12.816-27.224)	19.748 ± 2.271<br>(21.450-16.180)	19.510 ± 2.523<br>(21.380-15.510)	19.748 ± 2.271<br>(21.450-16.180)	19.748 ± 2.271<br>(21.450-16.180)	19.264 ± 1.908<br>(20.630-16.180)	19.690 ± 2.265<br>(21.390-16.130)	19.748 ± 2.271<br>(21.450-16.180)	19.612 ± 1.993<br>(21.130-16.510)
86	*A. talamancae* complex	13.485 ± 2.818<br>(8.913-19.922)	11.450 ± 0.910<br>(12.150-10.050)	11.130 ± 1.190<br>(12.010-9.240)	11.450 ± 0.910<br>(12.150-10.050)	11.450 ± 0.910<br>(12.150-10.050)	11.182 ± 0.709<br>(11.700-10.050)	11.414 ± 0.905<br>(12.110-10.020)	11.450 ± 0.910<br>(12.150-10.050)	11.374 ± 0.753<br>(11.970-10.240)
236	*Allobates* (without *A. alagoanus*)	25.699 ± 4.471<br>(18.040-35.391)	26.134 ± 1.436<br>(27.260-23.970)	25.866 ± 1.723<br>(27.180-23.200)	26.134 ± 1.436<br>(27.260-23.970)	26.134 ± 1.436<br>(27.260-23.970)	25.592 ± 1.031<br>(26.360-23.970)	26.054 ± 1.441<br>(27.180-23.880)	26.134 ± 1.436<br>(27.260-23.970)	26.034 ± 1.080<br>(26.930-24.520)
58	*Anomaloglossus*	19.726 ± 3.804<br>(13.166-28.023)	17.418 ± 0.148<br>(17.640-17.230)	17.284 ± 0.123<br>(17.370-17.070)	17.418 ± 0.148<br>(17.640-17.230)	17.418 ± 0.148<br>(17.640-17.230)	17.000 ± 0.380<br>(17.640-16.750)	17.342 ± 0.140<br>(17.540-17.150)	17.418 ± 0.148<br>(17.640-17.230)	17.326 ± 0.422<br>(18.080-17.100)
19	Disperals to Trinidad from Venezuela (*Mannophryne*)	8.300 ± 2.365<br>(4.474-13.648)	6.274 ± 0.009<br>(6.290-6.270)	6.228 ± 0.027<br>(6.250-6.190)	6.274 ± 0.009<br>(6.290-6.270)	6.274 ± 0.009<br>(6.290-6.270)	6.116 ± 0.119<br>(6.290-6.020)	6.254 ± 0.009<br>(6.270-6.250)	6.274 ± 0.009<br>(6.290-6.270)	6.236 ± 0.117<br>(6.440-6.170)
20	*Mannophryne*	11.101 ± 2.790<br>(6.508-17.465)	9.444 ± 0.065<br>(9.560-9.410)	9.380 ± 0.033<br>(9.420-9.330)	9.444 ± 0.065<br>(9.560-9.410)	9.444 ± 0.065<br>(9.560-9.410)	9.216 ± 0.217<br>(9.560-9.060)	9.414 ± 0.065<br>(9.530-9.380)	9.444 ± 0.065<br>(9.560-9.410)	9.398 ± 0.233<br>(9.810-9.270)
6	*Aromobates*	16.960 ± 3.664<br>(10.782-24.975)	15.736 ± 0.095<br>(15.890-15.630)	15.630 ± 0.075<br>(15.680-15.500)	15.736 ± 0.095<br>(15.890-15.630)	15.736 ± 0.095<br>(15.890-15.630)	15.372 ± 0.316<br>(15.890-15.150)	15.684 ± 0.091<br>(15.830-15.580)	15.736 ± 0.095<br>(15.890-15.630)	15.664 ± 0.356<br>(16.300-15.470)
784	Clade A (Allobatinae)	34.921 ± 5.373<br>(25.516-46.683)	34.972 ± 0.342<br>(35.280-34.580)	34.808 ± 0.477<br>(35.220-34.220)	34.972 ± 0.342<br>(35.280-34.580)	34.972 ± 0.342<br>(35.280-34.580)	34.508 ± 0.090<br>(34.630-34.380)	34.896 ± 0.350<br>(35.210-34.500)	34.972 ± 0.342<br>(35.280-34.580)	35.024 ± 0.347<br>(35.510-34.550)
785	Dendrobatidae	40.931 ± 5.360<br>(31.387-52.584)	42.910 ± 0.000<br>(42.910-42.910)	42.910 ± 0.000<br>(42.910-42.910)	42.910 ± 0.000<br>(42.910-42.910)	42.910 ± 0.000<br>(42.910-42.910)	42.910 ± 0.000<br>(42.910-42.910)	42.910 ± 0.000<br>(42.910-42.910)	42.910 ± 0.000<br>(42.910-42.910)	43.322 ± 0.403<br>(44.040-43.110)
