# Supplementary material for: Amazonian Amphibian Diversity Is Primarily Derived from Late Miocene Andean Lineages
Source: PLoS Biol. 2009 Mar 10;7(3):e1000056. doi: 10.1371/journal.pbio.1000056 (PMC2653552; doi:10.1371/journal.pbio.1000056)
Supplement: Supplementary file 12 [file pbio.1000056.st010.pdf]

Table S10. Node time differences between the chronogram age mean and those of the jacknifed constraint estimated chronograms for both r8s and MULTIDIVTIME, bold indicate difference (V), that is 2 SD > V > 1 SD of the TimeSet 1 chronogram. Node numbers form Supplementary Figure S5 A-D.

		MULTIDIVTIME	r8s	r8s without constraint							MULTIDIVTIME "bigtime"		MULTIDIVTIME without constraint						
node	Taxonomic Event	Time Set 1	With All	A	B	C	D	E	F	G	60	80	A	B	C	D	E	F	G
733	*H. azureiventris*/*H. chlorocraspedus* split	5.585 ± 1.592	-0.083	-0.097	-0.083	-0.083	-0.517	-0.097	-0.083	-0.265	-0.010	-0.010	1.395	1.104	0.909	-0.419	0.778	0.809	0.864
673	*H. bocagei* complex	13.198 ± 2.599	-1.924	-1.948	-1.924	-1.924	-2.484	-1.954	-1.924	-2.354	-0.053	-0.053	**2.708**	1.788	1.387	-0.645	1.257	1.177	1.483
781	Clade B (Hyloxinae)	30.918 ± 3.921	-1.432	-1.480	-1.432	-1.432	-2.854	-1.492	-1.432	-2.708	-0.002	-0.002	**7.529**	**5.044**	**4.150**	-1.812	3.673	3.890	**4.214**
599	Dispersal of Epipedobates to Gorgona Island (50 km W of Pacific Coast of Colombia)	2.380 ± 0.748	-0.130	-0.134	-0.130	-0.130	-0.210	-0.134	-0.130	-0.204	-0.008	-0.008	0.500	0.286	0.212	-0.045	0.169	0.183	0.200
623	*D. ventrimaculatus* complex (widespread Amazon Basin	6.545 ± 1.539	-0.361	-0.377	-0.361	-0.361	-0.599	-0.377	-0.361	-0.569	0.011	0.011	1.423	0.826	0.594	-0.100	0.525	0.544	0.594
560	*D. fulguritus* complex	9.312 ± 1.866	-0.136	-0.158	-0.136	-0.136	-0.488	-0.156	-0.136	-0.466	-0.055	-0.055	**2.032**	1.034	0.802	-0.276	0.770	0.773	0.812
534	*D. pumilio* complex	3.018 ± 0.890	-0.444	-0.450	-0.444	-0.444	-0.540	-0.450	-0.444	-0.532	0.006	0.006	0.863	0.514	0.381	-0.045	0.366	0.368	0.413
472	*D. quinquevittatus* complex (widespread Amazon Basin	10.803 ± 2.451	0.677	0.653	0.677	0.677	0.271	0.655	0.677	0.255	0.088	0.088	**2.640**	1.745	1.242	-0.042	1.120	1.194	1.284
627	*Dendrobates*	25.451 ± 3.680	0.307	0.265	0.307	0.307	-0.513	0.265	0.307	-0.809	0.064	0.064	**6.106**	**3.683**	2.721	-0.620	2.456	2.611	2.833
458	*Phyllobates*	12.060 ± 2.753	-2.446	-2.466	-2.446	-2.446	-2.782	-2.464	-2.446	**-2.838**	0.005	0.005	**3.024**	0.276	0.144	-1.049	0.030	0.090	0.134
628	Clade D (Dendrobatinae)	29.914 ± 4.038	0.344	0.300	0.344	0.344	-0.538	0.300	0.344	-1.068	0.026	0.026	**7.096**	3.966	3.034	-1.219	2.604	2.829	3.054
782	Clades B and D ancestor	34.633 ± 4.235	1.561	1.523	1.561	1.561	0.657	1.519	1.561	-0.305	0.037	0.037	**8.183**	**5.112**	4.029	-1.585	3.495	3.801	4.111
417	*A. trivittatus* complex (widespread Amazon Basin species)	1.502 ± 0.511	-0.502	-0.504	-0.502	-0.502	-0.538	-0.502	-0.502	**-0.534**	-0.007	-0.007	0.375	0.171	0.081	-0.029	0.101	0.093	0.103
372	*A. pictus* complex (widespread Amazon Basin taxa)	6.510 ± 1.473	-1.404	-1.418	-1.404	-1.404	**-1.586**	-1.414	-1.404	**-1.566**	-0.018	-0.018	**1.729**	0.716	0.381	-0.171	0.459	0.470	0.467
421	Major radiation of *Ameerega* in the Amazon Basin	7.237 ± 1.620	-1.611	**-1.625**	-1.611	-1.611	**-1.811**	**-1.621**	-1.611	**-1.791**	-0.026	-0.026	**1.961**	0.802	0.440	-0.193	0.520	0.536	0.538
438	*Ameerega*	8.722 ± 1.936	**-2.694**	**-2.708**	**-2.694**	**-2.694**	**-2.906**	**-2.708**	**-2.694**	**-2.892**	-0.041	-0.041	**2.418**	0.982	0.568	-0.255	0.658	0.664	0.670
345	*Colostethus* 2	18.807 ± 2.914	-2.545	-2.581	-2.545	-2.545	**-3.075**	-2.577	-2.545	**-3.167**	0.028	0.028	**5.034**	2.095	1.298	-0.413	1.229	1.305	1.377
326	*Colostethus* 1	13.146 ± 1.324	-1.036	-1.062	-1.036	-1.036	**-1.442**	-1.060	-1.036	**-1.478**	0.027	0.027	**4.595**	0.475	0.349	-0.147	0.315	0.337	0.344
294	*Silverstoneia*	14.884 ± 2.214	-1.742	-1.772	-1.742	-1.742	-2.178	-1.766	-1.742	-2.212	0.036	0.036	**3.037**	2.116	0.828	-0.303	0.725	0.801	0.796
265	*Epipedobates*	5.837 ± 1.514	**-1.805**	**-1.813**	**-1.805**	**-1.805**	**-1.945**	**-1.811**	**-1.805**	**-1.945**	0.046	0.046	**1.516**	0.882	0.476	-0.169	0.452	0.481	0.448
441	Clade C (Colostethinae)	27.090 ± 3.197	-1.662	-1.706	-1.662	-1.662	-2.378	-1.700	-1.662	-2.762	0.061	0.061	**6.824**	3.211	1.946	-0.623	1.728	1.898	1.989
783	Clade B, C and D ancestor	37.037 ± 4.422	1.345	1.315	1.345	1.345	0.669	1.315	1.345	-0.777	0.051	0.051	**8.567**	**5.263**	4.039	-1.460	3.450	3.803	4.127

Table S10 (Cont). Node time differences between the chronogram age mean and those of the jacknifed constraint estimated chronograms for both r8s and MULTIDIVTIME, bold indicate difference (V), that is 2 SD > V > 1 SD of the TimeSet 1 chronogram. Node numbers form Supplementary Figure S5 A-D.

		MULTIDIVTIME	r8s	r8s without constraint							MULTIDIVTIME "bigtime"		MULTIDIVTIME without constraint						
node	Taxonomic Event	Time Set 1	With All	A	B	C	D	E	F	G	60	80	A	B	C	D	E	F	G
226	*A. trilineatus* complex (widespread Amazon Basin	9.887 ± 2.334	1.895	1.767	1.895	1.895	1.575	1.859	1.895	1.787	-0.054	-0.054	1.281	1.140	0.811	-0.058	0.459	0.724	0.804
174	*A. marchesianus* complex (widespread Amazon Basin	8.009 ± 2.054	0.941	0.841	0.941	0.941	0.699	0.915	0.941	0.861	-0.032	-0.032	1.074	0.936	0.712	-0.039	0.405	0.611	0.698
228	Radiation of *Allobates* in the Amazon Basin	12.839 ± 2.852	**3.035**	**2.853**	**3.035**	**3.035**	2.619	**2.987**	**3.035**	**2.901**	-0.045	-0.045	1.702	1.504	1.074	-0.060	0.627	0.966	1.094
149	*A. femoralis* complex (widespread Amazon Basin species)	5.432 ± 1.557	0.250	0.188	0.250	0.250	0.098	0.232	0.250	0.200	-0.042	-0.042	0.724	0.645	0.484	0.007	0.288	0.442	0.463
99	*A. brunneus* group (widespread Amazon Basin	15.059 ± 3.201	1.011	0.815	1.011	1.011	0.609	0.961	1.011	0.891	-0.043	-0.043	2.045	1.774	1.383	-0.106	0.858	1.242	1.386
233	Radiation of *Allobates* in the Amazon Basin	19.008 ± 3.704	0.740	0.502	0.740	0.740	0.256	0.682	0.740	0.604	-0.059	-0.059	2.560	2.247	1.680	-0.114	1.061	1.541	1.726
86	*A. talamancae* complex	13.485 ± 2.818	-2.035	-2.355	-2.035	-2.035	-2.303	-2.071	-2.035	-2.111	-0.048	-0.048	1.222	1.402	1.092	-0.176	0.712	1.014	1.144
236	*Allobates* (without *A. alagoanus*)	25.699 ± 4.471	0.435	0.167	0.435	0.435	-0.107	0.355	0.435	0.335	-0.071	-0.071	3.943	3.206	2.523	-0.283	1.679	2.335	2.594
58	*Anomaloglossus*	19.726 ± 3.804	-2.308	-2.442	-2.308	-2.308	-2.726	-2.384	-2.308	-2.400	-0.055	-0.055	3.801	2.749	2.243	-0.234	1.552	2.094	2.351
19	Disperals to Trinidad from Venezuela (*Mannophryne*)	8.300 ± 2.365	-2.026	-2.072	-2.026	-2.026	-2.184	-2.046	-2.026	-2.064	-0.006	-0.006	1.550	1.159	0.879	-0.139	0.673	0.884	0.957
20	*Mannophryne*	11.101 ± 2.790	-1.657	-1.721	-1.657	-1.657	-1.885	-1.687	-1.657	-1.703	-0.026	-0.026	2.116	1.547	1.216	-0.215	0.909	1.217	1.309
6	*Aromobates*	16.960 ± 3.664	-1.224	-1.330	-1.224	-1.224	-1.588	-1.276	-1.224	-1.296	-0.011	-0.011	3.205	2.341	1.844	-0.281	1.329	1.791	1.937
784	Clade A (Allobatinae)	34.921 ± 5.373	0.051	-0.113	0.051	0.051	-0.413	-0.025	0.051	0.103	-0.012	-0.012	**6.581**	4.765	3.716	-0.517	2.719	3.525	3.916
785	Dendrobatidae	40.931 ± 5.360	1.979	1.979	1.979	1.979	1.979	1.979	1.979	2.391	0.066	0.066	**8.513**	**5.633**	4.344	-1.203	3.536	4.138	4.571
