# Supplementary material for: Amazonian Amphibian Diversity Is Primarily Derived from Late Miocene Andean Lineages
Source: PLoS Biol. 2009 Mar 10;7(3):e1000056. doi: 10.1371/journal.pbio.1000056 (PMC2653552; doi:10.1371/journal.pbio.1000056)
Supplement: Supplementary file 13 [file pbio.1000056.st011.pdf]

Table S11. Species of poison frogs not included in the analyses.

Genera	Species	Author	Distribution	Range (km^2)	Area	Phylogenetic grouping	GAA "Red List"
Allobates	*alessandroi*	Grant and Rodriguez, 2001	Peru: Departamento de Cuzco (820-1480 m)	1324	G	Clade B (*Hyloxalus*)	Data Deficient (DD)
Allobates	*bromelicola*	Test, 1956	Venezuela: Coastal Range (1300 m)	53	D	Clade A (*Allobates*)	Data Deficient (DD)
Allobates	*capixaba*	Bokermann, 1967	Brazil: Espiritu Santos, Linhares (40 m)	429	K	Clade A (*Allobates alagoanus* group)	Data Deficient (DD)
Allobates	*caribe*	Barrio-Amoros, Rivas-Fuenmayor, and Kaiser, 2006	Venezuela: Sucre, Peninsula de Paria (700 m)	--	D	Clade A (*Allobates*)	--
Allobates	*carioca*	Bokermann, 1967	Brazil: Espirito Santo and Rio de Janeiro, Guanabara (500 m)	81	K	Clade A (*Allobates*)	Data Deficient (DD)
Allobates	*cepedai*	Morales, 2002	Colombia: Meta, Villavicencio (600 m)	21	E	Clade A (*Allobates trilineatus/ marchesianus* group)	Data Deficient (DD)
Allobates	*chalcopis*	Kaiser, Coloma, and Gray, 1994	Martinique: Montagne Pelee (500 m)	3	D	Clade A (*Mannophryne*)	Vulnerable (VU)
Allobates	*crombiei*	Morales, 2002	Brazil: Para, Alta Mira do Xingu (10 m)	12906	C	Clade A (*Allobates trilineatus/ marchesianus* group)	Data Deficient (DD)
Allobates	*fratisenescus*	Morales, 2002	Ecuador: Pastaza, Rio Pastaza drainage (1100 m)	141	E	Clade A (*Allobates trilineatus/ marchesianus* group)	Data Deficient (DD)
Allobates	*fuscellus*	Morales, 2002	Brazil, Peru, and Colombia: western Amazon Basin (200-300 m)	676097	C	Clade A (*Allobates trilineatus/ marchesianus* group)	Data Deficient (DD)
Allobates	*goianus*	Bokermann, 1975	Brazil: Goias, Chapada dos Veadeiros (800-1200 m)	24785	K	Clade A (*Allobates alagoanus* group)	Data Deficient (DD)
Allobates	*granti*	Kok, MacCulloch, Gaucher, Poelman, Bourne, Lathrop, and Lenglet, 2006	French Guiana: Saul (100-730 m)	--	B	Clade A (*Allobates trilineatus/ marchesianus* group)	--
Allobates	*mandelorum*	Schmidt, 1932	Venezuela: Turumiquire (2630 m)	576	D	Clade A (*Mannophryne*)	Endangered (EN)
Allobates	*masniger*	Morales, 2002	Brazil: Para, Rio Tapajos (200 m)	10435	C	Clade A (*Allobates trilineatus/ marchesianus* group)	Data Deficient (DD)
Allobates	*mcdiarmidi*	Reynolds and Foster, 1992	Bolivia: La Paz and Cochabamba (1693 m)	32755	GC	Clade B (*Hyloxalus*)	Near Threatened (NT)

Table S11 (Cont). Species of poison frogs not included in the analyses.

Genera	Species	Author	Distribution	Range (km^2)	Area	Phylogenetic grouping	GAA "Red List"
Allobates	*melanolaemus*	Grant and Rodriguez, 2001	Peru: Loreto, lowe Napo drainage (100-200 m)	8508	C	Clade A (*Allobates trilineatus/ marchesianus* group)	Data Deficient (DD)
Allobates	*myersi*	Pyburn, 1981	Colombia: Caqueta and Vaupes (200-300 m)	54875	C	Clade A (*Allobates femoralis* group)	Least Concern (LC)
Allobates	*olfersioides*	Lutz, 1925	Brazil: Minas Gerais and Rio de Janeiro (400 m)	255832	K	Clade A (*Allobates*)	Vulnerable (VU)
Allobates	*picachos*	Ardila-Robayo, Acosta-Galvis, and Coloma, 2000	Colombia: Caqueta, Cordillera los Picachos (1600 m)	--	E	Clade B (*Hyloxalus*)	--
Allobates	*pittieri*	La Marca, Manzanilla, and Mijares-Urrutia, 2004	Venezuela: Cordillera de la Costa (150-1700 m)	10578	D	Clade A (*Allobates trilineatus* group)	Least Concern (LC)
Allobates	*rufulus*	Gorzula, 1990	Venezuela: Bolivar, Chimanta Massif (2600 m)	448	B	Clade A	Data Deficient (DD)
Allobates	*sanmartini*	Rivero, Langone, and Prigioni, 1986	Venezuela: Bolivar, Las Majadas (100 m)	504	B	Clade A (*Allobates trilineatus/ marchesianus* group)	Data Deficient (DD)
Allobates	*spumaponens*	Kok and Ernst, 2007	French Guyana: Mabura Hill Forest Reserve (100 m)	--	B	Clade A (*Allobates trilineatus/ marchesianus* group)	--
Allobates	*subfolionidificans*	Lima, Sanchez, and Souza, 2007	Brazil: Acre, Rio Branco (136 m)	--	C	Clade A (*Allobates trilineatus/ marchesianus* group)	--
Allobates	*sumtuosus*	Morales, 2002	Brazil and Peru: Loreto and Para, north bank of the Amazon (100-200 m)	158431	C	Clade A (*Allobates trilineatus/ marchesianus* group)	Data Deficient (DD)
Allobates	*vanzolinius*	Morales, 2002	Brazil: Amazonas, Rio Jurua (200-300 m)	93031	C	Clade A (*Allobates ornatus* group)	Data Deficient (DD)
Allobates	*wayuu*	Acosta-Galvis, Cuentas, and Coloma, 1999	Colombia: Guajira, Serrania de la Macuira (780 m)	243	I	Clade C (*Colostethus panamensis* group)	Vulnerable (VU)
Aromobates	*alboguttatus*	Boulenger, 1903	Venezuela: Merida, Merida Cordillera (1600-3100 m)	152	D	Clade A (*Aromobates*)	Endangered (EN)
Aromobates	*capurinensis*	Pefaur, 1993	Venezuela: Merida, Merida Cordillera (2350-2700 m)	72	D	Clade A (*Aromobates*)	Data Deficient (DD)
Aromobates	*duranti*	Pefaur, 1985	Venezuela: Merida, Merida Cordillera, La Culata (2600-3000 m)	230	D	Clade A (*Aromobates*)	Endangered (EN)

Table S11 (Cont). Species of poison frogs not included in the analyses.

Genera	Species	Author	Distribution	Range (km^2)	Area	Phylogenetic grouping	GAA "Red List"
Aromobates	*haydeeae*	Rivero, 1978	Venezuela: Tachira, Merida Cordillera, El Zumbador (1825-2670 m)	309	D	Clade A (*Aromobates*)	Endangered (EN)
Aromobates	*leopardalis*	Rivero, 1978	Venezuela: Merida, Merida Cordillera (2435-3300 m)	38	D	Clade A (*Aromobates*)	Critically Endangered (CR)
Aromobates	*mayorgai*	Rivero, 1980	Venezuela: Merida, Merida Cordillera, La Azulita (1700-2400 m)	405	D	Clade A (*Aromobates*)	Endangered (EN)
Aromobates	*meridensis*	Dole and Durant, 1973	Venezuela: Merida, Merida Cordillera, Chorotal (1880-2400 m)	205	D	Clade A (*Aromobates*)	Critically Endangered (CR)
Aromobates	*orostoma*	Rivero, 1978	Venezuela: Tachira, Merida Cordillera,Boca del Monte (2300-2800 m)	116	D	Clade A (*Aromobates*)	Endangered (EN)
Aromobates	*serranus*	Pefaur, 1985	Venezuela: Merida, Merida Cordillera, Libertador (1800-2300 m)	111	D	Clade A (*Aromobates*)	Endangered (EN)
Mannophryne	*caquetio*	Mijares-Urrutia and Arends-R., 1999	Venezuela: Falcon, Sierra de Churuguara (800 m)	83	D	Clade A (*Mannophryne*)	Critically Endangered (CR)
Mannophryne	*cordilleriana*	La Marca, 1995	Venezuela: Merida, Merida Cordillera (1300-1950 m)	92	D	Clade A (*Mannophryne*)	Vulnerable (VU)
Mannophryne	*lamarcai*	Mijares-Urrutia and Arends-R., 1999	Venezuela: Falcon, Cerro Socopo (600-1250 m)	50	D	Clade A (*Mannophryne*)	Critically Endangered (CR)
Mannophryne	*larandina*	Yustiz, 1991	Venezuela: Lara, Sierra de Barbacoas (1800 m)	5	D	Clade A (*Mannophryne*)	Data Deficient (DD)
Mannophryne	*leonardoi*	Manzanilla, La Marca, Jowers, Sanchez, and Garcia-Paris, 2007	Venezuela: Anzoategui, El Toyano (875 m)	--	D	Clade A (*Mannophryne*)	--
Mannophryne	*neblina*	Test, 1956	Venezuela: Aragua, Rancho Grande (900-1100 m)	96	D	Clade A (*Mannophryne*)	Critically Endangered (CR)
Mannophryne	*oblitterata*	Rivero, 1984	Venezuela: Guarico and Miranda, Cordillera de la Costa (150-750 m)	1685	D	Clade A (*Mannophryne*)	Data Deficient (DD)
Mannophryne	*trujillensis*	Vargas Galarce and La Marca, 2007	Venezuela: Trujillo, Trujillo City (850 m)	--	D	Clade A (*Mannophryne*)	--
Mannophryne	*yustizi*	La Marca, 1989	Venezuela: Lara, Sanare-Parque Nacional Yacambu (1200-1800 m)	1160	D	Clade A (*Mannophryne*)	Endangered (EN)
Anomaloglossus	*atopoglossus*	Grant, Humphrey, and Myers, 1997	Colombia: Valle del Cauca, El Boqueron (1800-2260 m)	13	F	?	Data Deficient (DD)
Anomaloglossus	*ayarzaguenai*	La Marca, 1997	Venezuela: Bolivar, Cerro Jaua (1600 m)	178	B	Clade A (*Anomaloglossus*)	Data Deficient (DD)
Anomaloglossus	*breweri*	Barrio-Amoros, 2006	Venezuela: Bolivar, Apradatepui (660 m)	--	B	Clade A (*Anomaloglossus*)	--

Table S11 (Cont). Species of poison frogs not included in the analyses.

Genera	Species	Author	Distribution	Range (km^2)	Area	Phylogenetic grouping	GAA "Red List"
Anomaloglossus	*guanayensis*	La Marca, 1997	Venezuela: Amazonas, Serrania de Guanay (1650-1800 m)	1123	B	Clade A (*Anomaloglossus*)	Data Deficient (DD)
Anomaloglossus	*kaiei*	Kok, Sambhu, Roopsind, Lenglet, and Bourne, 2006	Guyana: Kaieteur National Park (400 m)	--	B	Clade A (*Anomaloglossus*)	--
Anomaloglossus	*lacrimosus*	Myers, 1991	Colombia: Cauca and Choco, Rio Atrato, San Juan, and Guangui drainages (100-640 m)	2421	I	?	Data Deficient (DD)
Anomaloglossus	*murisipanensis*	La Marca, 1997	Venezuela: Bolivar, Murisipan-Tepui (2350 m)	12	B	Clade A (*Anomaloglossus*)	Vulnerable (VU)
Anomaloglossus	*parimae*	La Marca, 1997	Venezuela: Amazonas, Cerro Delgado Chalbaud (670 m)	3	B	Clade A (*Anomaloglossus*)	Data Deficient (DD)
Anomaloglossus	*parkerae*	Meinhardt and Parmalee, 1996	Venezuela: Bolivar, El Dorado (860-1300 m)	863	B	Clade A (*Anomaloglossus*)	Data Deficient (DD)
Anomaloglossus	*shrevei*	Rivero, 1961	Venezuela: Amazonas, Cerro Duida and Marahuaca (350-1829 m)	12987	B	Clade A (*Anomaloglossus*)	Data Deficient (DD)
Anomaloglossus	*tamacuarensis*	Myers and Donnelly, 1997	Venezuela: Amazonas, Pico Tamacuari (1160-1200 m)	539	B	Clade A (*Anomaloglossus*)	Data Deficient (DD)
Anomaloglossus	*triunfo*	Barrio-Amoros, Fuentes-Ramos, and Rivas-Fuenmayor, 2004	Venezuela: Bolivar, Serrania del Supamo (685 m)	10	B	Clade A (*Anomaloglossus*)	Data Deficient (DD)
Anomaloglossus	*wothuja*	Barrio-Amoros, Fuentes-Ramos, and Rivas-Fuenmayor, 2004	Venezuela: Amazonas, Cerro Sipapo (150-200 m)	10	B	Clade A (*Anomaloglossus*)	Data Deficient (DD)
Rheobates	*pseudopalmatus*	Rivero and Serna, 2000	Colombia: Antioquia, Amalfi (Los Auribios) (100-1500 m)	111	IF	Clade A (*Rheobates palmatus*)	Data Deficient (DD)
Ameerega	*andina*	Myers and Burrowes, 1987	Colombia: Narino, Western Andes (700-2020 m)	102	F	?	Data Deficient (DD)
Ameerega	*erythromos*	Vigle and Miyata, 1980	Ecuador: Santo Domingo de los Colorados and Esmeraldas (225-450 m)	33	IF	?	Data Deficient (DD)
Ameerega	*ingeri*	Cochran and Goin, 1970	Colombia: Caqueta and Putumayo, slope of the Eastern Andes(200 m)	16	C	Clade C (*Ameerega*)	Critically Endangered (CR)
Ameerega	*labialis*	Cope, 1874	Peru: Loreto, "Nauta" (equivocal)	--	--	--	Data Deficient (DD)
Ameerega	*maculata*	Peters, 1873	Panama: Chiriqui (equivocal)	--	--	--	Data Deficient (DD)
Ameerega	*peruviridis*	Bauer, 1986	Peru: Ucayali, Ucayali river drainage	--	C	Clade C (*Ameerega*)	--

Table S11 (Cont). Species of poison frogs not included in the analyses.

Genera	Species	Author	Distribution	Range (km^2)	Area	Phylogenetic grouping	GAA "Red List"
Ameerega	*planipaleae*	Morales and Velazco, 1998	Peru: Pasco, Oxapampa, Llamaquizú stream (2,010 m)	222	G	Clade C (*Ameerega*)	Critically Endangered (CR)
Ameerega	*yungicola*	Lotters, Schmitz, and Reichle, 2005	Bolivia: La Paz, Yungas de La Paz(600 m)	--	G	Clade C (*Ameerega*)	--
Colostethus	*agilis*	Lynch and Ruiz-Carranza, 1985	Colombia: Cauca and Valle del Cauca, Serrania de los Paraguas (2190-2600 m)	1066	F	Clade C (*Colostethus*)	Near Threatened (NT)
Colostethus	*alacris*	Rivero and Granados-Diaz, 1990	Colombia: Cauca, El Tambo (1400 m)	28	F	Clade C (*Colostethus*)	Data Deficient (DD)
Colostethus	*brachistriatus*	Rivero and Serna, 1986	Colombia: Valle del Cauca, Cordillera Central (1500 m)	79	F	Clade C (*Colostethus*)	Data Deficient (DD)
Colostethus	*dysprosium*	Rivero and Serna, 2000	Colombia: Antioquia, Urrao (1300-1400 m)	68	I	Clade C (*Colostethus*)	Data Deficient (DD)
Colostethus	*furviventris*	Rivero and Serna, 1991	Colombia: Antioquia, Antado (1500 m)	132	F	Clade C (*Colostethus*)	Data Deficient (DD)
Colostethus	*jacobuspetersi*	Rivero, 1991	Ecuador: Imbabura, Pichincha, Cotopaxi, Chimborazo, and Cañar, Inter-Andean valleys of Guayllabamba, Toachi, Chimbo, and Cañar, on the western slopes of the Andes (1500-3800 m)	5038	FE	Clade B (*Hyloxalus*)	Critically Endangered (CR)
Colostethus	*lynchi*	Grant, 1998	Colombia: Antioquia, Apartado (30 m)	73	I	?	Data Deficient (DD)
Colostethus	*mertensi*	Cochran and Goin, 1964	Colombia: Cauca, Popayan (2100-2350 m)	750	F	Clade C (*Colostethus*)	Endangered (EN)
Colostethus	*ruthveni*	Kaplan, 1997	Colombia: Magdalena, Santa Marta (680-1540 m)	368	I	Clade C (*Colostethus*)	Endangered (EN)
Colostethus	*thorntoni*	Cochran and Goin, 1970	Colombia: Antioquia, Cordillera Central (1480-2270 m)	2678	F	Clade C (*Colostethus*)	Data Deficient (DD)
Colostethus	*ucumari*	Grant, 2007	Colombia: Risaralda, Cordillera Central, Ucumari (2100-2500 m)	--	F	Clade C (*Colostethus*)	--
Colostethus	*yaguara*	Rivero and Serna, 1991	Colombia: Antioquia, Ituango (1575 m)	108	F	Clade C (*Colostethus*)	Data Deficient (DD)
Silverstoneia	*erasmios*	Rivero and Serna, 2000	Colombia: Antioquia, Urrao (1500-2000 m)	784	F	Clade C (*Silverstoneia)*	Data Deficient (DD)
Hyloxalus	*abditaurantius*	Silverstone, 1975	Colombia: Quindio and Risaralda,Cordillera Occidental and Central (1450-2000 m)	1088	F	Clade B (*Hyloxalus*)	Least Concern (LC)

Table S11 (Cont). Species of poison frogs not included in the analyses.

Genera	Species	Author	Distribution	Range (km^2)	Area	Phylogenetic grouping	GAA "Red List"
Hyloxalus	*aeruginosus*	Duellman, 2004	Peru: San Martin and Rioja, Abra Pardo de Miguel (2180 m)	--	G	Clade B (*Hyloxalus*)	--
Hyloxalus	*betancuri*	Rivero and Serna, 1991	Colombia: Antioquia, Ituango (1450 m)	149	F	Clade B (*Hyloxalus*)	Data Deficient (DD)
Hyloxalus	*borjai*	Rivero and Serna, 2000	Colombia: Antioquia, Amalfi (1000-1500 m)	211	F	Clade B (*Hyloxalus*)	Data Deficient (DD)
Hyloxalus	*breviquartus*	Rivero and Serna, 1986	Ecuador and Colombia: Cordillera Occidental (1700-1800 m)	132	F	Clade B (*Hyloxalus*)	Data Deficient (DD)
Hyloxalus	*cevallosi*	Rivero, 1991	Ecuador: Pastaza, eastern side of the Andes (480-970 m)	640	G	Clade C (*Colostethus*)	Endangered (EN)
Hyloxalus	*chocoensis*	Boulenger, 1912	Ecuador, Panama, and Colombia: Chocoan rainforest (100 m)	3493	I	Clade B (*Hyloxalus*)	Data Deficient (DD)
Hyloxalus	*edwardsi*	Lynch, 1982	Colombia: Cundinamarca, Cordillera Oriental (3030-3300 m)	49	E	?	Critically Endangered (CR)
Hyloxalus	*eleutherodactylus*	Duellman, 2004	Peru: San Martin,Shapaja (360 m)	--	G	Clade B (*Hyloxalus*)	--
Hyloxalus	*exasperatus*	Duellman and Lynch, 1988	Ecuador: Morona Santiago, Cordillera de Cutucu (970-1981 m)	964	G	Clade B (*Hyloxalus*)	Data Deficient (DD)
Hyloxalus	*excisus*	Rivero and Serna, 2000	Colombia: Antioquia, Enviagado (2200 m)	271	F	?	Data Deficient (DD)
Hyloxalus	*faciopunctulatus*	Rivero, 1991	Colombia: Amazonas, Puerto Narinio (100-200 m)	869	C	Clade B (*Hyloxalus*)	Data Deficient (DD)
Hyloxalus	*fallax*	Rivero, 1991	Ecuador: Cotopaxi, Pilalo (1760-2430 m)	310	F	Clade B (*Hyloxalus*)	Data Deficient (DD)
Hyloxalus	*fascianigrus*	Grant and Castro-Herrera, 1998	Colombia: Valle del Cauca, Dagua (1600-1960 m)	--	F	Clade B (*Hyloxalus*)	--
Hyloxalus	*fuliginosus*	Jimenez de la Espada, 1871	Ecuador: Napo, Sumaco Volcano (1660-1910 m)	602	E	Clade B (*Hyloxalus*)	Data Deficient (DD)
Hyloxalus	*lehmanni*	Silverstone, 1971	Ecuador and Colombia: Eastern slopes of the Central Andes (1460-2120 m)	77369	F	Clade B (*Hyloxalus*)	Near Threatened (NT)
Hyloxalus	*littoralis*	Pefaur, 1984	Peru: Lima, Huanuco and Ancash, Central Occidental Andes (665-2800 m)	5031	H	Clade B (*Hyloxalus*)	Least Concern (LC)

Table S11 (Cont). Species of poison frogs not included in the analyses.

Genera	Species	Author	Distribution	Range (km^2)	Area	Phylogenetic grouping	GAA "Red List"
Hyloxalus	*maquipucuna*	Coloma, 1995	Ecuador: Pichincha, Tandayapa (2390 m)	13	F	Clade B (*Hyloxalus*)	Data Deficient (DD)
Hyloxalus	*marmoreoventris*	Rivero, 1991	Ecuador: Tungurahua, RIo Negro (1225 m)	5	G	Clade B (*Hyloxalus*)	Data Deficient (DD)
Hyloxalus	*mittermeieri*	Rivero, 1991	Peru: San Martin, Venceremos (1620 m)	31	G	Clade B (*Hyloxalus*)	Data Deficient (DD)
Hyloxalus	*mystax*	Duellman and Simmons, 1988	Ecuador: Morona Santiago, Cordillera del Condor, Rio Piuntza (1830 m)	4	G	Clade B (*Hyloxalus*)	Data Deficient (DD)
Hyloxalus	*parcus*	Rivero, 1991	Ecuador: Zamora Chinchipe, San Juan Bosco (1981 m)	--	G	Clade B (*Hyloxalus*)	--
Hyloxalus	*patitae*	Lotters, Morales, and Proy, 2003	Peru: Pasco, Cordillera El Sira (210 m)	--	C	Clade B (*Hyloxalus*)	Data Deficient (DD)
Hyloxalus	*peculiaris*	Rivero, 1991	Ecuador: Morona Santiago, Pailas (2195 m)	2	G	Clade B (*Hyloxalus*)	Data Deficient (DD)
Hyloxalus	*pinguis*	Rivero and Granados-D√≠az, 1990	Colombia: Cauca, Totoro, Malvasa (2995 m)	164	F	Clade B (*Hyloxalus*)	Data Deficient (DD)
Hyloxalus	*pumilus*	Rivero, 1991	Ecuador: Azuay, Limon (2987 m)	3	G	Clade B (*Hyloxalus*)	Data Deficient (DD)
Hyloxalus	*ramosi*	Silverstone, 1971	Colombia: Antioquia, Alto de la Honda (1240 m)	1345	F	Clade B (*Hyloxalus*)	Data Deficient (DD)
Hyloxalus	*ruizi*	Lynch, 1982	Colombia: Cundinamarca, Fusagasuga (2410-2469 m)	81	E	?	Critically Endangered (CR)
Hyloxalus	*saltuarius*	Grant and Ardila-Robayo, 2002	Colombia: Caqueta, Guayabal (1600 m)	74	E	Clade B (*Hyloxalus*)	Data Deficient (DD)
Hyloxalus	*shuar*	Duellman and Simmons, 1988	Ecuador: Cordillera Oriental (1272-2370 m)	8426	EG	Clade B (*Hyloxalus*)	Near Threatened (NT)
Hyloxalus	*spilotogaster*	Duellman, 2004	Peru: Amazonas, La Peca (2326 m)	--	G	Clade B (*Hyloxalus*)	--
Hyloxalus	*utcubambensis*	Morales, 1994	Peru: Amazonas, Rio Utcubamba (2391 m)	272	G	Clade B (*Hyloxalus*)	Data Deficient (DD)
Hyloxalus	*vergeli*	Hellmich, 1940	Colombia: Cundinamarca, Fusagasuga (1500-1800 m)	78	E	Clade B (*Hyloxalus*)	Vulnerable (VU)

Table S11 (Cont). Species of poison frogs not included in the analyses.

Genera	Species	Author	Distribution	Range (km^2)	Area	Phylogenetic grouping	GAA "Red List"
Hyloxalus	*whymperi*	Boulenger, 1882	Ecuador: Pichincha, Tanti (607 m)	34	F	Clade B (*Hyloxalus*)	Data Deficient (DD)
Dendrobates	*nubeculosus*	Jungfer and B√∂hme, 2004	Guyana: Mazaruni Potaro District, Essequibo River (7 m)	10	B	Clade D (*Dendrobates*)	Data Deficient (DD)
Dendrobates	*occultator*	Myers and Daly, 1976	Colombia: Cauca, Rio Saija (50-200 m)	126	I	Clade D (*Dendrobates*)	Data Deficient (DD)
Dendrobates	*abditus*	Myers and Daly, 1976	Ecuador: Napo, Rio Azuela, Reventador (1700 m)	7	E	Clade D (*Dendrobates*)	Critically Endangered (CR)
Dendrobates	*altobueyensis*	Silverstone, 1975	Colombia: Choco, Alto del Buey (980-1070 m)	90	I	Clade D (*Dendrobates*)	Vulnerable (VU)
Dendrobates	*daleswansoni*	Rueda-Almonacid, Rada, Sanchez-Pacheco, Velasquez-Alvarez, and Quevedo-Gil, 2006	Colombia: Tolima, Samana (1950 m)	--	F	Clade D (*Dendrobates*)	--
Dendrobates	*dorisswansoni*	Rueda-Almonacid, Rada, Sanchez-Pacheco, Velasquez-Alvarez, and Quevedo-Gil, 2006	Colombia: Tolima, Falan (1780 m)	--	F	Clade D (*Dendrobates*)	--
Dendrobates	*igneus*	Melin, 1941	Peru: Loreto, Iquitos, Rio Itaya (100 m)	--	C	Clade D (*Dendrobates*)	--
Dendrobates	*intermedius*	Schulte, 1999	Peru: San Martin, Huallaga Canyon (200 m)	--	C	Clade D (*Dendrobates*)	--
Dendrobates	*opisthomelas*	Boulenger, 1899	Colombia: Antioquia, Santa Ines (1160-2200 m)	3472	F	Clade D (*Dendrobates*)	Vulnerable (VU)
Dendrobates	*rubrocephalus*	Schulte, 1999	Peru: Pasco and Junin, Ceja de Selva (600-1500 m)	--	G	Clade D (*Dendrobates*)	Data Deficient (DD)
Dendrobates	*sirensis*	Aichinger, 1991	Peru: Huanuco, Serrania de Sira (750-1000 m)	181	G	Clade D (*Dendrobates*)	Endangered (EN)
Dendrobates	*viridis*	Myers and Daly, 1976	Colombia: Valle del Cauca, Dagua (100-1200 m)	4966	IF	Clade D (*Dendrobates*)	Vulnerable (VU)
