# Supplementary material for: Amazonian Amphibian Diversity Is Primarily Derived from Late Miocene Andean Lineages
Source: PLoS Biol. 2009 Mar 10;7(3):e1000056. doi: 10.1371/journal.pbio.1000056 (PMC2653552; doi:10.1371/journal.pbio.1000056)
Supplement: Supplementary file 14 [file pbio.1000056.st012.pdf]

Table S12. Poison frog tree dispersal events indicated in Figure 2 with their estimated divergence times and node number from the ML phylogeny. Node numbers form Supplementary Figure S5 A-D.

Event	Origin	Destination	Figure 3 Code				Time Estimate	Node
			Amazon Basin	Andes	Guiana-Venezuela	C America-Chocó	Constratins Set 1	
G → C	Central Oriental Andes	Amazon Basin	green box (1)	--	--	--	6.545 ± 1.539 (3.971-9.943)	623
G → C	Central Oriental Andes	Amazon Basin	green box (2)	--	--	--	2.447 ± 0.798 (1.201-4.322)	571
E → C	North Oriental Andes	Amazon Basin	green box (3)	--	--	--	21.145 ± 3.346 (15.192-28.071)	539
G → C	Central Oriental Andes	Amazon Basin	green box (4)	--	--	--	5.585 ± 1.592 (3.070-9.225)	733
E → C	North Oriental Andes	Amazon Basin	green box (5)	--	--	--	4.735 ± 1.363 (2.596-7.849)	671
E → C	North Oriental Andes	Amazon Basin	green box (6)	--	--	--	1.593 ± 0.587 (0.682-2.977)	646
E → C	North Oriental Andes	Amazon Basin	green box (7)	--	--	--	5.129 ± 1.384 (2.938-8.325)	658
E → C	North Oriental Andes	Amazon Basin	green box (8)	--	--	--	7.355 ± 1.658 (5.128-11.369)	647
G → C	Central Oriental Andes	Amazon Basin	green box (9)	--	--	--	6.110 ± 1.407 (3.767-9.226)	418
G → C	Central Oriental Andes	Amazon Basin	green box (10)	--	--	--	7.237 ± 1.620 (4.522-10.811)	421
G → C	Central Oriental Andes	Amazon Basin	green box (11)	--	--	--	6.745 ± 1.613 (4.101-10.406)	433
G → C	Central Oriental Andes	Amazon Basin	green box (12)	--	--	--	3.109 ± 0.993 (1.535-5.395)	437
B → C	Guiana	Amazon Basin	green box (13)	--	--	--	23.753 ± 4.179 (16.638-32.958)	235
B → C	Guiana	Amazon Basin	green box (14)	--	--	--	15.525 ± 3.387 (9.763-22.980)	56
C → B	Amazon Basin	Guiana	grey box (1)	--	*ad* 10	--	6.039 ± 1.430 (3.635-9.201)	600
C → G	Amazon Basin	Central Oriental Andes	grey box (2)	--	--	--	4.436 ± 1.149 (2.556-7.075)	622

Table S12 (Cont). Poison frog tree dispersal events indicated in Figure 2 with their estimated divergence times and node number from the ML phylogeny. Node numbers form Supplementary Figure S5 A-D.

Event	Origin	Destination	Figure 3 Code				Time Estimate	Node
			Amazon Basin	Andes	Guiana-Venezuela	C America-Chocó	Constratins Set 1	
C → B	Amazon Basin	Guiana	grey box (3)	--	*ad* 9	--	10.542 ± 2.056 (6.913-15.005)	506
C → B	Amazon Basin	Guiana	grey box (4)	--	*ad* 8	--	10.803 ± 2.451 (6.692-16.236)	472
C → B	Amazon Basin	Guiana	grey box (5)	--	*ad* 7	--	1.263 ± 0.456 (0.533-2.324)	411
C → B	Amazon Basin	Guiana	grey box (6)	--	*ad* 6	--	5.963 ± 1.406 (3.639-9.082)	368
C → K	Amazon Basin	Brazilian Shield	grey box (7)	--	--	--	3.818 ± 1.067 (2.077-6.256)	371
C → G	Amazon Basin	North Oriental Andes	grey box (8)	--	--	--	2.427 ± 0.804 (1.170-4.287)	349
C → B	Amazon Basin	Guiana	grey box (9)	--	*ad* 5	--	2.427 ± 0.804 (1.170-4.287)	351
C → D	Amazon Basin	Venezuela Highlands	grey box (10)	--	*ad* 4	--	8.833 ± 2.135 (5.386-13.722)	214
C → B	Amazon Basin	Guiana	grey box (11)	--	*ad* 3	--	3.873 ± 1.125 (2.104-6.439)	224
C → E	Amazon Basin	North Oriental Andes	grey box (12)	--	--	--	1.164 ± 0.444 (0.514-2.230)	195
C → B	Amazon Basin	Guiana	grey box (13)	--	*ad* 2	--	5.143 ± 1.441 (2.869-8.517)	173
C → E	Amazon Basin	North Oriental Andes	grey box (14)	--	--	--	15.159 ± 3.202 (9.820-22.365)	230
C → B	Amazon Basin	Guiana	grey box (15)	--	*ad* 1	--	1.098 ± 0.426 (0.448-2.118)	144
C → G	Amazon Basin	Central Oriental Andes	grey box (16)	--	--	--	0.772 ± 0.323 (0.298-1.559)	121
C → G	Amazon Basin	Central Oriental Andes	grey box (17)	--	--	--	10.622 ± 2.512 (6.519-16.133)	114
C → E	Amazon Basin	North Oriental Andes	grey box (18)	--	--	--	21.836 ± 3.942 (15.241-30.623)	234

Table S12 (Cont). Poison frog tree dispersal events indicated in Figure 2 with their estimated divergence times and node number from the ML phylogeny. Node numbers form Supplementary Figure S5 A-D.

Event	Origin	Destination	Figure 3 Code				Time Estimate	Node
			Amazon Basin	Andes	Guiana-Venezuela	C America-Chocó	Constratins Set 1	
E → F	North Oriental Andes	North Occiden. Andes	--	*ca* 1	--	--	3.466 ± 1.090 (1.788-6.022)	335
G → H	Central Oriental Andes	Central Occiden. Andes	--	*ca* 2	--	--	25.429 ± 3.406 (19.415-32.640)	780
E → F	North Oriental Andes	North Occiden. Andes	--	ca 3	--	--	10.986 ± 2.089 (7.436-15.621)	713
G → H	Central Oriental Andes	Central Occiden. Andes	--	*ca* 4	--	--	11.465 ± 2.248 (7.595-16.372)	775
E → F	North Oriental Andes	North Occiden. Andes	--	*ca* 5	--	--	2.010 ± 0.724 (0.873-3.677)	549
E → G	North Oriental Andes	Central Oriental Andes	--	*ns* 1	--	--	21.681 ± 3.007 (16.087-27.900)	439
E → G	North Oriental Andes	Central Oriental Andes	--	*ns* 2	--	--	30.918 ± 3.921 (23.938-39.216)	781
E → G	North Oriental Andes	Central Oriental Andes	--	*ns* 3	--	--	16.805 ± 2.914 (11.752-23.127)	626
G → E	Central Oriental Andes	North Oriental Andes	--	*ns* 4	--	--	21.360 ± 3.266 (15.586-28.387)	693
G → E	Central Oriental Andes	North Oriental Andes	--	*ns* 5	--	--	14.930 ± 2.277 (11.031-19.897)	714
I → J	Chocó	Central America	--	--	--	*d1*	12.060 ± 2.753 (7.427-18.132)	458
E → IJ	North Oriental Andes	Chocó/C. America	--	--	--	*d*1/*ai* 5	8.331 ± 1.992 (4.962-12.728)	537
I → J	Chocó	Central America	--	--	--	*d2*	5.388 ± 1.086 (3.447-7.706)	308
I → J	Chocó	Central America	--	--	--	*d2*	4.732 ± 1.115 (3.044-7.388)	559

Table S12 (Cont). Poison frog tree dispersal events indicated in Figure 2 with their estimated divergence times and node number from the ML phylogeny. Node numbers form Supplementary Figure S5 A-D.

Event	Origin	Destination	Figure 3 Code				Time Estimate	Node
			Amazon Basin	Andes	Guiana-Venezuela	C America-Chocó	Constratins Set 1	
I → J	Chocó	Central America	--	--	--	*d2*	4.519 ± 0.822 (3.013-6.302)	273
I → J	Chocó	Central America	--	--	--	*d2*	4.149 ± 0.988 (2.593-6.356)	292
I → J	Chocó	Central America	--	--	--	*d2*	3.370 ± 0.679 (2.455-4.964)	322
I → J	Chocó	Central America	--	--	--	*d2*	3.172 ± 0.702 (2.426-5.058)	84
I → J	Chocó	Central America	--	--	--	*d3*	2.153 ± 0.586 (1.286-3.591)	83
I → J	Chocó	Central America	--	--	--	*d3*	0.578 ± 0.254 (0.136-1.126)	502
E → I	North Oriental Andes	Chocó	--	--	--	*ai* 1	13.485 ± 2.818 (8.913-19.922)	86
E → I	North Oriental Andes	Chocó	--	--	--	*ai* 2	27.090 ± 3.197 (21.248-33.546)	441
E → I	North Oriental Andes	Chocó	--	--	--	*ai* 3	10.085 ± 1.971 (6.785-14.477)	561
E → I	North Oriental Andes	Chocó	--	--	--	*ai* 4	29.914 ± 4.038 (22.662-38.326)	628
H → I	Central Occiden. Andes	Chocó	--	--	--	*ai* 6	9.440 ± 1.965 (6.122-13.767)	774
I → F	Chocó	North Occiden. Andes	--	--	--	*ia* 1	1.125 ± 0.473 (0.376-2.237)	260
I → H	Chocó	Central Occiden. Andes	--	--	--	*ia* 2	1.415 ± 0.547 (0.573-2.711)	262
I → F	Chocó	North Occiden. Andes	--	--	--	*ia* 3	2.723 ± 0.885 (1.337-4.804)	263

Table S12 (Cont). Poison frog tree dispersal events indicated in Figure 2 with their estimated divergence times and node number from the ML phylogeny. Node numbers form Supplementary Figure S5 A-D.

Event	Origin	Destination	Figure 3 Code				Time Estimate	Node
			Amazon Basin	Andes	Guiana-Venezuela	C America-Chocó	Constratins Set 1	
I → F	Chocó	North Occiden. Andes	--	--	--	*ia* 4	2.490 ± 0.814 (1.221-4.383)	518
I → F	Chocó	North Occiden. Andes	--	--	--	*ia* 5	6.640 ± 1.620 (3.954-10.321)	771
D → B	Venezuela Highlands	Guiana	--	--	--	--	31.247 ± 4.973 (22.625-42.240)	237
D → E	Venezuela Highlands	North Oriental Andes	--	--	--	--	10.660 ± 2.891 (5.955-17.245)	5
D → E	Venezuela Highlands	North Oriental Andes	--	--	--	--	3.135 ± 1.047 (1.474-5.584)	31
J → I	Central America	Chocó	--	--	--	--	0.916 ± 0.382 (0.286-1.808)	81
