# Supplementary material for: Amazonian Amphibian Diversity Is Primarily Derived from Late Miocene Andean Lineages
Source: PLoS Biol. 2009 Mar 10;7(3):e1000056. doi: 10.1371/journal.pbio.1000056 (PMC2653552; doi:10.1371/journal.pbio.1000056)
Supplement: Supplementary file 15 [file pbio.1000056.st013.pdf]

Table S13. Poison frog species group and taxonomic diversity assigned to the diversification rate analyses and Figure 3. The species sampled for the analyses are indicated by "*".

Group	Genera	Species
Aromobates (*n* = 12)	*Aromobates*	*alboguttatus*
	Aromobates	*capurinensis*
	Aromobates	*duranti*
	Aromobates	*haydeeae*
	Aromobates	*leopardalis*
	Aromobates	*mayorgai*
	Aromobates	*meridensis*
	Aromobates	*molinarii**
	Aromobates	*nocturnus**
	Aromobates	*orostoma*
	Aromobates	*saltuensis**
	Aromobates	*serranus*
Mannophryne (n = 15)	*Mannophryne*	*caquetio*
	Mannophryne	*collaris**
	Mannophryne	*cordilleriana*
	Mannophryne	*herminae**
	Mannophryne	*lamarcai*
	Mannophryne	*larandina*
	Mannophryne	*leonardoi*
	Mannophryne	*neblina*
	Mannophryne	*oblitterata*
	Mannophryne	*olmonae**
	Mannophryne	*riveroi**
	Mannophryne	*trinitatis**
	Mannophryne	*trujillensis*
	Mannophryne	*venezuelensis**
	Mannophryne	*yustizi*
Anomaloglossus (n = 18)	*Anomaloglossus*	*ayarzaguenai*
	Anomaloglossus	*baeobatrachus**
	Anomaloglossus	*beebei**
	Anomaloglossus	*breweri*
	Anomaloglossus	*degranvillei**
	Anomaloglossus	*guanayensis*
	Anomaloglossus	*kaiei*
	Anomaloglossus	*murisipanensis*
	Anomaloglossus	*parimae*
	Anomaloglossus	*parkerae*
	Anomaloglossus	*praderioi**
	Anomaloglossus	*roraima**
	Anomaloglossus	*shrevei*
	Anomaloglossus	*stepheni**
	Anomaloglossus	*tamacuarensis*
	Anomaloglossus	*tepuyensis**
	Anomaloglossus	*triunfo*
	Anomaloglossus	*wothuja*

Table S13 (Cont). Poison frog species group and taxonomic diversity assigned to the diversification rate analyses and Fig. 3. The species sampled for the analyses are indicated "*".

Group	Genera	Species
Rheobates (n = 2)	*Rheobates*	*palmatus**
	Rheobates	*pseudopalmatus*
Allobates sp. "Neblina" (n = 3)	*Allobates*	*alagoanus*
	Allobates	*capixaba*
	Allobates	sp. "Neblina"*
Allobates undulatus (n = 1)	*Allobates*	*undulatus**
Allobates talamancae (n = 2)	*Allobates*	*talamancae**
	Allobates	*niputidea**
Allobates nidicola (n = 2)	*Allobates*	*nidicola**
	Allobates	*brunneus**
Allobates kingsburyi (n = 2)	*Allobates*	*kingsburyi**
	Allobates	*peruvianus**
Allobates femoralis (n = 3)	*Allobates*	*femoralis**
	Allobates	*zaparo**
	Allobates	*myersi*
Allobates juanii (n = 1)	*Allobates*	*juanii**
Allobates marchesianus (n = 12)	*Allobates*	*cepedai*
	Allobates	*chalcopis*
	Allobates	*fratisenescus*
	Allobates	*fuscellus*
	Allobates	*marchesianus**
	Allobates	*masniger*
	Allobates	*picachos*
	Allobates	*pittieri*
	Allobates	*rufulus*
	Allobates	*sanmartini*
	Allobates	*subfolionidificans*
	Allobates	*wayuu*
Allobates ornatus (n = 4)	*Allobates*	*conspicuus**
	Allobates	*insperatus**
	Allobates	*ornatus**
	Allobates	*vanzolinius*
Allobates gasconi (n = 3)	*Allobates*	*gasconi**
	Allobates	*olfersioides*
	Allobates	*goianus*
Allobates trilineatus (n = 11)	*Allobates*	*alessandroi*
	Allobates	*bromelicola*
	Allobates	*caribe*
	Allobates	*carioca*
	Allobates	*crombiei*
	Allobates	*humilis*
	Allobates	*mandelorum*
	Allobates	*melanolaemus*
	Allobates	*spumaponens*
	Allobates	*sumtuosus*
	Allobates	*trilineatus**

Table S13 (Cont). Poison frog species group and taxonomic diversity assigned to the diversification rate analyses and Fig. 3. The species sampled for the analyses are indicated "*".

Group	Genera	Species
Allobates caeruleodactylus (n = 1)	*Allobates*	*caeruleodactylus**
Epipedobates (n = 5)	*Epipedobates*	*anthonyi**
	Epipedobates	*boulengeri**
	Epipedobates	*espinosai**
	Epipedobates	*machalilla**
	Epipedobates	*tricolor**
Silverstoneia (n = 3)	*Silverstoneia*	*erasmios*
	Silverstoneia	*flotator**
	Silverstoneia	*nubicola**
Colostethus pratti (n = 2)	*Colostethus*	*latinasus**
	Colostethus	*pratti**
Colostethus inguinalis (n = 17)	*Colostethus*	*agilis*
	Colostethus	*alacris*
	Colostethus	*brachistriatus*
	Colostethus	*dunni*
	Colostethus	*dysprosium*
	Colostethus	*furviventris*
	Colostethus	*imbricolus**
	Colostethus	*inguinalis**
	Colostethus	*jacobuspetersi*
	Colostethus	*mertensi*
	Colostethus	*panamensis**
	Colostethus	*poecilonotus*
	Colostethus	*ramirezi*
	Colostethus	*ruthveni*
	Colostethus	*thorntoni*
	Colostethus	*ucumari*
	Colostethus	*yaguara*
*Colostethus*_2 (n = 3)	*Colostethus*	*argyrogaster**
	Colostethus	*fraterdanieli**
	Colostethus	*fugax**
Ameerega hahneli (n = 22)	*Ameerega*	*altamazonica*
	Ameerega	*bassleri**
	Ameerega	*bilinguis**
	Ameerega	*boliviana*
	Ameerega	*braccata**
	Ameerega	*cainarachi**
	Ameerega	*flavopicta**
	Ameerega	*hahneli**
	Ameerega	*ingeri*
	Ameerega	*macero**
	Ameerega	*peruviridis*
	Ameerega	*petersi**
	Ameerega	*picta**
	Ameerega	*planipaleae*
	Ameerega	*pongoensis**

Table S13 (Cont). Poison frog species group and taxonomic diversity assigned to the diversification rate analyses and Fig. 3. The species sampled for the analyses are indicated "*".

Group	Genera	Species
Ameerega hahneli (n = 22)	*Ameerega*	*pulchripecta**
	Ameerega	*rubriventris**
	Ameerega	*silverstonei**
	Ameerega	*simulans**
	Ameerega	*smaragdina**
	Ameerega	*trivittata**
	Ameerega	*yungicola*
Ameerega parvula (n = 1)	*Ameerega*	*parvula**
Hyloxalus leucophaeus (n = 4)	*Hyloxalus*	*aeruginosus*
	Hyloxalus	*leucophaeus**
	Hyloxalus	*mittermeieri*
	Hyloxalus	*sordidatus**
Hyloxalus subpuctatus (n = 2)	*Hyloxalus*	*ranoides**
	Hyloxalus	*subpunctatus**
Hyloxalus bocagei (n = 6)	*Hyloxalus*	*abditaurantius*
	Hyloxalus	*bocagei**
	Hyloxalus	*fuliginosus*
	Hyloxalus	*maculosus**
	Hyloxalus	*sauli**
	Hyloxalus	*vergeli*
Hyloxalus azureiventris (n = 2)	*Hyloxalus*	*azureiventris**
	Hyloxalus	*chlorocraspedus**
Hyloxalus craspedoceps (n = 2)	*Hyloxalus*	*craspedoceps**
	Hyloxalus	*eleutherodactylus*
Hyloxalus idiomelus (n = 1)	*Hyloxalus*	*idiomelus**
Hyloxalus insulatus (n = 3)	*Hyloxalus*	*cevallosi*
	Hyloxalus	*insulatus**
	Hyloxalus	*peculiaris*
Hyloxalus nexipus (n = 3)	*Hyloxalus*	*nexipus**
	Hyloxalus	*mcdiarmidi*
	Hyloxalus	*patitae*
Hyloxalus pulcherrimus (n = 7)	*Hyloxalus*	*exasperatus*
	Hyloxalus	*faciopunctulatus*
	Hyloxalus	*pulcherrimus**
	Hyloxalus	*saltuarius*
	Hyloxalus	*spilotogaster*
	Hyloxalus	*sylvaticus**
	Hyloxalus	*utcubambensis*
Hyloxalus sp. "El Copal" (n = 4)	*Hyloxalus*	sp. "El Copal"*
	Hyloxalus	*fallax*
	Hyloxalus	*marmoreoventris*
	Hyloxalus	*pumilus*
Hyloxalus pulchellus (n = 4)	*Hyloxalus*	*delatorreae**
	Hyloxalus	*pulchellus**
	Hyloxalus	*mystax*
	Hyloxalus	*shuar*

Table S13 (Cont). Poison frog species group and taxonomic diversity assigned to the diversification rate analyses and Fig. 3. The species sampled for the analyses are indicated "*".

Group	Genera	Species
Hyloxalus anthracinus (n = 10)	*Hyloxalus*	*anthracinus**
	Hyloxalus	*betancuri*
	Hyloxalus	*borjai*
	Hyloxalus	*breviquartus*
	Hyloxalus	*chocoensis*
	Hyloxalus	*fascianigrus*
	Hyloxalus	*lehmanni*
	Hyloxalus	*maquipucuna*
	Hyloxalus	*pinguis*
	Hyloxalus	*ramosi*
Hyloxalus vertebralis (n = 1)	*Hyloxalus*	*vertebralis**
Hyloxalus elachyhistus (n = 3)	*Hyloxalus*	*elachyhistus**
	Hyloxalus	*littoralis*
	Hyloxalus	*infraguttatus**
Hyloxalus awa (n = 3)	*Hyloxalus*	*awa**
	Hyloxalus	*toachi**
	Hyloxalus	*whymperi*
Hyloxalus sp. "MasValle" (n = 1)	*Hyloxalus*	sp. "MasValle"*
Phyllobates (n = 5)	*Phyllobates*	*aurotaenia**
	Phyllobates	*bicolor**
	Phyllobates	*lugubris**
	Phyllobates	*terribilis**
	Phyllobates	*vittatus**
Dendrobates galactonotus (n = 4)	*Dendrobates*	*castaneoticus**
	Dendrobates	*galactonotus**
	Dendrobates	*quinquevittatus**
	Dendrobates	*steyermarki**
Dendrobates tinctorius (n = 5)	*Dendrobates*	*auratus**
	Dendrobates	*leucomelas**
	Dendrobates	*nubeculosus*
	Dendrobates	*tinctorius**
	Dendrobates	*truncatus**
Dendrobates granuliferus (n = 1)	*Dendrobates*	*granuliferus**
Dendrobates pumilio (n = 8)	*Dendrobates*	*arboreus**
	Dendrobates	*histrionicus**
	Dendrobates	*lehmanni**
	Dendrobates	*occultator*
	Dendrobates	*pumilio**
	Dendrobates	*speciosus**
	Dendrobates	*sylvaticus**
	Dendrobates	*vicentei**
Dendrobates captivus (n = 2)	*Dendrobates*	*captivus**
	Dendrobates	*mysteriosus**
Dendrobates minutus (n = 12)	*Dendrobates*	*abditus*
	Dendrobates	*altobueyensis*
	Dendrobates	*bombetes**

Table S13 (Cont). Poison frog species group and taxonomic diversity assigned to the diversification rate analyses and Fig. 3. The species sampled for the analyses are indicated "*".

Group	Genera	Species
Dendrobates minutus (n = 12)	*Dendrobates*	*claudiae**
	Dendrobates	*daleswansoni*
	Dendrobates	*dorisswansonae*
	Dendrobates	*fulguritus**
	Dendrobates	*minutus**
	Dendrobates	*opisthomelas*
	Dendrobates	*tolimense*
	Dendrobates	*viridis*
	Dendrobates	*virolinensis**
Dendrobates imitator (n = 6)	*Dendrobates*	*biolat**
	Dendrobates	*lamasi**
	Dendrobates	*imitator**
	Dendrobates	*vanzolinii**
	Dendrobates	*intermedius*
	Dendrobates	*flavovittatus**
Dendrobates ventrimaculatus (n = 10)	*Dendrobates*	*amazonicus**
	Dendrobates	*duellmani**
	Dendrobates	*fantasticus**
	Dendrobates	*igneus*
	Dendrobates	*reticulatus**
	Dendrobates	*rubrocephalus*
	Dendrobates	*sirensis*
	Dendrobates	*uakarii**
	Dendrobates	*variabilis**
	Dendrobates	*ventrimaculatus**
Excluded (unknown group)	*Ameerega*	*andina*
	Ameerega	*erythromos*
	Ameerega	*labialis*
	Ameerega	*maculata*
	Anomaloglossus	*atopoglossus*
	Anomaloglossus	*lacrimosus*
	Colostethus	*lynchi*
	Hyloxalus	*edwardsi*
	Hyloxalus	*excisus*
	Hyloxalus	*parcus*
	Hyloxalus	*ruizi*
