# Supplementary material for: Amazonian Amphibian Diversity Is Primarily Derived from Late Miocene Andean Lineages
Source: PLoS Biol. 2009 Mar 10;7(3):e1000056. doi: 10.1371/journal.pbio.1000056 (PMC2653552; doi:10.1371/journal.pbio.1000056)
Supplement: Supplementary file 16 [file pbio.1000056.st014.pdf]

Table S14. Diversification rate estimates under the flexible-rate model with the lowest extinction fraction (a = 0). Node numbers correspond to Supplementary Figure S8.

Node	Clade/MRCA	Age (MYA)	*log* L	LRT	p	AIC	r	r_{CL}	λ	λ_{CL}
6	*Aromobates*	16.960	-258.4034	0.3892	0.5327	522.8068	0.1175	0.0921	0.1175	0.0921
20	*Mannophryne*	11.101	-258.5064	0.1832	0.6686	523.0128	0.1173	0.1003	0.1173	0.1003
22	*Aromobates+Mannophryne*	26.992	-258.3693	0.4574	0.4988	522.7387	0.1199	0.1073	0.1199	0.1073
31	*Rheobates*	3.135	-256.5737	4.0486	0.0442	519.1474	0.1185	0.0241	0.1185	0.0241
58	*Anomaloglossus*	19.726	-258.4993	0.1974	0.6568	522.9987	0.1174	0.1006	0.1174	0.1006
59	*Anomaloglossus+Rheobates*	28.720	-258.5902	0.0156	0.9006	523.1804	0.1171	0.1145	0.1171	0.1145
86	*Allobates talamancae*	13.485	-257.3433	2.5094	0.1132	520.6865	0.1180	0.0317	0.1180	0.0317
99	*Allobates nidicola*	15.059	-257.6433	1.9094	0.1670	521.2867	0.1177	0.0365	0.1177	0.0365
114	*Allobates kingsburyi*	10.622	-257.7726	1.6508	0.1989	521.5452	0.1176	0.0391	0.1176	0.0391
149	*Allobates femoralis*	5.432	-258.2427	0.7106	0.3992	522.4855	0.1174	0.0655	0.1174	0.0655
174	*Allobates marchesianus*	8.009	-257.7254	1.7452	0.1865	521.4507	0.1145	0.1935	0.1145	0.1935
199	*Allobates ornatus*	10.375	-258.5974	0.0012	0.9724	523.1949	0.1165	0.1178	0.1165	0.1178
214	*Allobates trilineatus*	8.833	-256.9521	3.2918	0.0696	519.9043	0.1139	0.2425	0.1139	0.2425
224	*Allobates gasconi*	3.873	-258.5955	0.0050	0.9436	523.1910	0.1166	0.1111	0.1166	0.1111
226	*A. trilineatus + A. gasconi*	9.887	-258.2959	0.6042	0.4370	522.5917	0.1079	0.1214	0.1079	0.1214
227	*A. ornatus + A. trilineatus*	11.766	-257.4327	2.3306	0.1269	520.8653	0.1140	0.1968	0.1140	0.1968
228	*A. marchesianus + A. trilineatus*	12.839	-257.3826	2.4308	0.1190	520.7652	0.1134	0.1828	0.1134	0.1828
229	*A. caeruleodactylus + A. trilineatus*	13.953	-255.9671	5.2618	0.0218	517.9342	0.1106	0.1943	0.1106	0.1943
230	*A. juanii + A. trilineatus*	15.159	-256.5800	4.0360	0.0445	519.1600	0.1110	0.1781	0.1110	0.1781
231	*A. femoralis + A. trilineatus*	16.772	-257.1272	2.9416	0.0863	520.2544	0.1116	0.1645	0.1116	0.1645
232	*A. kingsburyi + A. trilineatus*	17.745	-257.4575	2.2810	0.1310	520.9150	0.1118	0.1547	0.1118	0.1547
233	*A. nidicola + A. trilineatus*	19.008	-257.8020	1.5920	0.2070	521.6039	0.1124	0.1459	0.1124	0.1459
234	*A. talamancae + A. trilineatus*	21.836	-258.1059	0.9842	0.3212	522.2119	0.1131	0.1379	0.1131	0.1379
235	*A. undulatus + A. trilineatus*	23.753	-258.3991	0.3978	0.5282	522.7982	0.1142	0.1290	0.1142	0.1290
236	*Allobates* (without *A. alagoanus*)	25.699	-258.5701	0.0558	0.8133	523.1402	0.1156	0.1208	0.1156	0.1208
237	*Anomaloglossus+Rheobates+Allobates*	31.247	-257.7047	1.7866	0.1813	521.4093	0.1195	0.0802	0.1195	0.0802
265	*Epipedobates*	5.837	-258.5289	0.1382	0.7101	523.0577	0.1170	0.0954	0.1170	0.0954
294	*Silverstoneia*	14.884	-258.2345	0.7270	0.3939	522.4691	0.1174	0.0651	0.1174	0.0651
295	*Epipedobates+Silverstoneia*	16.874	-255.5862	6.0236	0.0141	517.1725	0.1083	0.1765	0.1083	0.1765
308	*Colostethus pratti*	5.388	-258.2053	0.7854	0.3755	522.4105	0.1172	0.0527	0.1172	0.0527
325	*Colostethus inguinalis*	11.388	-256.8978	3.4004	0.0652	519.7956	0.1132	0.2155	0.1132	0.2155

Table S14 (Cont). Diversification rate estimates under the flexible-rate model with the lowest extinction fraction (a = 0). Node numbers correspond to Supplementary Figure S8.

Node	Clade/MRCA	Age (MYA)	*log* L	LRT	p	AIC	*r*	r_{CL}	λ	λ_{CL}
326	*Colostethus 1*	13.146	-256.6427	3.9106	0.0480	519.2854	0.1117	0.1844	0.1117	0.1844
345	*Colostethus fugax*	18.807	-257.8024	1.5912	0.2072	521.6049	0.1178	0.0507	0.1178	0.0507
438	*Ameerega*	8.722	-251.8757	13.4446	0.0002	509.7514	0.1096	0.3544	0.1096	0.3544
439	*Colostethus 2+Ameerega*	21.681	-253.6445	9.9070	0.0016	513.2890	0.1102	0.2800	0.1102	0.2800
440	*Colostethus 1+Ameerega*	24.412	-257.7406	1.7148	0.1904	521.4813	0.1139	0.1713	0.1139	0.1713
441	Clade C (Colostethinae)	27.090	-258.2479	0.7002	0.4027	522.4958	0.1178	0.0822	0.1178	0.0822
458	*Phyllobates*	12.060	-257.4646	2.2668	0.1322	520.9291	0.1184	0.0538	0.1184	0.0538
472	*Dendrobates galactonotus*	10.803	-258.1077	0.9806	0.3221	522.2154	0.1176	0.0656	0.1176	0.0656
506	*Dendrobates tinctorius*	10.542	-258.3850	0.4260	0.5140	522.7699	0.1173	0.0823	0.1173	0.0823
536	*Dendrobates pumilio*	3.706	-257.2814	2.6332	0.1047	520.5629	0.1146	0.2496	0.1146	0.2496
537	*D. pumilio* complex	8.331	-256.9643	3.2674	0.0707	519.9286	0.1117	0.1725	0.1117	0.1725
538	*D. pumilio + D. tinctorius*	19.549	-258.0778	1.0404	0.3077	522.1555	0.1152	0.1806	0.1152	0.1806
539	*D.pumilio + D. galactonotus*	21.145	-258.5739	0.0482	0.8262	523.1478	0.1161	0.1247	0.1161	0.1247
561	*Dendrobates minutus*	10.085	-257.9248	1.3464	0.2459	521.8495	0.1147	0.1817	0.1147	0.1817
571	*Dendrobates imitator*	2.447	-258.3070	0.5820	0.4455	522.6139	0.1157	0.1736	0.1157	0.1736
600	*Dendrobates ventrimaculatus*	6.545	-257.3985	2.3990	0.1214	520.7970	0.1144	0.2231	0.1144	0.2231
624	*D.ventrimaculatus* complex	10.320	-256.6775	3.8410	0.0500	519.3551	0.1266	0.0910	0.1266	0.0910
625	*D.ventrimaculatus + D. minutus*	13.674	-257.1553	2.8854	0.0894	520.3107	0.1135	0.2015	0.1135	0.2015
626	*D.ventrimaculatus + D. captivus*	16.805	-256.1415	4.9130	0.0267	518.2829	0.1111	0.1972	0.1111	0.1972
626	*Dendrobates captivus*	16.805	-257.8664	1.4632	0.2264	521.7328	0.1175	0.0412	0.1175	0.0412
627	*Dendrobates*	25.451	-258.5938	0.0084	0.9270	523.1876	0.1167	0.1139	0.1167	0.1139
628	Clade D (Dendrobatinae)	29.914	-257.5709	2.0542	0.1518	521.1418	0.1111	0.1455	0.1111	0.1455
633	*Hyloxalus leucophaeus*	7.183	-257.4367	2.3226	0.1275	520.8735	0.1183	0.0494	0.1183	0.0494
638	*Hyloxalus subpunctatus*	20.574	-256.9642	3.2676	0.0707	519.9283	0.1183	0.0274	0.1183	0.0274
673	*Hyloxalus bocagei*	13.198	-258.0735	1.0490	0.3057	522.1471	0.1179	0.0709	0.1179	0.0709
674	*H. bocagei + H. subpunctatus*	25.274	-258.0438	1.1084	0.2924	522.0876	0.1209	0.0995	0.1209	0.0995
675	*H. bocagei + H. leucophaeus*	28.079	-256.8164	3.5632	0.0591	519.6328	0.1197	0.0548	0.1197	0.0548
678	*Hyloxalus pulcherrimus*	11.143	-258.1763	0.8434	0.3584	522.3527	0.1178	0.0765	0.1178	0.0765
692	*Hyloxalus nexipus*	4.852	-257.8337	1.5286	0.2163	521.6675	0.1178	0.0514	0.1178	0.0514
693	*H. anthracinus + H. nexipus*	21.360	-258.2657	0.6646	0.4149	522.5315	0.1190	0.0987	0.1190	0.0987
713	*Hyloxalus pulchellus*	10.986	-258.5270	0.1420	0.7063	523.0539	0.1169	0.0928	0.1169	0.0928

Table S14 (Cont). Diversification rate estimates under the flexible-rate model with the lowest extinction fraction (a = 0).

Node	Clade/MRCA	Age (MYA)	*log* L	LRT	p	AIC	*r*	r_{CL}	λ	λ_{CL}
714	*H. vertebralis* + *H. nexipus*	14.929	-258.4772	0.2416	0.6231	522.9544	0.1178	0.1037	0.1178	0.1037
733	*Hyloxalus azureiventris*	5.585	-258.0728	1.0504	0.3054	522.1457	0.1174	0.0473	0.1174	0.0473
734	*H. azureiventris* + *H. craspedoceps*	14.640	-258.5889	0.0182	0.8927	523.1778	0.1168	0.1115	0.1168	0.1115
735	*H. azureiventris* + *H.* sp "EL Copal"	16.198	-257.5386	2.1188	0.1455	521.0773	0.1182	0.0473	0.1182	0.0473
770	*Hyloxalus awa*	4.645	-258.5919	0.0122	0.9120	523.1837	0.1164	0.1262	0.1164	0.1262
772	*H. awa* + *H. elachyhistus*	8.703	-258.5860	0.0240	0.8769	523.1721	0.1163	0.1262	0.1163	0.1262
774	*Hyloxalus elachyhistus*	9.440	-258.5919	0.0122	0.9120	523.1837	0.1164	0.1262	0.1164	0.1262
775	*H. awa* + *H. insulatus*	11.465	-258.5966	0.0028	0.9578	523.1932	0.1165	0.1189	0.1165	0.1189
776	*H. awa* + *H. idiomelus*	13.690	-258.5809	0.0342	0.8533	523.1619	0.1162	0.1246	0.1162	0.1246
777	*H. awa* + *H. azureiventris*	18.457	-258.0381	1.1198	0.2900	522.0762	0.1183	0.0765	0.1183	0.0765
778	*H. awa* + *H. vertebralis*	22.389	-257.8890	1.4180	0.2337	521.7781	0.1180	0.0614	0.1180	0.0614
779	*H. awa* + *H. anthracinus*	23.799	-258.2305	0.7350	0.3913	522.4611	0.1182	0.0876	0.1182	0.0876
780	*H. awa* + *H. pulcherrimus*	25.429	-258.2062	0.7836	0.3760	522.4124	0.1199	0.1007	0.1199	0.1007
781	Clade B (Hyloxinae)	30.918	-256.1778	4.8404	0.0278	518.3555	0.1212	0.0588	0.1212	0.0588
782	Clades B and D ancestor	34.633	-258.1962	0.8036	0.3700	522.3925	0.1128	0.1326	0.1128	0.1326
783	Clade B, C and D ancestor	37.037	-256.7121	3.7718	0.0521	519.4242	0.1089	0.1551	0.1089	0.1551
238	Clade A (Allobatinae)	32.850	-258.3148	0.5664	0.4517	522.6296	0.1183	0.0964	0.1183	0.0964
785	Dendrobatidae	40.931	-258.2165	0.7630	0.3824	522.4330	0.1219	0.1067	0.1219	0.1067
--	*Allobates undulatus*	23.753	-255.8129	5.5702	0.0183	517.6257	0.1182	0.0001	0.1182	0.0001
--	*Allobates juanii*	15.159	-256.8252	3.5456	0.0597	519.6503	0.1176	0.0001	0.1176	0.0001
--	*Hyloxalus vertebralis*	5.821	-256.8521	3.4918	0.0617	519.7042	0.1176	0.0001	0.1176	0.0001
--	*Allobates caeruleodactylus*	13.953	-256.9667	3.2626	0.0709	519.9334	0.1175	0.0001	0.1175	0.0001
--	*Hyloxalus idiomelus*	13.690	-256.9976	3.2008	0.0736	519.9951	0.1175	0.0001	0.1175	0.0001
--	*Allobates* sp. "Neblina"	25.699	-257.3907	2.4146	0.1202	520.7813	0.1182	0.0427	0.1182	0.0427
--	*Hyloxalus* sp. "Masvalle"	2.982	-257.4958	2.2044	0.1376	520.9916	0.1172	0.0001	0.1172	0.0001
--	*Ameerega parvula*	1.502	-257.5797	2.0366	0.1536	521.1594	0.1172	0.0001	0.1172	0.0001
--	*Dendrobates granuliferus*	1.396	-257.6256	1.9448	0.1631	521.2511	0.1171	0.0001	0.1171	0.0001
--	*Hyloxalus craspedoceps*	14.640	-258.0728	1.0504	0.3054	522.1457	0.1174	0.0473	0.1174	0.0473
--	*Ameerega hahneli*	8.722	-258.2330	0.7300	0.3929	522.4661	0.1233	0.1089	0.1233	0.1089
--	*Hyloxalus* sp. "El Copal"	16.198	-258.4647	0.2666	0.6056	522.9294	0.1171	0.0856	0.1171	0.0856
--	*Hyloxalus insulatus*	11.465	-258.5607	0.0746	0.7848	523.1214	0.1167	0.0958	0.1167	0.0958
--	*Hyloxalus anthracinus*	21.360	-258.5791	0.0378	0.8458	523.1581	0.1168	0.1078	0.1168	0.1078

Table S14 (Cont). Diversification rate estimates under the flexible-rate model with the lowest extinction fraction (a = 0.99). Node numbers correspond to Supplementary Figure S8.

Node	Clade/MRCA	Age (MYA)	*log* L	LRT	p	AIC	r	r_{CL}	λ	λ_{CL}
6	*Aromobates*	16.960	-275.2743	0.0234	0.8784	556.5486	0.0033	0.0038	0.3314	0.3847
20	*Mannophryne*	11.101	-275.2005	0.1710	0.6792	556.4011	0.0033	0.0049	0.3292	0.4873
22	*Aromobates+Mannophryne*	26.992	-274.9977	0.5766	0.4476	555.9954	0.0036	0.0028	0.3599	0.2750
31	*Rheobates*	3.135	-274.1683	2.2354	0.1349	554.3367	0.0034	0.0004	0.3401	0.0367
58	*Anomaloglossus*	19.726	-275.1271	0.3178	0.5729	556.2541	0.0033	0.0055	0.3257	0.5465
59	*Anomaloglossus+Rheobates*	28.720	-274.9366	0.6988	0.4032	555.8733	0.0036	0.0026	0.3589	0.2612
86	*Allobates talamancae*	13.485	-274.3956	1.7808	0.1821	554.7912	0.0034	0.0005	0.3396	0.0466
99	*Allobates nidicola*	15.059	-274.5041	1.5638	0.2111	555.0081	0.0034	0.0005	0.3393	0.0507
114	*Allobates kingsburyi*	10.622	-274.5556	1.4608	0.2268	555.1113	0.0034	0.0006	0.3391	0.0564
149	*Allobates femoralis*	5.432	-274.9624	0.6472	0.4211	555.9248	0.0034	0.0012	0.3377	0.1191
174	*Allobates marchesianus*	8.009	-274.7596	1.0528	0.3049	555.5192	0.0032	0.0081	0.3237	0.8143
199	*Allobates ornatus*	10.375	-275.2567	0.0586	0.8087	556.5133	0.0033	0.0025	0.3345	0.2498
214	*Allobates trilineatus*	8.833	-274.5218	1.5284	0.2164	555.0436	0.0032	0.0097	0.3211	0.9660
224	*Allobates gasconi*	3.873	-275.2025	0.1670	0.6828	556.4050	0.0034	0.0020	0.3355	0.2005
226	*A. trilineatus + A. gasconi*	9.887	-275.2284	0.1152	0.7343	556.4568	0.0031	0.0035	0.3062	0.3456
227	*A. ornatus + A. trilineatus*	11.766	-274.9316	0.7088	0.3998	555.8631	0.0032	0.0059	0.3237	0.5890
228	*A. marchesianus + A. trilineatus*	12.839	-275.0123	0.5474	0.4594	556.0245	0.0032	0.0051	0.3228	0.5074
229	*A. caeruleodactylus + A. trilineatus*	13.953	-274.4049	1.7622	0.1844	554.8097	0.0031	0.0061	0.3061	0.6099
230	*A. juanii + A. trilineatus*	15.159	-274.8117	0.9486	0.3301	555.6234	0.0031	0.0050	0.3135	0.5041
231	*A. femoralis + A. trilineatus*	16.772	-275.0834	0.4052	0.5244	556.1667	0.0032	0.0043	0.3208	0.4299
232	*A. kingsburyi + A. trilineatus*	17.745	-275.1900	0.1920	0.6613	556.3801	0.0032	0.0039	0.3222	0.3941
233	*A. nidicola + A. trilineatus*	19.008	-275.2691	0.0338	0.8541	556.5383	0.0033	0.0036	0.3290	0.3572
234	*A. talamancae + A. trilineatus*	21.836	-275.2846	0.0028	0.9578	556.5693	0.0033	0.0032	0.3344	0.3240
235	*A. undulatus + A. trilineatus*	23.753	-275.2351	0.1018	0.7497	556.4703	0.0034	0.0030	0.3412	0.3003
236	*Allobates* (without *A. alagoanus*)	25.699	-275.0923	0.3874	0.5337	556.1847	0.0035	0.0028	0.3516	0.2760
237	*Anomaloglossus+Rheobates+*Allobates	31.247	-275.2756	0.0208	0.8853	556.5512	0.0033	0.0030	0.3345	0.3001
265	*Epipedobates*	5.837	-275.2353	0.1014	0.7502	556.4707	0.0034	0.0023	0.3351	0.2337
294	*Silverstoneia*	14.884	-274.9589	0.6542	0.4186	555.9177	0.0034	0.0012	0.3377	0.1183
295	*Epipedobates+Silverstoneia*	16.874	-273.0211	4.5298	0.0333	552.0422	0.0029	0.0074	0.2876	0.7429
308	*Colostethus pratti*	5.388	-274.7658	1.0404	0.3077	555.5315	0.0034	0.0007	0.3383	0.0749
325	*Colostethus inguinalis*	11.388	-274.0999	2.3722	0.1235	554.1998	0.0031	0.0113	0.3145	1.1283

Table S14 (Cont). Diversification rate estimates under the flexible-rate model with the lowest extinction fraction (a = 0.99). Node numbers correspond to Supplementary Figure S8.

Node	Clade/MRCA	Age (MYA)	*log* L	LRT	p	AIC	*r*	r_{CL}	λ	λ_{CL}
326	*Colostethus 1*	13.146	-273.8272	2.9176	0.0876	553.6543	0.0030	0.0078	0.3023	0.7808
345	*Colostethus fugax*	18.807	-274.8013	0.9694	0.3248	555.6027	0.0034	0.0009	0.3385	0.0897
438	*Ameerega*	8.722	-271.5490	7.4740	0.0063	549.0981	0.0029	0.0219	0.2927	2.1851
439	*Colostethus 2+Ameerega*	21.681	-272.9899	4.5922	0.0321	551.9797	0.0030	0.0114	0.2991	1.1443
440	*Colostethus 1+Ameerega*	24.412	-274.8202	0.9316	0.3344	555.6404	0.0032	0.0062	0.3214	0.6192
441	Clade C (Colostethinae)	27.090	-274.9908	0.5904	0.4423	555.9817	0.0034	0.0017	0.3402	0.1732
458	*Phyllobates*	12.060	-274.9793	0.6134	0.4335	555.9587	0.0034	0.0013	0.3379	0.1301
472	*Dendrobates galactonotus*	10.803	-275.0313	0.5094	0.4754	556.0626	0.0034	0.0014	0.3374	0.1377
506	*Dendrobates tinctorius*	10.542	-275.1878	0.1964	0.6576	556.3757	0.0034	0.0020	0.3359	0.2016
536	*Dendrobates pumilio*	3.706	-274.8164	0.9392	0.3325	555.6329	0.0032	0.0081	0.3232	0.8135
537	*D. pumilio* complex	8.331	-274.6038	1.3644	0.2428	555.2076	0.0031	0.0057	0.3109	0.5694
538	*D. pumilio + D. tinctorius*	19.549	-275.2459	0.0802	0.7770	556.4918	0.0033	0.0041	0.3296	0.4124
539	*D.pumilio + D. galactonotus*	21.145	-275.2828	0.0064	0.9362	556.5657	0.0033	0.0035	0.3316	0.3523
561	*Dendrobates minutus*	10.085	-274.8387	0.8946	0.3442	555.6774	0.0032	0.0076	0.3223	0.7626
571	*Dendrobates imitator*	2.447	-275.2272	0.1176	0.7317	556.4544	0.0033	0.0047	0.3299	0.4745
600	*Dendrobates ventrimaculatus*	6.545	-274.7500	1.0720	0.3005	555.5001	0.0032	0.0083	0.3242	0.8344
624	*D.ventrimaculatus* complex	10.320	-272.9643	4.6434	0.0312	551.9287	0.0041	0.0019	0.4093	0.1877
625	*D.ventrimaculatus + D. minutus*	13.674	-274.7523	1.0674	0.3015	555.5046	0.0032	0.0066	0.3199	0.6555
626	*D.ventrimaculatus + D. captivus*	16.805	-274.1298	2.3124	0.1283	554.2596	0.0031	0.0072	0.3052	0.7188
626	*Dendrobates captivus*	16.805	-274.5958	1.3804	0.2400	555.1916	0.0034	0.0006	0.3390	0.0581
627	*Dendrobates*	25.451	-275.2755	0.0210	0.8848	556.5510	0.0034	0.0031	0.3351	0.3054
628	Clade D (Dendrobatinae)	29.914	-274.8429	0.8862	0.3465	555.6859	0.0031	0.0045	0.3109	0.4549
633	*Hyloxalus leucophaeus*	7.183	-274.8593	0.8534	0.3556	555.7187	0.0034	0.0011	0.3384	0.1059
638	*Hyloxalus subpunctatus*	20.574	-274.2761	2.0198	0.1553	554.5522	0.0034	0.0004	0.3399	0.0385
673	*Hyloxalus bocagei*	13.198	-275.1682	0.2356	0.6274	556.3364	0.0034	0.0019	0.3363	0.1946
674	*H. bocagei + H. subpunctatus*	25.274	-273.8209	2.9302	0.0869	553.6417	0.0038	0.0020	0.3814	0.1996
675	*H. bocagei + H. leucophaeus*	28.079	-274.5579	1.4562	0.2275	555.1158	0.0034	0.0012	0.3436	0.1167
678	*Hyloxalus pulcherrimus*	11.143	-275.2269	0.1182	0.7310	556.4537	0.0034	0.0023	0.3354	0.2304
692	*Hyloxalus nexipus*	4.852	-274.8114	0.9492	0.3299	555.6227	0.0034	0.0009	0.3385	0.0911
693	*H. anthracinus + H. nexipus*	21.360	-273.6011	3.3698	0.0664	553.2022	0.0037	0.0017	0.3737	0.1698
713	*Hyloxalus pulchellus*	10.986	-275.1891	0.1938	0.6598	556.3782	0.0034	0.0020	0.3358	0.1964

Table S14 (Cont). Diversification rate estimates under the flexible-rate model with the lowest extinction fraction (a = 0.99).

Node	Clade/MRCA	Age (MYA)	*log* L	LRT	p	AIC	*r*	r_{CL}	λ	$λ_{CL}$
714	*H. vertebralis* + *H. nexipus*	14.929	-274.0916	2.3888	0.1222	554.1833	0.0036	0.0017	0.3620	0.1742
733	*Hyloxalus azureiventris*	5.585	-274.6936	1.1848	0.2764	555.3872	0.0034	0.0007	0.3386	0.0693
734	*H. azureiventris* + *H. craspedoceps*	14.640	-274.6445	1.2830	0.2573	555.2891	0.0035	0.0018	0.3514	0.1774
735	*H. azureiventris* + *H.* sp "EL Copal"	16.198	-274.0886	2.3948	0.1217	554.1771	0.0034	0.0007	0.3447	0.0693
770	*Hyloxalus awa*	4.645	-275.2386	0.0948	0.7582	556.4771	0.0033	0.0023	0.3349	0.2286
772	*H. awa* + *H. elachyhistus*	8.703	-275.1891	0.1938	0.6598	556.3783	0.0034	0.0023	0.3370	0.2286
774	*Hyloxalus elachyhistus*	9.440	-275.2386	0.0948	0.7582	556.4771	0.0033	0.0023	0.3349	0.2286
775	*H. awa* + *H. insulatus*	11.465	-274.9742	0.6236	0.4297	555.9483	0.0034	0.0019	0.3417	0.1888
776	*H. awa* + *H. idiomelus*	13.690	-274.9783	0.6154	0.4328	555.9566	0.0034	0.0021	0.3435	0.2076
777	*H. awa* + *H. azureiventris*	18.457	-274.4418	1.6884	0.1938	554.8837	0.0035	0.0013	0.3485	0.1274
778	*H. awa* + *H. vertebralis*	22.389	-274.4802	1.6116	0.2043	554.9603	0.0034	0.0010	0.3434	0.0978
779	*H. awa* + *H. anthracinus*	23.799	-275.2160	0.1400	0.7083	556.4320	0.0034	0.0025	0.3369	0.2490
780	*H. awa* + *H. pulcherrimus*	25.429	-273.8109	2.9502	0.0859	553.6218	0.0038	0.0019	0.3781	0.1943
781	Clade B (Hyloxinae)	30.918	-274.3028	1.9664	0.1608	554.6056	0.0035	0.0013	0.3502	0.1254
782	Clades B and D ancestor	34.633	-275.0154	0.5412	0.4619	556.0309	0.0031	0.0042	0.3132	0.4164
783	Clade B, C and D ancestor	37.037	-273.7471	3.0778	0.0794	553.4942	0.0029	0.0059	0.2890	0.5928
238	Clade A (Allobatinae)	32.850	-275.2001	0.1718	0.6785	556.4001	0.0033	0.0044	0.3260	0.4357
785	Dendrobatidae	40.931	-275.1987	0.1746	0.6761	556.3975	0.0035	0.0030	0.3502	0.3017
--	*Allobates undulatus*	23.753	-273.3511	3.8698	0.0492	552.7022	0.0034	0.0001	0.3407	0.0146
--	*Allobates juanii*	15.159	-273.6564	3.2592	0.0710	553.3127	0.0034	0.0001	0.3401	0.0146
--	*Hyloxalus vertebralis*	5.821	-273.6667	3.2386	0.0719	553.3335	0.0034	0.0001	0.3401	0.0146
--	*Allobates caeruleodactylus*	13.953	-273.7128	3.1464	0.0761	553.4255	0.0034	0.0001	0.3400	0.0146
--	*Hyloxalus idiomelus*	13.690	-273.7257	3.1206	0.0773	553.4514	0.0034	0.0001	0.3400	0.0146
--	*Hyloxalus* sp. "Masvalle"	2.982	-273.9746	2.6228	0.1053	553.9493	0.0034	0.0001	0.3394	0.0146
--	*Ameerega parvula*	1.502	-274.0262	2.5196	0.1124	554.0524	0.0034	0.0001	0.3393	0.0146
--	*Dendrobates granuliferus*	1.396	-274.0559	2.4602	0.1168	554.1118	0.0034	0.0001	0.3392	0.0146
--	*Ameerega hahneli*	8.722	-274.5877	1.3966	0.2373	555.1753	0.0040	0.0028	0.3988	0.2751
--	*Allobates* sp. "Neblina"	25.699	-274.6813	1.2094	0.2715	555.3626	0.0034	0.0008	0.3390	0.0762
--	*Hyloxalus craspedoceps*	14.640	-274.6936	1.1848	0.2764	555.3872	0.0034	0.0007	0.3386	0.0693
--	*Hyloxalus insulatus*	11.465	-275.1492	0.2736	0.6009	556.2984	0.0034	0.0017	0.3362	0.1734
--	*Hyloxalus* sp. "El Copal"	16.198	-275.1580	0.2560	0.6129	556.3160	0.0034	0.0018	0.3362	0.1823
--	*Hyloxalus anthracinus*	21.360	-275.2670	0.0380	0.8454	556.5339	0.0033	0.0040	0.3311	0.4044
