# Supplementary material for: Correction: Amazonian Amphibian Diversity Is Primarily Derived from Late Miocene Andean Lineages
Source: PLoS Biol. 2010 Sep 13;8(9):10.1371/annotation/18e722e3-05db-4e40-86a0-bedfc4934a5a. doi: 10.1371/annotation/18e722e3-05db-4e40-86a0-bedfc4934a5a (PMC2938366; doi:10.1371/annotation/18e722e3-05db-4e40-86a0-bedfc4934a5a)
Supplement: Supplementary file 1 [file pbio.18e722e3-05db-4e40-86a0-bedfc4934a5a.s001.doc]

**Supplementary Text:**

**Corrections to Taxonomy**

Our phylogenetic analysis found results generally similar to previous phylogenetic hypotheses [22,23]. For the purpose of this paper, some corrections to the Grant *et al.*’s [23] taxonomy must be made in order to have a nomenclature concordant with well-supported clades, and to avoid ambiguity and subsequent confusion in the literature of this family.

The monophyly of Dendrobatidae (*sensu* Noble 1926) [114] has always been strongly supported (see Figure S3) by previous studies [22,23]. The taxonomic split of Dendrobatidae into two families (i.e., Aromobatidae and Dendrobatidae) is unnecessary and adds no new information. Grant *et al.*’s [23] primary reason for the split is the inability to sequester alkaloids (i.e., chemical defense) in Allobatinae or Clade A (Aromobatidae *sensu* Grant *et al*.). Very few species of Aromobatidae have been documented as being unable to sequester alkaloids, and many species of Colostethinae (Clade C) and Hyloxalinae (Clade B) are unable to do so [115,116]. The restriction of Dendrobatidae to include only Colostethinae, Dendrobatinae, and Hyloxinae (Clade C+B+D) is rejected here, because (1) some of the putative synapomorphies are poorly defined, (2) the characterization of some Colostethinae and Hyloxinae as not able to sequester alkaloids is unfounded, because the chemical profile of the vast majority of species is lacking, (3) the alignment and phylogenetic analysis methods used are dubious [117], and (4) repeated mistakes in their molecular dataset were found (see below). For these reasons, the proposed split is not followed and we return Dendrobatidae to a single family (i.e., Clade A+B+C+D) that includes all members of both of the Grant *et al*.’s Dendrobatidae and Aromobatidae.

Grant *et al*. [23] redefined *Colostethus* to be monophyletic*,* but did not include the type species *Colostethus latinasus* in their analysis. We found clear paraphyly of Grant *et al.*'s [23] redefined *Colostethus* (indicated by *Colostethus* 1 and 2, each with high support see Figure S3), which paradoxically still renders *Colostethus* paraphyletic even after all the proposed taxonomic rearrangements to remove paraphyly. For the moment, we restrict *Colostethus* to the group of *C. latinasus* and allies (species in *Colostethus* 1 clade). *Colostethus* sensu lato is applied to the aggregate of species found in *Colostethus* 1 and 2 clades in our phylogeny.

Third, *Dendrobates* sensu lato (including *Adelphobates*, *Dendrobates*, *Excidobates*, *Minyobates, Oophaga,* and *Ranitomeya*) was found to be a well-supported monophyletic group (as it was previously and see Figure S3); thus the splitting of *Dendrobates* into several genera is unnecessary (e.g., *Excidobates* [118]) and Grant *et al*. did not provide an unambiguous list of synapomorphies for their generic concepts. Some of the putative synapomorphies for genera used by Grant *et al*. [23] are ambiguous, and others are problematic; These include those with sequence errors (see below), those that are polymorphic intraspecifically (coloration pattern and alkaloid presence), poorly defined (e.g., advertisement call type), or undetermined in most species (e.g., larvae morphology, chromosome number, alkaloid profile, and type of parental care). Therefore, we synonymize *Adelphobates*, *Excidobates*, *Minyobates, Oophaga,* and *Ranitomeya* in the genus *Dendrobates*.

Other minor changes are: (1) *Allobates craspedoceps* was found within *Hyloxalus*, so a new combination is proposed: *Hyloxalus craspedoceps*; (2) *Hyloxalus argyrogaster* was found with *Colostethus* sensu lato, so its new combination is *Colostethus argyrogaster*, and (3) we were able to find remnant populations of *Allobates peruensis* (closely allied to *A. kingsburyi*); thus it is a valid name and not a *nomen dubium* as proposed by Grant *et al*. [23].

Some discrepancies between our results and those of Grant *et al*. [23] are explainable by mistakes in their molecular dataset. One set of mistakes possibly came from contamination. For example, the cytochrome oxidase (COX) sequence of *Allobates brunneus* (GenBank DQ502910) is identical by BLAST search to the paper wasp *Polistes dominulus* (GenBank DQ172914); similarly, the COX sequence of *Allobates femoralis* (GenBank DQ502733) is identical to Mediterranean sand smelt fish *Atherina hepsetus* (GenBank AY290810). Apparently, some tube mislabeling also occurred; as one example, the RAG-1 sequence of *Colostethus fraterdanieli* (GenBank DQ503373) is identical to *Phyllobates bicolor* (GenBank DQ503377) and not to other *C. fraterdanieli* RAG-1 sequences of the same population (GenBank DQ503371-2 and DQ503375). Although we used some sequences from Grant *et al*. [23] in our analysis, none yielded anomalous results. However, the possibility that other sequences not used by us might come from misidentified specimens remains. For these reasons, we suggest the sequences in Grant *et al*. [23] require thorough vetting through BLAST (<http://www.ncbi.nlm.nih.gov/BLAST/>) and other means.

**Supporting Literature**

114. Noble GK (1926) The pectoral girdle of the brachycephalid frogs. Am Mus Novit 230: 1-14.

115. Myers CW, Paolillo O. A, Daly JW (1991) Discovery of a defensively malodorous and nocturnal frog in the family Dendrobatidae: phylogenetic significance of a new genus and species from the venezuelan Andes. Am Mus Novit 3002: 1-33.

116. Daly JW, Gusovsky F, Myers CW, Yotsu-Yamashita M, Yasumoto T (1994) First occurrence of tetrodotoxin in a dendrobatid frog (*Colostethus* *inguinalis*), with further reports for the bufonid genus *Atelopus*. Toxicon 32: 279-285.

117. Wiens JJ (2007) Review of "The amphibian tree of life" by Frost et al. Q Rev Biol 82: 55–56.

118. Twomey E, Brown JL (2008) Spotted poison frog: rediscovery of the lost species and a new genus (Anura: Dendrobatidae) from Northwestern Peru. Herpetologica 64: 121-137.

119. Van Tuinen M, Hadly EA (2004) Error in estimation of rate and time inferred from the early amniote fossil record and avian molecular clocks. J Mol Evol 59: 267-276.

120. Benton MJ, Donoghue PCJ (2007) Paleontological evidence to date the tree of life. Mol Biol Evol 24: 26-53.

121. Graur D, Martin W (2004) Reading the entrails of chickens: molecular timescales of evolution and the illusion of precision. Trends Genet 20: 80-86.

122. Rage J-C, Rocek Z (1989) Redescription of *Triadobatrachus* *massinoti* (Piveteau, 1936) an anuran amphibian from the early Triassic. Palaeontographica Abt A 206: 1-16.

123. Estes R (1970) New fossil pelobatid frogs and a review of the genus *Eopelobates*. Bull Mus Comp Zool 139: 293-340.

124. Estes R, Sanchiz B (1982) New discoglossid and palaeobatrachid frogs from the late Cretaceous of Wyoming and Montana, and a review of other frogs from the Lance and Hell Creek formations. J Vertebr Paleontol 2: 9-20.

125. Báez AM, Peri SI (1989) *Baurubatrachus pricei* nov. ge. et sp., un anuro del Cretacico superior de Minas Gerais, Brasil. An Acad Bras Cienc 61: 447-458.

126. Henrici AC (1998) A new pipoid anuran from the Late Jurassic Morrison Formation at Dinosaur National Monument, Utah. J Vertebr Paleontol 18: 321-332.

127. Pitman WC, Cande S, LaBrecque J, Pindell JL (1993) Fragmentation of Gondwana: the separation of Africa from South America. In: Goldblatt P, editor. Biological Relationships Between Africa and South America. New Haven, Connecticut: Yale University Press. pp. 15-34.

128. Rogers JJW (1996) A history of continents in the past three billion years. J Geol 104: 91-107.

129. Evans SE, Milner AR, Mussett F (1990) A discoglossid frog from the Middle Jurassic of England. Palaeontology 33: 299-311.

130. Rocek Z (2000) Mesozoic anurans. In: Heatwole H, Carroll RL, editors. Amphibian Biology Volume 4. Chipping Norton: Surrey Beatty & Sons. pp. 1295-1331.

131. MacFadden BJ, Anaya F, Argollo J (1993) Magnetic polarity stratigraphy of Inchasi: A Pliocene mammal-bearing locality from the Bolivian Andes deposited just before the Great American Interchange. Earth Planet Sci Lett 114: 229-241.

132. Hoorn C, Guerrero J, Sarmiento GA, Lorente MA (1995) Andean tectonics as a cause for changing drainage patterns in Miocene northern South America. Geology 23: 237-240.

133. Coates AG (1997) The forging of Central America. In: Coates AG, editor. Central America: A Natural and Cultural History. New Haven: Yale University Press. pp. 1-37.

134. Hansson PF, Fisher AG (1986) Obervations on the Neogene of northwestern Ecuador. Micropaleontology 32.

135. Miller W, Vokes E (1998) Large *Phymatoderma* in Pliocene slope deposits, northwestern Ecuador: associated ichnofauna, fabrication, and behavioral ecology. Ichnos 6: 23-45.

136. Cronin TM, Dowsett HJ (1996) Biotic and oceanographic response to the Pliocene closing of the Central American Isthmus. In: Jackson JBC, Budd AF, Coates AG, editors. Evolution and Environment in Tropical America. Chicago: The University of Chicago Press.

137. Webb SD, Rancy A (1996) Late Cenozoic evolution of the neotropical mammal fauna. In: Jackson JBC, Budd AF, Coates AG, editors. Evolution and Environment in Tropical America. Chicago, IL: The University of Chicago Press. pp. 595-680.

138. Orgeira MJ (1990) Paleomagnetism of Late Cenozoic fossiliferous sediments from Barranca de Los Lobos (Buenos Aires Province, Argentina): The magnetic age of the South American land mammal ages. Phys Earth Planet Int 64: 121-132.

139. Reguero MA, Candela AM, Alonso RN (2007) Blochronology and blostratigraphy of the Uquia Formation (Pliocene-early Pleistocene, NW Argentina) and its significance in the Great American Biotic Interchange. J S Am Earth Sci 23: 1-16.

140. Collins LS, Budd AF, Coates AG (1996) Earliest evolution associated with closure of the tropical American seaway. Proc Nat Acad Sci USA 93: 6069-6072.

141. Beu AG (2001) Gradual Miocene to Pleistocene uplift of the Central American isthmus: Evidence from tropical American tonnoidean gastropods. J Paleontol 75: 706-720.

142. Harrington HJ (1962) Paleogeographic development of South America. Am Assoc Pet Geol Bull 46: 1773–1814.

143. Mégard F (1992) The evolution of the Pacific Ocean margin in South America north of Arica elbow (18° S). In: Ben-Avraham Z, editor. Evolution of the Pacific Ocean Margins. New York: Oxford University Press. pp. 208–230.

144. Webb SD (1978) A history of savanna vertebrates in the New World. Annu Rev Ecol Syst 9: 393-426.

145. Dick CW, Abdul-Salim K, Bermingham E (2003) Molecular systematics reveals cryptic Tertiary diversification of a widespread tropical rainforest tree. Am Nat 162: 691-703.

146. Weigt LA, Crawford AJ, Rand AS, Ryan MJ (2005) Biogeography of the tungara frog, *Physalaemus pustulosus*: a molecular perspective. Mol Ecol 14: 3857-3876.

147. Zeh JA, Zeh DW, Bonilla MM (2003) Phylogeography of the harlequin beetle-riding pseudoscorpion and the rise of the Isthmus of Panama. Mol Ecol 10: 2759-2769.

148. Marko PB (2002) Fossil calibration of molecular clocks and the divergence times of geminate species pairs separated by the Isthmus of Panama. Mol Biol Evol 19: 2005-2021.

149. Gardner TW, Back W, Bullard TF, Hare PW, Kesel RH, et al. (1987) Central America and the Caribbean. In: Graf WL, editor. Geomorphic systems of North America. Boulder, CO: Geological Society of America, Centennial Special Volume 2. pp. 343–402.

150. Grafe K, Frisch W, Villa IM, Meschede M (2002) Geodynamic evolution of southern Costa Rica related to low-angle subduction of the Cocos Ridge: constraints from thermochronology. Tectonophysics 348: 187-204.

151. Wuster W, Ferguson JE, Quijada-Mascarenas A, Pook CE, Salomao M, et al. (2005) Tracing an invasion: landbridges, refugia, and the phylogeography of the Neotropical rattlesnake (Serpentes: Viperidae: *Crotalus durissus*). Mol Ecol 14: 1095-1108.

152. Hughes C, Eastwood R (2006) Island radiation on a continental scale: Exceptional rates of plant diversification after uplift of the Andes. Proc Nat Acad Sci USA 103: 10334-10339.

153. Maxson LR, Myers CW (1985) Albumin evolution in tropical poison frogs (Dendrobatidae): a preliminary report. Biotropica 17: 50-56.

154. Hartley AJ (2003) Andean uplift and climate change. J Geol Soc London 160: 7-10.

155. Mann P, Schubert C, Burke K (1990) Review of Caribbean neotectonics. In: Dengo G, Case JE, editors. The geology of North America, volume H The Caribbean region. Boulder: The Geological Society of America. pp. 307-338.

156. Pindell JL, Barrett SF (1990) Geological evolution of the Caribbean region; a plate tectonic perspective. In: Dengo G, Case JE, editors. The geology of North America, volume H The Caribbean region. Boulder: The Geological Society of America. pp. 405–432.

157. Lundberg JG, Chernoff B (1992) A Miocene fossil of the Amazonian fish *Arapaima* (Teleostei, Arapaimidae) from the Magdalena River region of Colombia-Biogeographic and evolutionary implications. Biotropica 24: 2-14.

158. Brumfield RT, Capparella AP (1996) Historical diversification of birds in northwestern South America: a molecular perspective on the role of vicariant events. Evolution: 1607–1624.

159. Nores M (2004) The implications of Tertiary and Quaternary sea level rise events for avian distribution patterns in the lowlands of northern South America. Glob Ecol Biogeogr 13: 149-161.

160. Lundberg JG, Marshall LG, Guerrero J, Horton B, Malabarba MCSL, et al. (1998) The stage for Neotropical fish diversification: A history of tropical South American rivers. In: Malabarba LR, Reis RE, Vari RP, Lucena ZMS, Lucena CAS, editors. Phylogeny and Classification of Neotropical Fishes. Porto Alegre, Brazil: Editora Universitária PUCRS. pp. 13-48.

161. Campbell KE, Frailey CD, Romero-Pittman L (2006) The Pan-Amazonian Ucayali Peneplain, late Neogene sedimentation in Amazonia, and the birth of the modern Amazon River system. Palaeogeog Palaeoclim Palaeoecol 239: 166-219.

162. Mégard F (1984) The Andean orogenic period and its major structures in central and northern Peru. 141: 893–900.

163. Mégard F (1987) Structure and evolution of the Peruvian Andes. In: Schaer JP, Rodgers J, editors. The Anatomy of Mountain Ranges. Princeton: Princeton University Press. pp. 179–210.

164. Sébrier M, Lavenu A, Fornari M, Soulas J-P (1988) Tectonics and uplift in Central Andes (Peru, Bolivia and Northern Chile) from Eocene to present. Géodynamique 3 (1/2): 85–106.

165. Steinmann M, Hungerbühler D, Seward D, Winkler W (1999) Neogene tectonic evolution and exhumation of the southern Ecuadorian Andes; a combined stratigraphy and fission-track approach. Tectonophysics 307: 255–276.
